# Structural Transformation of a BRAF Inhibitor into a Selective PKR Inhibitor

**DOI:** 10.1021/acs.jmedchem.5c03664

**Published:** 2026-06-22

**Authors:** Jay Yin, Smriti Srivastava, Xiaojing Tang, Caleb Galbraith, Oro Uchenunu, Joshua Miller, Yuwei Liu, Isabella Crescenzi, Taira Kiyota, Igor Kurinov, Mauro Costa-Mattioli, Radek Laufer, Ahmed Aman, Robert Rottapel, Jailall Ramnauth, Diane L. Haakonsen, David E. Uehling, Frank Sicheri

**Affiliations:** † Lunenfeld-Tanenbaum Research Institute, Sinai Health System, Toronto, Ontario M5G 1X5, Canada; ‡ Department of Biochemistry, University of Toronto, Toronto, Ontario M5S 1A8, Canada; § Department of Molecular Genetics, University of Toronto, Toronto, Ontario M5S 1A8, Canada; ∥ Drug Discovery Program, 90775 Ontario Institute for Cancer Research, MaRS Centre, , Toronto, Ontario M5G 0A3, Canada; ⊥ Princess Margaret Cancer Centre, University Health Network, Toronto, Ontario M5G 1L7, Canada; # Departments of Medicine, Medical Biophysics and Immunology, University of Toronto, Toronto, Ontario M5G 1L7, Canada; ¶ Division of Rheumatology, St Michael’s Hospital, Toronto, Ontario M5B 1W8, Canada; ∇ Neuroscience, 3989 Baylor College of Medicine, One Baylor Plaza, Houston, Texas 77030, United States; ○ NE-CAT APS, Building 436E, Argonne National Laboratory, 9700 S. Cass Avenue, Argonne, Illinois 60439, United States; ⧫ Leslie Dan Faculty of Pharmacy, University of Toronto, Toronto, Ontario M5S 3M2, Canada

## Abstract

The RNA-dependent protein kinase PKR regulates responses to viral infection and has emerging roles in memory formation. Inhibition of PKR enhances long-term memory in mice and reverses cognitive decline in models of aging and Alzheimer’s disease. However, existing PKR inhibitors have poor selectivity and pharmacokinetic properties, limiting therapeutic development. Here, we describe the transformation of dabrafenib, an FDA-approved oncogenic BRAF inhibitor, into a selective PKR inhibitor. Dabrafenib was identified by screening as a promising PKR lead with similar potency against BRAF and PKR. Guided by X-ray cocrystal structures, we introduced modifications that removed BRAF while retaining PKR inhibition. This optimization yielded **OICR-403184**, which shows markedly reduced BRAF activity, improved PKR selectivity (IC_50_ > 10,000 nM against BRAF vs IC_50_ = 263 nM against PKR *in vitro*), and minimal activity against related eIF2α kinases in cells. These findings establish **OICR-403184** as a promising chemical starting point for further PKR inhibitor optimization.

## Graphical abstract

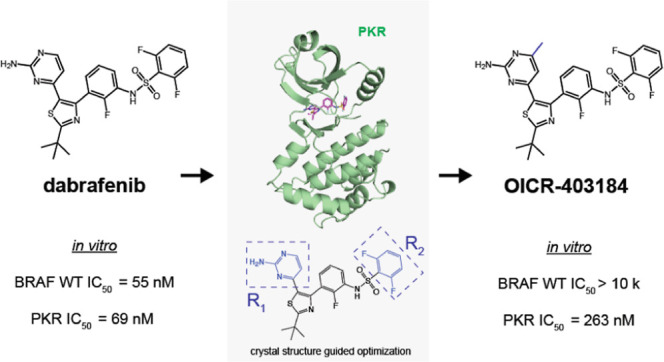

## Introduction

The RNA-dependent protein kinase PKR is a stress sensor that signals by phosphorylating the translation initiation factor eIF2α to regulate protein synthesis. It is best characterized for its role in mediating a cellular antiviral response.^1^ Three additional eIF2α kinases are found in vertebrates, namely, GCN2, PERK, and HRI, which respond to distinct cues, namely, nutrient limitation, unfolded protein in the endoplasmic reticulum, and mitochondrial stress, respectively.^2^ The eIF2α kinases detect their respective stress cues through diverging regulatory domains and yet phosphorylate a common substrate, the translation initiation factor eIF2α subunit, by use of a bilobal protein kinase domain that is highly conserved across the eIF2α kinase family.

The domain architecture of PKR consists of two N-terminal double-stranded RNA-binding domains and a C-terminal protein kinase domain.^1^ Binding of viral double-stranded RNA to the two N-terminal RNA-binding domains during viral infection activates PKR kinase activity by promoting the dimerization of the protein kinase domain and, in response, autophosphorylation of Thr446 within the kinase activation segment.^3–5^ An extended and laterally displaced αG helix in the C-lobe of the protein kinase domain, which is uniquely conserved in the eIF2α kinase family, confers specificity to its primary substrate eIF2α.^4^

EIF2 is a GTP-dependent translation initiation factor composed of eIF2α, eIF2β, and eIF2γ subunits. GTP-bound eIF2 forms an active ternary complex with initiator methionyl tRNA. Upon delivery of the initiator methionyl tRNA to the ribosome translation initiation complex, GTP is hydrolyzed and inactive eIF2 in its GDP-bound state is released. Reactivation of eIF2 to a GTP-bound state is carried out by the guanine nucleotide exchange factor eIF2B. Phosphorylation of eIF2α on Ser51 by PKR and other eIF2α kinases converts a transient interaction of eIF2 with the nucleotide exchange factor eIF2B into a tight sequestering interaction that inhibits nucleotide exchange.^6–10^ Inhibition of eIF2B in turn reduces the level of active eIF2, which inhibits overall protein synthesis while selectively inducing expression of stress response genes such as the transcription factors ATF4 and CHOP.^11^ In the case of viral infection, activation of PKR leads to the inhibition of both viral and host protein synthesis and induces apoptosis of infected cells as part of the host immune response. In the case of nutrient limitation, mitochondrial stress, or unfolded protein stress in the ER, activation of GCN2, HRI, and PERK, respectively, inhibits overall protein synthesis to allow the cell to resolve the offending stress or to undergo apoptosis if the stress is too severe to be resolved.

PKR also promotes inflammation as part of the cell’s immune response to invading pathogens.^12^ Chronic inflammation, however, is disadvantageous to a host and can contribute to adverse conditions such as inflammatory bowel disease (IBD), including Crohn’s disease and ulcerative colitis. IBD is a highly prevalent condition affecting ∼5 million people worldwide with symptoms that include intestinal pain and anemia of varying severity. Notably, PKR was shown to promote inflammation in a mouse model of colitis, while its knockdown or chemical inhibition reduced the expression of pro-inflammatory genes such as nitric oxide synthase.^13^

Recent work has linked PKR kinase activity to an expanding repertoire of brain-related functions and diseases beyond those related to the detection of viral infection and inflammation. Owing to the dependency of long-term memory on protein synthesis, PKR and the eIF2α kinases have emerged as key regulators of this process. Indeed, genetic knock out of PKR leads to improved learning and memory in mice models,^14,15^ and elevated PKR activity is associated with cognitive decline and memory impairment in Alzheimer’s, Huntington’s, and other age-related neurodegenerative disorders.^16,17^ The improvement in learning and memory upon PKR inhibition, the association of PKR activity with age-related neurological disorders, PKR’s link to inflammation, and the druggable nature of protein kinases in general identify PKR as an attractive potential target for therapeutic intervention.

To date, few highly selective and potent protein kinase inhibitors have been described for PKR. For example, C16 is an early described inhibitor of PKR with moderate ability to inhibit PKR autophosphorylation *in vitro* (IC_50_ = 210 nM).^18^ While shown to reduce intestinal inflammation in an IBD mice model and to improve memory in an Alzheimer’s mice model,^14^ its low selectivity confounds its use as a molecular probe of PKR biology.^19,20^ In particular, while C16 can protect neuronal cells from apoptosis, this effect appeared independent of PKR^19^ and instead may be a result of off-target inhibition of CDK2.

More recent efforts have led to inhibitors with improved potency and selectivity toward PKR.^20–22^ Notably, Lopez-Grancha et al.^21^ reported the highly selective and potent PKR inhibitor SAR439883 and used it to explore therapeutic utility in Alzheimer’s disease. SAR439883 inhibits PKR with an IC_50_ of 30 nM *in vitro* and an IC_50_ of 179 nM in cells. In addition, SAR439883 displays considerable selectivity for PKR over other eIF2α kinases; while exhibiting only 2-fold selectivity over GCN2 *in vitro* (IC_50_ = 62 nM), SAR439883 displays greater than 35-fold selectivity over GCN2 in cells (IC_50_ = 6300 nM) with no detectable activity against PERK and HRI at up to 30 μM concentrations. Profiling of SAR439883 against a panel of 304 protein kinases and 148 receptor/ion channels revealed cross-reactivity only with CDK9 (IC_50_ of 965 nM) and with 30-fold weaker potency than against PKR *in vitro*. Importantly, SAR439883 is brain penetrant and has been shown to restore cognitive function and prevent cognitive decline in a mouse model of Alzheimer’s disease. The only limitation of SAR439883 may be its rapid clearance (half-life = 2.6 h), which could necessitate a less-than-ideal dosing regimen in preclinical studies.

Despite the apparent cleanliness of SAR439883, additional PKR inhibitors, preferably with distinct chemical scaffolds, would be valuable for orthogonal validation as they can help confirm target-specific effects, distinguish true on-target activity from off-target liabilities, and deconvolute the mechanisms underlying unexpected toxicity or efficacy. To this end, we report the development of a selective inhibitor of PKR based on dabrafenib, a non-brain penetrant drug originally developed to target oncogenic forms of BRAF.^23–25^ As dabrafenib has entered the clinic with good safety and PK/PD profiles, our findings identify an attractive starting point for further optimization of a PKR targeting tool compound for studying non-neurological conditions.

## Results

To identify alternative chemical starting points for the development of a potent and selective inhibitor of PKR catalytic activity, we computationally screened the kinase domain of PKR against a library of 647 kinase inhibitors from the Ontario Institute for Cancer Research (OICR) kinase inhibitor collection using the open-source biomolecular interaction foundation model Boltz2 (Figure 1a; see Table S1, for screen output). This virtual screen identified numerous hits, with the strongest 15 hits displaying promising affinity probability binary scores greater than 0.75 and affinity prediction value scores < −1.25. Based on binding scores, commercial availability, and the availability of previously solved cocrystal structures bound to other kinase targets, we selected four molecules (highlighted by red arrows in Figure 1a, inset) to profile for inhibitory activity against PKR *in vitro* at Eurofins. The selected compounds and their Boltz2 scores were PIK-75 (0.95/–2.38), gilteritinib (0.80/–1.90), A-443654 (0.91/–1.86), and dabrafenib (0.77/–1.45). Notably the binding mode of all four molecules to their cognate kinase targets closely matched the binding modes to PKR predicted by Boltz2, with RMSD values of 0.7, 2.0, 0.6, and 0.9 Å for PIK75, gilteritinib, A-443654, and dabrafenib, respectively (Figure 1b). We found that all four molecules inhibited PKR with moderate to high potency *in vitro* (Figure 1b and Table 1), with IC_50_ values of 12, 69, 233, and 570 nM, respectively, for the PI3K inhibitor PIK-75, the mutant BRAF inhibitor dabrafenib, the pan-AKT inhibitor A-443654, and the FLT3 inhibitor gilteritinib. Three of the four inhibitors also showed potent activity against PERK but modest activity against GCN2 and HRI (Table 1). Given its potency against PKR *in vitro* and its favorable PK/PD properties as an approved drug to treat melanoma, we prioritized dabrafenib as a starting point for developing a potent and selective PKR inhibitor. Interestingly, we tested 4 other known BRAF inhibitors, namely, encorafenib,^26^ PLX7904,^27^ XP-102,^28^ and LXH254,^29^ and none of these showed any activity against PKR (Table 1).

**Figure 1:**
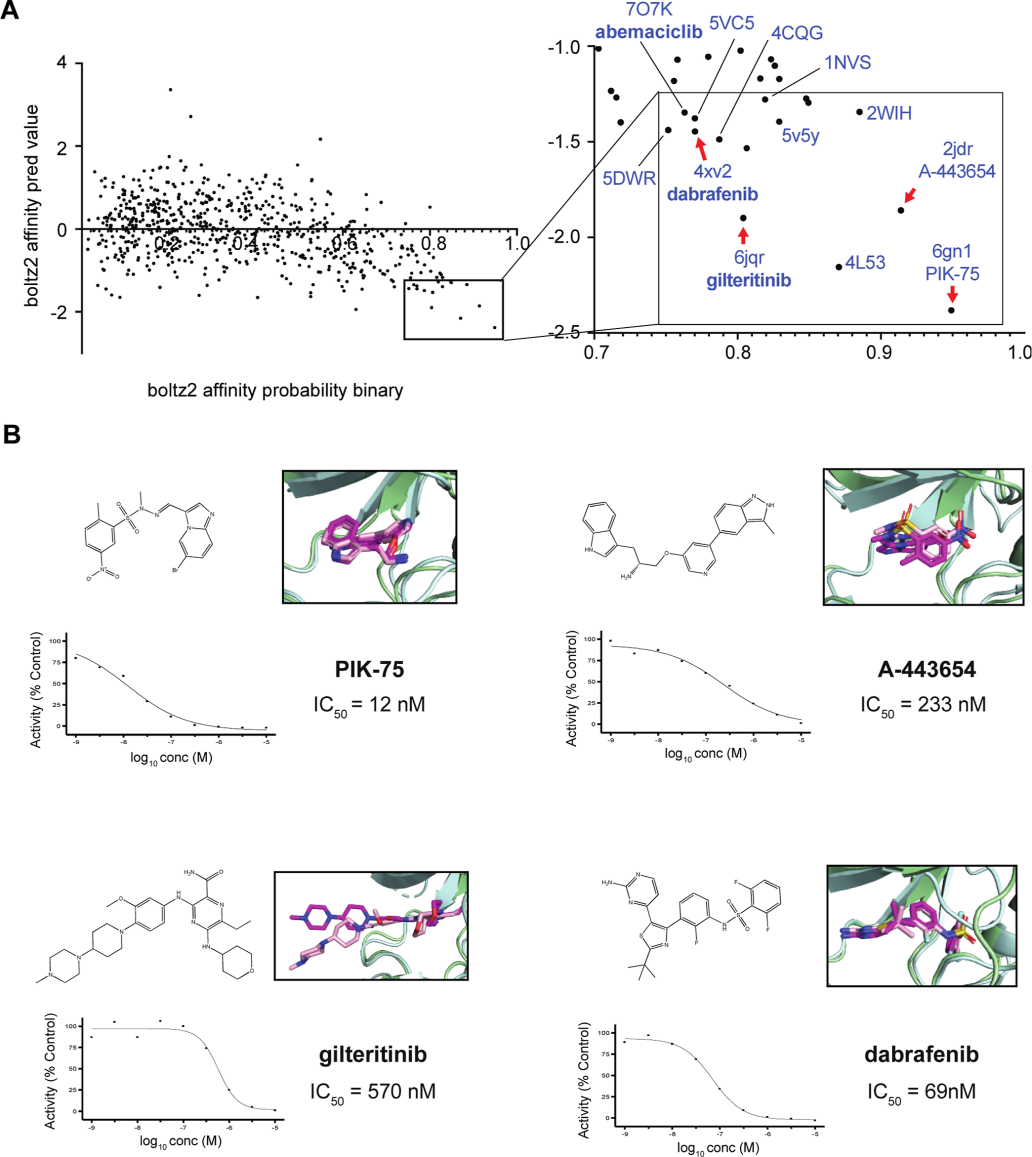
Summary of Boltz2 kinase inhibitor library screen. (A) Molecules were sorted by predicted affinity probability binary and affinity pred value. Inset shows top 15 candidate molecules (predicted affinity probability binary and affinity pred value better than 0.75 and −1.25). The PDB codes of structures of molecules solved in complex with their target kinase are shown. The names of the approved drugs are shown in bold. Four molecules selected for *in vitro* validation are indicated by red arrows. (B) Chemical structure, overlay of modeled structure bound to PKR vs X-ray crystal structure bound to target kinase, and *in vitro* IC_50_ values against PKR for PIK-75, A-443654, gilteritinib, and dabrafenib inhibitors (PDB 6GN1, 2JDR, 6JQR, and 4XV2, respectively).

**Table 1:**
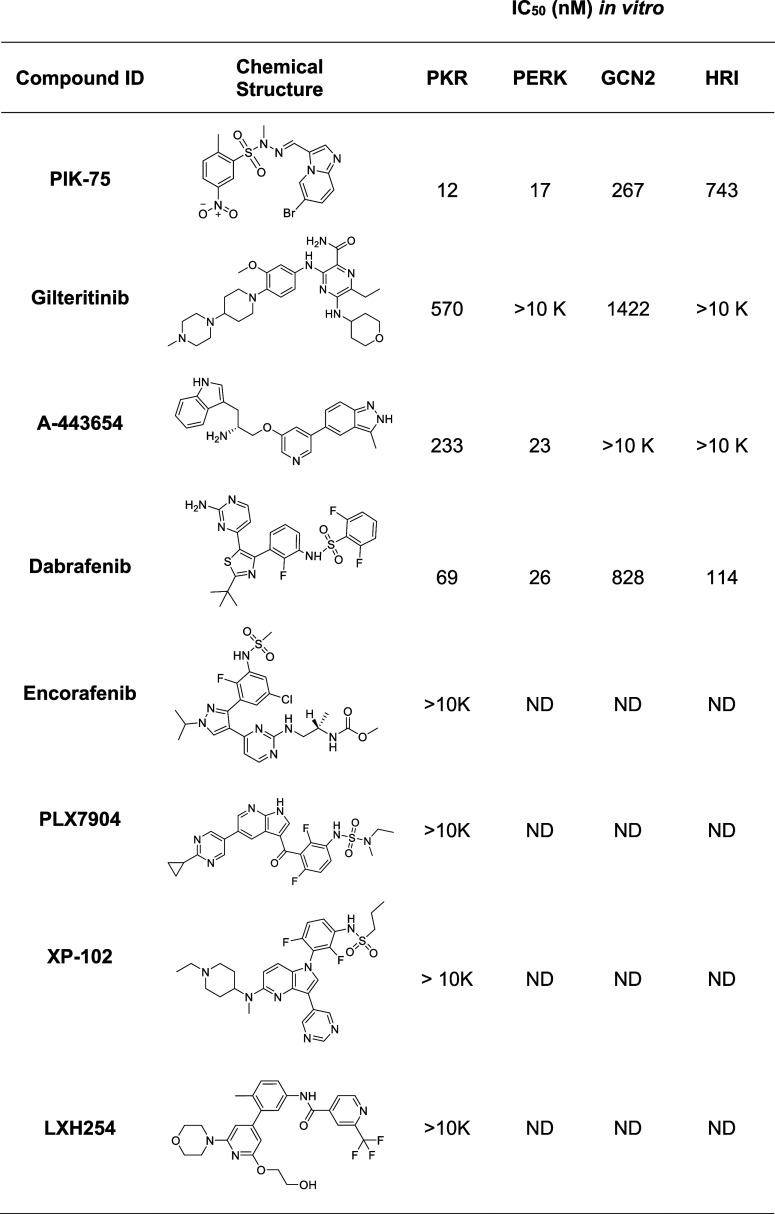
*In Vitro* Kinase Profiling of Boltz2 Screen Hits and BRAF Inhibitors at Eurofins (Associated with Figure 1)­^a^

a ND = not determined.

Dabrafenib is a highly potent and selective inhibitor of BRAF approved for the treatment of melanoma driven by the activating valine 600 to glutamate (V600E) mutation.^23,25^ Activating BRAF mutations drive cancer growth by activating the RAS-RAF-MEK-ERK signaling pathway through enhanced phosphorylation of MEK. Activated MEK in turn phosphorylates and activates ERK, which phosphorylates a wide range of targets involved in promoting cell growth, proliferation, differentiation, survival, and migration.^30^ Dabrafenib inhibits oncogenic BRAF^V600E^ enzyme activity *in vitro* with an IC_50_ of 0.6 nM^24^ and inhibits RAF signaling in SKMEL28 cells with an IC_50_ of 3 nM.^25^ Profiling against a panel of 270 protein kinases revealed dabrafenib to be highly selective with limited (and apparently manageable) cross-reactivity against other kinases *in vitro*, including Alk5, LIMK1, NEK11, SIK, CK1, PKD2, and BRK protein kinases *in vitro* (IC_50_ values = 11 nM, 15 nM, 20 nM, 27 nM, 41 nM, 57 nM, and 79 nM, respectively).^24^ In addition, dabrafenib has been shown to have limited exposure in the brain as a result of rapid clearance by the efflux pumps P-glycoprotein and breast cancer resistance protein.^31^ We reasoned that dabrafenib’s newly uncovered inhibitory activity against PKR, its manageable target selectivity in cells, the absence of obvious pharmacological liabilities, and good safety profile as a drug would make it an excellent starting scaffold for the development of a PKR-selective tool inhibitor.

### Structure Analysis of PKR Bound to Dabrafenib

To understand the structural basis for the cross-reactivity of dabrafenib against PKR, we solved the structure of PKR bound to dabrafenib using X-ray crystallography (see Table 2 for X-ray data collection and refinement statistics). The structure revealed that dabrafenib binds to the active site of PKR in a manner highly similar to how dabrafenib binds to BRAF (Figures 2a and S3). Interestingly, when bound to dabrafenib, the kinase domain of PKR adopts a back-to-back dimer configuration reflective of an active state with a small lateral displacement of helix αC relative to the ATP bound state (Figure S4). Similar to the structure of BRAF bound to dabrafenib (PDB 4XV2), the kinase domain of PKR adopts an inactive-like conformation with helix αC in an outward position and the DFG motif in an inward position, commonly referred to as a type $1\frac{1}{2}$ binding mode.^32^ Features of the dabrafenib binding mode conserved across PKR and BRAF include (i) the pyrimidine group hydrogen bonds with the backbone of a cysteine residue in the kinase hinge, (ii) the *tert*-butyl group and thiazole core pack against the glycine-rich loop, (iii) the sulfonamide group makes two hydrogen bonds with the backbone amide groups of aspartic acid and phenylalanine of the inward positioned DFG motif (with the sulfonamide amine deprotonated), (iv) the aromatic ring of the aryl sulfonamide group sits on top of the phenyl group of the DFG motif, and (v) the aryl sulfonamide group occupies an allosteric pocket created by the outward position of the αC helix.

**Table 2:** X-ray Data and Crystallographic Refinement Statistics for the Crystal Structure of PKR Bound to Dabrafenib

	PKR_dabrafenib
resolution range	68.54–2.5 (2.56–2.5)
space group	*P*31
unit cell	95.33, 95.33, 122.97, 90, 90, 120
total reflections	227636 (15138)
unique reflections	43270 (2856)
multiplicity	5.3 (5.3)
completeness (%)	99.76 (99.54)
mean I/sigma (I)	19.67 (0.69)
wilson *B*-factor	90.49
*R*-merge	0.03351 (1.901)
*R*-meas	0.03725 (2.111)
*R*-pim	0.01614 (0.9089)
CC_1/2_	1 (0.348)
CC*	1 (0.719)
reflections used in refinement	43111 (2823)
reflections used for *R*-free	2153 (138)
*R*-work	0.2553 (0.4602)
*R*-free	0.2800 (0.5138)
CC (work)	
CC (free)	
number of non-hydrogen atoms	7292
macromolecules	7152
ligands	140
solvent	0
protein residues	992
nucleic acid bases	
RMS (bonds)	0.002
RMS (angles)	0.4
ramachandran favored (%)	98.75
ramachandran allowed (%)	1.25
ramachandran outliers (%)	0
rotamer outliers (%)	0
clashscore	3.42
average *B*-factor	111.24
macromolecules *B*-factor	111.71
ligands *B*-factor	87.23
solvent *B*-factor	
number of TLS groups	

**Figure 2:**
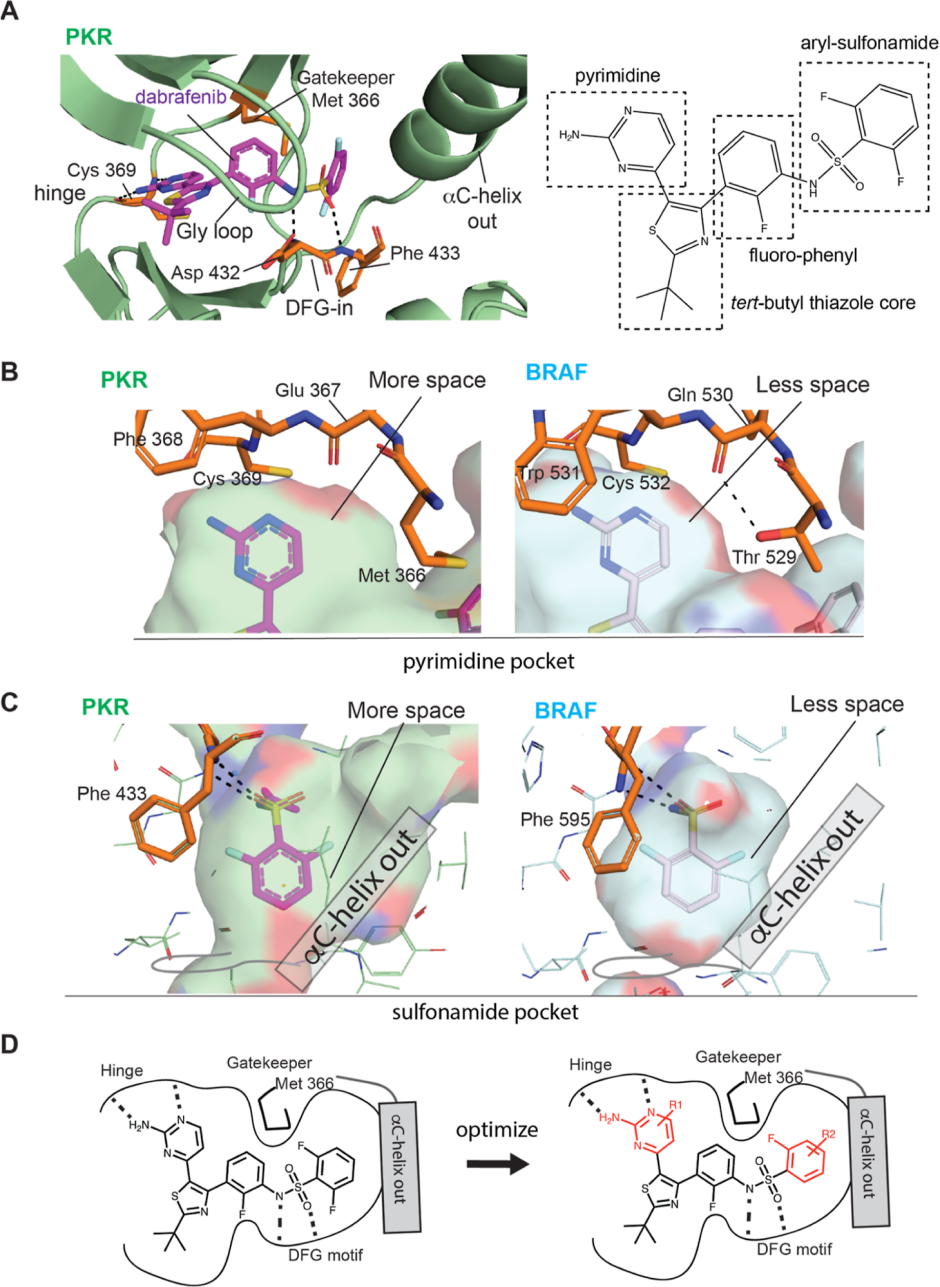
Crystal structure of dabrafenib bound to PKR. (A) Left: dabrafenib binds to PKR with an αC helix-out and DFG-in binding mode. Right: chemical structure of dabrafenib. (B) Solvent accessible protein surface around the pyrimidine binding pocket of PKR and BRAF (PDB 4XV2). (C) Solvent accessible protein surface around the sulfonamide binding pocket of PKR and BRAF (PDB 4XV2). (D) Schematic of inhibitor optimization. Hydrogen bonds are shown in dashed lines. Key residues and features are labeled.

Prominent structural differences between PKR and BRAF in the close vicinity to dabrafenib include (i) the residues around the pyrimidine group, which create additional unoccupied space in the pocket in PKR and (ii) a larger pocket at the base of helix αC occupied by the sulfonamide group in PKR (Figure 2B,C). We hypothesized that differences in these two pockets could be exploited to evolve inhibitor selectivity and potency toward PKR. Specifically, the larger pockets in PKR than in BRAF suggest that elaboration of the pyrimidine and the sulfonamide groups of dabrafenib could improve favorable contact with PKR and introduce unfavorable steric clash with BRAF (Figure 2D).

### Modifications to the Pyrimidine Group Abolish Activity against BRAF while Retaining Activity against PKR

We first focused on inhibitor optimization by modifying the pyrimidine group of dabrafenib. In PKR, the linear and aliphatic side chain of Met 366 packs loosely with the pyrimidine group, whereas in BRAF, the branched and polar side chain of Thr 529 packs tightly with the pyrimidine group (Figure 2B). In addition, in BRAF, the hydroxyl group of the Thr 529 side chain makes a hydrogen bond with the main-chain carbonyl of the adjacent Gln 530 residue. We predicted that these differences would limit the flexibility of the pocket in BRAF compared to PKR and thus that the addition of substituents to the pyrimidine group would be better accommodated by PKR than BRAF. To test this hypothesis, we introduced methyl, amino, ethyl, and methoxy groups to the C^6^ position of the pyrimidine moiety of dabrafenib (compounds **5a**, **5b**, **5c**, and **5d**, respectively, Table 3). Modeling using Boltz2 predicted that these modifications would be tolerated (e.g. 3-fold reduction in predicted affinity of **5a** compared to dabrafenib, Tables S1 and S2).

**Table 3:**
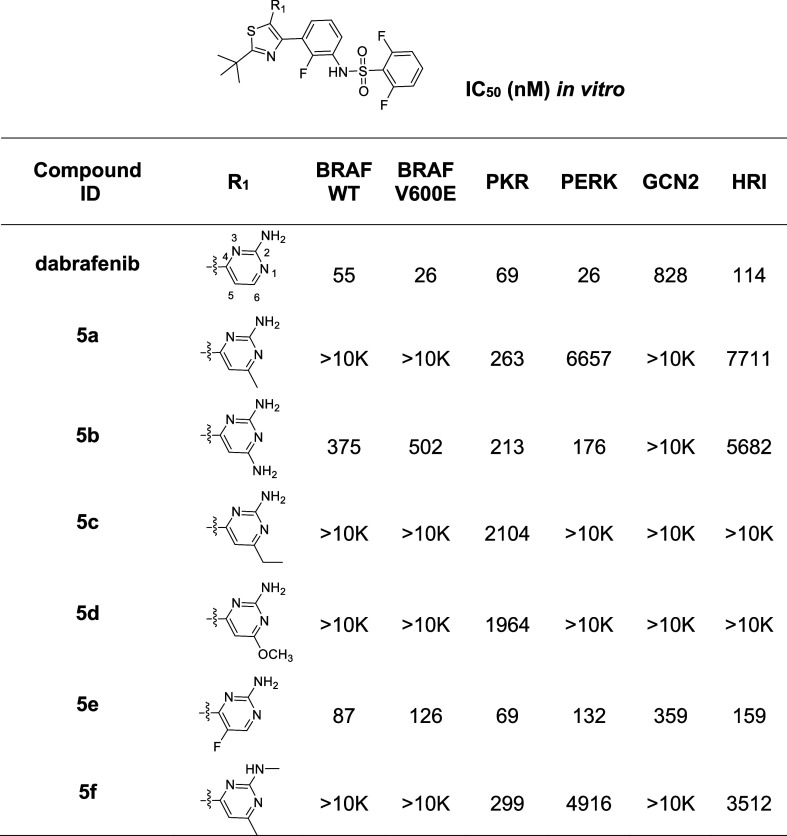
*In Vitro* Kinase Profiling of Dabrafenib Pyrimidine Analogues (Associated with Figure S5)

Compounds **5a**–**5d** were synthesized as outlined in Scheme 1. Synthesis began with the commercially available ester **1**, which was condensed with the lithium anion of 2-chloro-4-methylpyrimidine derivatives (**2a**–**d**) to obtain ketone intermediates **3a–d** in 60–76% yields. Bromination of the intermediates **3a–d** was performed using *N*-bromosuccinimide (NBS), followed by the cyclization reaction with 2,2-dimethylthiopropionamide to give compounds **4a–d** in 55–78% yields. Finally, an S_N_Ar displacement reaction using ammonia solution or methanolic ammonia at the chloropyrimidine moiety led to the desired products **5a–d** in 45–71% yields.

**Scheme 1:**
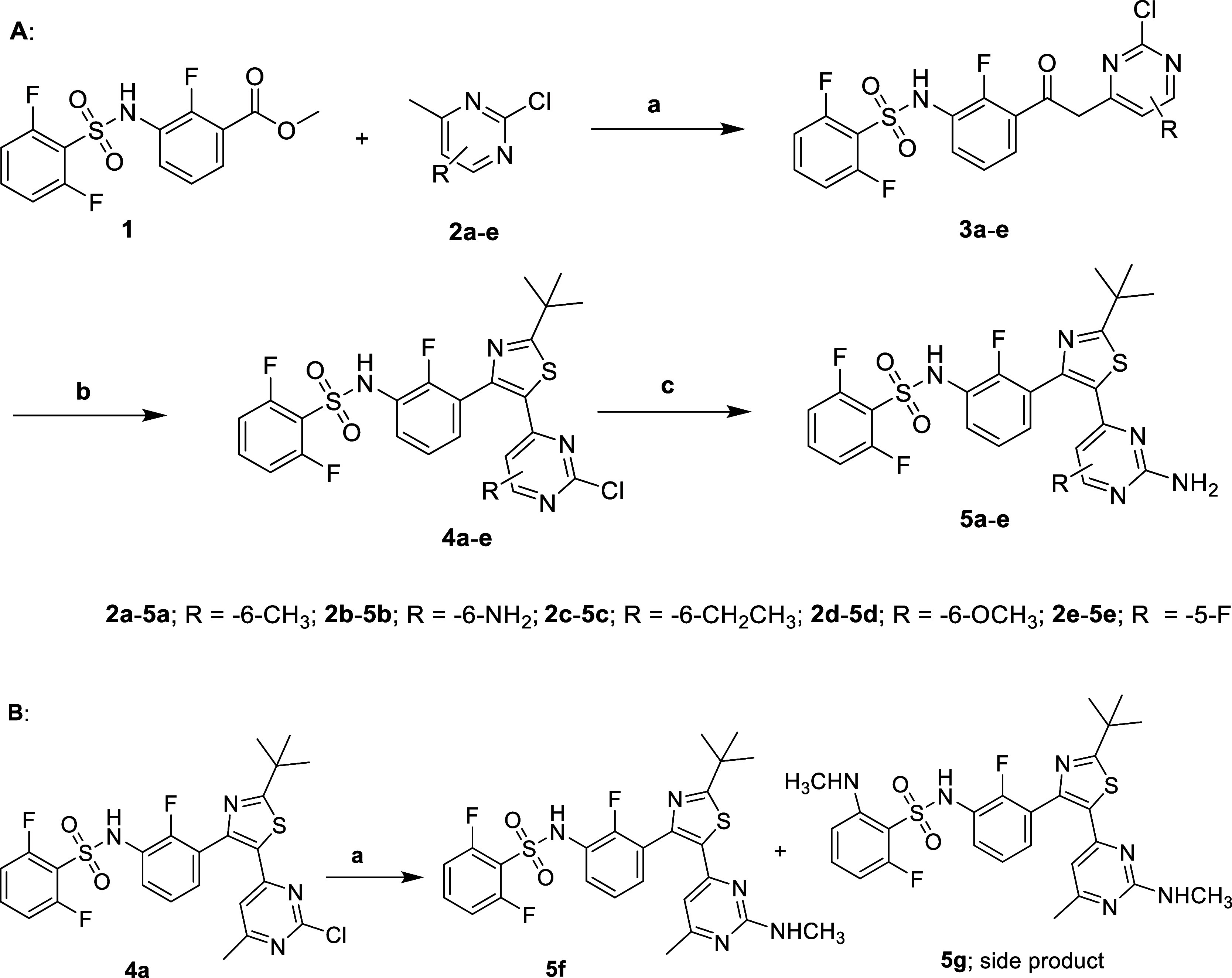
Synthesis of Dabrafenib Analogues **5a**–**f** ^a^,^b^

a Reagents and conditions: (a) LiHMDS, tetrahydrofuran (THF), 0 °C rt, 2–3 h, 60–76%; (b) NBS, 2,2-dimethylthiopropionamide, DMA, 80 °C, 1–2 h, 55–78%; (c) ammonia solution, 7N in MeOH/NH_4_OH solution, MW, 120 °C, 1 h, 45–71%.
b Reagents and conditions: (a) CH_3_NH_2_, 2.0 M in THF, 60 °C, 16 h, 40%.

*In vitro* kinase activity analysis carried out at Eurofins revealed that the methyl substitution in compound **5a** caused almost a complete loss of activity against BRAF WT (IC_50_ > 10,000 nM) while only a modest 3.8-fold loss of activity against PKR (IC_50_ of 263 nM) (Table 3, see also Figure S5 for profiles). The amino substitution in compound **5b** caused a comparable 3-fold loss of activity against PKR (IC_50_ of 213 nM) but a less severe (relative to **5a**) 6.8-fold loss of activity against BRAF WT (IC_50_ = 375 nM). Lastly, the ethyl and methoxy substitutions (compound **5c/5d**) caused large ∼30-fold losses of activity against PKR (IC_50_ 2104 and 1964 nM) and more drastic losses of activity against BRAF WT (IC_50_ > 10,000 nM).

Next, we explored substitutions on the adjacent C^5^ position of the pyrimidine group with a small fluorine substituent (compound **5e**). Compound **5e** was synthesized as outlined in Scheme 1A, as described above. A small fluoro-substitution of the C^5^ position of pyrimidine (compound **5e**) resulted in no change in activity against PKR relative to dabrafenib (IC_50_ = 69 nM) and a minor 1.6-fold loss of activity against BRAF WT (IC_50_ = 87 nM) (Table 3). Finally, we explored growing into the kinase hinge region by elaborating substituents on the amine at the C^2^ position of the pyrimidine group, combined with a methyl at the C^6^ position (compound **5f**). Compound **5f** was synthesized as outlined in Scheme 1B, beginning with compound **4a**, which was treated with methylamine to give **5f** in 40% yield, along with **5g** as a side product. The methylamine substitution (compound **5f**) was well tolerated with similar potency against PKR as compound **5a** (IC_50_ = 299 nM) and displayed no apparent activity against BRAF (Table 3). Together, these results demonstrated that the pocket in PKR occupied by the pyrimidine group is accommodating of changes to the pyrimidine group and that a methyl group addition to position C^6^ gave rise to the greatest enhancement of specificity while minimizing the loss of activity against PKR.

### Elaboration of the Sulfonamide Group Can Improve Potency and Selectivity toward PKR over BRAF

We next explored varying the sulfonamide moiety of dabrafenib, which occupies a pocket formed by the outward movement of the αC helix. As this pocket appears larger in PKR than in BRAF, we hypothesized that the sulfonamide group may be a viable vector to enhance favorable interactions with PKR while generating unfavorable steric clashes with BRAF (Figure 2C). We note that a similar design strategy was successfully applied for the development of a GCN2 selective inhibitor GCN2iB,^33^ which is highly similar to PKR in the sulfonamide binding pocket. To explore if changes to substituents on the sulfonamide phenyl ring could improve potency and/or selectivity, we modeled the binding of a small library of 322 analogues with systematic 1 or 2 substitutions of H, F, Cl, Br, trifluoromethyl, methyl, ethyl, and methoxy groups in the ortho, meta, and para positions of the phenyl ring (while fixing the ortho fluorine that is expected to participate in deprotonating the sulfonamide amine) to PKR using Boltz2 ^34^ (Figure 3, see also Table S3 for Boltz2 screen output). We then selected seven analogues based on a combination of visual inspection of modeled poses, availability of starting reagents for synthesis and Boltz2 scores (compounds **9a**–**9g**, Table 4).

**Figure 3:**
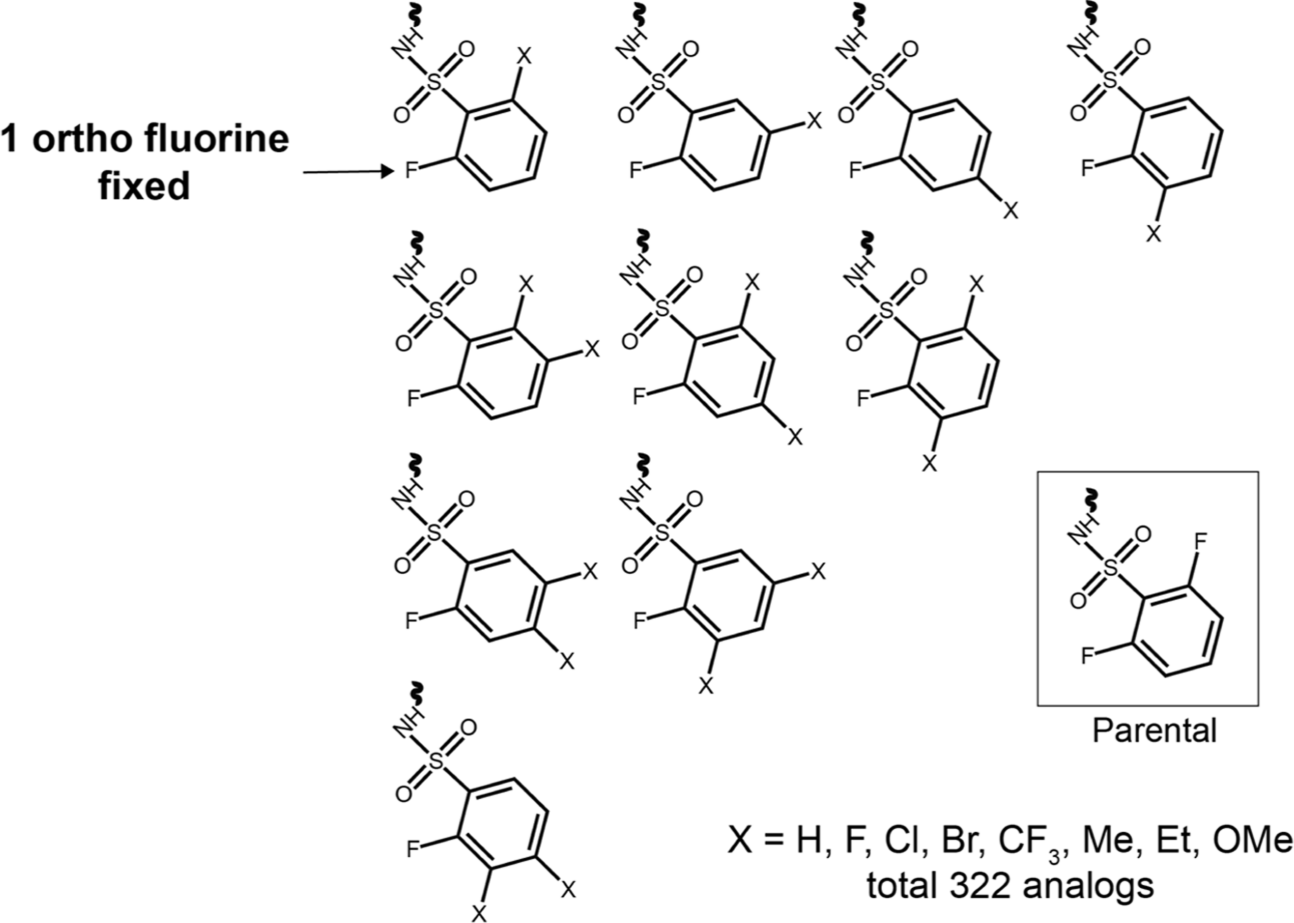
Summary of sulfonamide library used for Boltz2 analysis. Chemical structures of the sulfonamide headgroups are shown with modifications as indicated.

**Table 4:**
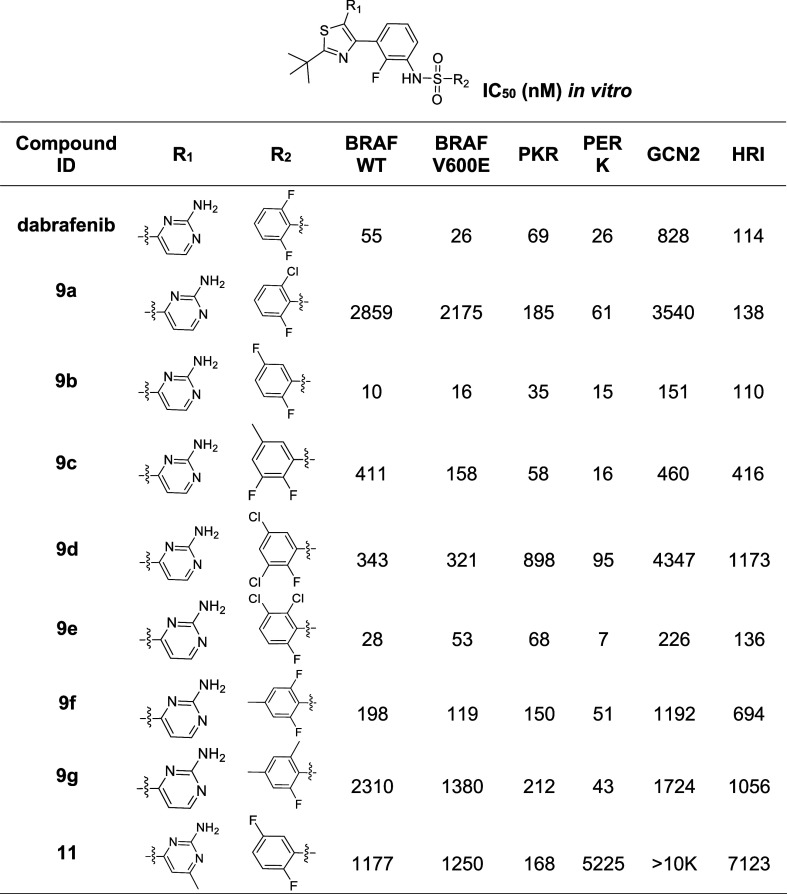
*In Vitro* Kinase Profiling of Dabrafenib Sulfonamide and Combination Analogues (Associated with Figure S5)

We employed a synthetic route that allowed us to efficiently exchange the aryl sulfonamide headgroup of dabrafenib in a 2-step reaction to generate the desired analogue (Scheme 2),^35^ thereby bypassing the 4–5 step reaction required to start a synthesis from scratch.^25,36^ The first step involved three consecutive one-pot reactions where the secondary sulfonamide reacts rapidly and chemo selectively with ethyl benzoylformate and tris­(dimethylamino)­phosphine to form an intermediate *N*-sulfonyl phenylglycine ester. Next, *tert*-butyl tetramethylguanidine (BTMG, Barton’s base) was used for fragmentation reactions to generate the sulfinate and imine product. The imine intermediate when treated with aqueous hydroxylamine led to the free amine intermediate **7** in 60% yield, which was further reacted with substituted phenyl sulfonyl chlorides (**8a**–**g**) to give the desired products **9a**–**g** in 40–96% yield.

**Scheme 2:**
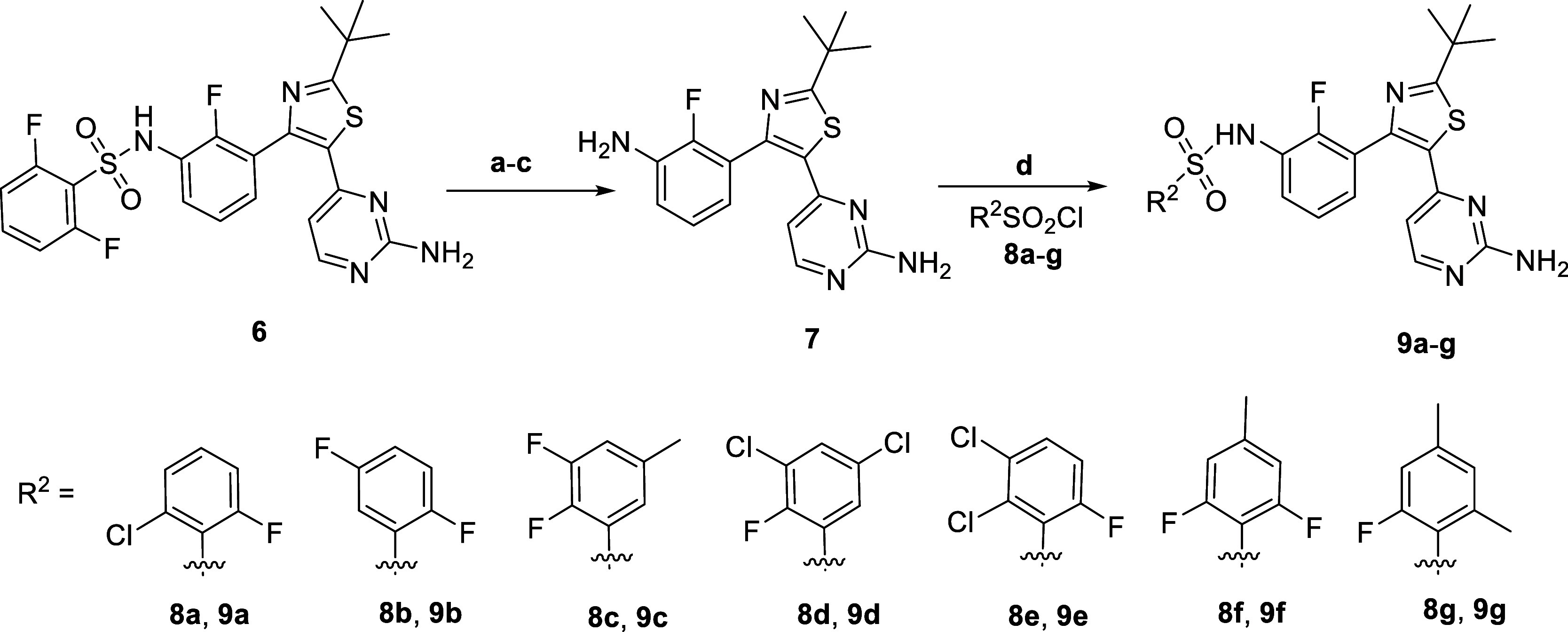
Synthesis of Dabrafenib Analogues **9a**–**g** ^a^

a Reagents and conditions: (a) ethylbenzoylformate, tris­(dimethylamino)­phosphine, rt, 1 h; (b) 2-*tert*-butyl-1,1,3,3-tetramethylguanidine (BTMG), 65 °C, 4 −5 h; (c) hydroxylamine, rt, 1 h, 60%; (d) pyridine, DCM, rt, 12–15 h, 40–96%.

Notably, *in vitro* kinase activity analysis revealed that substituting one of the ortho-fluorines with chlorine in compound **9a** dramatically reduced potency against BRAF by 52-fold (IC_50_ = 2859 nM) while only slightly reducing potency against PKR by 2.7-fold (IC_50_ = 185 nM), thereby improving overall selectivity for PKR (Table 4, see also Figure S5 for inhibitor profiles). Moving one of the ortho fluorine substituents to the meta position in compound **9b** improved potency against PKR by 2-fold (IC_50_ = 35 nM). However, this change also enhanced potency against BRAF by 5.5-fold (IC_50_ = 10 nM), therefore reducing overall selectivity for PKR. Encouragingly, moving one of the ortho-fluorine to the meta position and introducing a methyl group in the second meta position in compound **9C** reduced potency against BRAF by 7.5-fold (IC_50_ = 411 nM) while largely leaving intact the potency against PKR (IC_50_ = 58 nM). Together, these results suggest that changes to the sulfonamide group are a viable path to improving both potency and selectivity toward PKR.

Having shown that introducing a methyl substitution at the C^6^ position of the pyrimidine of dabrafenib improves selectivity for PKR over BRAF by >180-fold at a small 3.8-fold loss of overall potency against PKR (compare dabrafenib vs compound **5a**) and that moving one of the ortho fluorines to the meta position of the sulfonamide group can improve potency against PKR by 2-fold (compare dabrafenib to compound **9b**), we asked whether combining the two features in compound **11** could maintain both the high selectivity of **5a** and the improved potency of **9b**.

Compound **11** was synthesized, as outlined in Scheme 3. The first step involved the conversion of the analogue **5a** to an amine intermediate (**10**) following synthesis steps similar to those in Scheme 2. The amine intermediate **10** was further reacted with phenyl sulfonyl chloride **8b** to give the desired product 4-{2-*tert*-butyl-4-{3-(2,5-difluorophenylsulfonylamino)-2-fluorophenyl}-1,3-thiazol-5-yl}-6-methyl-2-pyrimidinamine (**11**) in 70% yield.

**Scheme 3:**
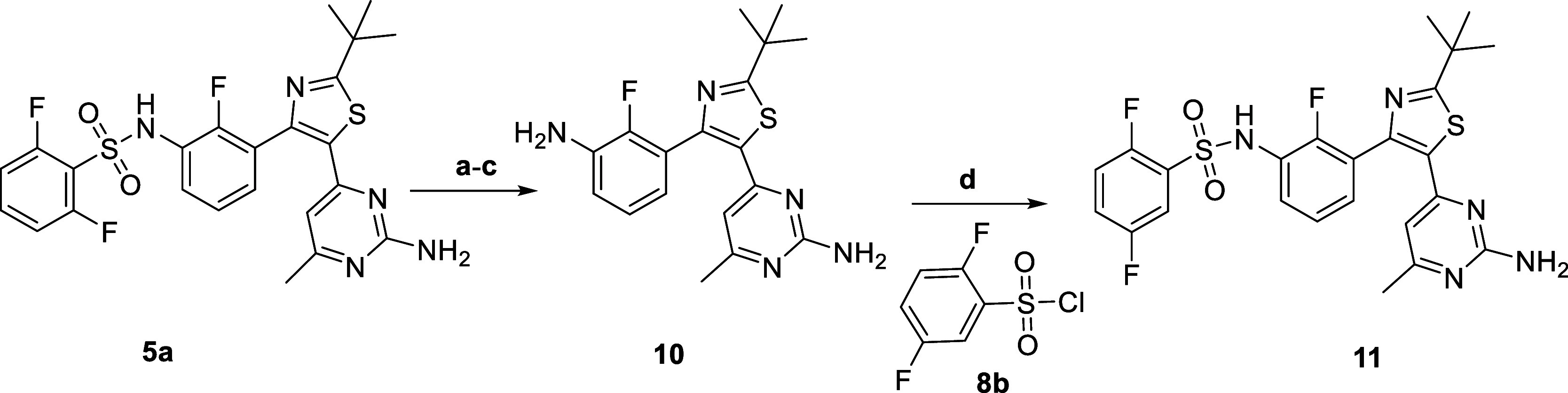
Synthesis of Dabrafenib Analogue 11^a^

a Reagents and conditions: (a) ethylbenzoylformate, tris­(dimethylamino)­phosphine, rt, 1 h; (b) 2-*tert*-butyl-1,1,3,3-tetramethylguanidine (BTMG), 65 °C, 4–5 h; (c) hydroxylamine, rt, 1 h, 65%; (d) pyridine, DCM, rt, 12 h, 70%.

Contrary to expectation, compound **11** displayed reduced potency against PKR by 2.4-fold relative to dabrafenib (IC_50_ 168 nM vs 69 nM), while imparting only 7-fold selectivity for PKR over BRAF, which is better than the selectivity of dabrafenib (1.25-fold selectivity against PKR) but inferior to the selectivity displayed by compound **5a** (180-fold selectivity for PKR over BRAF) (Table 4). Nonetheless, together, these results identify the pyrimidine and sulfonamide groups of dabrafenib as productive vectors to enhance inhibitor selectivity and potency by exploiting differences in their respective binding pockets in PKR and BRAF.

### Profiling of Dabrafenib Analogues against BRAF^V600E^ and Other eIF2α Kinases

As dabrafenib was originally optimized to target the BRAF^V600E^ mutant, we also investigated the activity of the dabrafenib analogues against BRAF^V600E^. Overall, the inhibitory activity of the analogues mirrored their activity against BRAF WT (Tables 3 and 4). This was not surprising as the site of the V600E mutation is remote from the inhibitor binding site. We also profiled the activity of our analogues against PERK, GCN2, and HRI since the eIF2α kinases are highly similar in their kinase domains. Dabrafenib itself showed strong cross-reactivity against PERK (IC_50_ = 26 nM) and HRI (IC_50_ = 114 nM) but far less cross-reactivity to GCN2 (IC_50_ of 828 nM) *in vitro* (Table 3). The activity against PERK was surprising, as this was not reported previously and would be predicted to be toxic to insulin-producing cells.^37^ Importantly, elaboration of the C^6^ position of the pyrimidine group of dabrafenib appeared effective at dialing out activity against PERK, GCN2, and HRI. For example, relative to dabrafenib, compound **5a** showed 256-fold loss of activity against PERK (IC_50_ 6657 nM vs 26 nM), 12-fold loss against GCN2 (IC_50_ > 10,000 nM vs 828 nM), and 67-fold loss against HRI (IC_50_ 7711 nM vs 114 nM) while modestly reducing activity against PKR by only 3.8-fold (Table 3).

Elaboration of the sulfonamide group also effectively dialed out activity against BRAF while maintaining potency against PKR, but this was less effective in dialing out activity against the other eIF2α kinases (Table 4). For example, relative to dabrafenib, compound **9c** displayed reduced potency against BRAF WT by 7.5-fold (IC_50_ = 411 nM vs 55 nM) while marginally improving potency against PKR by 1.2-fold (IC_50_ = 58 nM vs 69 nM). However, **9c** also displayed improved potency against PERK by 1.6-fold (IC_50_ = 16 nM vs 26 nM) and GCN2 by 1.8-fold (IC_50_ = 460 nM vs 828 nM). Together, these results show that the pyrimidine group of dabrafenib and, to a lesser extent, the sulfonamide group are viable sites for modification to enhance activity against PKR while reducing activity against BRAF and other eIF2α kinases. Consistent with our dabrafenib analogue series maintaining a common binding mode to PKR, both dabrafenib and **5a** competitively displaced a kinase tracer from the active site of PKR (Figure S7).

### Functional Testing of Dabrafenib and Compound **5a** in Cells

Having shown that we can dial out activity against BRAF while maintaining considerable potency against PKR *in vitro*, we next investigated if inhibitor properties were maintained in a cellular context. Following treatment of HEK293T cells with poly­(I:C) to activate PKR autophosphorylation activity as detected by PKR phosphor Thr 446 immunoblot, dabrafenib inhibited PKR autophosphorylation with an IC_50_ of ∼100 nM (Figure 4A) while compound **5a** inhibited PKR with 10-fold reduced potency of IC_50_ of ∼1 μM. These results paralleled those observed *in vitro* (Table 3). Consistent with this, compound **5a** reduced both PKR autophosphorylation and downstream eIF2α phosphorylation in wild-type cells but had no effect in ΔPKR HEK293T cells (Figure 4B), supporting the notion that compound **5a** acts on-target to inhibit the cellular response to exposure to poly­(I:C).

**Figure 4:**
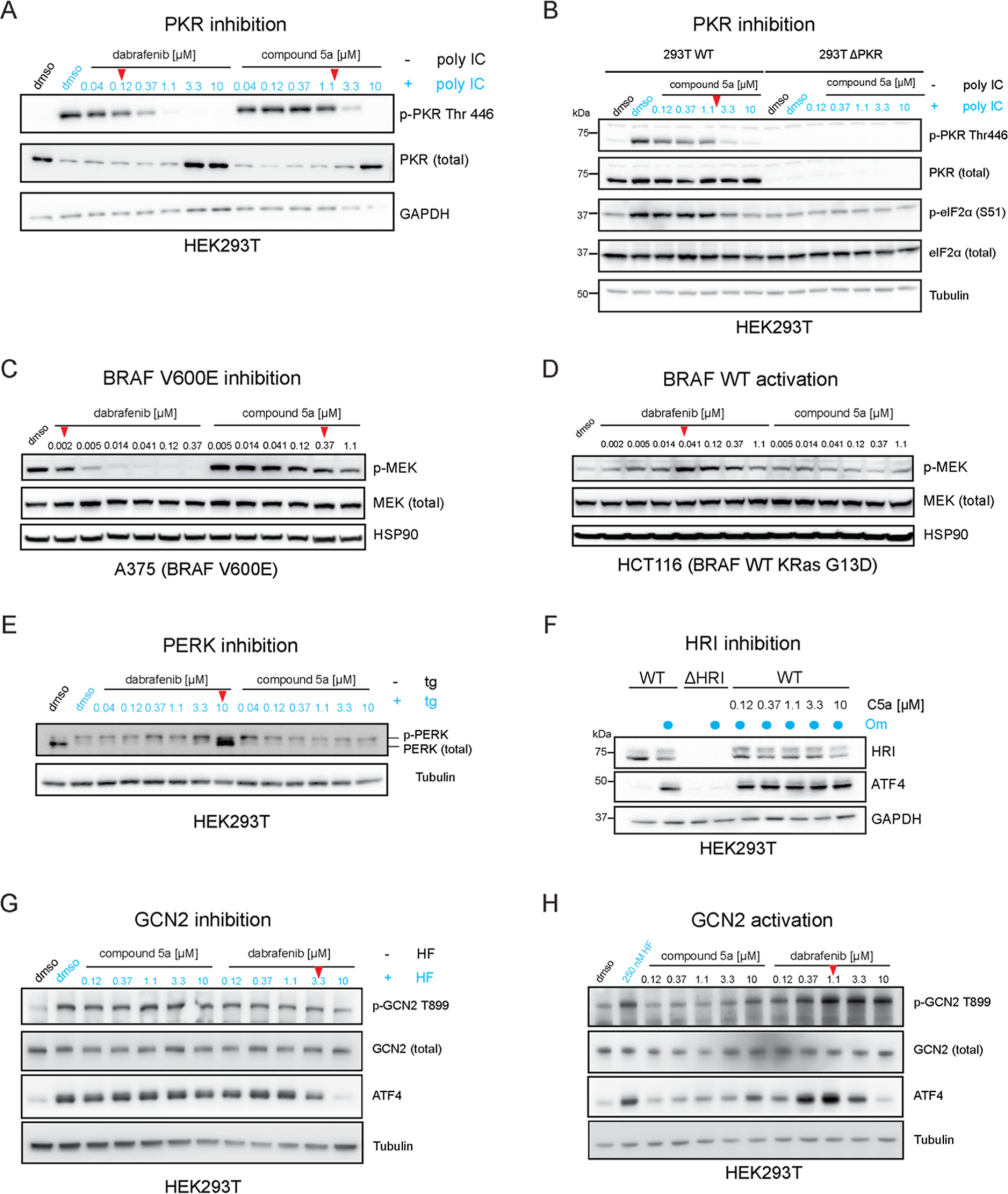
Functional testing of dabrafenib and **5a** inhibitors in cells. (A) Profiling activity of dabrafenib and compound **5a** against PKR in HEK293T cells by Western blot analysis using PKR phosphor-Thr 446 as a readout following treatment with poly­(I:C). (B) Profiling activity of compound **5a** against PKR and ΔPKR HEK293T cells by Western blot analysis using PKR phosphor-Thr 446 and *p*-eIF2α (Ser 51) as readouts following treatment with poly­(I:C). (C) Profiling activity of dabrafenib and compound **5a** for effect on RAF signaling in A375 cells (BRAF V600E) by Western blot analysis monitored by MEK phosphorylation status. (D) Profiling activity of dabrafenib and compound **5a** for effect on RAF signaling in HCT116 cells (BRAF WT and KRASG13D) by Western blot analysis monitored by MEK phosphorylation status. (E) Profiling activity of dabrafenib and compound **5a** against PERK in HEK293T cells by Western blot analysis of PERK auto phosphorylation status following treatment with thapsigargin (tg). (F) Profiling activity of compound **5a** against HRI in WT or ΔHRI HEK293T cells by Western blot of ATF4 levels after treatment with oligomycin (Om). (G) Profiling activity of dabrafenib and compound **5a** by Western blot of ATF4 levels for effect on GCN2 inhibition in HEK293T cells treated with halofuginone (HF). (H) Profiling activity of dabrafenib and compound **5a** for effect on GCN2 paradoxical activation in HEK293T cells monitored by Western blot analysis of ATF4 levels (all data shown are representatives of *n* ≥ 2 independent experiments).

Next, we examined the activity of dabrafenib and compound **5a** against BRAF. As the oncogenic BRAF V600E mutant is the primary target of dabrafenib, we first examined the activity of dabrafenib in the A375 melanoma cell line that harbors homozygous V600E mutation of BRAF. Dabrafenib inhibited RAF signaling as read out by pMEK levels with an IC_50_ of ∼1.5 nM (Figure 4C), while compound **5a** displayed more than 100-fold weaker inhibitory activity against RAF with an IC_50_ of >150 nM. These results also parallel our *in vitro* observations (Table 3). We then profiled dabrafenib and compound **5a** against BRAF WT in HCT116 cell line that harbors homozygous wild-type BRAF and the heterozygous KRAS WT/G13D activating mutation. As reported by others, rather than mediating the inhibition of pathway signaling, dabrafenib caused paradoxical activation of RAF, with peak activation at ∼40 nM of dabrafenib (Figure 4D). In contrast, compound **5a** showed no paradoxical activation of RAF even at concentrations up to 1.1 μM. Together, these results in cells are consistent with the reduced potency of compound **5a** relative to dabrafenib observed against both BRAF WT and BRAF V600E *in vitro* (Table 3).

Since dabrafenib potently inhibited PERK *in vitro*, with potency greater than against PKR (Table 1), we next profiled dabrafenib inhibitory activity against PERK in cells. Surprisingly, dabrafenib displayed no activity against PERK in HEK293T cells until the highest concentration of 10 μM tested, as measured by its inability to inhibit PERK autophosphorylation after treatment of cells with the PERK activator thapsigargin (Figure 4E). This observation might explain why pancreatic toxicity has not been described in cancer patients taking dabrafenib. In contrast, compound **5a**, which displays 256-fold weaker potency against PERK than dabrafenib *in vitro* (IC_50_ = 6657 nM) (Table 3) displayed no ability to inhibit PERK in cells even at 10 μM concentration.

We next tested compound **5a** for inhibition of HRI and GCN2. In HEK293T cells treated with oligomycin to induce HRI activation, compound **5a** showed no detectable inhibition of HRI, even at 10 μM (Figure 4F). Similarly, in cells treated with halofuginone to activate GCN2, compound **5a** did not inhibit GCN2 activation, whereas dabrafenib inhibited GCN2 with an IC_50_ ∼3–4 μM (Figure 4G). Both these results are consistent with our *in vitro* analysis (Table 3).

Finally, dabrafenib was reported to induce the expression of ATF4 through the paradoxical activation of GCN2. As paradoxical activation of GCN2 would negate the utility of inhibiting PKR, we assessed whether this behavior also held true for compound **5a**. While we could detect the induction of ATF4 by dabrafenib with maximum induction at 1 μM in HEK293T cells (Figure 4H), we observed only slight induction of ATF4 by compound **5a** at 10 μM. These results are consistent with our finding that compound **5a** is less potent at engaging GCN2 than dabrafenib *in vitro* (IC_50_ > 10,000 nM for compound **5a** vs 828 nM for dabrafenib) (Table 3).

Overall, the activity of dabrafenib and compound **5a** in cells parallels their activity *in vitro*, with the exception of their activity against PERK; while dabrafenib is potent against PERK *in vitro,* it showed very weak inhibitory activity against PERK in cells. The behavior of compound **5a** demonstrates that we can maintain considerable activity against PKR while dialing out activity against BRAF *in vitro* and in a cellular context. Furthermore, relative to dabrafenib, compound **5a** displays greater selectivity for PKR over the other eIF2α kinases PERK, HRI, and GCN2 both *in vitro* and in cells, which would reduce liabilities associated with the positive or negative modulation of these kinases.

### Broader Profiling of **5a** against 58 Representative Protein Kinases

To assess its selectivity across the kinome, we tested **5a** alongside dabrafenib at a single concentration of 1 μM in the Eurofins Diversity Kinase Panel, which consists of 58 diverse kinases, at each kinases’ respective [Km] ATP. Gratifyingly, in contrast to dabrafenib, which was found to inhibit seven out of 58 kinases at >50%, compound **5a** was found to inhibit only one kinase at >50% (CaMKI, 52% inhibition) at 1 μM, with little inhibition seen across the majority of other kinases tested in this panel (Table S4). This data suggests that, in addition to enhancing selectivity over the primary original target of the scaffold (B-Raf), the structural modifications incorporated into compound **5a** also increased its overall selectivity across the kinome.

### Rapid Pharmacokinetic Analysis

To investigate the suitability of **5a** for *in vivo* studies, we carried out a rapid PK analysis. Following a 10 mg/kg oral dose in male NOD-SCID mice, **5a** achieved adequate systemic exposure, with plasma concentrations of approximately 1000 nM sustained at both 2 and 4 h postdose (Table 5). In contrast, brain exposure was limited, with concentrations substantially lower than those observed in plasma. This resulted in high plasma-to-brain ratios of 72.8 and 64.7 at the respective time points, indicating poor blood–brain barrier (BBB) penetration, a characteristic shared with the parental molecule dabrafenib.^31^

**Table 5:** Pharmacokinetic Analysis of Compound **5a** ^a^

sample	concentration (nM)	standard deviation	plasma/brain
plasma 2 h	1887	125	72.8
brain 2 h	25.9	1.6	
plasma 4 h	776	395	64.7
brain 4 h	12.0	3.7	

a Plasma and brain concentrations of compound **5a** (OICR-040318) at 2 and 4 h following a single 10 mg/kg oral dose in male NOD-SCID mice, highlighting limited brain exposure and high plasma-to-brain ratios. Three biological replicates were analyzed per time point.

## Discussion and Conclusions

PKR is a compelling therapeutic target for small molecule inhibitors, given its dual role in promoting neurodegeneration and inflammation, pathways that are implicated in Alzheimer’s disease and IBD. We discovered that dabrafenib, an FDA-approved drug designed to target the oncogenic BRAF^V600E^ mutant, is also a potent inhibitor of PKR. Employing crystal structure and computational modeling, we leveraged this cross-reactivity to rationally design compound **5a (OICR-403184**), which retains moderate potency against PKR while enhancing selectivity for PKR over BRAF WT and other eIF2α kinases both *in vitro* and in cellular contexts. We note that while Boltz2 proved useful in our hit-finding exercise, it proved less effective in guiding SAR optimization.

Compound **5a (OICR-403184**) provides a chemically tractable starting point for the development of more potent and selective PKR inhibitors. Future optimization of the pyrimidine and sulfonamide groups of compound **5a (OICR-403184**) is expected to lead to further improvement in potency and selectivity toward PKR. In addition, we showed that elaboration of the pyrimidine group into the kinase hinge is well-tolerated, suggesting it is an additional vector for optimization. Finally, we found PIK-75, gilteritinib, and A-443654 to display potent inhibitory activity against PKR, thereby providing additional chemical series for the development of future generations of PKR inhibitors. These inhibitors will allow more rigorous testing of therapeutic hypotheses for targeting PKR in antiviral, neurodegenerative, and IBD contexts *in vivo*.

PKR activation proceeds through a sequential mechanism in which dsRNA first engages the tandem dsRNA-binding domains, promoting back-to-back dimerization of the kinase domain, *trans*-autophosphorylation of Thr446 within the kinase activation segment, and subsequent binding and phosphorylation of eIF2α.^4,5^ Our data indicate that compound **5a** acts at an early step in this pathway by preventing PKR autophosphorylation through competition with ATP for binding at the kinase active site (Figure S7). This interpretation is consistent with the binding mode observed for the closely related parental inhibitor dabrafenib in the PKR cocrystal structure (Figure 2). We further infer that compound **5a** is unlikely to disrupt back-to-back dimerization of the kinase domain, as PKR adopts the expected back-to-back dimer configuration when bound to dabrafenib (Figure S4). Likewise, compound **5a** is unlikely to directly interfere with eIF2α binding, as the eIF2α interaction surface is centered on helix αG, which lies 21 Å from the dabrafenib-binding site (Figure S4).

In addition to its catalytic role in transferring phosphate from ATP to itself and to eIF2α, PKR has also been proposed to function in a catalytic-independent manner in several signaling contexts, including NF-κB signaling,^38,39^ inflammasome signaling,^40,41^ and mammarenavirus infection.^42^ Because compound **5a** appears to act primarily by competing with ATP at the kinase active site, it is less likely to perturb these kinase-independent functions, although this cannot be concluded with certainty at present. More generally, because several prior studies relied on less selective small-molecule probes, including C16 ^42^ and 7DG,^40^ compound **5a** may provide a cleaner pharmacological tool for re-examining the extent to which earlier mechanistic conclusions regarding noncatalytic PKR functions are probe-dependent.

## Experimental Section

### Virtual Screen for PKR Inhibitors Using Boltz2 against the OICR Kinase Inhibitor Library

Boltz2 ^34^ was used to predict the binding mode, binding affinity (affinity pred value), and likelihood of binding (predicted affinity probability binary) for 647 molecules in the OICR kinase inhibitor library against the kinase domain of PKR (residues 250–543) using 10 recycling steps and inference time potentials. The molecules were then sorted by predicted binding affinity and likelihood of binding to aid in the selection of molecules for further validation. See Supporting Information for Boltz2 input parameter and output details.

### Boltz2 Computational Modeling of Sulfonamide Analogues

A library of 322 dabrafenib analogues in SMILES format was generated by ChatGPT with the sulfonamide amine deprotonated and the following structural changes: only the aromatic group of the sulfonamide moiety was varied by fixing the ortho fluorine and systematically introducing 1 or 2 substitutions of H, F, Cl, Br, CF_3_, methyl, ethyl, and methoxy groups in the remaining 2, 3, 4, and 5 positions. Boltz2 was then used to predict the binding affinity and binding mode of each analogue to the kinase domain of PKR using 10 recycling steps and inference time potentials. See Supporting Information for Bolz2 input parameter and output details.

### PKR Protein Expression

The kinase catalytic domain of PKR was expressed and purified as previously described.^4^ Briefly, PKR (residues 258–551) with an N-terminal TEV cleavable GST tag was expressed in *E. coli* BL21 cells. The expressed protein was captured on glutathione sepharose resin and eluted by cleavage with the TEV protease. The eluted protein was then captured on a Q-sepharose column and eluted with NaCl gradient and then further purified by gel filtration chromatography in 10 mM HEPES, pH 7, 150 mM NaCl, and 5 mM DTT. The final protein was concentrated to 10 mg/mL before flash-freeze in liquid nitrogen for long-term storage.

### Protein Crystallization and Structure Determination

PKR protein at 10 mg/mL was incubated with dabrafenib at 1 mM at a final dimethyl sulfoxide concentration of 2% and incubated for 1 h on ice. Hanging drops were set up with 2 μL of protein and 1 μL of mother liquor containing 15% PEG 3350, 0.2 M sodium fluoride, and 0.1 M HEPES pH 7.5. Crystals were cryoprotected with mother liquor containing 20% glycerol prior to flash freezing in liquid nitrogen. Diffraction data were collected at the advanced photon source (APS) at a wavelength of 0.979180 Å. Data reduction was performed using XDS.^43^ Structure was solved by molecular replacement in Phaser^44^ using the structure of PKR kinase domain from PDB 2A19 as a search model. Model building and refinement were carried out in Coot^45^ and Phenix.^46^ Constraints for dabrafenib were generated using eLBOW.^47^ X-ray data and crystallographic refinement statistics are shown in Table 2.

### *In Vitro* Kinase Inhibition Assay

*In vitro* inhibitory activity of compounds against PKR (no. 4–955 KP), PERK (no. 14–916 KP), GCN2 (no. 14–934 KP), HRI (no. 16–013 KP), BRAF V600E (no. 14–557 KP), and BRAF WT (no. 14–530 KP) were carried out at Eurofins using Kinase Profiler.

### Kinase Tracer Competition Assay

Purified PKR protein at 750 nM was incubated with 20 nM of kinase tracer 236 (Thermo Scientific, Cat# PV5592) and serial dilutions of compound **5a** or dabrafenib in a buffer consisting of 10 mM MOPS pH 7, 0.2 mM EDTA, and 10 mM MgAcetate in a final assay volume of 20 μL/well in triplicate. Assays were performed in 384-well plates (Corning, Cat # 3573) with a final DMSO concentration of 2%. Tracer binding was quantified by fluorescent polarization after a 40 min incubation at room temperature using a Synergy NEO microplate reader. Dose-response curves were plotted using GraphPad PRISM, and average IC_50_ values of three independent experiments were reported.

### ΔPKR and ΔHRI HEK293T Cell Line Generation

ΔPKR and ΔHRI HEK293T cells were generated with CRISPR Cas12a editing using sgRNAs with protospacer sequence targeting PKR (5′-ATCCAAAGGCAATACGTACC-3′) or HRI (5′-GGGCTTACCTGACGAACTCTC-3′). sgRNAs were *in vitro* transcribed using the HiScribe T7 RNA Synthesis Kit (New England Biolabs, E2050). Following DNA template digestion with DNase I (NEB, M0303), sgRNAs were purified using NucleoMag NGS cleanup beads (Machery-Nagel, 744970). Cas12a RNP was reconstituted *in vitro* by adding 100 pmol of purified sgRNA to 1 μL of Cas12a (67 μM, Integrated DNA Technologies 10007923) in Opti-MEM (Gibco, 11058021) to a final volume of 10 μL at room temperature. 200,000 cells were pelleted, washed once with D-PBS, resuspended in 15 μL Opti-MEM, and added to the RNP mixture. The entire resulting volume was nucleofected in a Nucleocuvette well (Lonza) using a 4D-Nucleofector X Unit (Lonza, AAF-1003X) with program CM-130. Nucleofected cells were immediately transferred to 1-well in a 6-well plate containing 2 mL of prewarmed Dulbecco’s modified Eagle media (DMEM) supplemented with 10% FBS and 1x Pen-Strep. After 4 days, nucleofected cells were seeded at a density of 1 cell/well in 96-well plates. After 2 weeks of growth colonies were screened by Western blot detection of PKR (anti-PKR, rabbit, D7F7, CST, 12297) or HRI protein (anti-HRI, rabbit, Proteintech # 20499–1-AP), and colonies that did not express PKR or HRI were chosen. Indel formation was further validated using Sanger sequencing of the CRISPR-Cas12 targeted locus followed by ICE/TIDE analysis.

### Profiling Kinase Inhibitor Activity in Cells

Dabrafenib and compound **5a** were profiled against BRAF V600E in the A375 cell line and against BRAF WT in the HCT116 cell line. Cells were grown in DMEM (GIBCO) supplemented with 10% FBS, penicillin, and streptomycin. Cells were grown to 80–85% confluence prior to inhibitor treatment. Cells were treated as indicated with inhibitor or 0.1% DMSO solvent vehicle for 4 h. Treated cells were washed and scraped into PBS to harvest. Cell pellets were collected by centrifugation at 350*g* for 5 min and lysed in a buffer containing 50 mM HEPES, pH 7.4, 150 mM NaCl, 5 mM EDTA, 0.5% NP-40, 5 mM NaF, 10% glycerol, supplemented with fresh protease inhibitor cocktail (Roche 5056489001) and phosphatase inhibitor cocktail PhosStop (4906845001). Cell lysates were clarified by centrifugation at 18,000*g* for 30 min. Protein concentration of the supernatant was determined by Bradford assay (Bio-Rad). For Western blotting, protein was separated by sodium dodecyl sulfate–polyacrylamide gel electrophoresis (SDS–PAGE), transferred onto nitrocellulose membranes and subjected to a standard immunoblot protocol. Western blots were visualized using Bio-Rad Clarity ECL Western Blotting Substrate on a Bio-Rad ChemiDoc MP imaging system. The following antibodies were used for immunoblot analyses: pMEK (Santa Cruz sc-81503), total MEK (Cell Signaling 61B12), and HSP90 (Santa Cruz sc-13119).

Dabrafenib and compound **5a** were profiled against PKR, PERK, and HRI in HEK293T cells. HEK293T cells were maintained in DMEM + Glutamaplus (Wisent Biocenter, 319–415 CL) plus 10% fetal bovine serum (Wisent Biocenter, 090–150). All cell lines were routinely tested for mycoplasma contamination using the Mycoplasma PCR Detection Kit (abmGood, G238) and tested negative for mycoplasma. For PKR analysis, poly­(I:C) (Invivogen, tlrl-pic) was transfected in WT HEK293T or ΔPKR cells using polyethylenimine (PEI STAR, Tocris Biosciences, 7854) at a 1:6 ratio of poly­(I:C) (in μg) to PEI (in μL at a 1 mg/mL stock concentration). Cells were pretreated with dabrafenib, compound **5a**, or DMSO for 4 h prior to transfection of 1 μg of poly­(I:C) (in a 12-well plate) and harvested 6 h later. For PERK analysis, thapsigargin (1 μM) (Cayman Chemical, 10522–1) was added to cells pretreated with dabrafenib, compound **5a**, or DMSO for 4 h, and cells were harvested 4 h after thapsigargin addition. For HRI analysis, WT or ΔHRI cells were pretreated with compound **5a** for 4 h followed by treatment with oligomycin (1 μM) (CST #9996L) for 4 h, harvested, and flash frozen.

For Western blot analysis of whole cell lysates, cells were harvested at the indicated time points by washing in PBS, pelleting, and snap freezing. Cells were lysed in lysis buffer (150 mM NaCl, 50 mM HEPES pH 7.5, 1% NP-40 substitute) supplemented with Roche cOmplete Protease Inhibitor Cocktail (Sigma, 11836145001), PhosSTOP Phosphatase Inhibitor Cocktail (Roche, 4906837001), carfilzomib (2 μM, Cayman Chemicals, 17554–50), and benzonase (EMD Millipore, 70746–4) on ice. Samples were then normalized to protein concentration with the Pierce BCA Protein Assay kit (Thermo Fisher, A55865). 2x urea sample buffer (120 mM Tris pH 6.8, 4% SDS, 4 M urea, 20% glycerol, bromophenol blue) was then added to the samples. SDS–PAGE and immunoblotting were performed with the indicated antibodies. Images were captured on a Bio-Rad ChemiDoc instrument. The following antibodies were used for immunoblot analyses: anti-GAPDH (rabbit, D16H11, CST, 5174), anti-ATF4 (rabbit, D4B8, CST, 11815S), anti-PERK (rabbit, D11A8, CST, 5683), anti-PKR (rabbit, D7F7, CST, 12297), anti-phosphoPKR (rabbit, AB32036, abcam), anti-eIF2α (rabbit, D7D3, CST,5324), anti *p*-eIF2α (rabbit, D9G8, CST, 3398), and anti-HRI (rabbit, Proteintech # 20499–1-AP).

For GCN2 inhibition and activation profiling, HEK293T cells were cultured in DMEM (Gibco) supplemented with 10% fetal bovine serum and 1% penicillin–streptomycin. Cells were treated at 80% confluence with the indicated compound or 0.1% DMSO vehicle control for 4 h. For cotreatment conditions with halofuginone, cells were pretreated with dabrafenib or compound **5a** for 30 min, followed by cotreatment with halofuginone for an additional 3 h 30 min. Cells were then scraped, washed with PBS, and collected by centrifugation at 500*g* for 5 min at 4 °C. Cell pellets were lysed in 1× SDS sample buffer containing 58.3 mM Tris–HCl (pH 6.8), 5% glycerol, 1.67% SDS, 97 mM DTT, and 0.002% bromophenol blue, supplemented immediately before use with SuperNuclease (Sino Biological). Samples were boiled for 5 min, and protein concentrations were determined using a BCA assay supplemented with ionic detergent compatibility reagent powder, according to the manufacturer’s instructions. Equal amounts of protein were separated by SDS–PAGE and transferred to PVDF membranes for standard immunoblot analysis. Primary antibodies used were phospho-GCN2 (Abcam, ab75836), GCN2 (Cell Signaling Technology, 3302S), ATF4 (Cell Signaling Technology, 11815S), and α-tubulin (Santa Cruz Biotechnology, sc-32293).

### In Vitro Profiling against a Diversity Kinase Panel at Eurofins

Compound **5a** and dabrafenib were tested at a single concentration of 1 μM of each compound in the Eurofins Diversity Kinase [Km ATP] KinaseProfiler LeadHunter Panel (CAT# 50–015 KP), at each kinases’ respective [Km] ATP.

### Rapid PK Analysis

In vivo BBB penetration: All animal experiments were conducted in accordance with CCAC guidelines and approved by the UHN Animal Care Committee. Animal Use Protocol # 7040.1. Male NOD-SCID mice received a single oral dose of OICR-0403184 (10 mg/kg) formulated in 5% DMSO, 47.5% PEG 400, and 47.5% aqueous solution (10% Tween-80 in water). Three biological replicates were analyzed per time point. At 2 and 4 h post dose, blood was collected by cardiac puncture into K_2_-EDTA tubes and centrifuged to obtain plasma; mice were then transcardially perfused with PBS prior to brain excision, snap-freezing, and storage at – 80 °C until analysis. Brain tissues were homogenized in water (1:2, *w*/*v*) using a Precellys 24 homogenizer. Protein precipitation was performed by adding acetonitrile containing an internal standard to plasma or brain homogenate samples, followed by centrifugation. Supernatants were transferred to LC–MS vials for analysis. Calibration standards were prepared in blank plasma or brain homogenate from treatment-naïve mice. Quantitative LC–MS/MS analysis was carried out using an Agilent 1200 high-performance liquid chromatography (HPLC) system coupled to a Sciex QTRAP 5500 mass spectrometer. Separation was achieved on a Waters XSelect BEH C18 column (2.1 × 50 mm, 3.5 μm) at a flow rate of 0.500 mL/min. The gradient increased from 10% to 95% solvent B over 1.5 min, was held until 2.0 min, returned to 10% B by 2.5 min, and maintained until 5.5 min for re-equilibration. Data were acquired and processed using Analyst software (v1.7).

### Synthetic Procedures

General Information: all chemical reagents were purchased from commercial vendors and used without further purification. Volatiles were removed under reduced pressure by using either rotary evaporation or the Biotage V-10 solvent evaporator system. Very high boiling point (6000 rpm, 0 mbar, 56 °C), mixed volatile (7000 rpm, 30 mbar, 36 °C), and volatile (6000 rpm, 30 mbar, 36 °C) methods were used to evaporate solvents. The yields given refer to chromatographically purified and spectroscopically pure compounds, unless otherwise stated. Compounds were purified using Teledyne ISCO Combi Flash Rf system. Normal phase chromatography was performed using Teledyne ISCO RediSep Rf silica, and the reverse-phase chromatography was performed using Teledyne ISCO RediSep Rf C18 columns. Preparative chromatography was carried out using a Waters Autopure system with 3767 sample manager (injector/collector) attached to PDA UV/vis and single quadrapole mass spectrometer. For compound purification, either an XSelect CSH Prep C18 column (5 μm, OBD, 19 × 100 mm) or an XBridge Prep C8 column (5 μm, 10 × 100 mm) was utilized. The final compounds were dried using the Labconco Benchtop FreeZone freeze-dry system (4.5 L model) or left overnight over high vacuum. ^1^H NMR spectra were recorded on a Bruker AVANCE-III 500 MHz spectrometer at ambient temperature. Residual protons of DMSO-*d*_6_ and CD_3_OD solvents were used as internal references. Spectral data are reported as follows: chemical shift (δ in ppm), multiplicity (br = broad, s = singlet, d = doublet, dd = doublet of doublets, t = triplet, m = multiplet), coupling constants (*J* in Hz), and proton integration. Compound purity and low-resolution mass spectrometry analyses were conducted using a Waters Acquity UPLC system equipped with a photodiode array (PDA) detector and an SQD mass spectrometer. Purity was determined by UV absorbance at 254 nm, with all final compounds exhibiting a purity of ≥95%. Mass spectrometry was performed in both positive and negative ion modes using electrospray ionization (ESI). Mobile phase A consisted of 0.1% formic acid in water, whereas mobile phase B consisted of 0.1% formic acid in acetonitrile. One column was used: Waters Acquity UPLC HSS T3 (2.1 × 50 mm, 100 Å, 1.8 μm). Method 1: the gradient went from 98% to 5% mobile phase A over 1.8 min, maintained at 5% for 0.5 min, then increased to 98% over 0.2 min for a total run time of 3 min. Method 2: the gradient went from 98% to 5% mobile phase A over 8.0 min, maintained at 5% for 1.0 min, then increased to 98% over 1.0 min for a total run time of 10 min. The flow rate was 0.4 mL/min throughout both runs. All columns were used with the temperature maintained at 30 °C. High-resolution mass spectra (HRMS) were acquired using an Orbitrap Exploris 240 (Thermo Fisher Scientific) in positive ion mode across an m/z range of 100–1000. Instrument settings included a vaporizer temperature of 250 °C, ion transfer tube temperature of 325 °C, capillary voltage of 3500 V, and RF lens voltage at 70%. Sheath, auxiliary, and sweep gas flows were set to 50, 10, and 1 arbitrary units, respectively. The Orbitrap resolution was 120,000 fwhm at m/z 200, with a scan rate of 3 Hz, automatic maximum injection time, and microscan set to 1. Data acquisition was performed using TraceFinder 5.1, and analysis was conducted in FreeStyle 1.8 (Thermo Fisher Scientific, Waltham, Massachusetts, USA).

### General Synthesis

General synthesis of compounds **3a–e**: in a vial, methyl 3-(2,6-difluorophenylsulfonamido)-2-fluorobenzoate (300 mg, 0.869 mmol) was placed, and THF (10 mL) was added under argon. The vial was placed in an ice bath (0 °C), and lithium bis­(trimethylsilyl)­amide (1 M in THF, 2.87 mL, 2.87 mmol) was added. To this reaction mixture, substituted chloropyrimidines (**2a–e**, 1.086 mmol) in THF (1 mL) were added dropwise using a syringe. After the completion of the addition, the reaction was allowed to warm up to rt and then stirred for 2–3 h. The volume was reduced to half under reduced pressure, and 1N aq HCl was added. The layers were separated using EtOAc (2 × 100 mL), and the combined organic layer was washed once with brine, dried (MgSO_4_), and concentrated under reduced pressure. The crude material for **3a** and **3c** was further purified by flash column chromatography using hexanes/EtOAc to obtain products as a mixture of keto and enol isomers in 60 and 76% yields, respectively. Compounds **3b**, **3d**, and **3e** were obtained as a mixture of keto and enol isomers as off-white solids, confirmed by LCMS analysis and were used further for the next step without further purification.

#### *N*-(3-(2-(2-Chloro-6-methylpyrimidin-4-yl)­acetyl)-2-fluorophenyl)-2,6-difluorobenzenesulfonamide (**3a**)

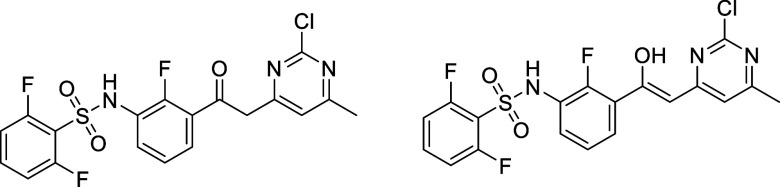



^1^H NMR (500 MHz, DMSO-*d*_6_, keto–enol mixture) δ ppm 13.46 (brs, 1 H), 10.90–11.05 (m, 2 H), 7.75 (t, J = 6.11 Hz, 3 H), 7.62–7.67 (m, 1 H), 7.55–7.60 (m, 1 H), 7.41 (s, 2 H), 7.35 (s, 1 H), 7.31–7.34 (m, 1 H), 7.26–7.31 (m, 4 H), 6.06 (s, 1 H), 4.42 (s, 2 H), 2.48 (s, 3 H), 2.45 (s, 3 H); 60% yield. ESI-MS (m/z): [M + H]^+^ calcd for C_19_H_13_ClF_3_N_3_O_3_S, 456.04; found, 456.26.

#### *N*-(3-(2-(2-Chloro-6-ethylpyrimidin-4-yl)­acetyl)-2-fluorophenyl)-2,6-difluorobenzenesulfonamide (**3c**)

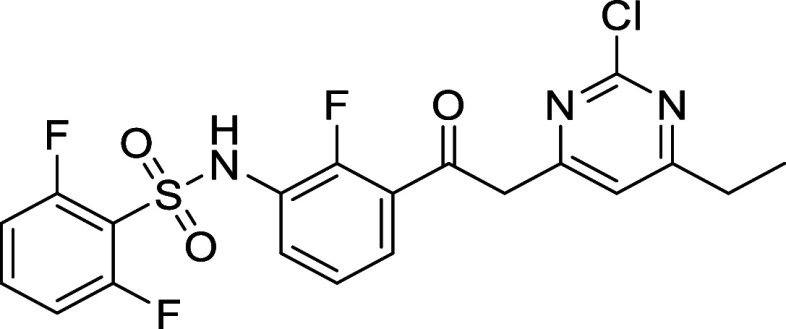



^1^H NMR (500 MHz, DMSO-*d*_6_, keto–enol mixture) δ ppm 13.47 (brs, 1 H), 10.94 (brs, 2 H), 7.73 (d, J = 4.89 Hz, 3 H), 7.62–7.66 (m, 1 H), 7.53–7.58 (m, 1 H), 7.40–7.44 (m, 2 H), 7.36 (s, 1 H), 7.31–7.34 (m, 1 H), 7.26–7.30 (m, 4 H), 7.20–7.25 (m, 1 H), 6.09 (s, 1 H), 4.42 (s, 2 H), 2.68–2.79 (m, 4 H), 1.18–1.24 (m, 6 H); 76% yield. ESI-MS (m/z): [M + H]^+^ calcd for C_20_H_15_ClF_3_N_3_O_3_S, 470.06; found, 470.10.

General synthesis of compounds **4a**–**e**: to a solution of compounds **3a–e** (1.0 equiv, 0.22 mmol) in DMA (2 mL)­NBS (1.0 equiv, 0.22 mmol) was added, and the reaction mixture was allowed to stir for 15 min at rt. 2,2-Dimethylthiopropionamide (1.0 equiv, 0.22 mmol) was then added to the reaction mixture at rt. The reaction vial was then heated at 80 °C for 1–2 h. The reaction mixture was diluted with H_2_O and extracted with EtOAc. The combined organic layer was washed with H_2_O two times to remove DMA, dried (MgSO_4_), filtered, and concentrated under reduced pressure. The crude material was purified by flash column chromatography using hexanes/EtOAc. The desired products **4a**, **4c**, and **4e** were obtained in 55–78% yield. Compounds **4b** and **4d** were obtained as pale-yellow solids, confirmed by LCMS analysis and were used for the next step without further purification.

#### *N*-(3-(2-(*tert*-Butyl)-5-(2-chloro-6-methylpyrimidin-4-yl)­thiazol-4-yl)-2-fluorophenyl)-2,6-difluorobenzenesulfonamide (**4a**)

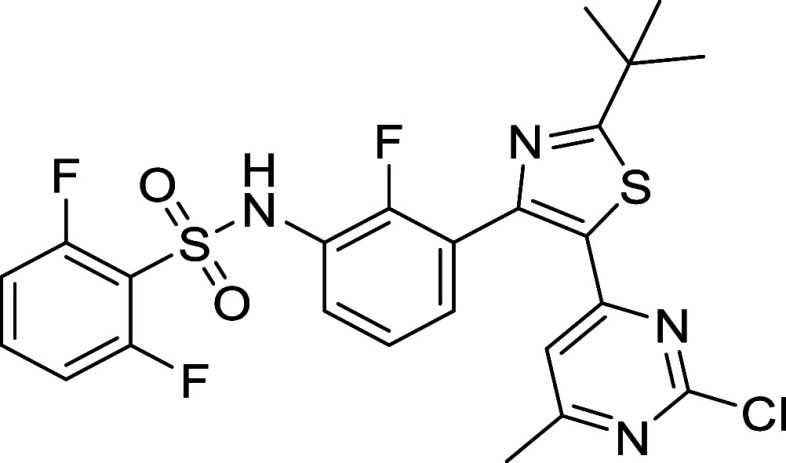



^1^H NMR (500 MHz, DMSO-*d*_6_) δ ppm 10.90 (s, 1 H), 7.66–7.73 (m, 1 H), 7.46–7.50 (m, 1 H), 7.37–7.42 (m, 1 H), 7.31–7.35 (m, 1 H), 7.21–7.26 (m, 2 H), 6.83 (s, 1 H), 2.32 (s, 3 H), 1.43 (s, 9 H). 78% yield. ESI-MS (m/z): [M + H]^+^ calcd for C_24_H_20_ClF_3_N_4_O_2_S_2_, 553.07; found, 553.31.

#### *N*-(3-(2-(*tert*-Butyl)-5-(2-chloro-6-ethylpyrimidin-4-yl)­thiazol-4-yl)-2-fluorophenyl)-2,6-difluorobenzenesulfonamide (**4c**)

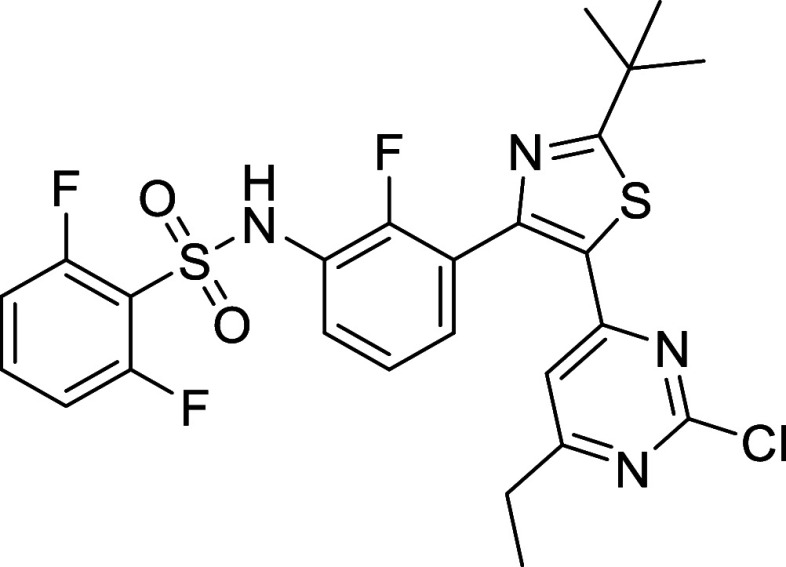



^1^H NMR (500 MHz, DMSO-*d*_6_) δ ppm 10.92 (s, 1 H), 7.67–7.73 (m, 1 H), 7.41–7.49 (m, 2 H), 7.31–7.36 (m, 1 H), 7.21–7.26 (m, 2 H), 6.79 (s, 1H), 2.55–2.62 (m, 2 H), 1.44 (s, 9 H), 0.98–1.04 (m, 3 H). 60% yield. ESI-MS (m/z): [M + H]^+^ calcd for C_25_H_22_ClF_3_N_4_O_2_S_2_, 567.09; found, 567.08.

#### *N*-(3-(2-(*tert*-Butyl)-5-(2-chloro-5-fluoropyrimidin-4-yl)­thiazol-4-yl)-2-fluorophenyl)-2,6-difluorobenzenesulfonamide (**4e**)

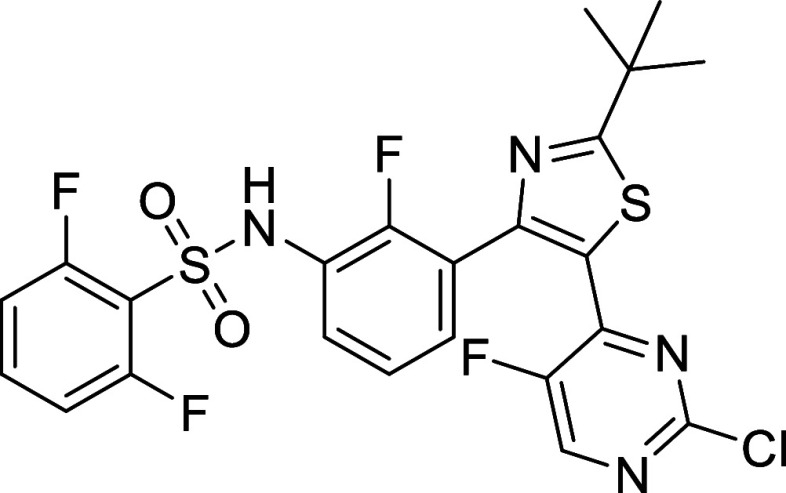



^1^H NMR (500 MHz, DMSO-*d*_6_) δ ppm 8.80 (d, J = 2.20 Hz, 1 H), 7.66–7.74 (m, 1 H), 7.43–7.50 (m, 1 H), 7.36–7.40 (m, 1 H), 7.21–7.28 (m, 3H), 1.46 (s, 9 H). 55% yield. ESI-MS (*m*/*z*): [M + H]^+^ calcd for C_23_H_17_ClF_4_N_4_O_2_S_2_, 557.05; found, 557.30.

General synthesis of compound **5a**–**f**: Method A: a suspension of **4a**/**4e** (0.099 mmol) and NH_3_ (7 N in MeOH, 15.42 mmol) was heated in a sealed tube at 90 °C for 24 h. The product was formed only in 20%. The reaction mixture was heated further in a microwave reactor at 120 °C for 1 h. The solvent was removed. The crude product was extracted using CH_2_Cl_2_, and organic layer was washed with std aq NaHCO_3_. The CH_2_Cl_2_ layer was dried (MgSO_4_), filtered, and concentrated under reduced pressure. The crude was purified by flash column chromatography using hexanes/EtOAc to obtain the pure product **5a** and **5e** in 71% and 65% yields, respectively.

Method B: in a microwave, vial **4b**/**4c**/**4d** (0.096 mmol) was placed, and aq NH_4_OH solution (30–33%, 48 mmol) was added. The reaction mixture was heated at 120 °C for 1 h in a microwave reactor. Reaction mixture was diluted with 5% aq. HCl, and the product was extracted using CH_2_Cl_2_. Organic layer was dried (MgSO_4_), filtered, and concentrated under reduced pressure. Hexanes/EtOAc were used to purify by flash column chromatography. The desired products (**5b**, **5c**, and **5d**) were eluted around 55–60% EtOAc/hexanes and were obtained in 45–60% yields.

Method C: a suspension of **4a** (112 mg, 0.20 mmol) and MeNH_2_ (2 M in THF, 0.55 mL, 1.1 mmol) was heated at 60 °C overnight. The reaction was diluted with 1 N aq HCl, and the crude product was extracted with CH_2_Cl_2_, dried (MgSO_4_), and concentrated under reduced pressure. The crude material was purified by prep HPLC. The desired product **5f**, along with a side product **5g**, were isolated in 40% and 10% yields, respectively.

#### *N*-(3-(5-(2-Amino-6-methylpyrimidin-4-yl)-2-(*tert*-butyl)­thiazol-4-yl)-2-fluorophenyl)-2,6-difluorobenzenesulfonamide (**5a**, OICR-403184)

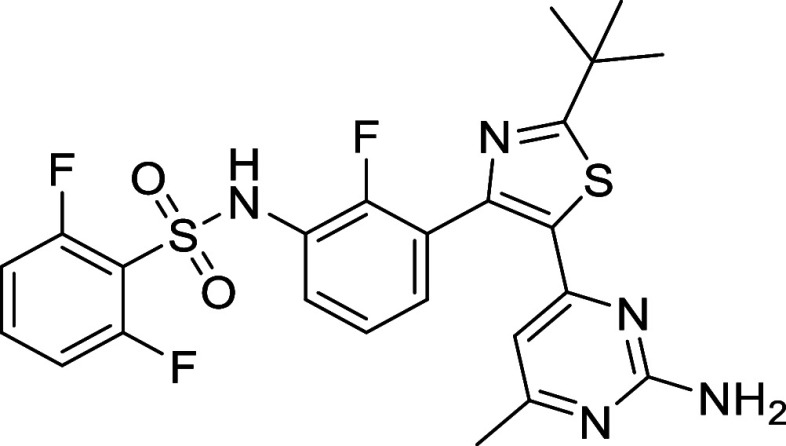



^1^H NMR (500 MHz, *CD*_3_ *OD*) δ ppm 7.53–7.64 (m, 2 H), 7.31–7.36 (m, 1 H), 7.25–7.30 (m, 1 H), 7.08 (t, J = 8.80 Hz, 2 H), 6.05 (s, 1 H), 2.12 (s, 3 H), 1.48 (s, 9 H). ^13^C NMR (*CD*_3_ *OD*, 126 MHz) δ 182.9, 168.5, 163.0, 160.6, 158.5, 154.1, 152.1, 145.8, 135.1 (d, ^2^ *J*_CF_ = 60 Hz), 134.0, 128.5, 126.4, 124.3, 124.3, 124.1 (d, ^2^ *J*_CF_ = 60 Hz), 112.7 (dd, ^2^ *J*_CF_ = 15 Hz, ^2^ *J*_CF_ = 80 Hz), 105.9, 37.6, 29.5, 22.4. ^19^F NMR (471 MHz, *CD*_3_ *OD*) δ ppm −108.64 (s, 2 F), −127.14 (brs, 1 F); 71% yield; 96% purity. ESI-MS (*m*/*z*): [M + H]^+^ calcd for C_24_H_22_F_3_N_5_O_2_S_2_, 534.12; found, 534.28. HRMS (*m*/*z*): [M + H]^+^ calcd for C_24_H_22_F_3_N_5_O_2_S_2_, 534.1240; found 534.1235.

#### *N*-(3-(2-(*tert*-Butyl)-5-(2,6-diaminopyrimidin-4-yl)­thiazol-4-yl)-2-fluorophenyl)-2,6-difluorobenzenesulfonamide (**5b**)

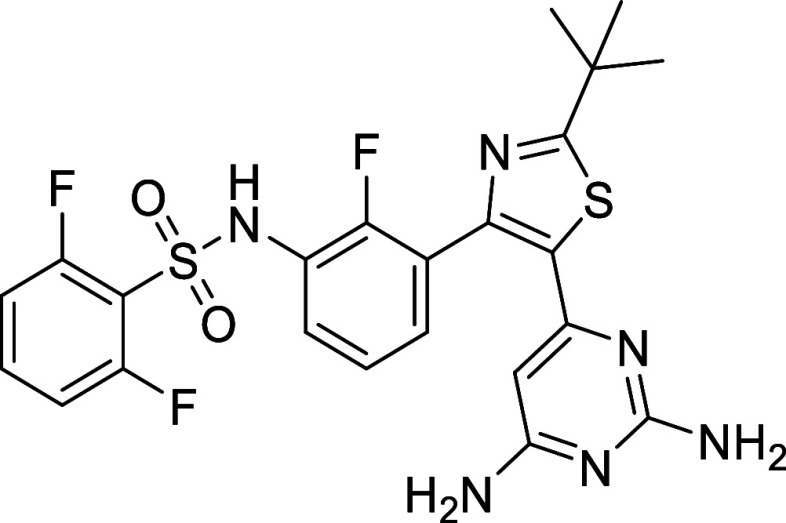



^1^H NMR (500 MHz, DMSO-*d*_6_) δ ppm 10.88 (brs, 1 H), 7.68 (brs, 1 H), 7.31–7.41 (m, 2 H), 7.21–7.26 (m, 3 H), 6.19 (brs, 2 H), 5.95 (brs, 2 H), 5.39 (s, 1 H), 1.39 (s, 9 H); 45% yield; 96% purity. ESI-MS (*m*/*z*): [M + H]^+^ calcd for C_23_H_21_F_3_N_6_O_2_S_2_, 535.12; found, 535.18.

#### *N*-(3-(5-(2-Amino-6-ethylpyrimidin-4-yl)-2-(*tert*-butyl)­thiazol-4-yl)-2-fluorophenyl)-2,6-difluorobenzenesulfonamide (**5c**)

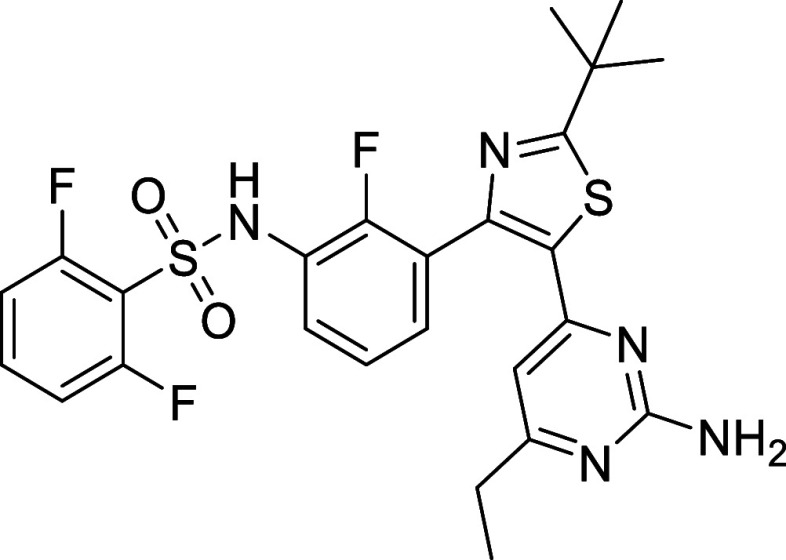



^1^H NMR (500 MHz, *CD*_3_ *OD*) δ ppm 7.56–7.66 (m, 2 H), 7.24–7.35 (m, 2 H), 7.05–7.11 (m, 2 H), 6.05 (s, 1 H), 2.36–2.43 (m, 2 H), 1.49 (s, 9 H), 0.98–1.03 (m, 3 H); 60% yield and 98% purity. ESI-MS (*m*/*z*): [M + H]^+^ calcd for C_25_H_24_F_3_N_5_O_2_S_2_, 548.14; found, 548.20.

#### *N*-(3-(5-(2-Amino-6-methoxypyrimidin-4-yl)-2-(*tert*-butyl)­thiazol-4-yl)-2-fluorophenyl)-2,6-difluorobenzenesulfonamide (**5d**)

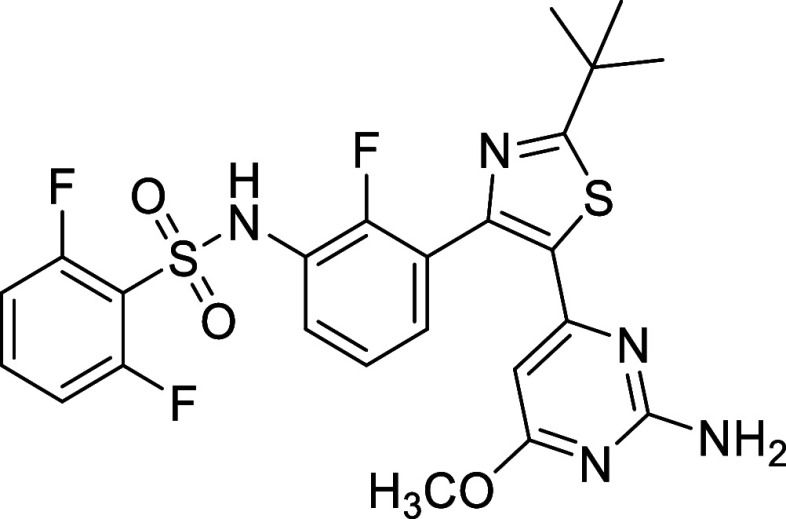



H NMR (500 MHz, *CD*_3_ *OD*) δ ppm 7.56–7.65 (m, 2 H), 7.23–7.30 (m, 2 H), 7.03–7.08 (m, 2 H), 5.49 (s, 1 H), 3.80 (s, 3 H), 1.47 (s, 9 H); 55% yield and 95% purity. ESI-MS (*m*/*z*): [M + H]^+^ calcd for C_24_H_22_F_3_N_5_O_3_S_2_, 550.12; found, 550.08.

#### *N*-(3-(5-(2-Amino-5-fluoropyrimidin-4-yl)-2-(*tert*-butyl)­thiazol-4-yl)-2-fluorophenyl)-2,6-difluorobenzenesulfonamide (**5e**)

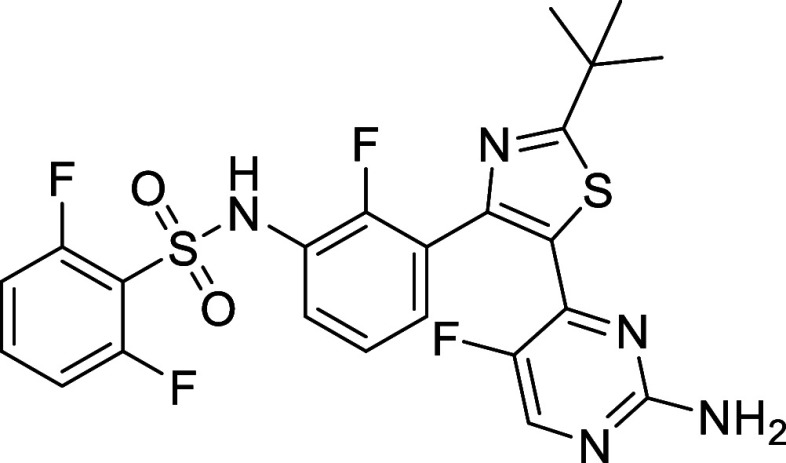



^1^H NMR (500 MHz, *CD*_3_ *OD*) δ ppm 8.05 (d, J = 2.69 Hz, 1 H), 7.58–7.65 (m, 1 H), 7.49–7.54 (m, 1 H), 7.35–7.40 (m, 1 H), 7.16–7.21 (m, 1 H), 7.07–7.12 (m, 2 H), 1.51 (s, 9 H); 65% yield and 96% purity. ESI-MS (*m*/*z*): [M + H]^+^ calcd for C_23_H_19_F_4_N_5_O_2_S_2_, 538.10; found, 538.12.

#### *N*-(3-(2-(*tert*-Butyl)-5-(6-methyl-2-(methylamino)­pyrimidin-4-yl)­thiazol-4-yl)-2-fluorophenyl)-2,6-difluorobenzenesulfonamide (**5f**)

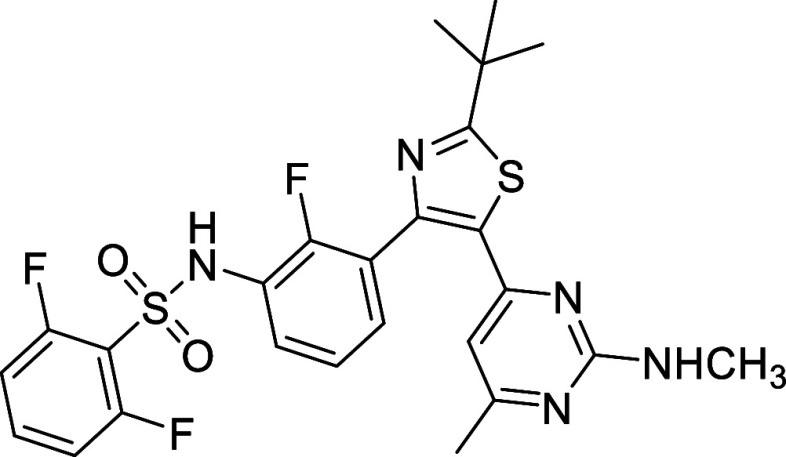



^–1^H NMR (500 MHz, *CD*_3_ *OD*) δ ppm 7.41–7.51 (m, 2 H), 7.11–7.22 (m, 2 H), 6.93–6.99 (m, 2 H), 5.96 (brs, 1 H), 2.69 (brs, 3 H), 2.03 (s, 3 H), 1.37 (s, 9 H); 40% yield and 99% purity. ESI-MS (*m*/*z*): [M + H]^+^ calcd for C_25_H_24_F_3_N_5_O_2_S_2_, 548.14; found, 548.12. ^19^F NMR (471 MHz, *CD*_3_ *OD*) δ ppm −108.61 (d, J = 3.47 Hz, 2 F), −127.46 (brs, 1 F).

#### *N*-(3-(2-(*tert*-Butyl)-5-(6-methyl-2-(methylamino)­pyrimidin-4-yl)­thiazol-4-yl)-2-fluorophenyl)-2-fluoro-6-(methylamino)­benzenesulfonamide (**5g**)

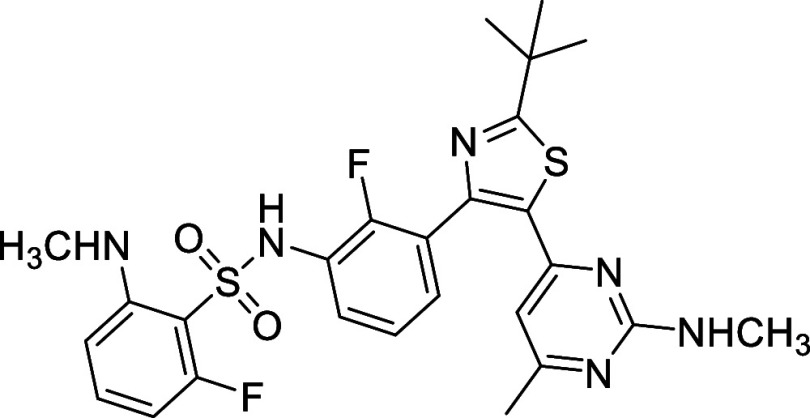



^1^H NMR (500 MHz, *CD*_3_ *OD*) δ ppm 7.48–7.53 (m, 1 H), 7.28–7.36 (m, 1 H), 7.18–7.26 (m, 2 H), 6.52 (d, J = 8.80 Hz, 1 H), 6.29–6.35 (m, 1 H), 6.08 (brs, 1 H), 2.85 (s, 3 H), 2.80 (brs, 3 H), 2.13 (s, 3 H), 1.49 (s, 9 H); 10% yield and 97% purity. ESI-MS (*m*/*z*): [M + H]^+^ calcd for C_26_H_28_F_2_N_6_O_2_S_2_, 559.18; found, 559.18. ^19^F NMR (471 MHz, *CD*_3_ *OD*) δ ppm −108.63 (d, J = 3.47 Hz, 1 F), −128.65 (brs, 1 F).

General synthesis of compounds **7** and **10**: to a screw cap vial was added dabrafenib (**6**)/*N*-(3-(5-(2-amino-6-methylpyrimidin-4-yl)-2-(*tert*-butyl)­thiazol-4-yl)-2-fluorophenyl)-2,6-difluorobenzenesulfonamide (**5a**), (1.0 equiv, 0.50 mmol), anhydrous THF (1.25 mL, ethyl benzoylformate (1.1 equiv, 0.55 mmol), and tris­(dimethylamino)­phosphine (1.2 equiv, 0.60 mmol). The reaction was stirred at rt for 1 h, after which 2-*tert*-butyl-1,1,3,3-tetramethylguanidine (1.25 equiv, 0.63 mmol) was added, and the resulting mixture was heated to 65 °C for 4–5 h to induce the elimination/fragmentation reaction. The reaction mixture from the N–S cleavage step was cooled to rt and treated with NH_2_OH (50% wt in H_2_O) (2.0 equiv, 1.001 mmol). The full conversion of the imine to the free amine was ensured by keeping the reaction mixture at room temperature for 1 h. THF was removed under reduced pressure, and the residue was extracted using EtOAc. The combined organic layer was dried (MgSO_4_), and the solvent was removed under reduced pressure. Purification was performed using flash chromatography on silica gel using 4–5% CH_2_Cl_2_ in MeOH to afford intermediates **7** and **10**.

#### 4-(4-(3-Amino-2-fluorophenyl)-2-(*tert*-butyl)­thiazol-5-yl)­pyrimidin-2-amine (**7**)

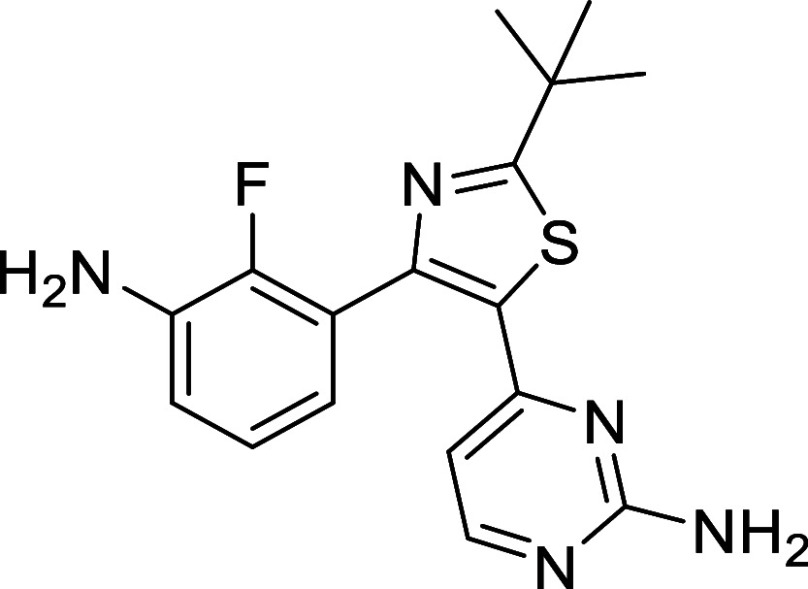



^1^H NMR (500 MHz, *CD*_3_ *OD*) δ ppm 8.00 (d, J = 5.26 Hz, 1 H), 7.02–7.08 (m, 1 H), 6.95–7.01 (m, 1 H), 6.69–6.75 (m, 1 H), 6.30 (d, J = 5.26 Hz, 1 H), 1.50 (s, 9 H); 60% yield. ESI-MS (*m*/*z*):: [M + H]^+^ calcd for C_17_H_18_FN_5_S, 344.13; found, 344.40.

#### 4-(4-(3-Amino-2-fluorophenyl)-2-(*tert*-butyl)­thiazol-5-yl)-6-methylpyrimidin-2-amine (**10**)

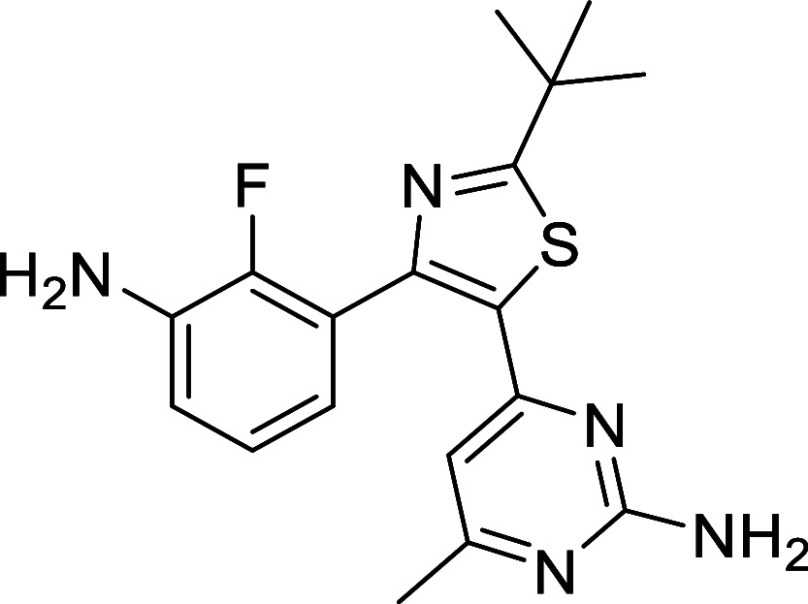



^1^H NMR (500 MHz, *CD*_3_ *OD*) δ ppm 7.04–7.08 (m, 1 H), 6.97–7.02 (m, 1 H), 6.71–6.75 (m, 1 H), 6.20 (s, 1 H), 2.11 (s, 3 H), 1.51 (s, 9H).; 65% yield. ESI-MS (*m*/*z*): [M + H]^+^ calcd for C_18_H_20_FN_5_S, 358.15; found, 358.24.

General synthesis of compounds **9a**–**g**/**11**: a mixture of intermediate **7**/**10** (1.0 equiv, 0.116 mmol), substituted aryl sulfonyl chlorides (**8a–g/8b**, 0.175 mmol), and CH_2_Cl_2_ (1 mL) was stirred at rt before pyridine (0.349 mmol) was added, and the reaction mixture was stirred at rt for 12–15 h. The crude product was extracted using CH_2_Cl_2_ (2 × 30 mL)/H_2_O, and the combined organic layer was washed with H_2_O three times (3 × 30 mL), dried (MgSO_4_), and concentrated under reduced pressure. The crude product was purified by either flash chromatography on silica gel using 4–5% of CH_2_Cl_2_ in MeOH or prep HPLC to afford final products **9a**–**g** and **11** in 40–96% yield and 70% yield, respectively.

#### *N*-(4-(4-(3-Amino-2-fluorophenyl)-2-(*tert*-butyl)­thiazol-5-yl)­pyrimidin-2-yl)-2-chloro-6-fluorobenzenesulfonamide (**9a**)

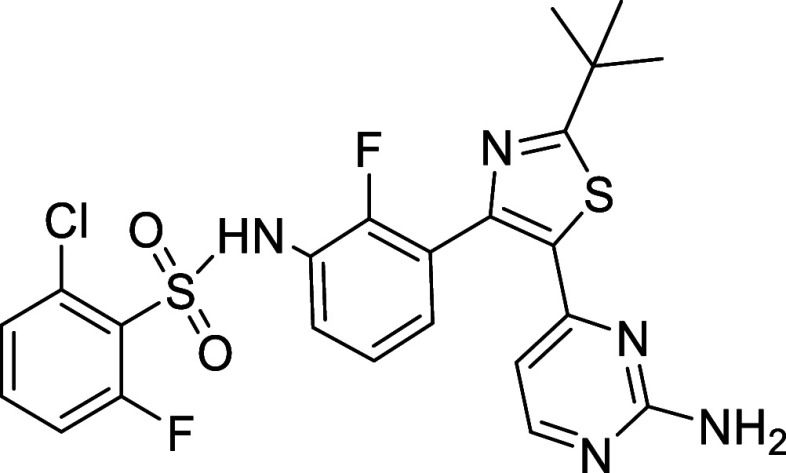



^1^H NMR (500 MHz, *CD*_3_ *OD*) δ ppm 7.95 (d, J = 5.26 Hz, 1 H), 7.57–7.63 (m, 1 H), 7.51–7.56 (m, 1 H), 7.39 (d, J = 8.19 Hz, 1 H), 7.29–7.33 (m, 1 H), 7.27 (d, J = 7.95 Hz, 1 H), 7.19–7.25 (m, 1 H), 6.05 (d, J = 5.26 Hz, 1 H), 1.48 (s, 9 H); 50% yield; 97% purity. ESI-MS (*m*/*z*): [M + H]^+^ calcd for C_23_H_20_ClF_2_N_5_O_2_S_2_, 536.08; found, 536.16.

#### *N*-(4-(4-(3-Amino-2-fluorophenyl)-2-(*tert*-butyl)­thiazol-5-yl)­pyrimidin-2-yl)-2,5-difluorobenzenesulfonamide (**9b**)

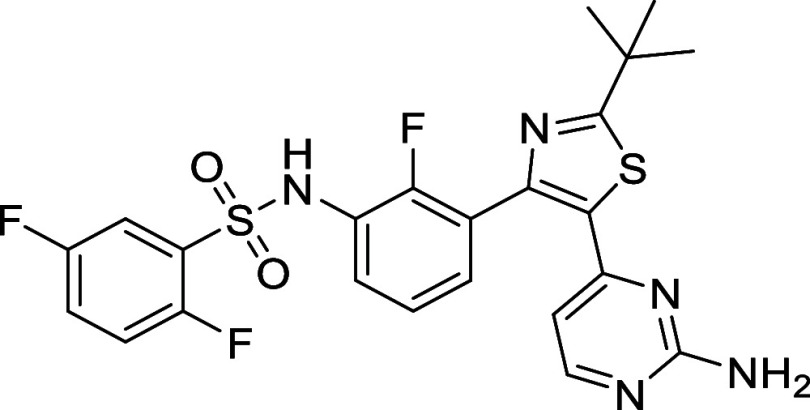



^1^H NMR (500 MHz, *CD*_3_ *OD*) δ ppm 7.94 (d, J = 5.38 Hz, 1 H), 7.58–7.63 (m, 1 H), 7.50–7.55 (m, 1 H), 7.35–7.41 (m, 1 H), 7.26–7.34 (m, 3 H), 6.03 (d, J = 5.38 Hz, 1 H), 1.48 (s, 9 H); 40% yield; 97% purity. ESI-MS (*m*/*z*): [M + H]^+^ calcd for C_23_H_20_F_3_N_5_O_2_S_2_, 520.11; found, 520.21.

#### *N*-(3-(5-(2-Aminopyrimidin-4-yl)-2-(*tert*-butyl)­thiazol-4-yl)-2-fluorophenyl)-2,3-difluoro-5-methylbenzenesulfonamide (**9c**)

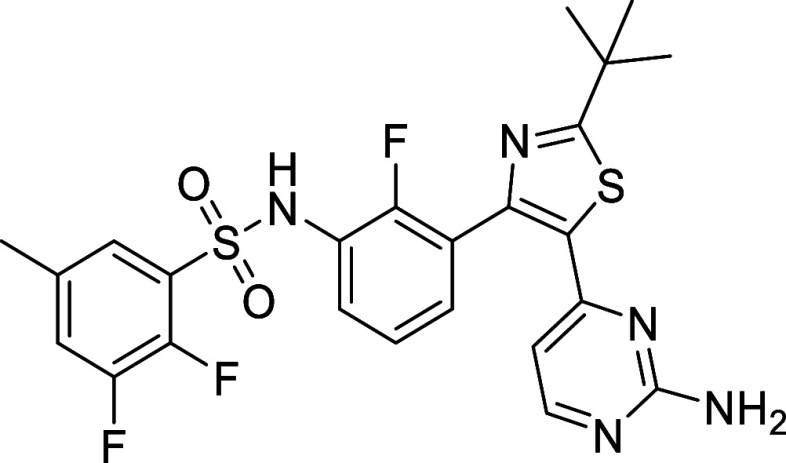



^1^H NMR (500 MHz, *CD*_3_ *OD*) δ ppm 7.93 (d, J = 5.62 Hz, 1 H), 7.52–7.57 (m, 1 H), 7.39 (d, J = 4.40 Hz, 1 H), 7.33–7.38 (m, 2 H), 7.24–7.29 (m, 1 H), 6.18 (d, J = 5.75 Hz, 1 H), 2.33 (s, 3 H), 1.47 (s, 9 H); 87% yield; 98% purity. ESI-MS (*m*/*z*): [M + H]^+^ calcd for C_24_H_22_F_3_N_5_O_2_S_2_, 534.12; found, 534.39.

#### *N*-(3-(5-(2-Aminopyrimidin-4-yl)-2-(*tert*-butyl)­thiazol-4-yl)-2-fluorophenyl)-3,5-dichloro-2-fluorobenzenesulfonamide (**9d**)

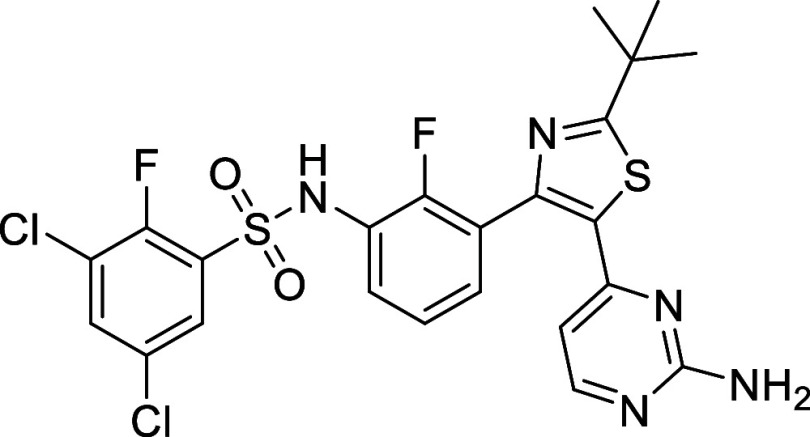



^1^H NMR (500 MHz, *CD*_3_ *OD*) δ ppm 7.96 (d, J = 5.01 Hz, 1 H), 7.86 (d, J = 8.56 Hz, 1 H), 7.80 (d, J = 6.11 Hz, 1 H), 7.55–7.61 (m, 1 H), 7.31–7.36 (m, 1 H), 7.25–7.30 (m, 1 H), 6.05 (d, J = 5.26 Hz, 1 H), 1.48 (s, 9 H); 85% yield; 95% purity. ESI-MS (*m*/*z*): [M + H]^+^ calcd for C_23_H_19_Cl_2_F_2_N_5_O_2_S_2_, 570.04; found, 570.32.

#### *N*-(4-(4-(3-Amino-2-fluorophenyl)-2-(*tert*-butyl)­thiazol-5-yl)­pyrimidin-2-yl)-2,3-dichloro-6-fluorobenzenesulfonamide (**9e**)

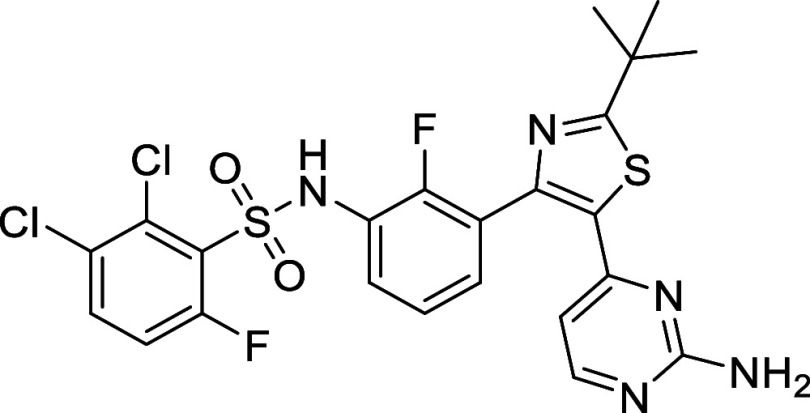



^1^H NMR (500 MHz, *CD*_3_ *OD*) δ ppm 7.84 (d, J = 4.89 Hz, 1 H), 7.66–7.69 (m, 1 H), 7.45–7.50 (m, 1 H), 7.19–7.25 (m, 1 H), 7.11–7.18 (m, 2 H), 5.94 (d, J = 5.26 Hz, 1 H), 1.37 (s, 9 H); 88% yield; 96% purity. ESI-MS (*m*/*z*): [M + H]^+^ calcd for C_23_H_19_Cl_2_F_2_N_5_O_2_S_2_, 570.04; found, 570.17.

#### *N*-(3-(5-(2-Aminopyrimidin-4-yl)-2-(*tert*-butyl)­thiazol-4-yl)-2-fluorophenyl)-2,6-difluoro-4-methylbenzenesulfonamide (**9f**)

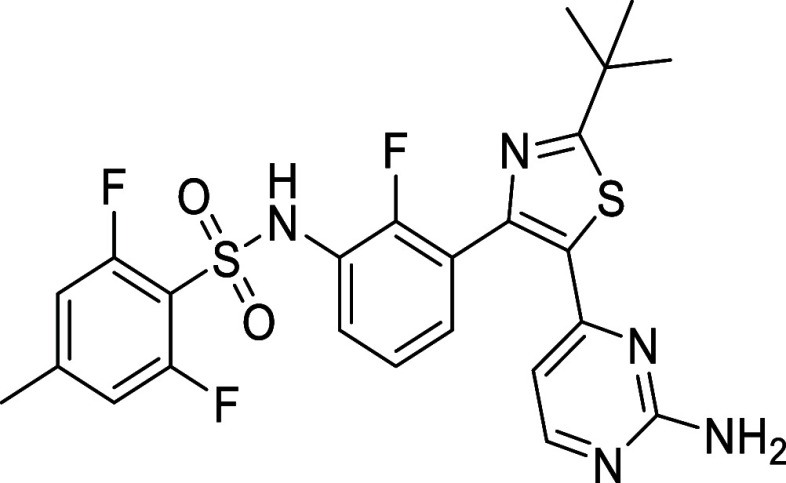



^1^H NMR (500 MHz, *CD*_3_ *OD*) δ ppm 7.93 (d, J = 5.14 Hz, 1 H), 7.59–7.64 (m, 1 H), 7.30–7.36 (m, 1 H), 7.25–7.30 (m, 1 H), 6.88–6.97 (m, 2 H), 6.03 (d, J = 5.14 Hz, 1 H), 2.37 (s, 3 H), 1.48 (s, 9 H); 96% yield; 97% purity. ESI-MS (*m*/*z*): [M + H]^+^ calcd for C_24_H_22_F_3_N_5_O_2_S_2_, 534.12; found, 534.36.

#### *N*-(3-(5-(2-Aminopyrimidin-4-yl)-2-(*tert*-butyl)­thiazol-4-yl)-2-fluorophenyl)-2-fluoro-4,6-dimethylbenzenesulfonamide (**9g**)

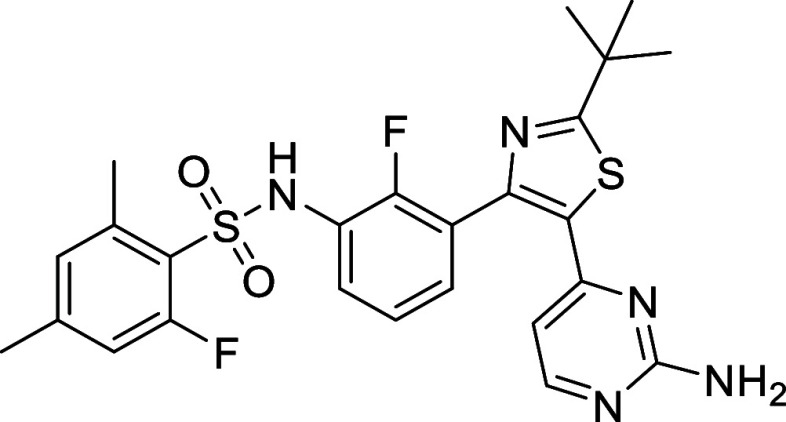



^1^H NMR (500 MHz, *CD*_3_ *OD*) δ ppm 7.90 (d, J = 5.14 Hz, 1 H), 7.57–7.63 (m, 1 H), 7.22–7.29 (m, 2 H), 6.94 (s, 1 H), 6.86–6.92 (m, 1 H), 5.96 (d, J = 5.26 Hz, 1 H), 2.54 (s, 3 H), 2.31 (s, 3 H), 1.48 (s, 9 H); 86% yield; 96% purity. ESI-MS (*m*/*z*): [M + H]^+^ calcd for C_25_H_25_F_2_N_5_O_2_S_2_, 530.15; found, 530.44.

#### *N*-(3-(5-(2-Amino-6-methylpyrimidin-4-yl)-2-(*tert*-butyl)­thiazol-4-yl)-2-fluorophenyl)-2,5-difluorobenzenesulfonamide (**11**)

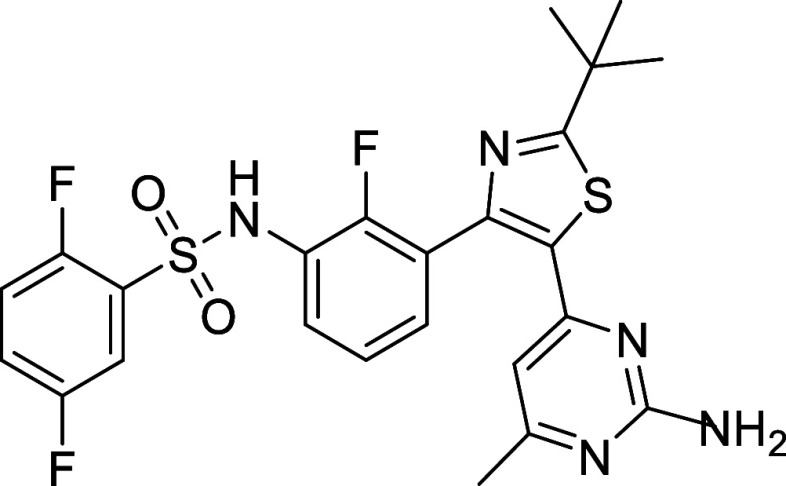



^1^H NMR (500 MHz, *CD*_3_ *OD*) δ ppm 7.49–7.58 (m, 2 H), 7.36–7.41 (m, 1 H), 7.26–7.35 (m, 3 H), 6.04 (s, 1 H), 2.12 (s, 3 H), 1.48 (s, 9 H); 70% yield; 97% purity. ESI-MS (*m*/*z*): [M + H]^+^ calcd for C_24_H_22_F_3_N_5_O_2_S_2_, 534.12; found, 534.13.

## Supplementary Material





## Notes

Inhibitor screening and modeling, crystal structure, profiling of inhibitor activity in vitro, and characterization of compounds (PDF)
Molecular formula strings (CSV)

## ‡‡

Altos Laboratories, Inc., Bay Area Institute of Science, Redwood City, CA 94065, USA

## Abbreviations

ATF4: activating transcription factor 4
BRAF: v-raf murine sarcoma viral oncogene homologue B
DCM: dichloromethane
DMA: *N*,*N*-dimethylacetamide
DTT: dithiothreitol
GCN2: general control nonderepressible 2
GST: glutathione S-transferase
HRI: Heme-regulated inhibitor
LiHMDS: lithium bis­(trimethylsilyl)­amide
MEK: dual specificity mitogen-activated protein kinase
NBS: *N*-bromosuccinimide
OICR: Ontario Institute for Cancer Research
PDB: protein data bank
PERK: protein kinase R-like endoplasmic reticulum kinase
PKR: Interferon-induced double-stranded RNA-activated protein kinase
pMEK: phosphorylated MEK
poly­(I:C): polyinosinic–polycytidylic acid
THF: tetrahydrofuran
