# Supplementary material for: Structural Transformation of a BRAF Inhibitor into a Selective PKR Inhibitor
Source: J Med Chem. 2026 Jun 22;69(13):15227–46. doi: 10.1021/acs.jmedchem.5c03664 (PMC13371904; doi:10.1021/acs.jmedchem.5c03664)
Supplement: Supplementary file 1 [file jm5c03664_si_001.pdf]

Supporting Information

Structural Transformation of a BRAF Inhibitor into a Selective PKR Inhibitor

Jay Yin,[†,‡,x] Smriti Srivastava,[§,x] Xiaojing Tang,[†] Caleb Galbraith,[†,#] Oro Uchenunu,[%] Joshua Miller,[†] Yuwei Liu,[∥,⊥] Isabella Crescenzi,[†] Taira Kiyota,[§] Igor Kurinov,[††] Mauro Costa-Mattioli,[∥,⊥] Radek Laufer,[§] Ahmed Aman,[§,§§] Robert Rottapel,[%,∃,∀] Jailall Ramnauth,[§] Diane L. Haakonsen,[†,#,] David Uehling,[§,*] and Frank Sicheri[†,‡,#,*]*

AUTHOR ADDRESS

† Lunenfeld-Tanenbaum Research Institute, Sinai Health System, Toronto, Ontario M5G 1X5, Canada

‡ Department of Biochemistry, University of Toronto, Toronto, Ontario M5S 1A8, Canada

Department of Molecular Genetics, University of Toronto, Toronto, Ontario M5S 1A8, Canada

§ Drug Discovery Program, Ontario Institute for Cancer Research, MaRS Centre, Toronto, Ontario M5G 0A3, Canada

% Princess Margaret Cancer Centre, University Health Network, Toronto, Ontario M5G 1L7, Canada

∃ Departments of Medicine, Medical Biophysics and Immunology, University of Toronto, Toronto, Ontario M5G 1L7, Canada

∀ Division of Rheumatology, St Michael's Hospital, Toronto, Ontario M5B 1W8, Canada

|| Neuroscience, Baylor College of Medicine, One Baylor Plaza, Houston, Texas 77030, United States

†† NE-CAT APS, Building 436E, Argonne National Laboratory, 9700 S. Cass Avenue, Argonne, Illinois 60439, United States

[§§] Leslie Dan Faculty of Pharmacy, University of Toronto, Toronto, Ontario M5S 3M2, Canada

Present Addresses

[⊥]Altos Labs, Inc, Bay Area Institute of Science, Redwood City, California 94065, United States

* Corresponding authors

[X] These authors contributed equally

Table of Contents:

Figure S1 ..... S4

Figure S2 ..... S5

Figure S3 ..... S6

Figure S4 ..... S7

Figure S5 ..... S8

Figure S6 ..... S9

Figure S7 ..... S10

^{1}H NMR Spectra for the Intermediates (**3a**, **3c**, **4a**, **4c** and **4e**) S11-S13

^{1}H, ^{19}F and ^{13}C NMR Spectra for the Compound **OICR-403184** (**5a**) S14-S15

^{1}H NMR Spectra for the Compounds (**5b-5e**) S16-S17

^{1}H and ^{19}F NMR Spectra for the Compounds (**5f** and **5g**) S18-S19

^{1}H NMR Spectra for the Intermediates (**7** and **10**) S20

^{1}H NMR Spectra for the Compounds (**9a-9g** and **11**) S21-S24

HRMS Spectra for the Compound **OICR-403184** (**5a**) S25

LCMS Spectra for the Compounds (**5a-5g**, **9a-9g** and **11**) S26-S40

Table S1 ..... S41-S50

Table S2 ..... S51

Table S3 ..... S52-S55

Table S4 ..... S56

Additional Boltz2 information S57-S58

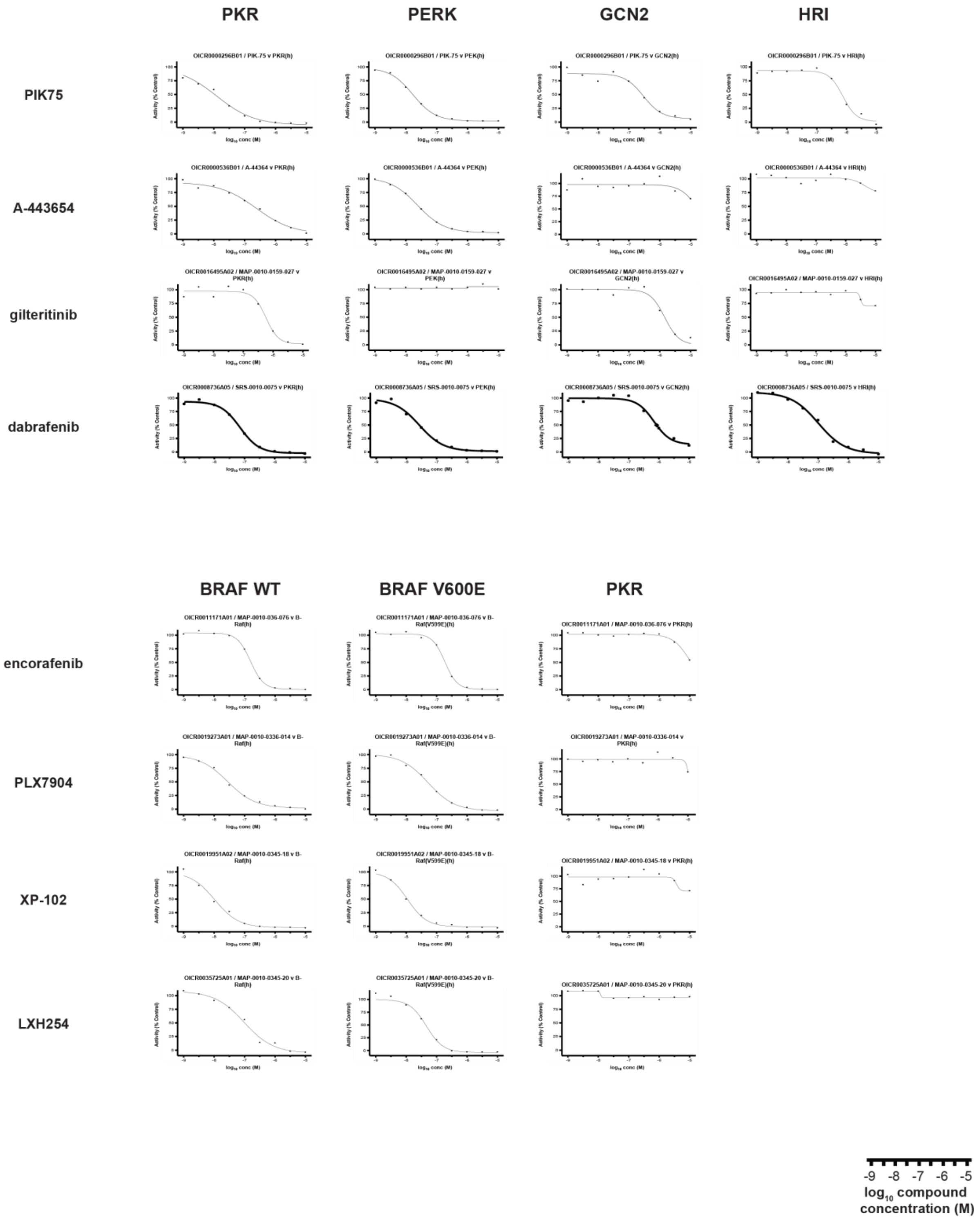

Figure S1. Inhibitory profiles of PIK75, A443654, gilteritinib, dabrafenib, and other BRAF inhibitors carried out against the indicated kinases at Eurofins, [ATP] = K_m (associated with **Table 1**).

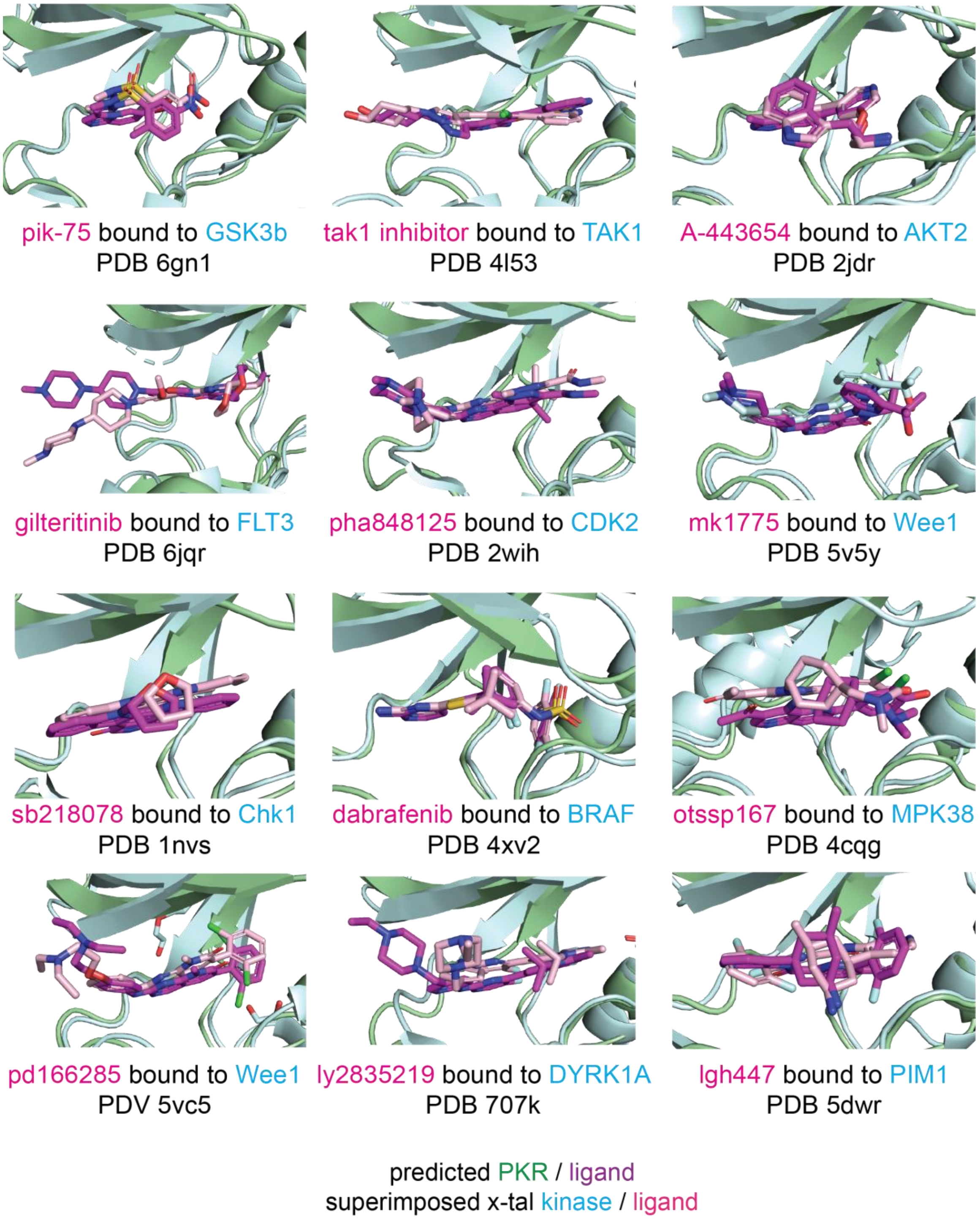

Figure S2. Comparison of inhibitor binding modes modeled by Boltz2 to PKR with experimentally determined structures of same inhibitor bound to the indicated kinases for top candidate molecules based on predicted affinity probability binary and affinity pred value better than 0.75 and -1.25 respectively (associated with **Figure 1**).

PKR

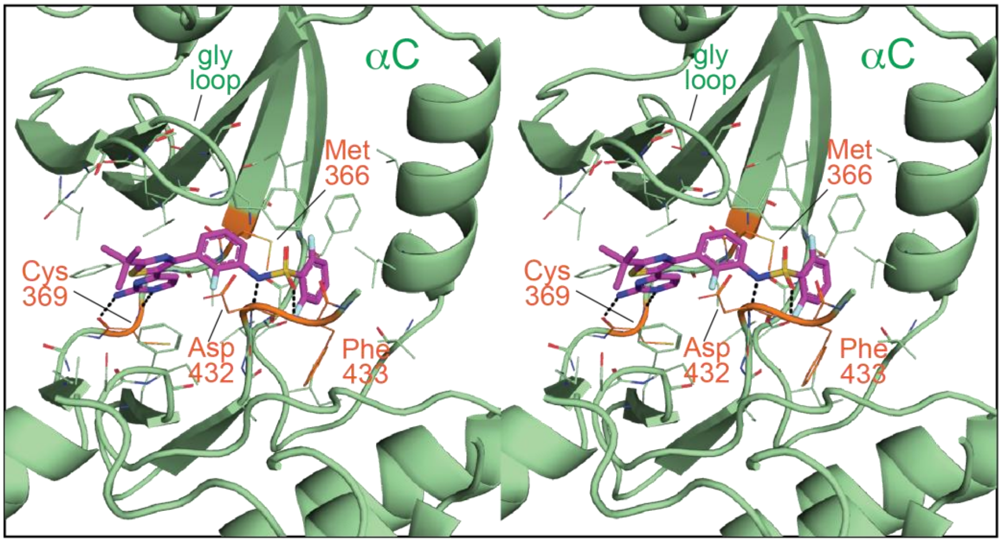

BRAF

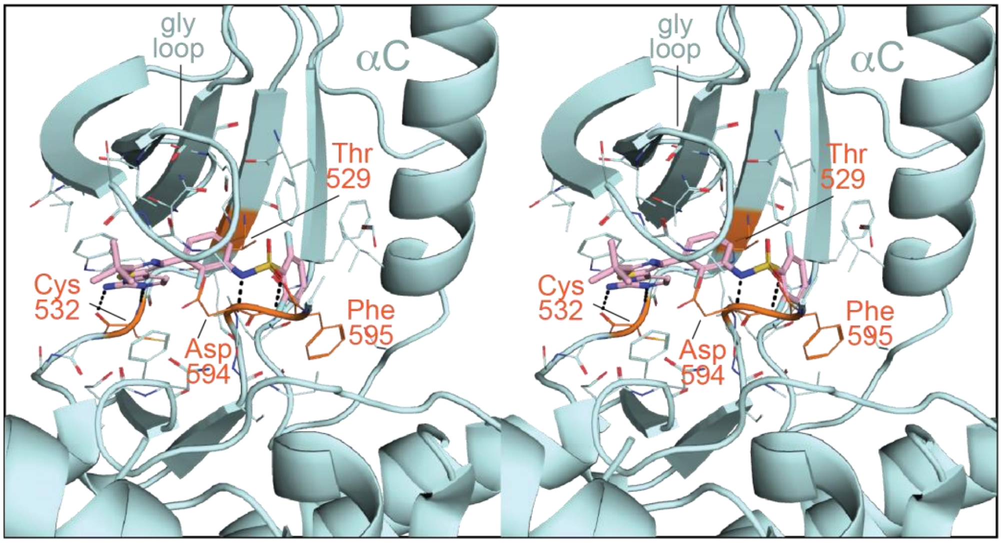

Figure S3. Wall-eye stereo views of dabrafenib bound to PKR (top) and BRAF (bottom) (PDB 4XV2). Sidechains of residues within 7 Å of dabrafenib are shown. Key residues are labeled (associated with **Figure 2**).

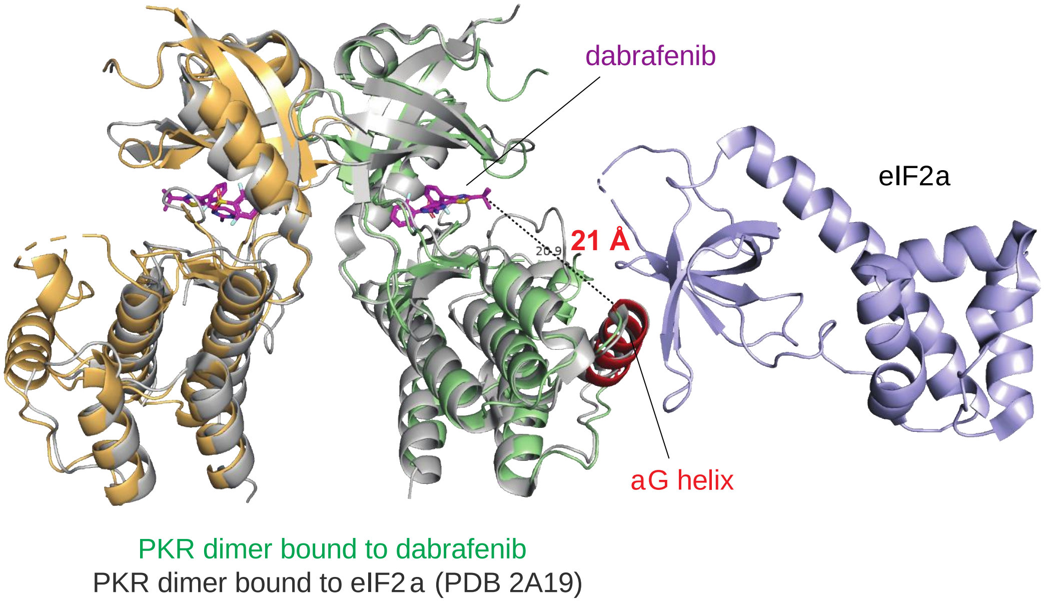

Figure S4. Dabrafenib binds to the active site of PKR. PKR kinase domain in complex with dabrafenib adopts a back-to-back dimer configuration similar to the PKR kinase domain dimer configuration when bound to ATP and its substrate eIF2α (PDB 2A19), RMSD = 1.7 Å. The PKR dimer in complex with dabrafenib is generated by crystallographic symmetry. The distance between dabrafenib and the α-G helix of PKR (red) on which eIF2α binds is highlighted.

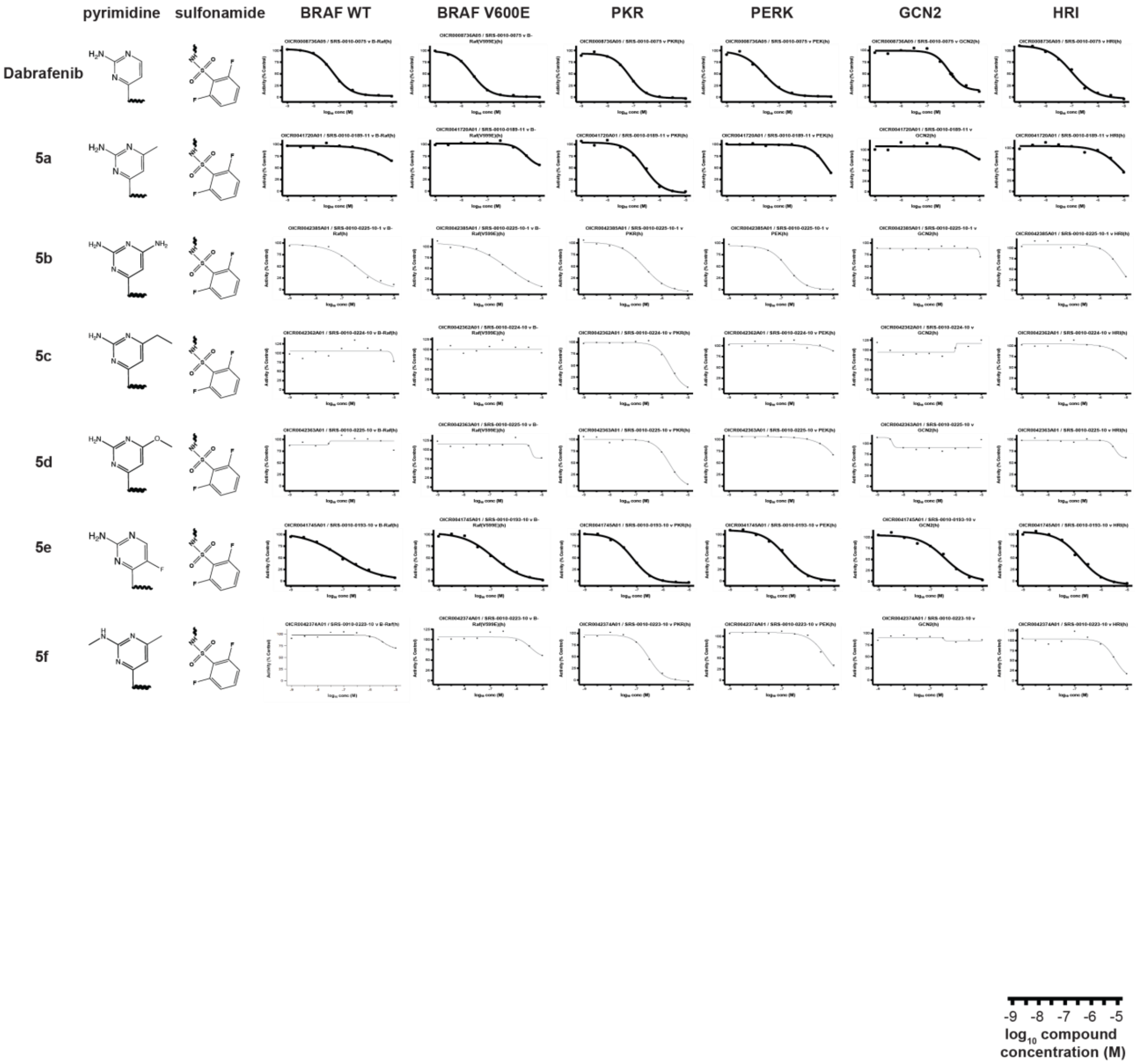

Figure S5. *In vitro* inhibitory profiles of dabrafenib pyrimidine analogs against the indicated kinases carried out at Eurofins. Chemical structure of the pyrimidine and sulfonamide groups are shown, [ATP] = K_m (associated with **Table 3**).

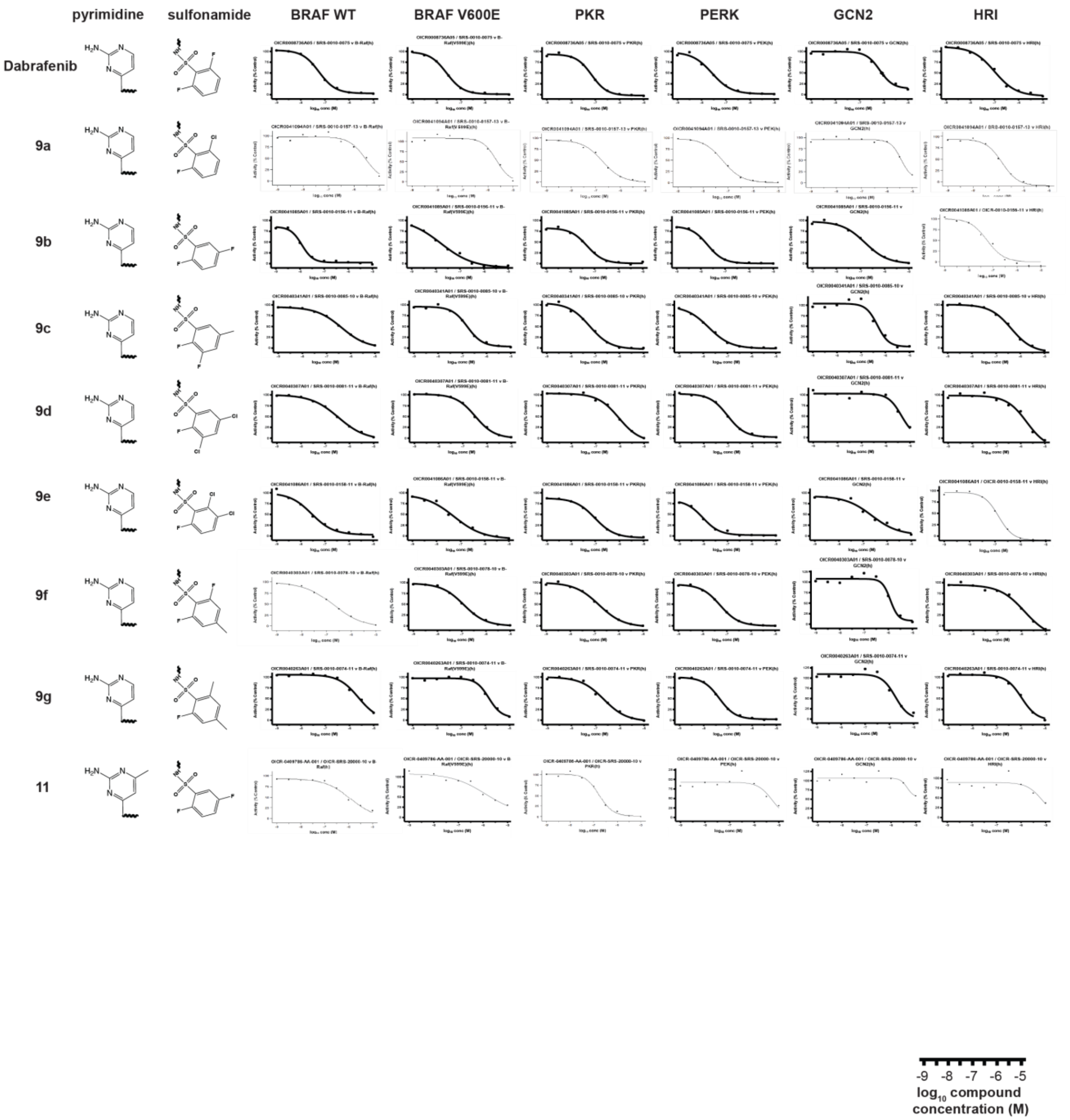

Figure S6. *In vitro* inhibitory profiles of dabrafenib sulfonamide and combination analogs against the indicated kinases carried out at Eurofins. Chemical structure of the pyrimidine and sulfonamide groups are shown, [ATP] = K_m (associated with **Table 4**).

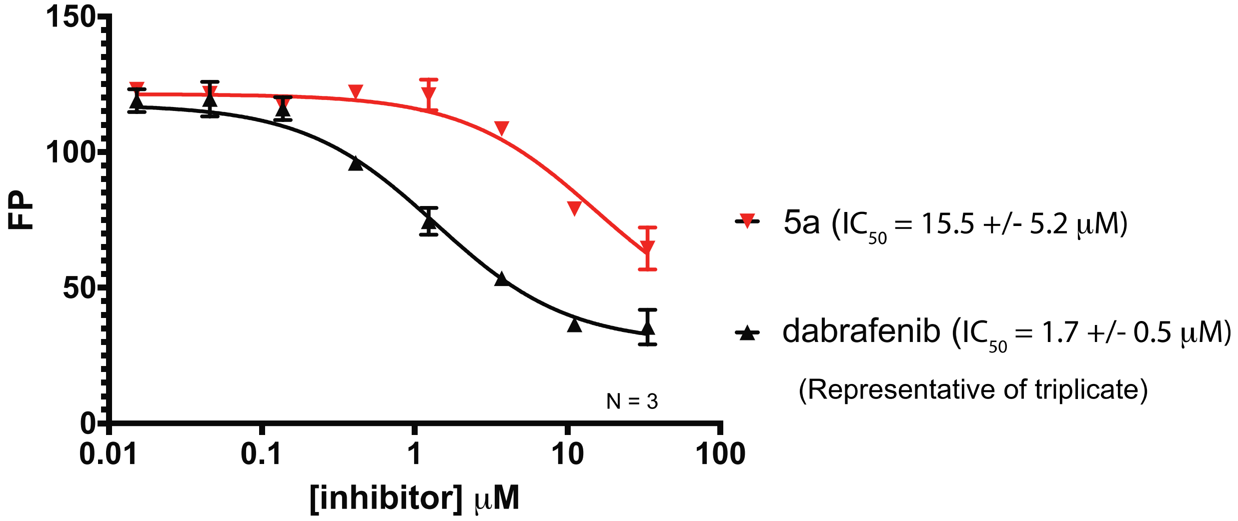

Figure S7. Dabrafenib and compound **5a** competitively displace the kinase tracer 236 (Thermo Scientific, Cat# PV5592) from the kinase active site of PKR. Error bars indicate standard deviation (N = 3). Average IC 50 values +/- standard deviation of 3 independent replicates are reported.

^{1}H NMR spectra for Compound **3a**

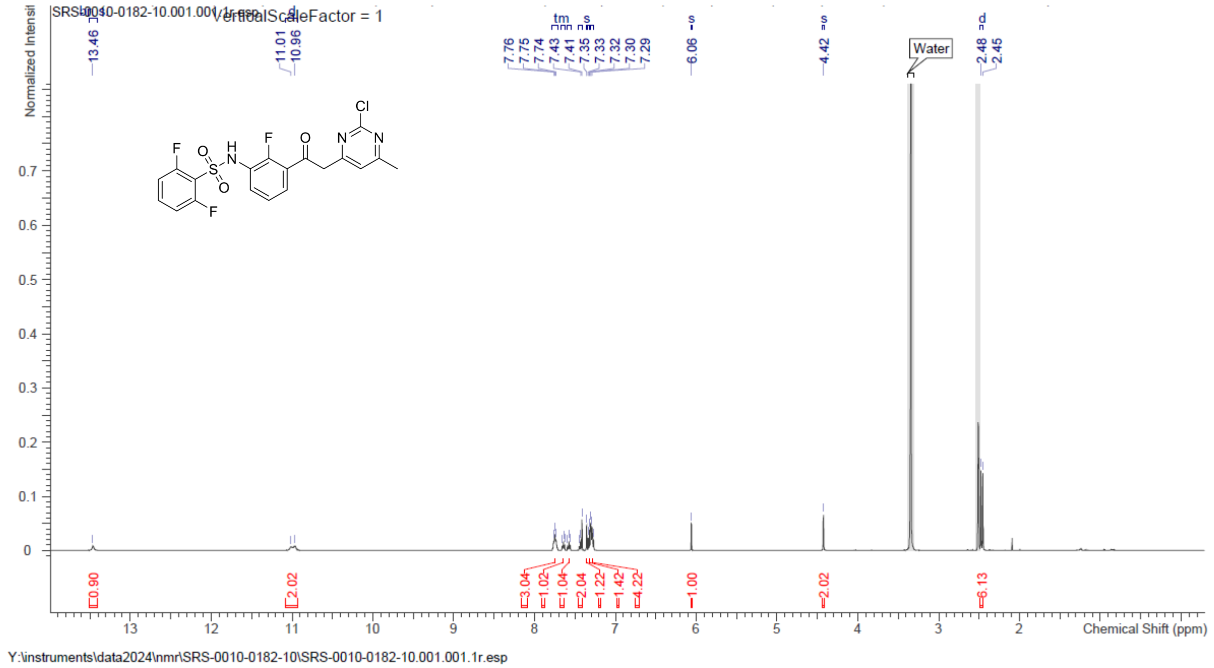

^{1}H NMR spectra for compound **3c**

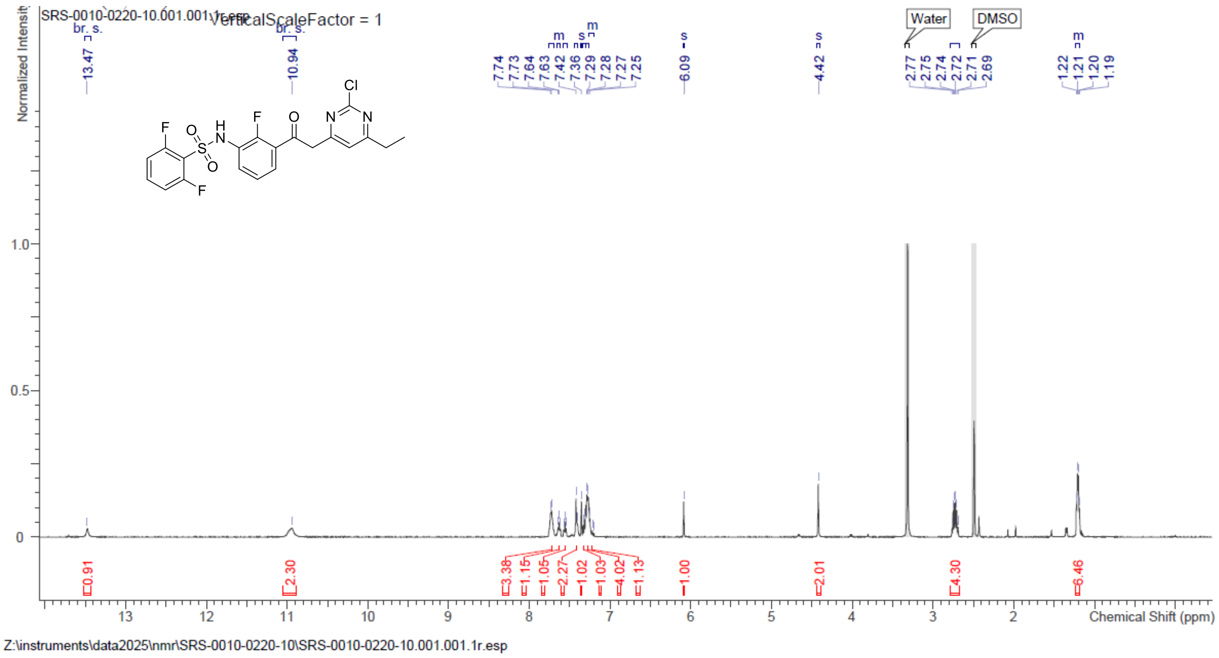

^{1}H NMR spectra for compound **4a**

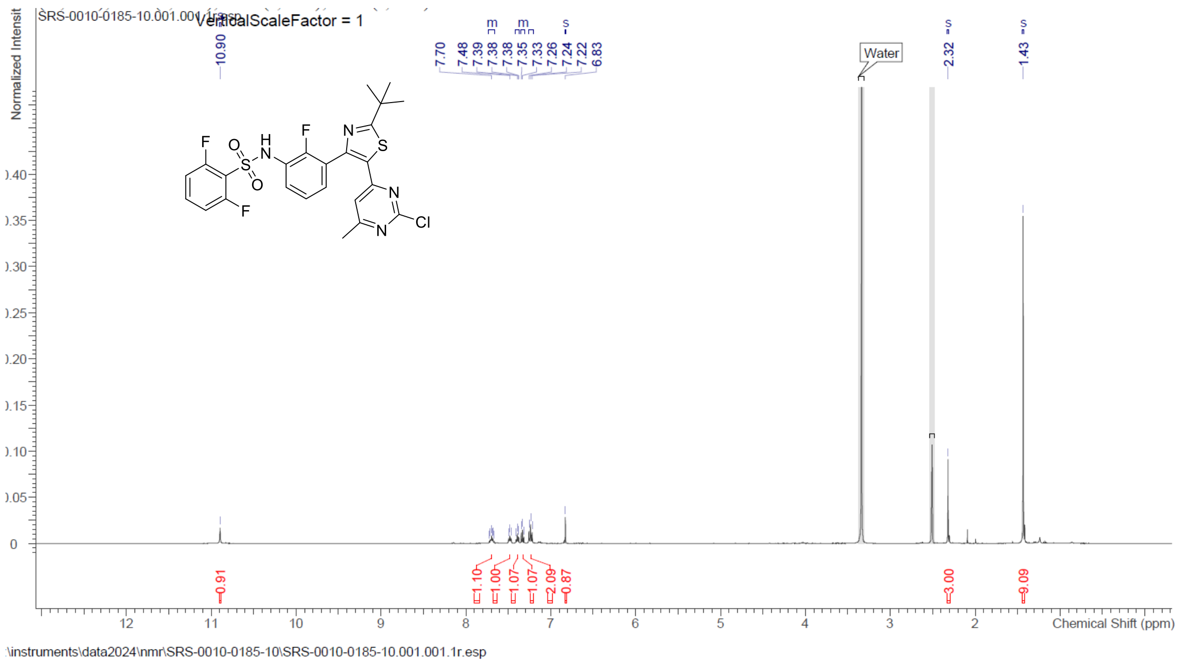

^{1}H NMR spectra for compound **4c**

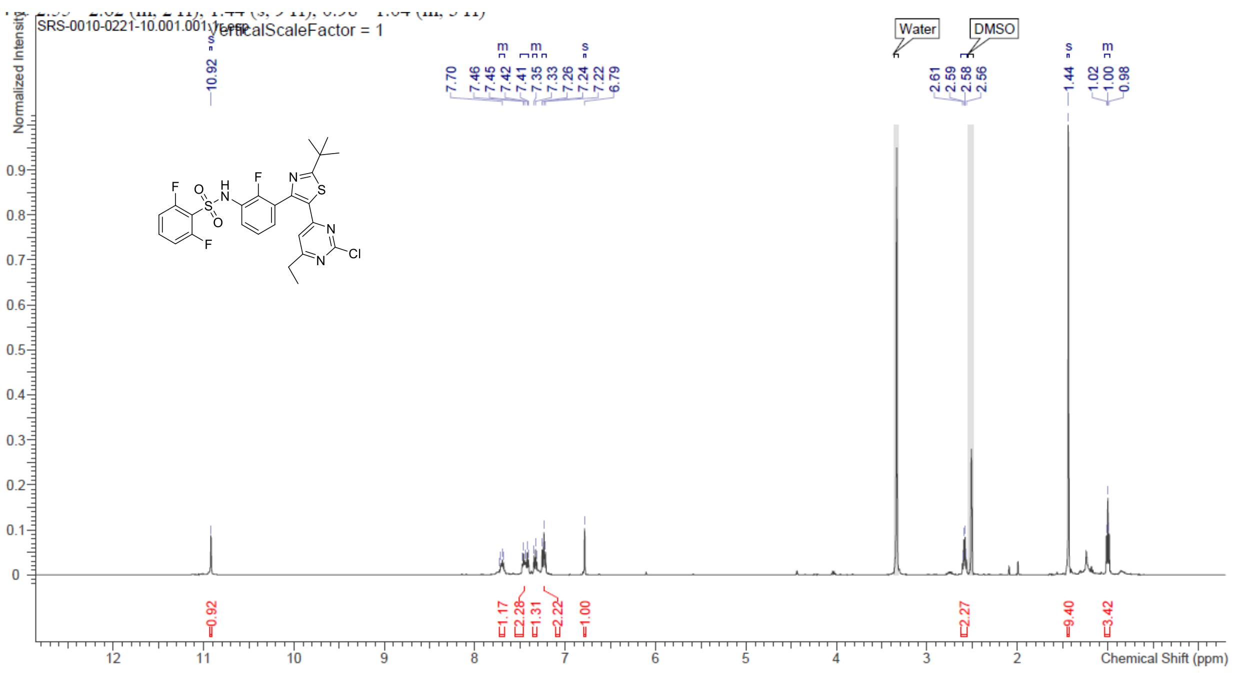

^{1}H NMR spectra for compound **4e**

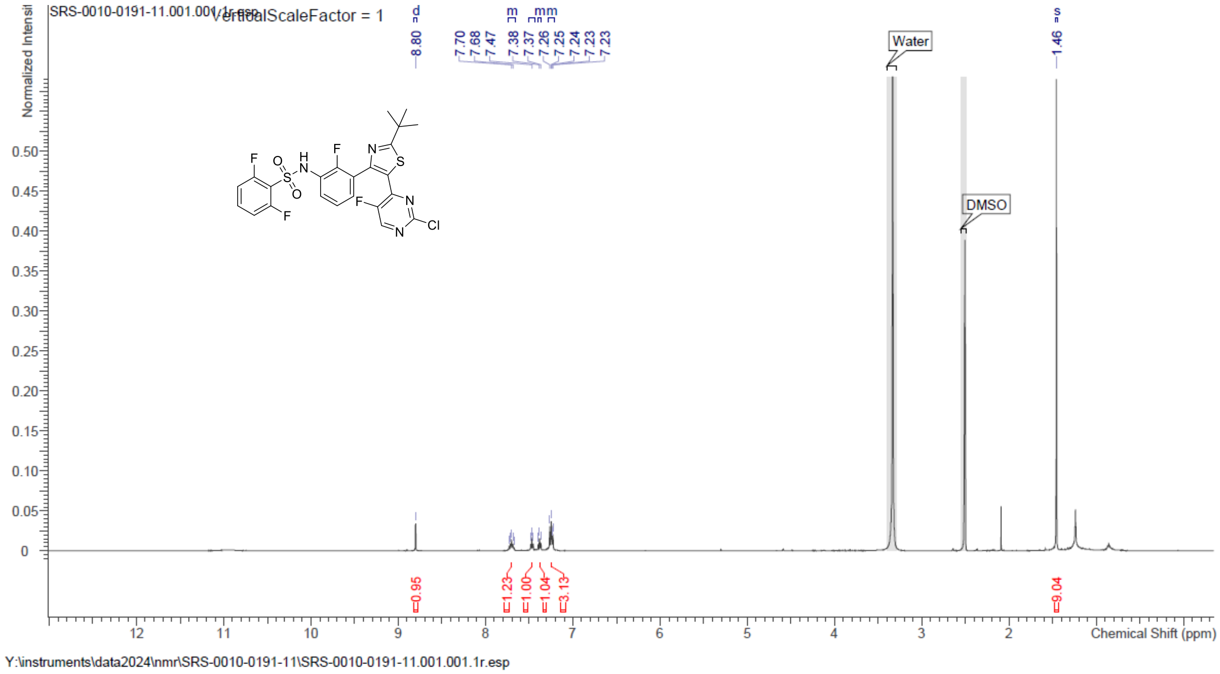

^{1}H NMR spectra for compound **5a** (**OICR-403184**)

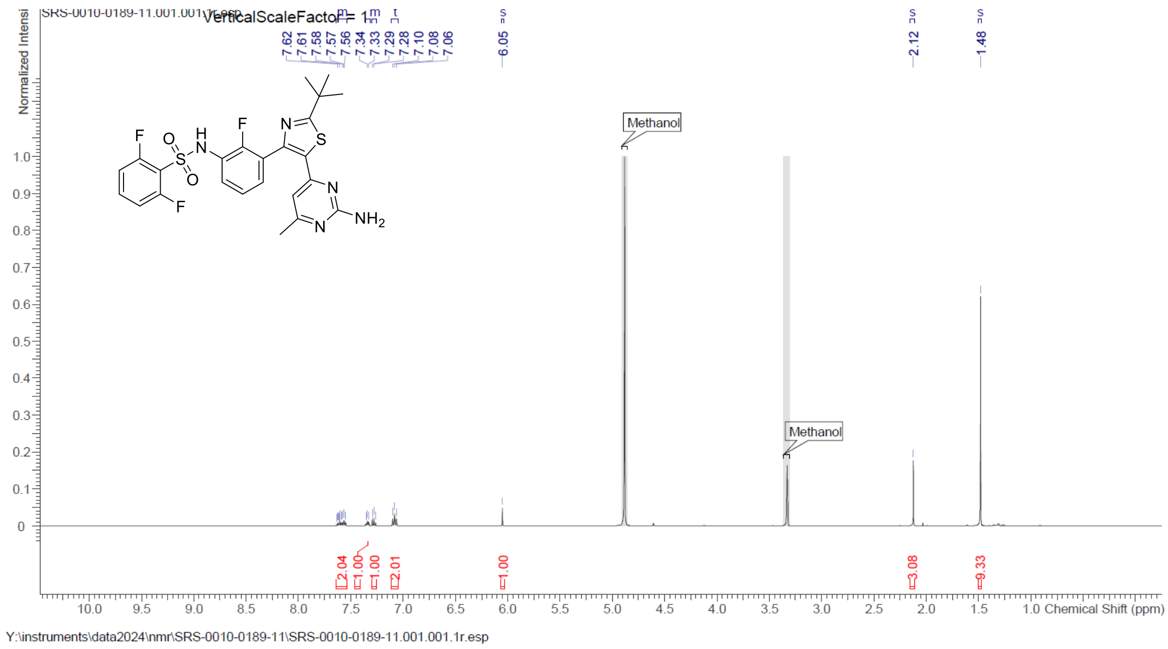

^{19}F NMR spectra for compound **5a** (**OICR-403184**)

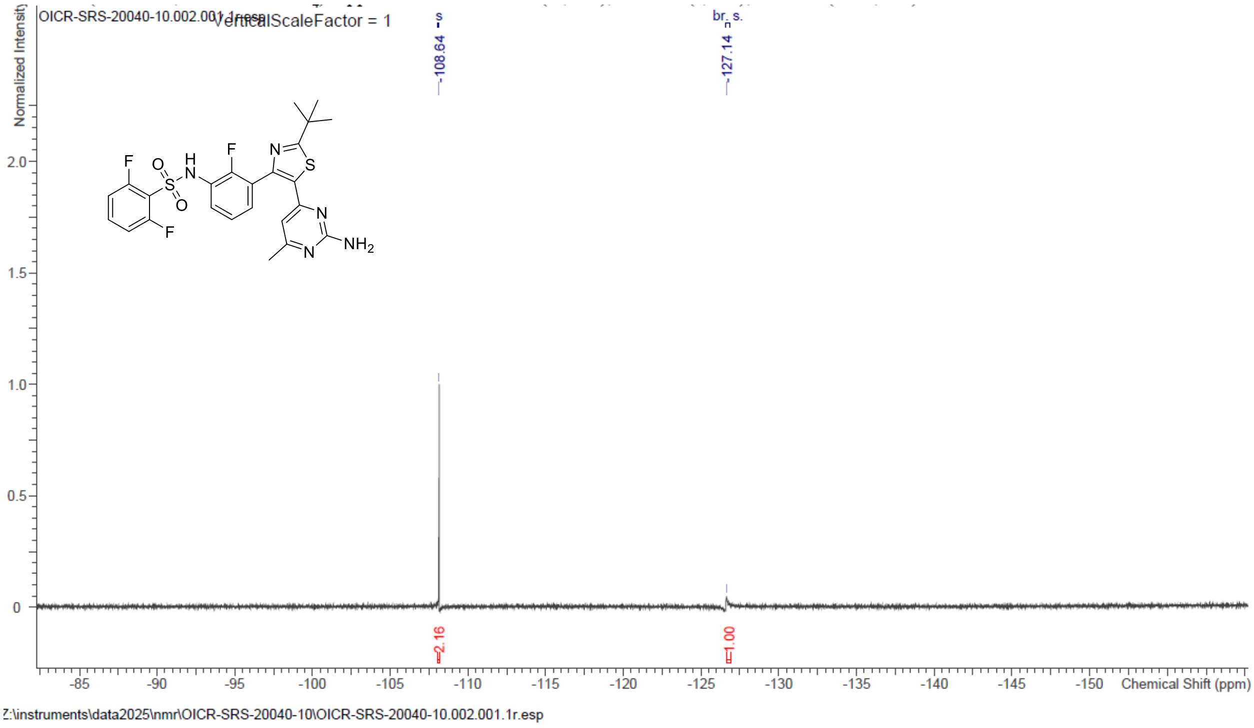

^{13}C NMR spectra for compound **5a** (**OICR-403184**)

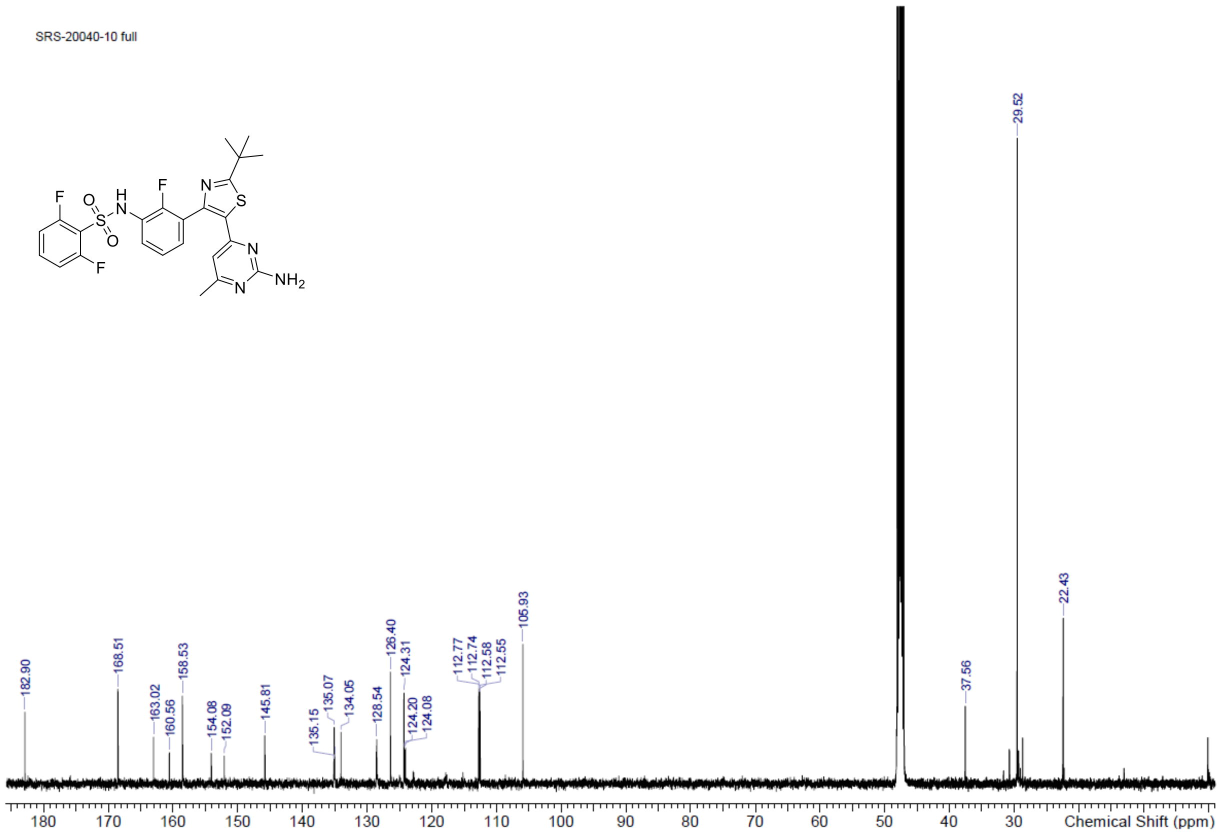

^{1}H NMR spectra for compound **5b**

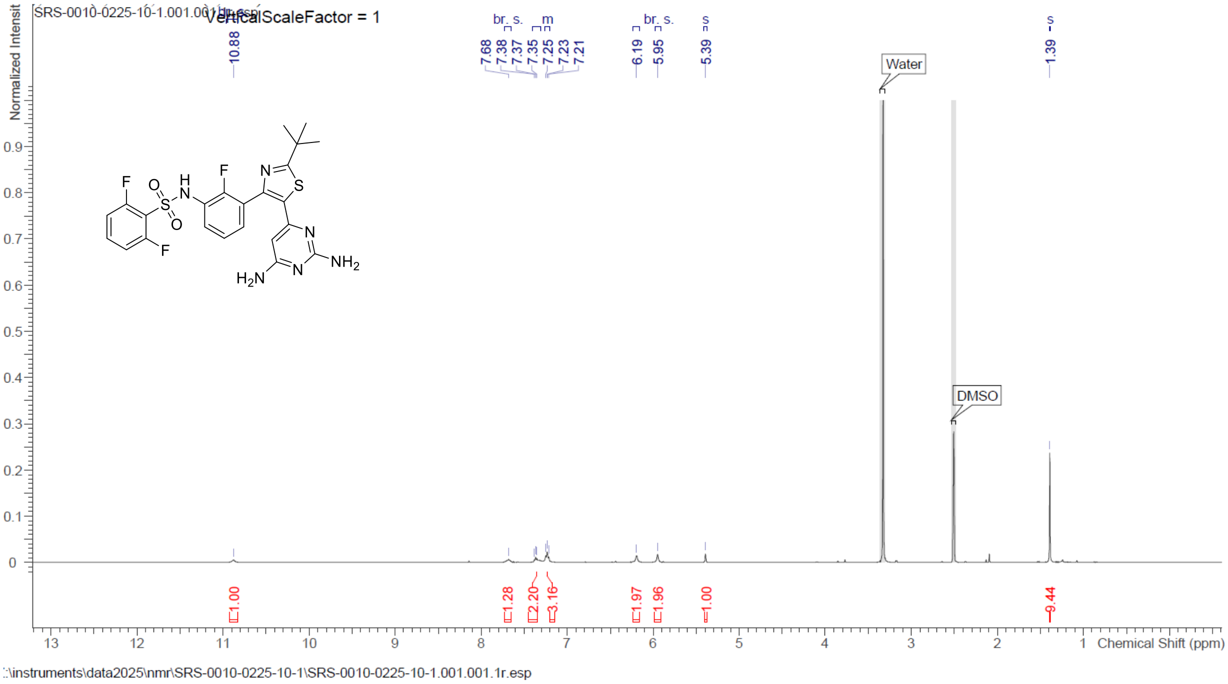

^{1}H NMR spectra for compound **5c**

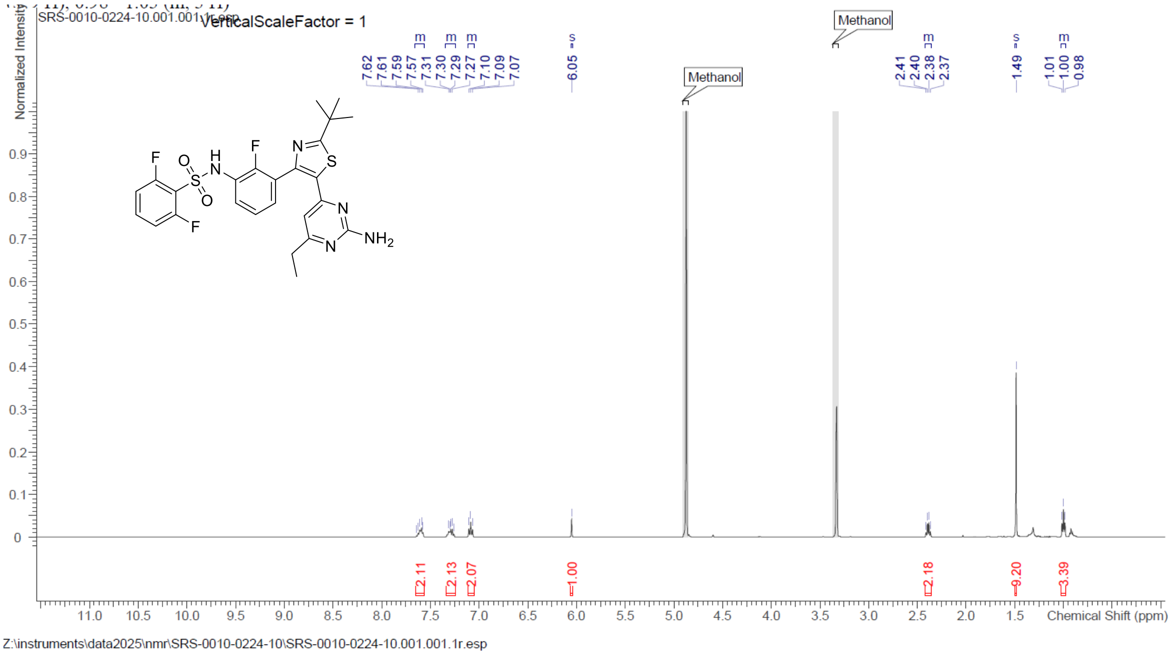

^{1}H NMR spectra for compound **5d**

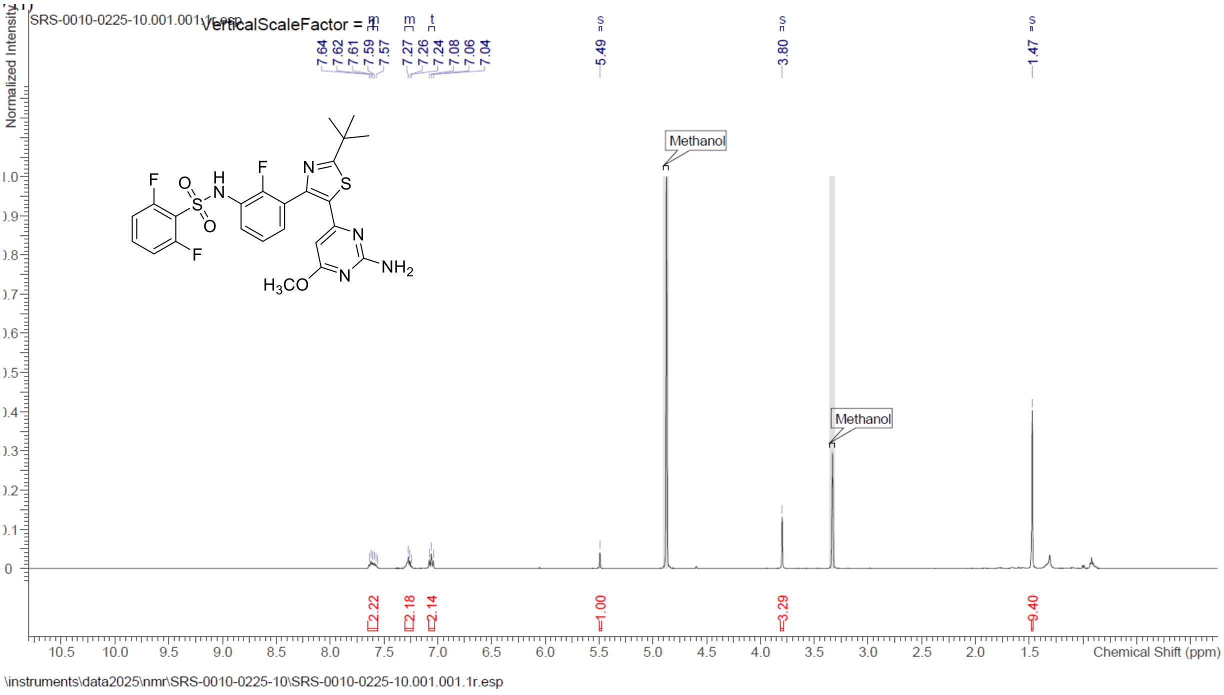

^{1}H NMR spectra for compound **5e**

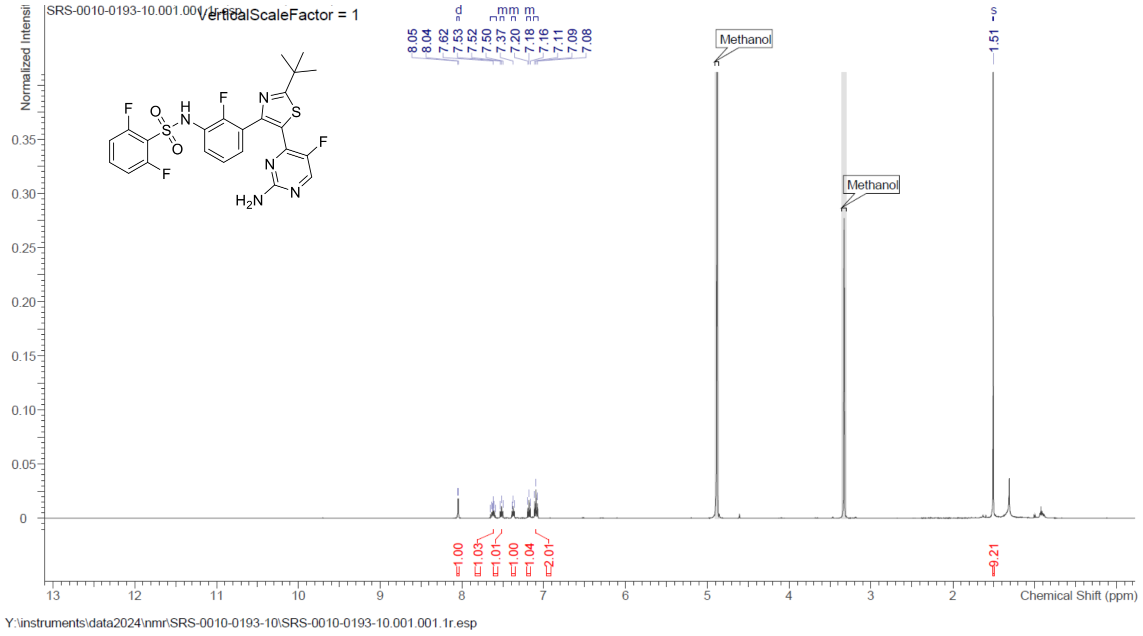

^{1}H NMR spectra for compound **5f**

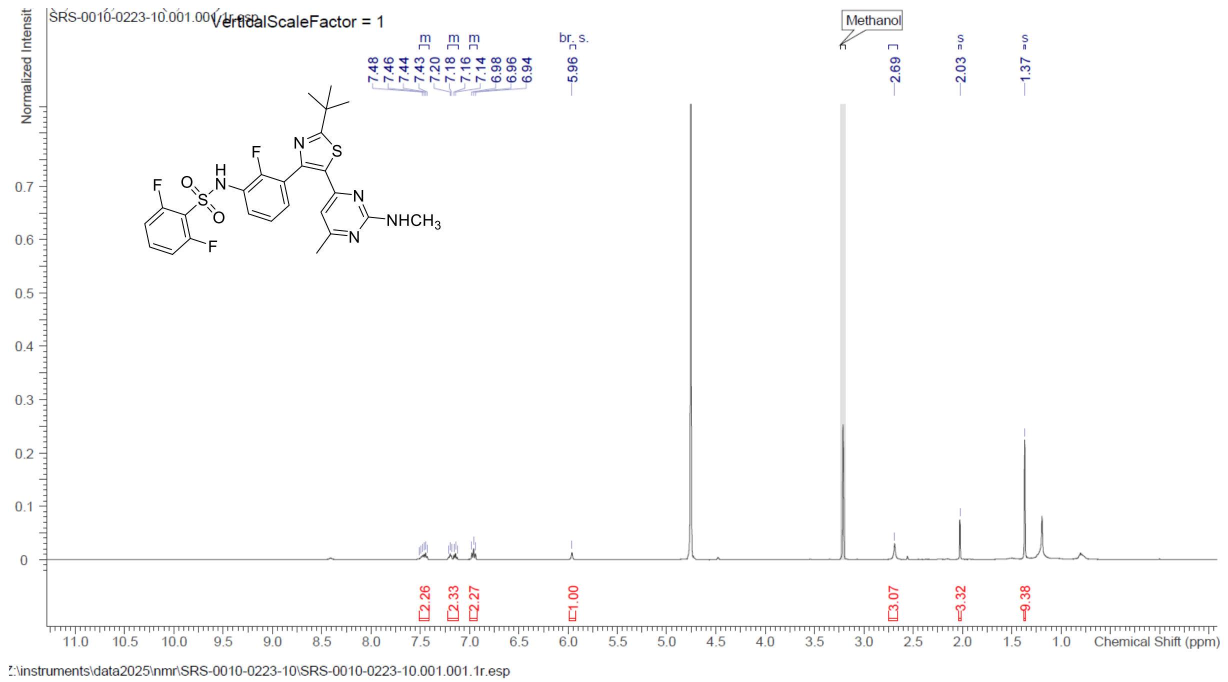

^{19}F NMR spectra for compound **5f**

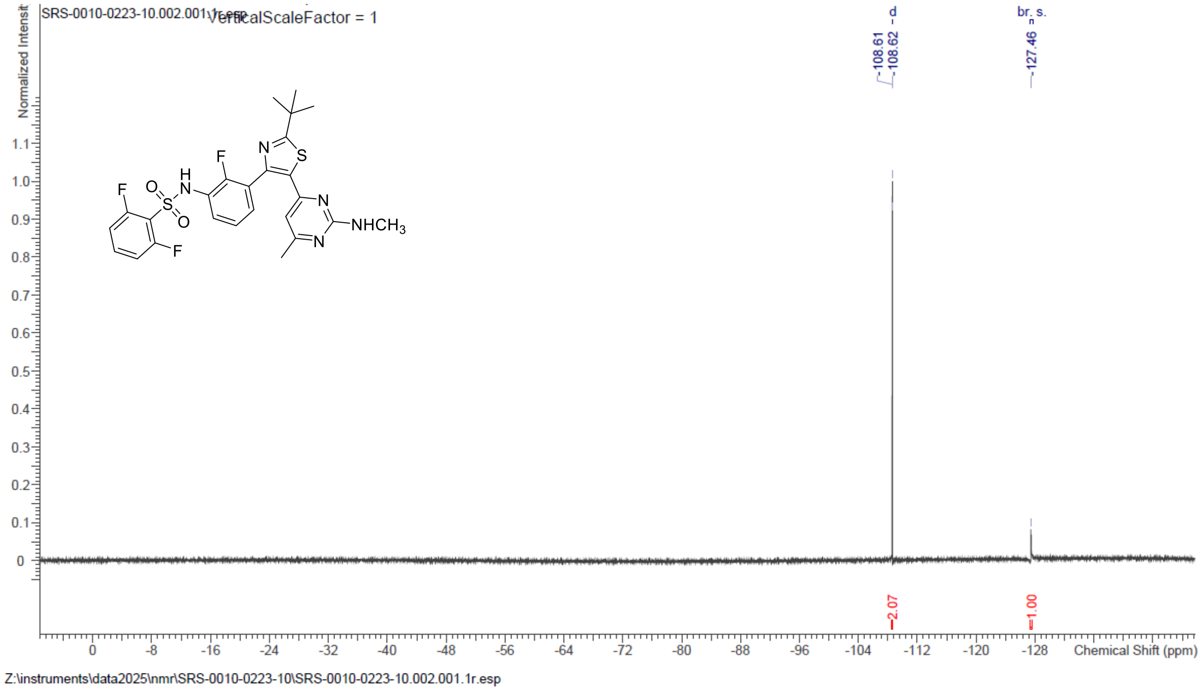

^{1}H NMR spectra for compound **5g**

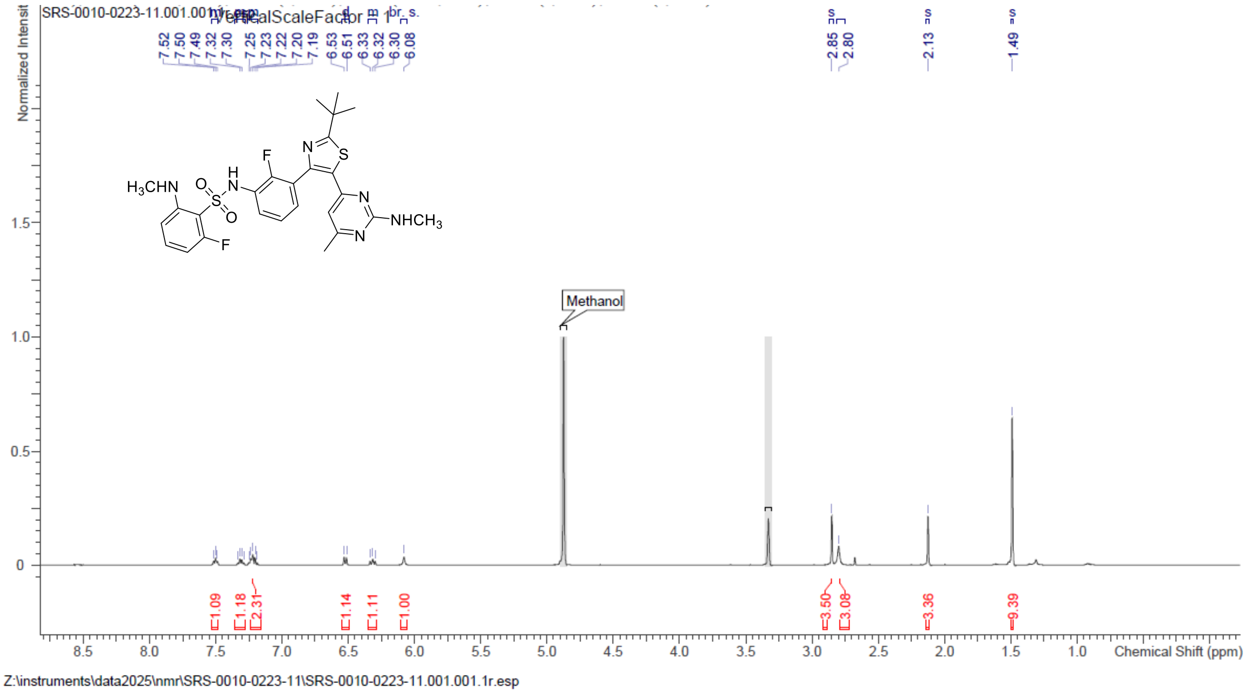

^{19}F NMR spectra for compound **5g**

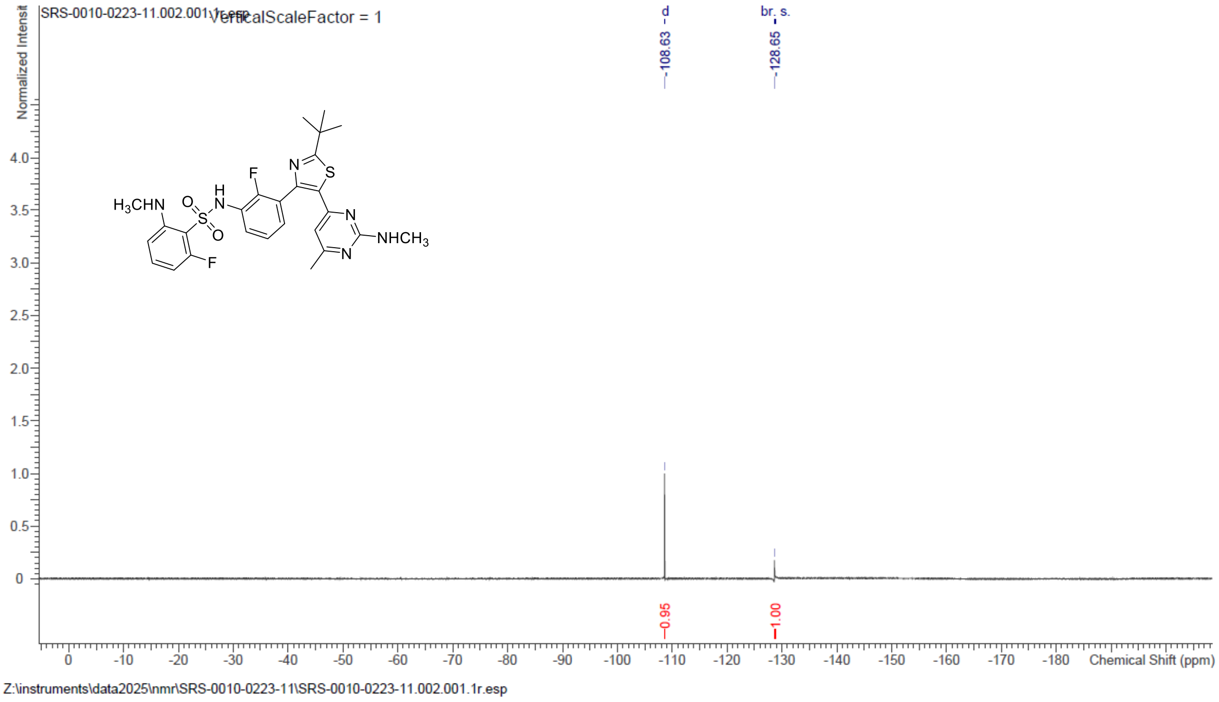

^{1}H NMR spectra for compound **7**

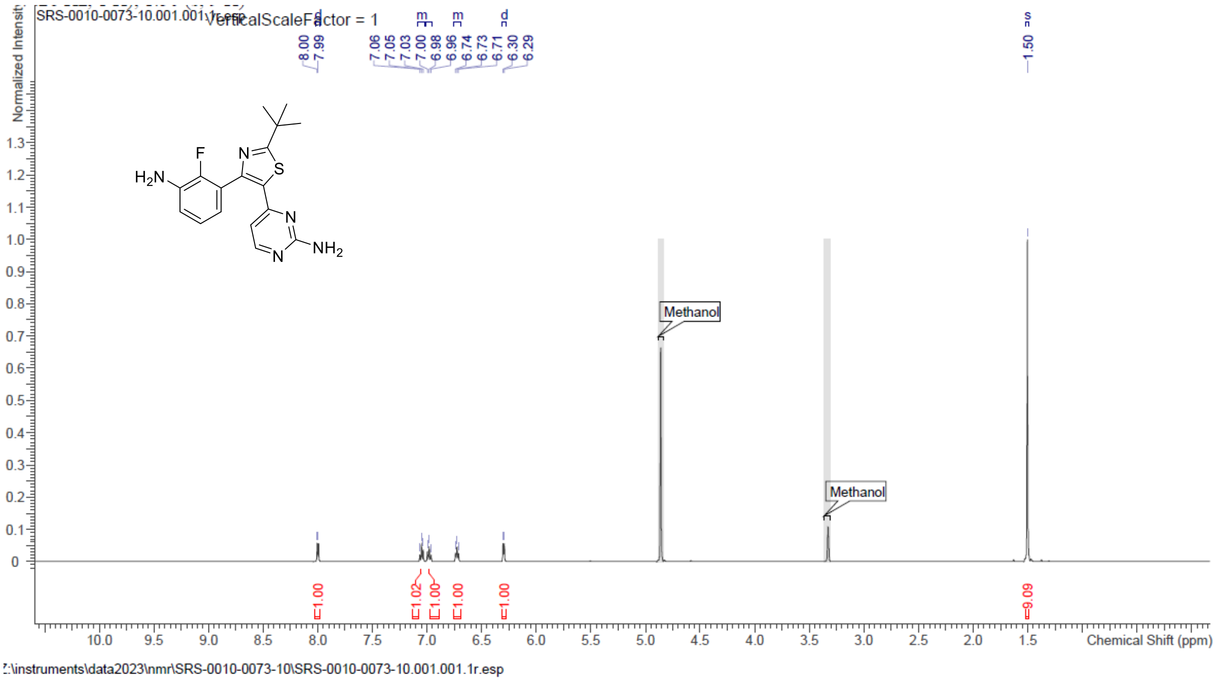

^{1}H NMR spectra for compound **10**

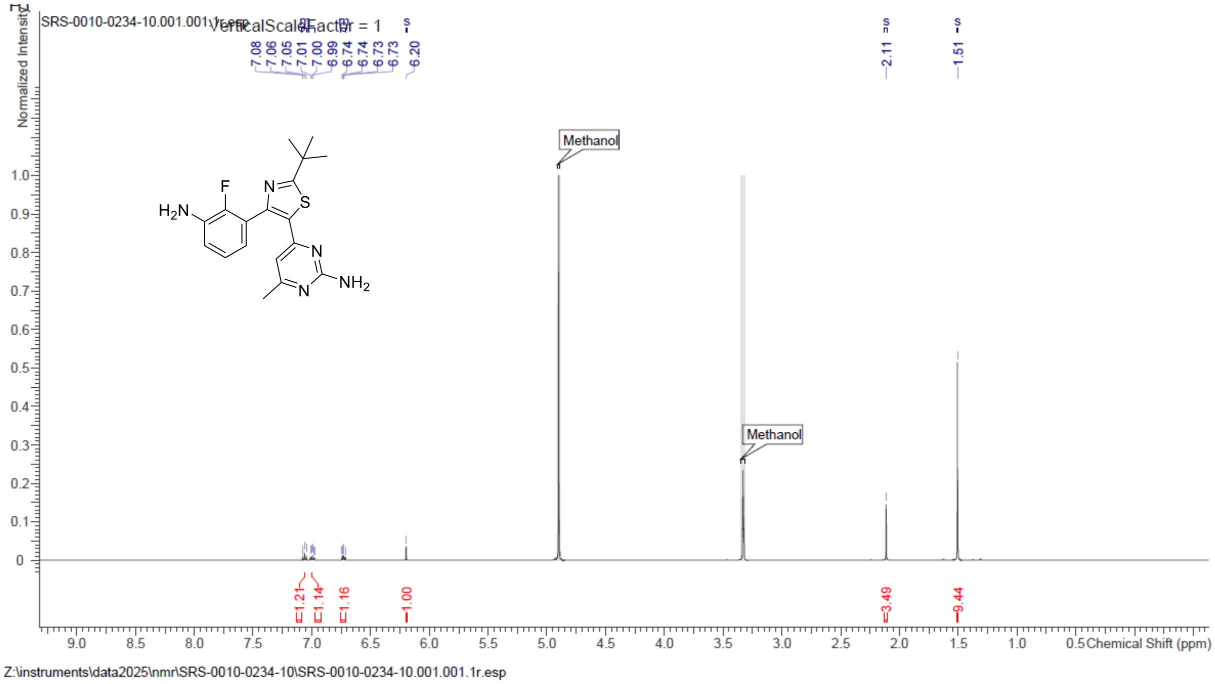

^{1}H NMR spectra for compound **9a**

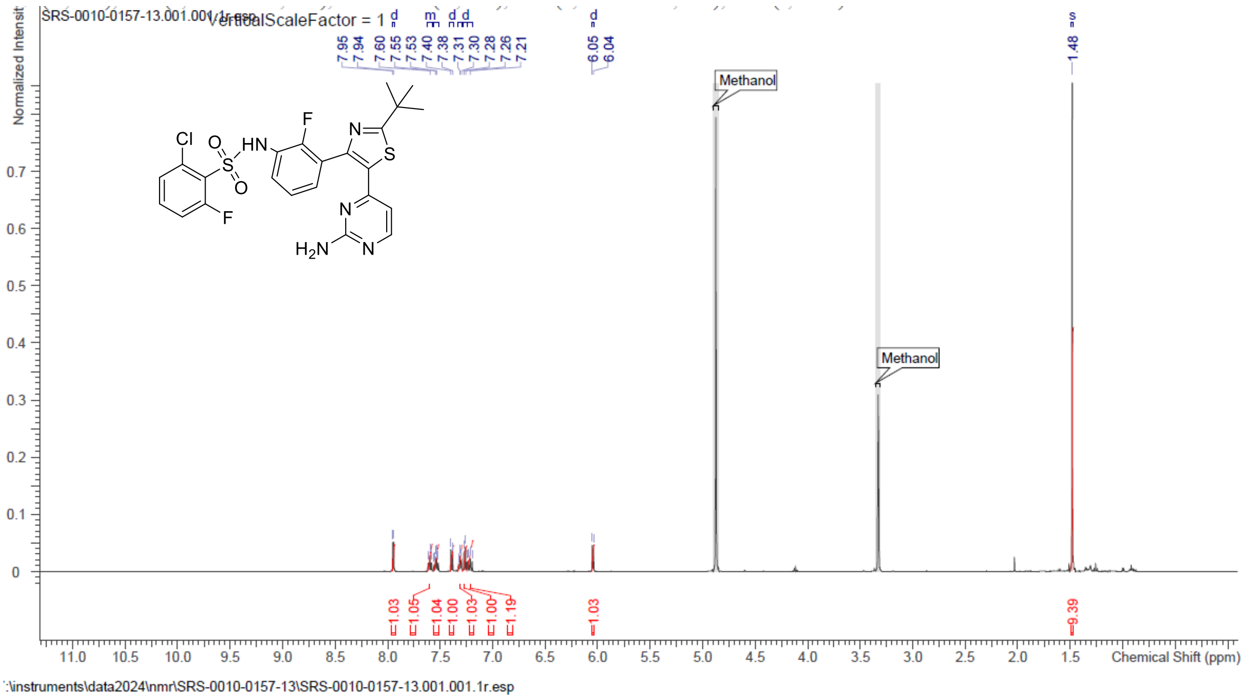

^{1}H NMR spectra for compound **9b**

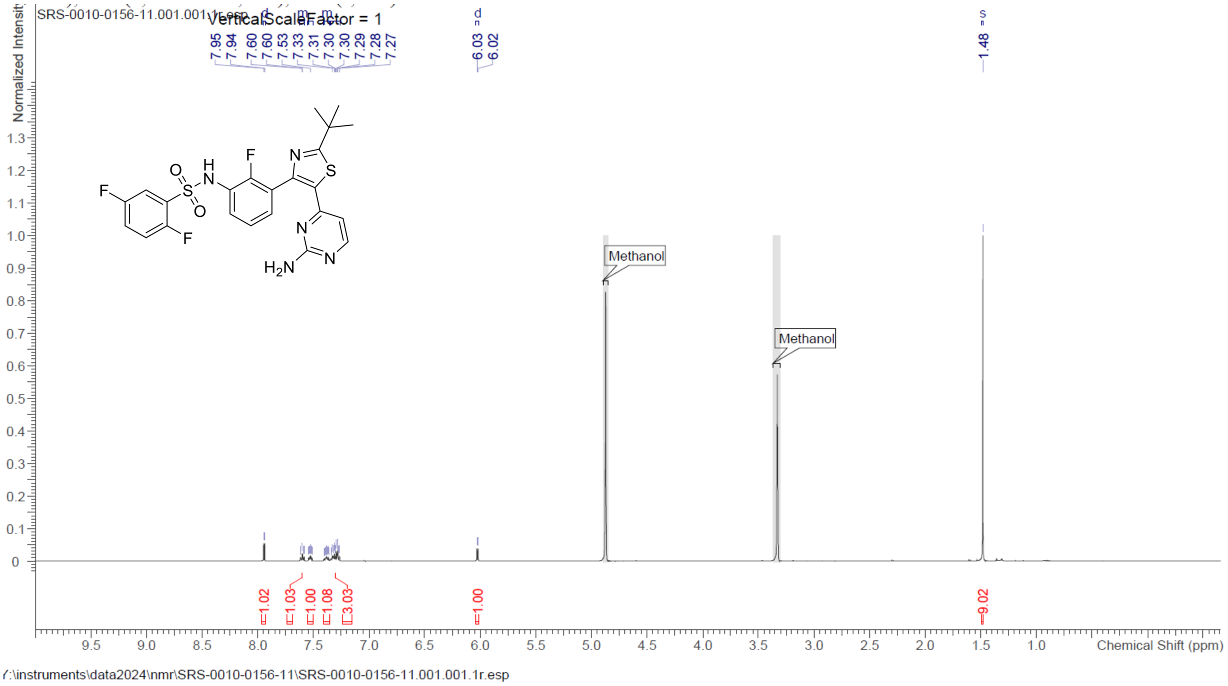

^{1}H NMR spectra for compound **9c**

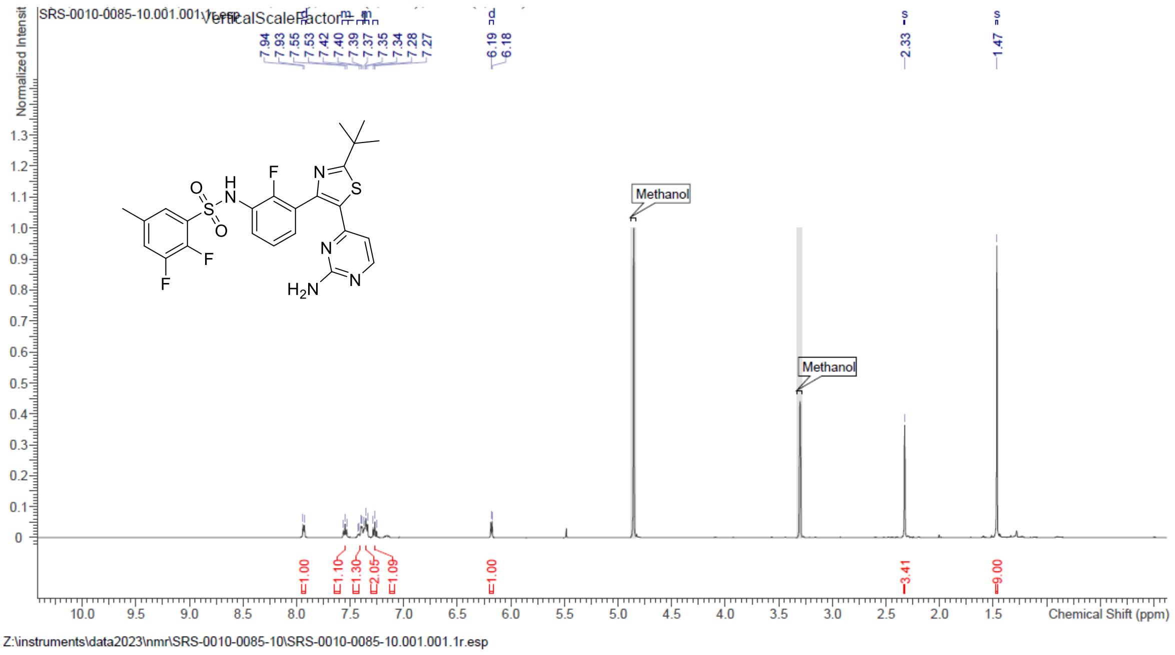

^{1}H NMR spectra for compound **9d**

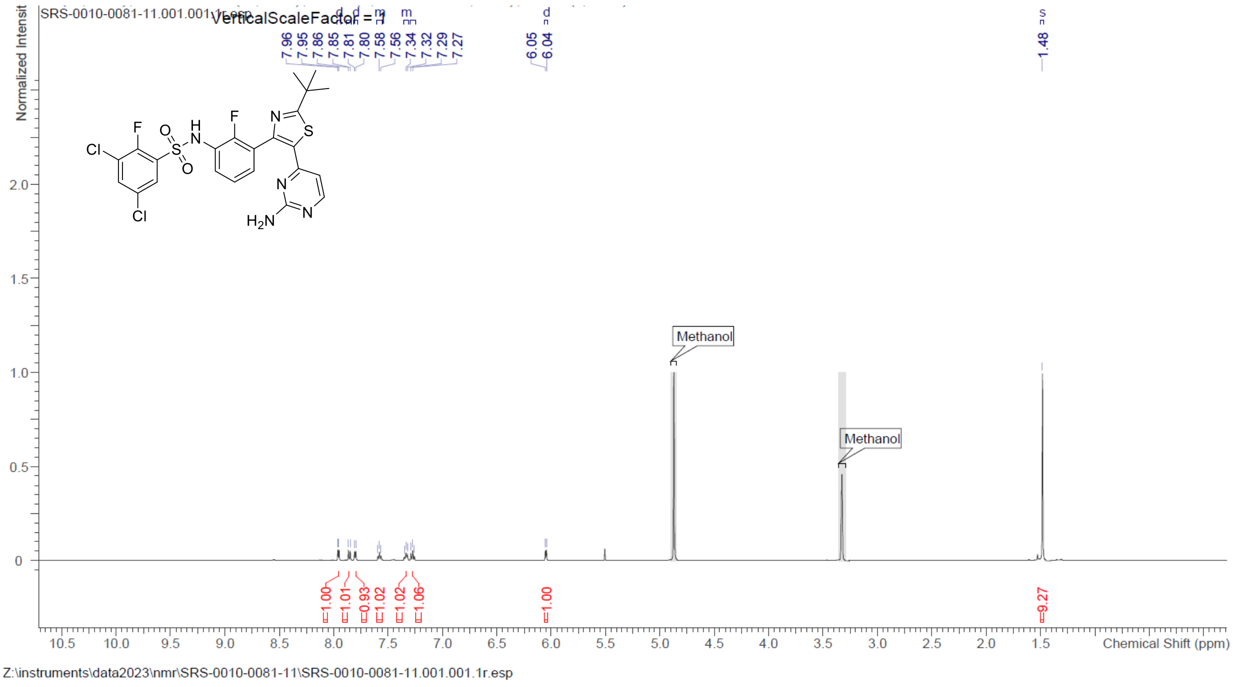

^{1}H NMR spectra for compound **9e**

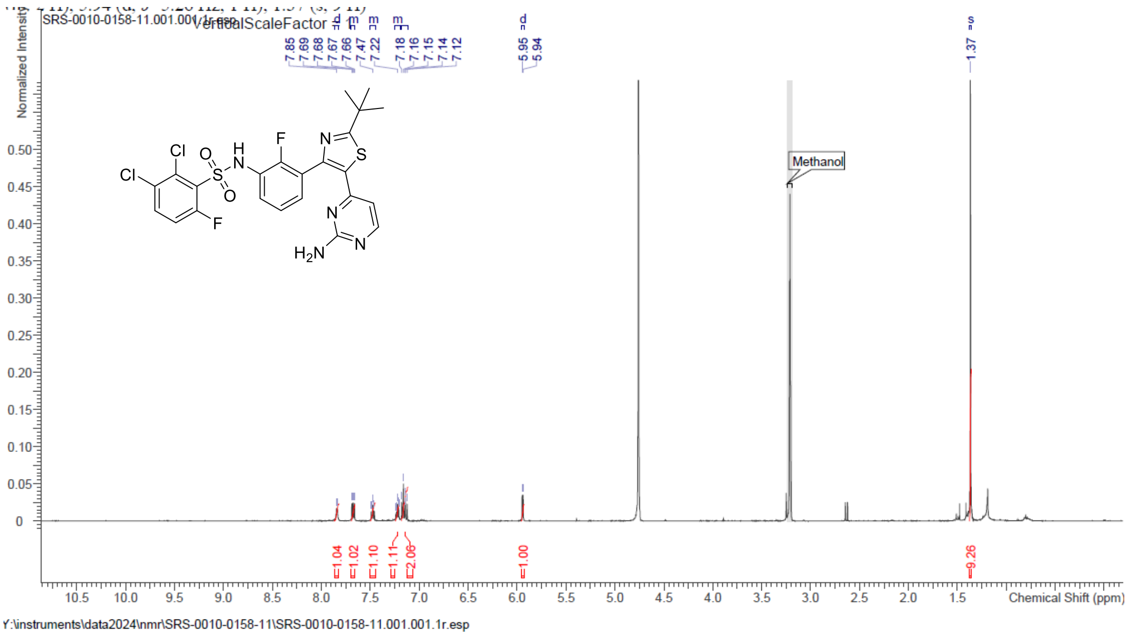

^{1}H NMR spectra for compound **9f**

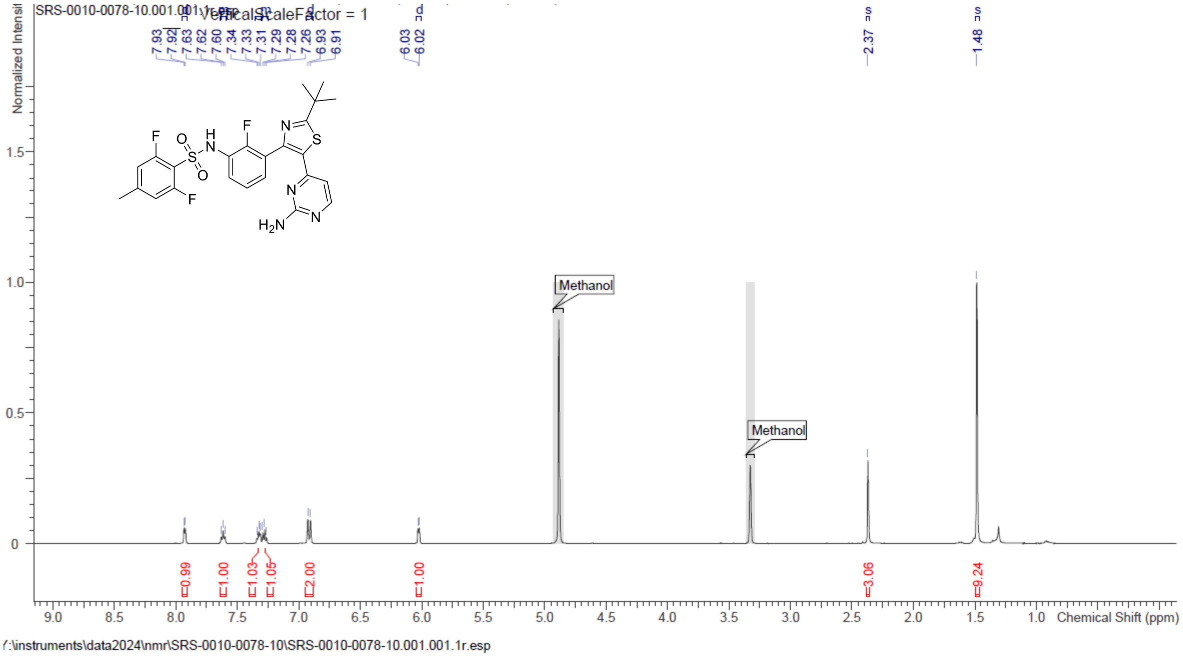

^{1}H NMR spectra for compound **9g**

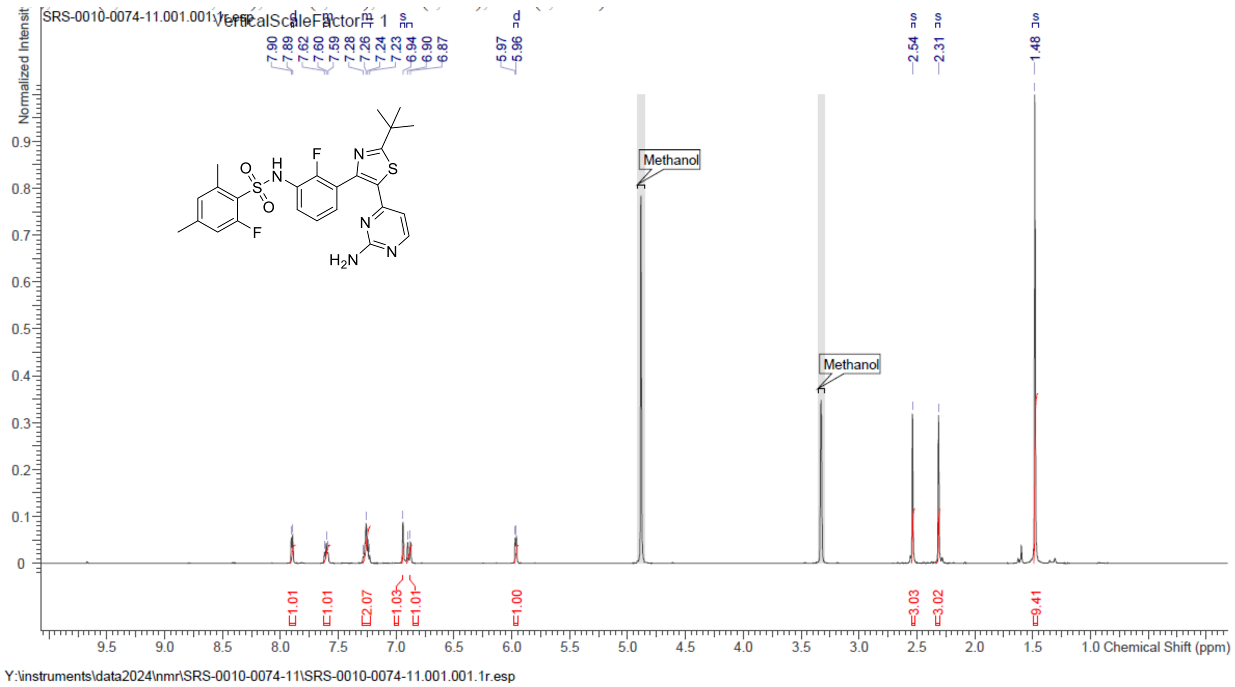

^{1}H NMR spectra for compound **11**

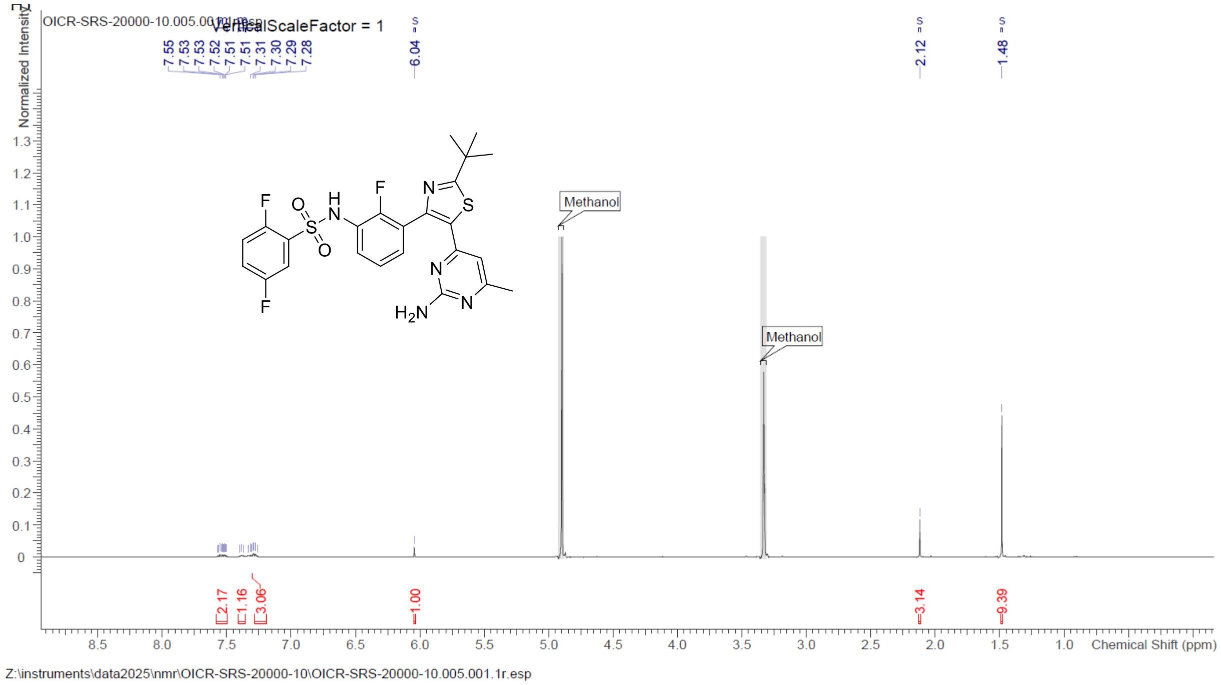

HRMS spectra for compound **5a** (**OICR-403184**)

OICR ID	Chemical Formula	Theoretical $[M+H]^+$	Observed $[M+H]^+$	Δmass (Da)	Error (ppm)
OICR-0403184-AA-002 (OICR-SRS-20040-10)	C24H22F3N5O2S2	534.1240	534.1235	-0.0005	-0.9

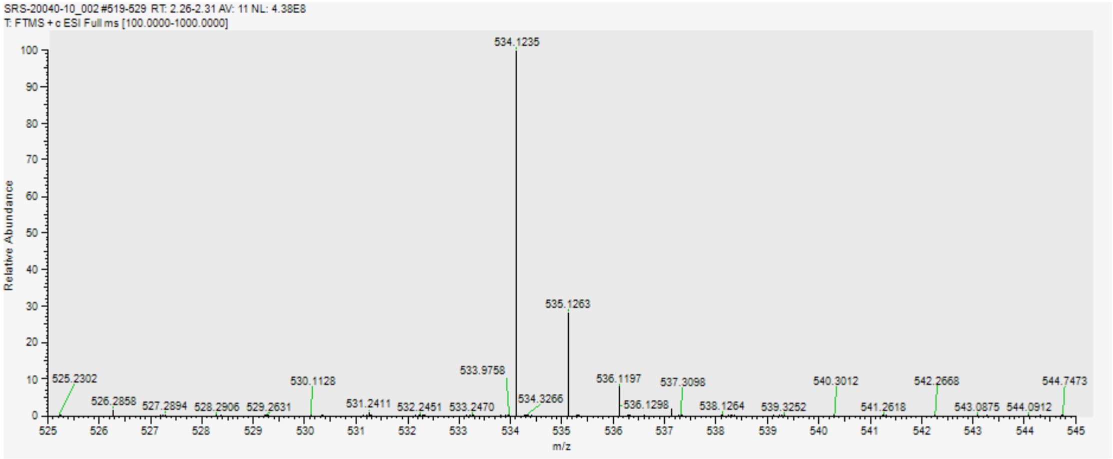

LCMS spectra for compound **5a** (**OICR-403184**)

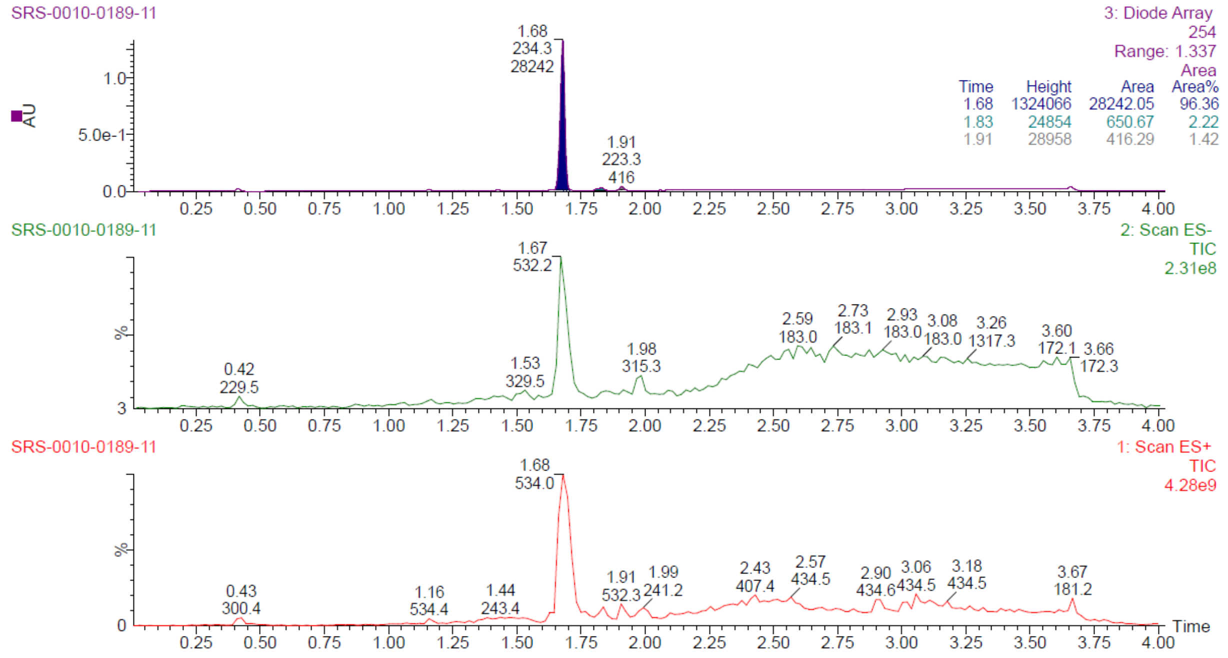

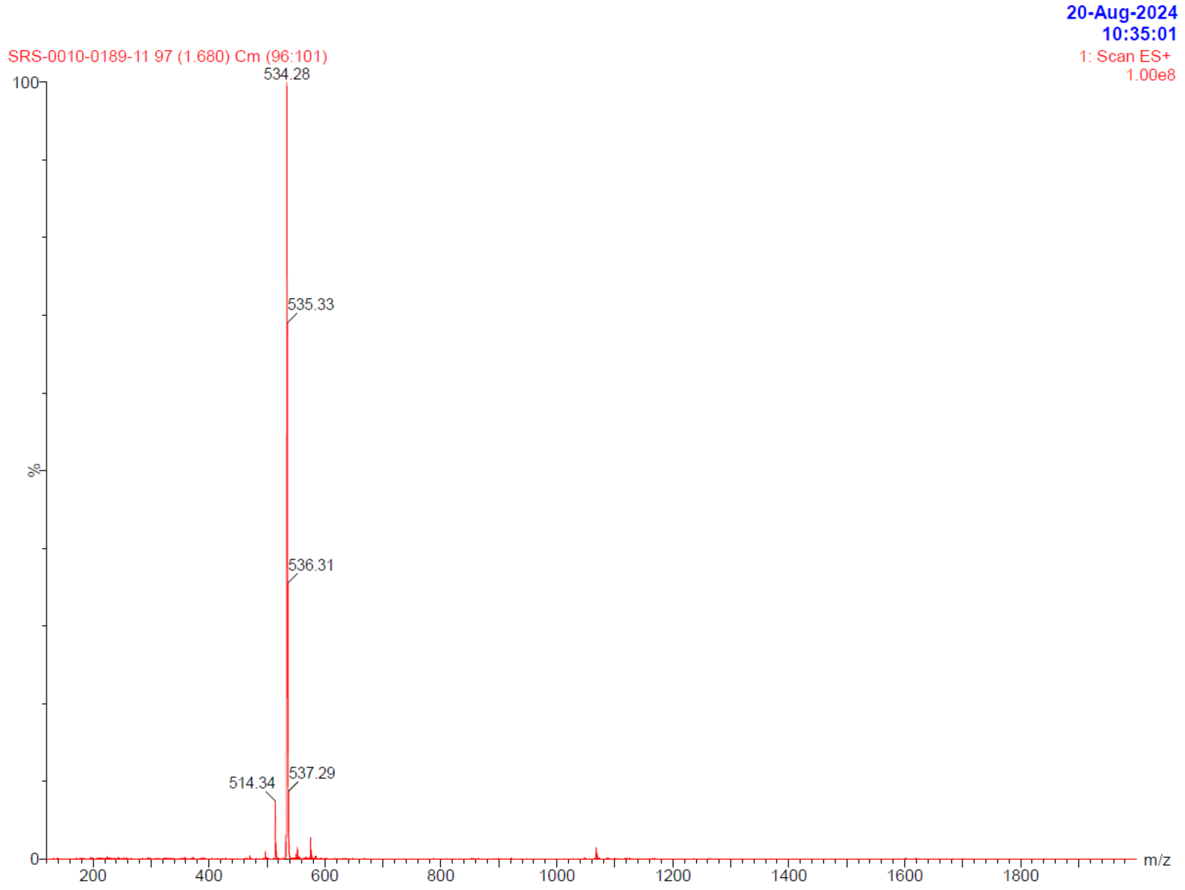

LCMS spectra for compound **5b**

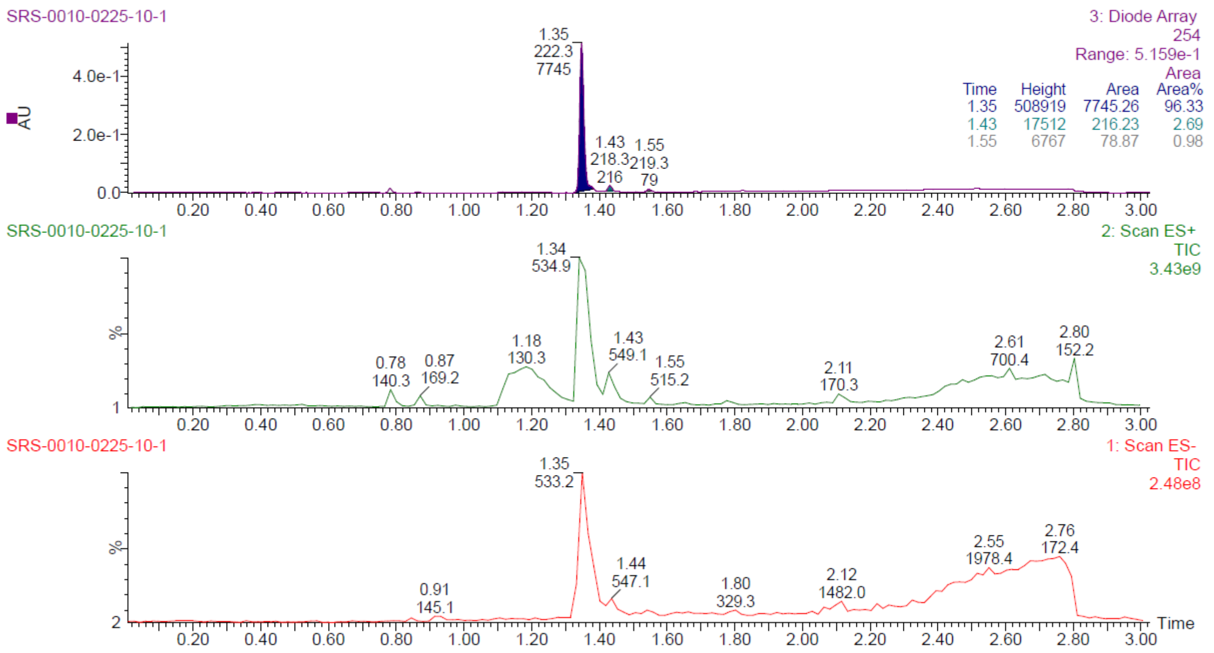

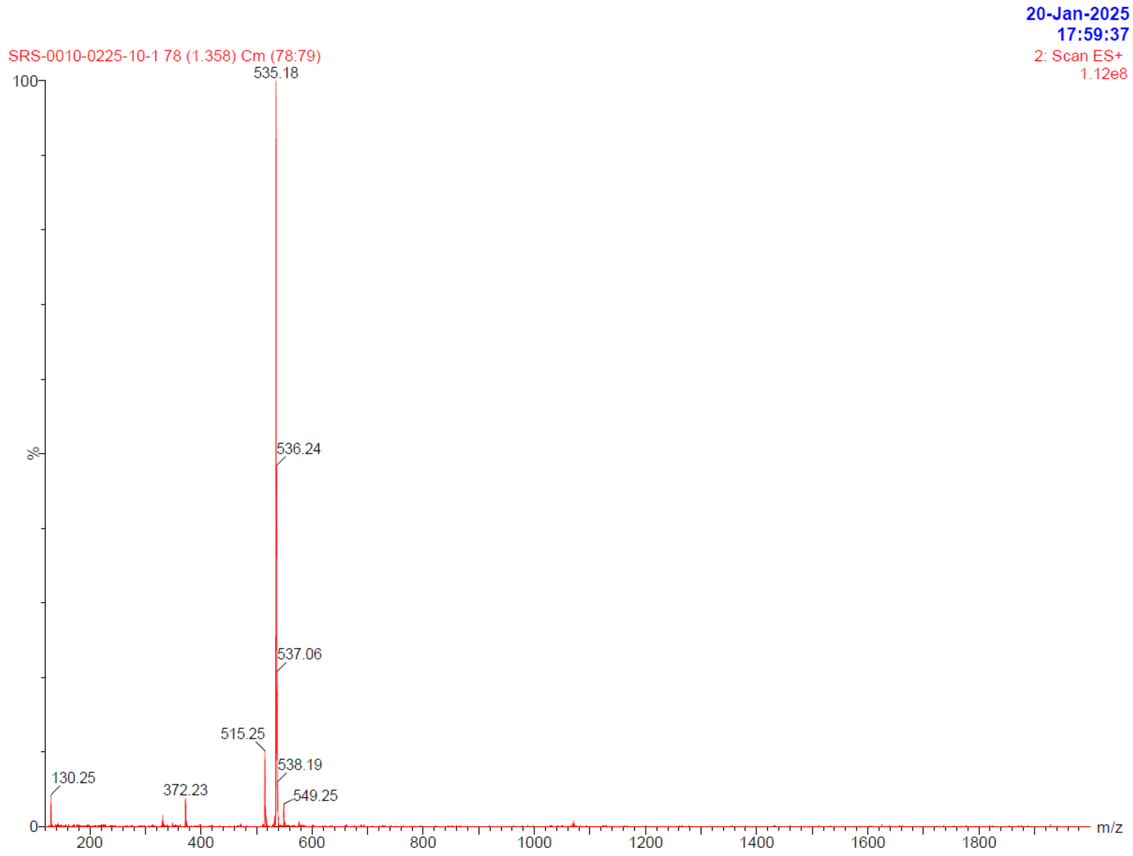

LCMS spectra for compound **5c**

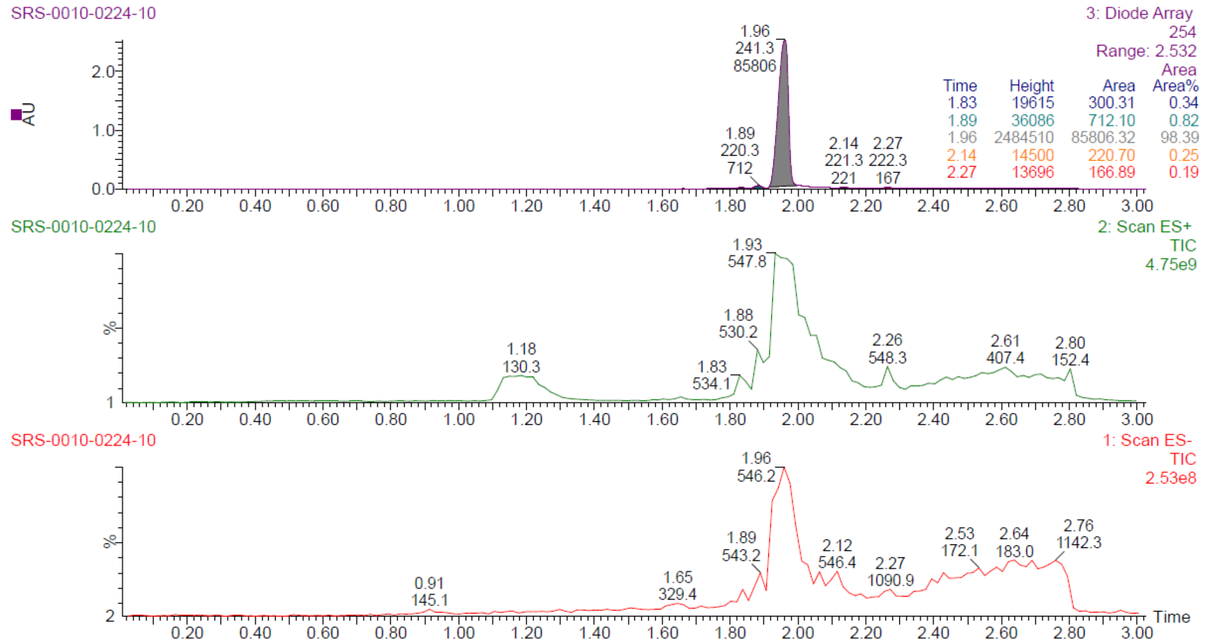

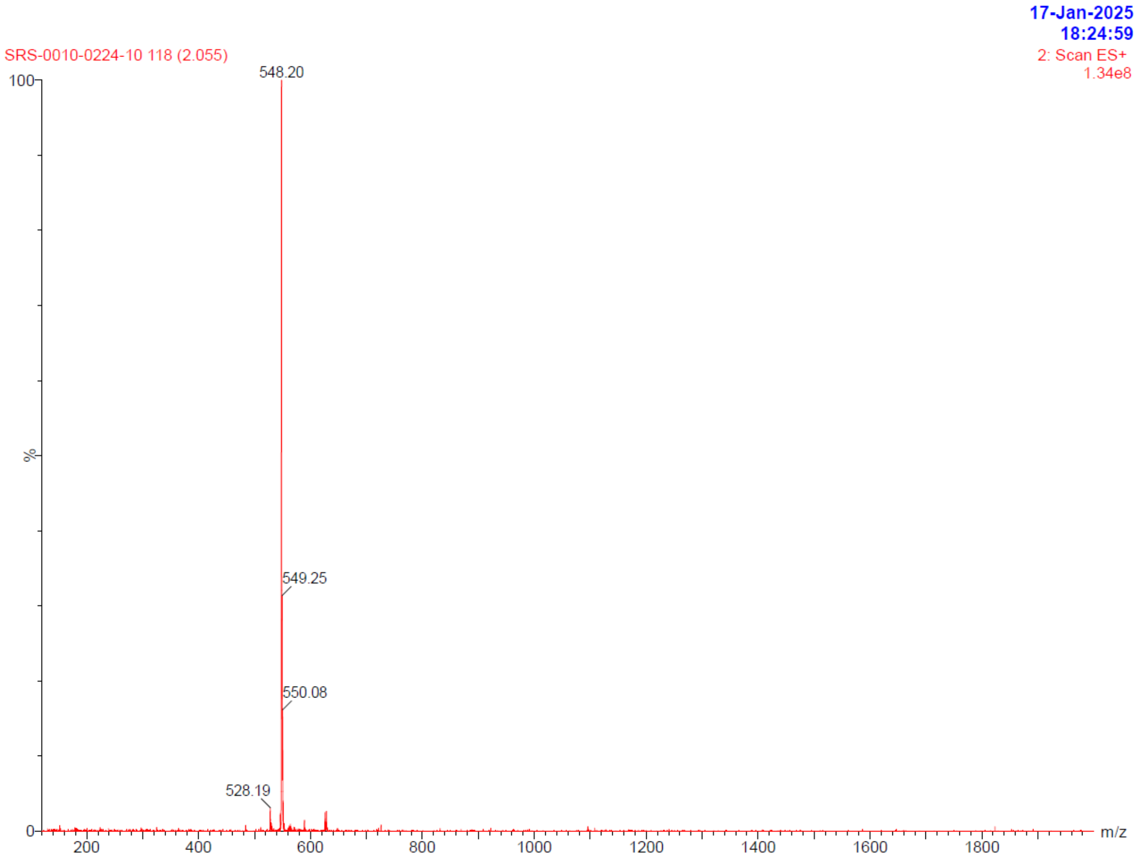

LCMS spectra for compound **5d**

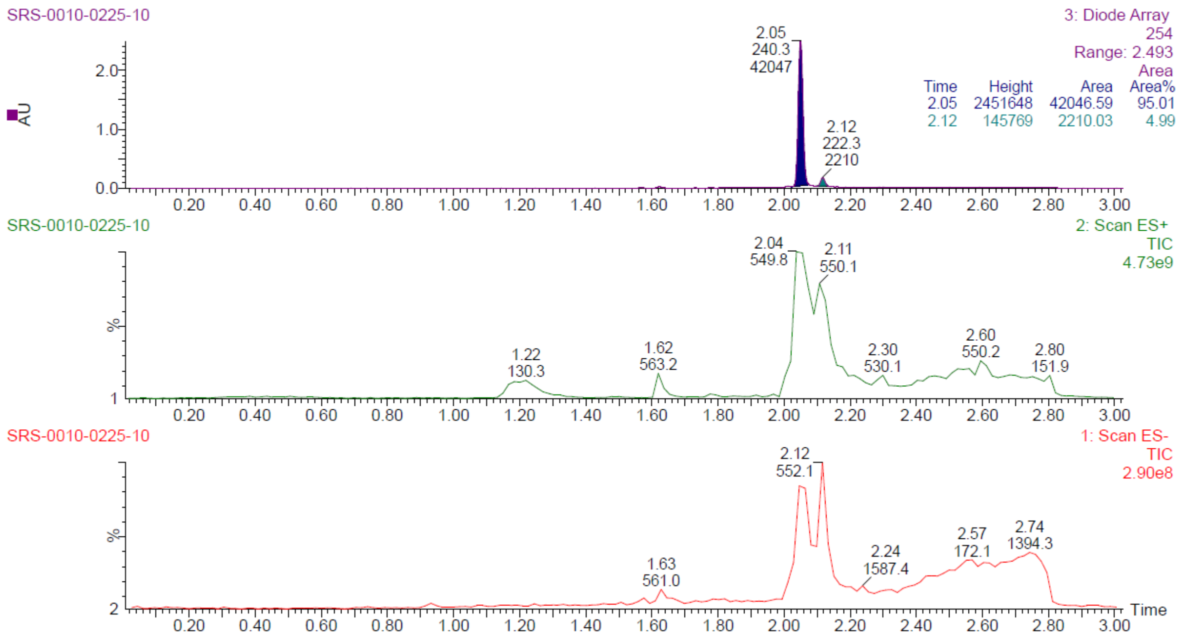

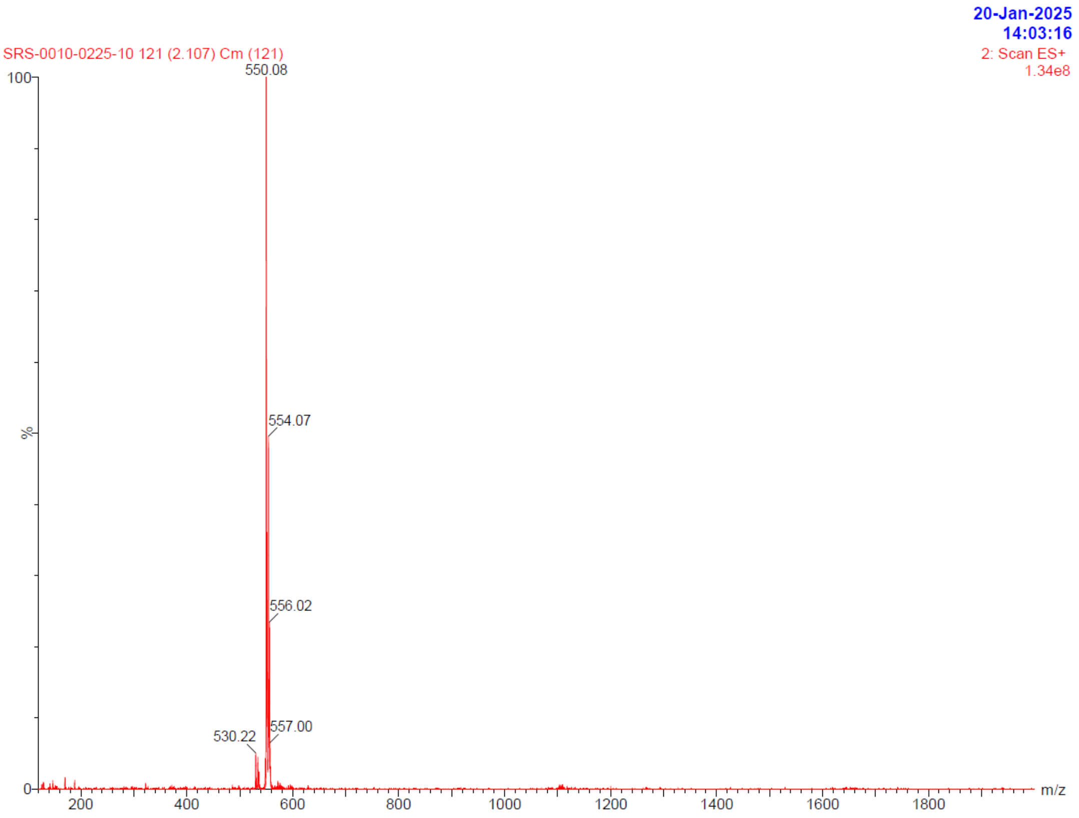

LCMS spectra for compound **5e**

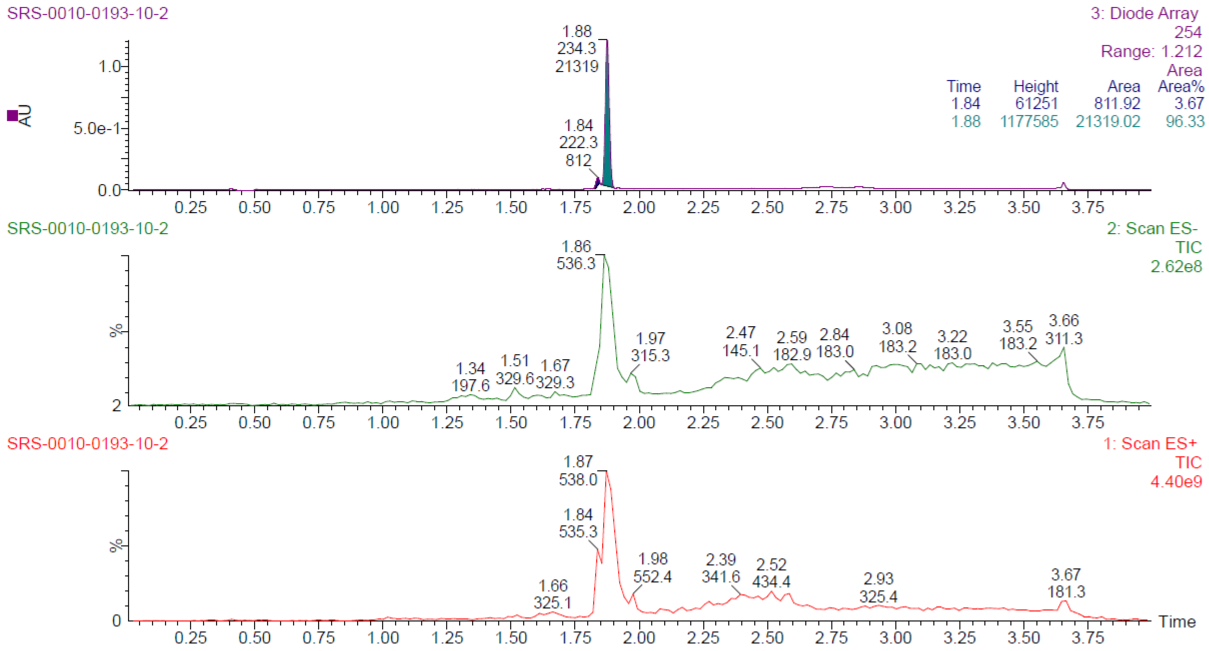

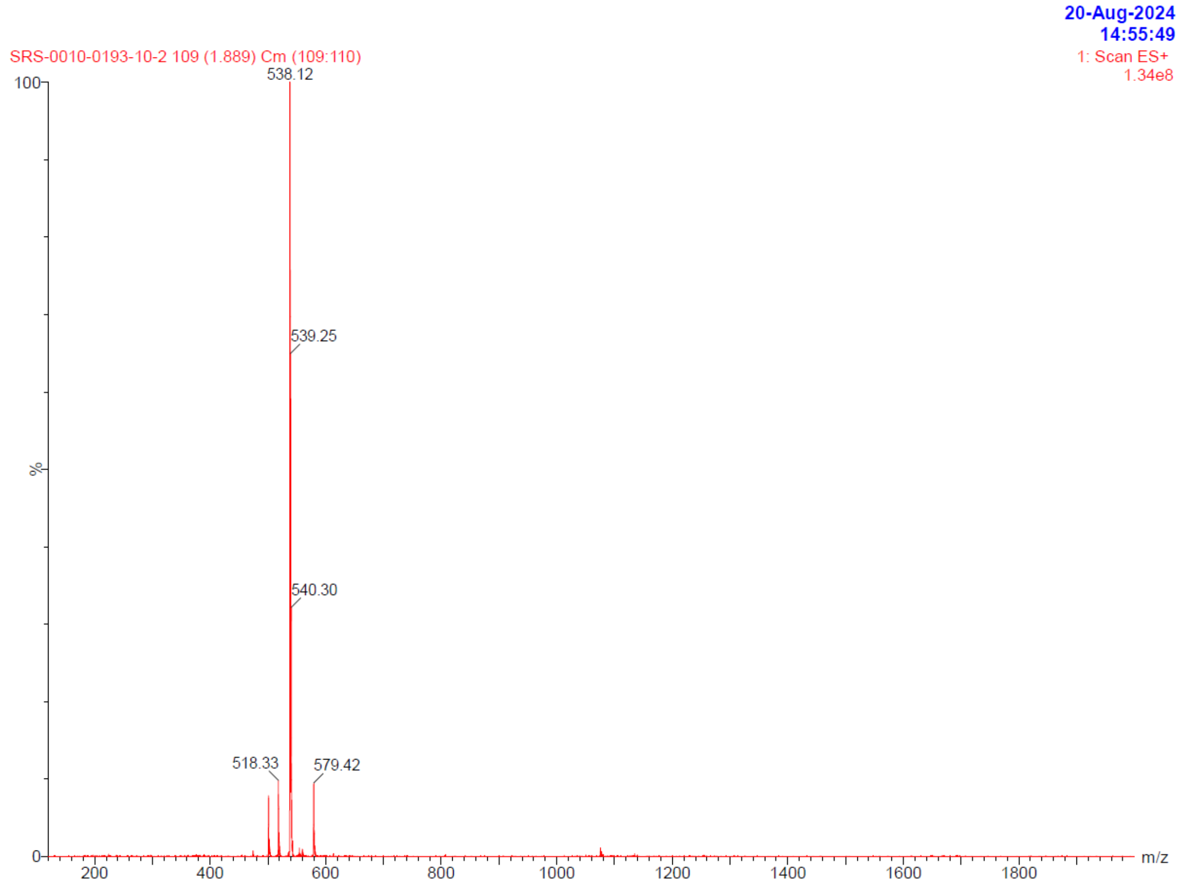

LCMS spectra for compound **5f**

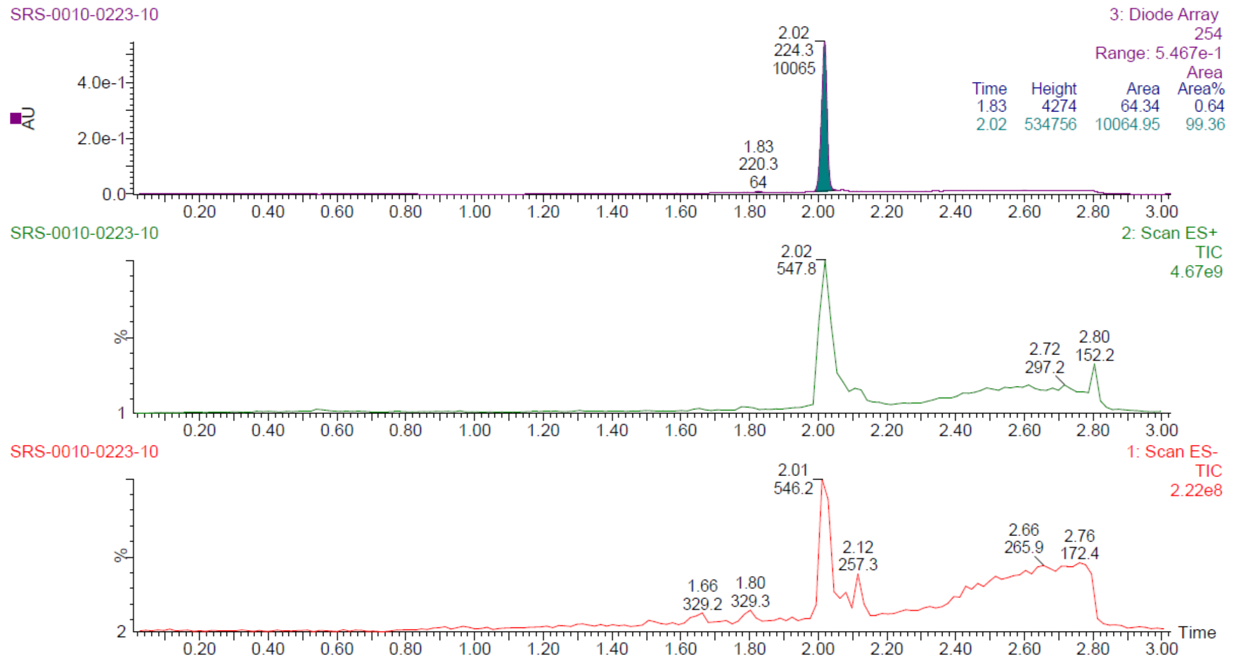

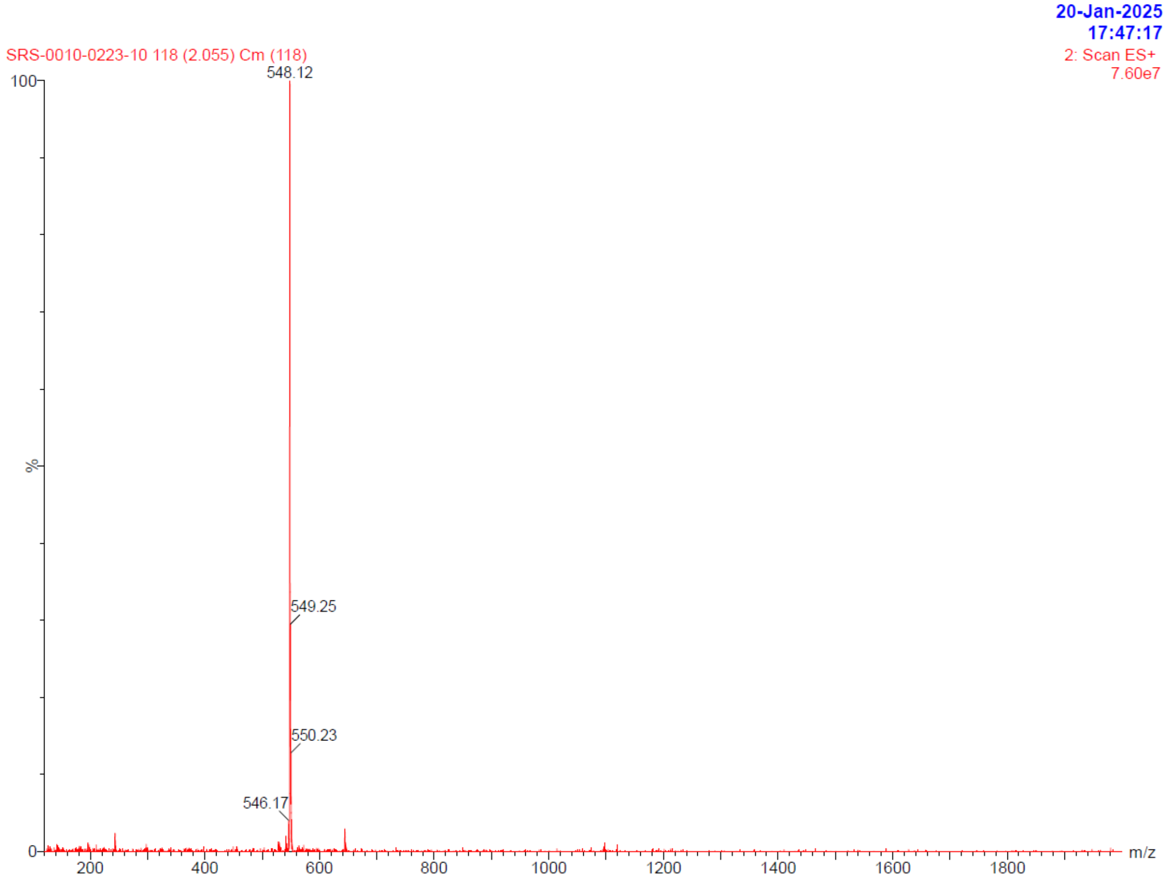

LCMS spectra for compound **5g**

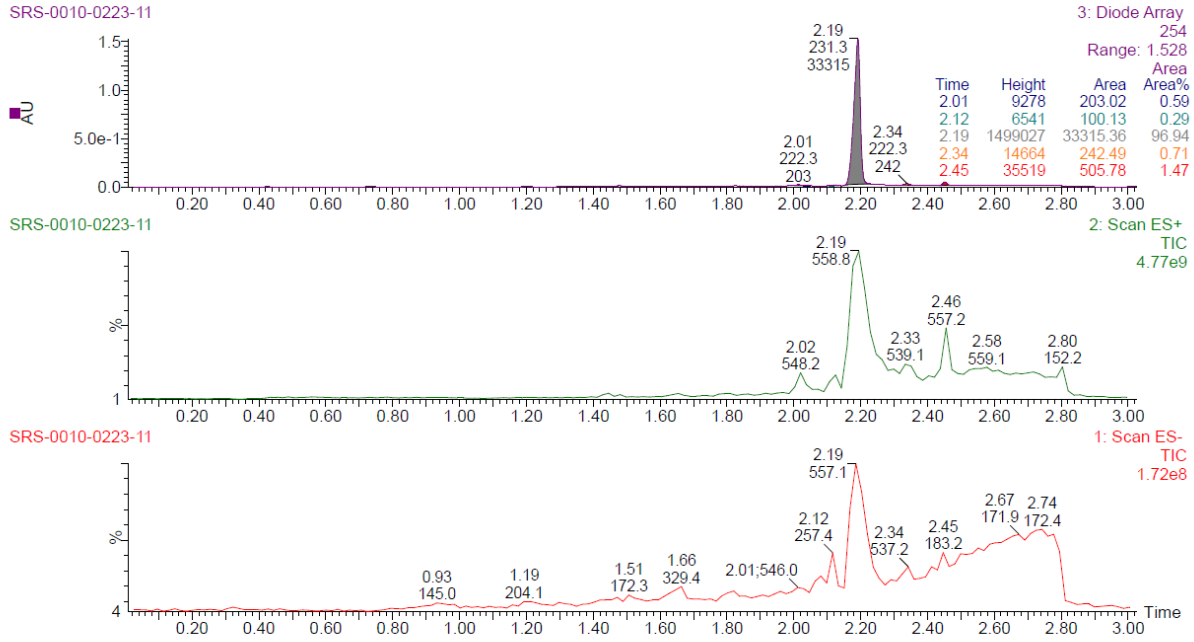

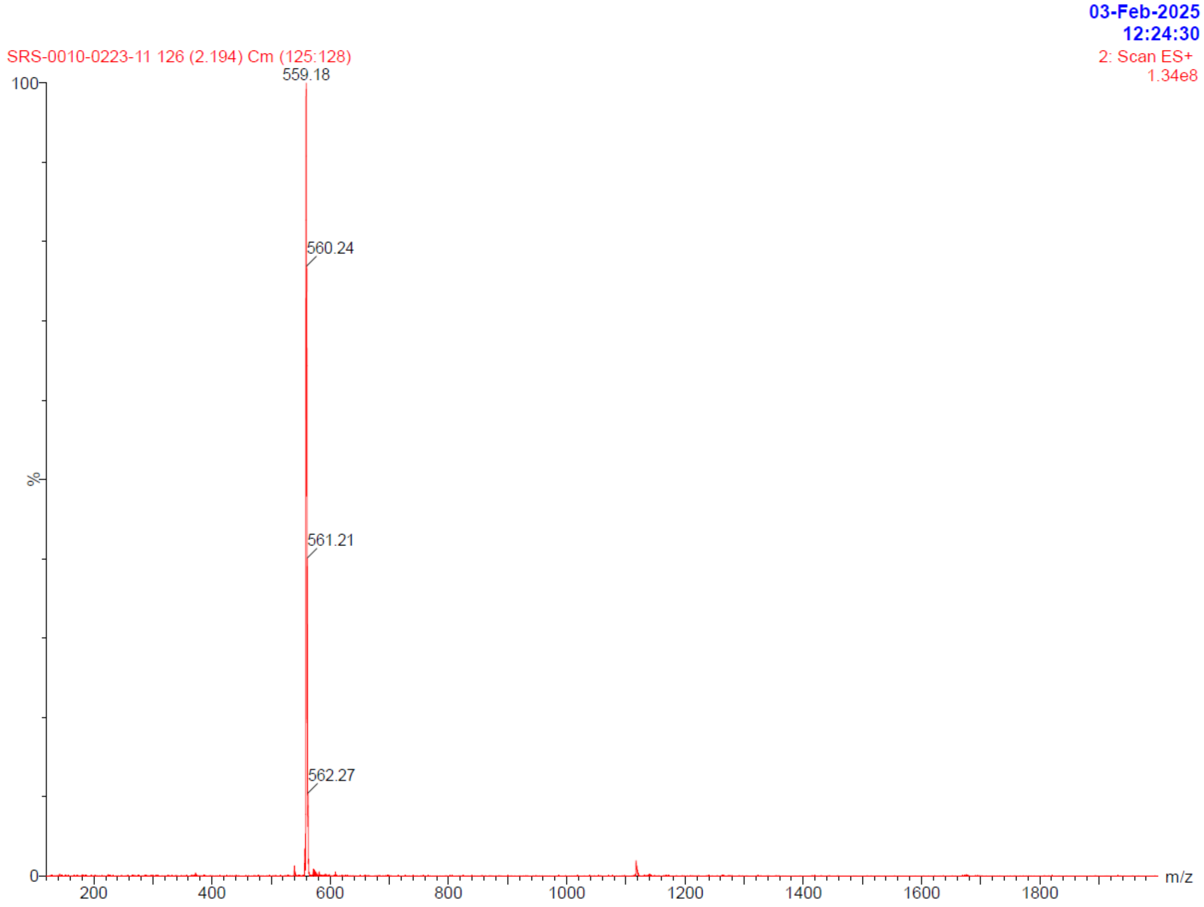

LCMS spectra for compound **9a**

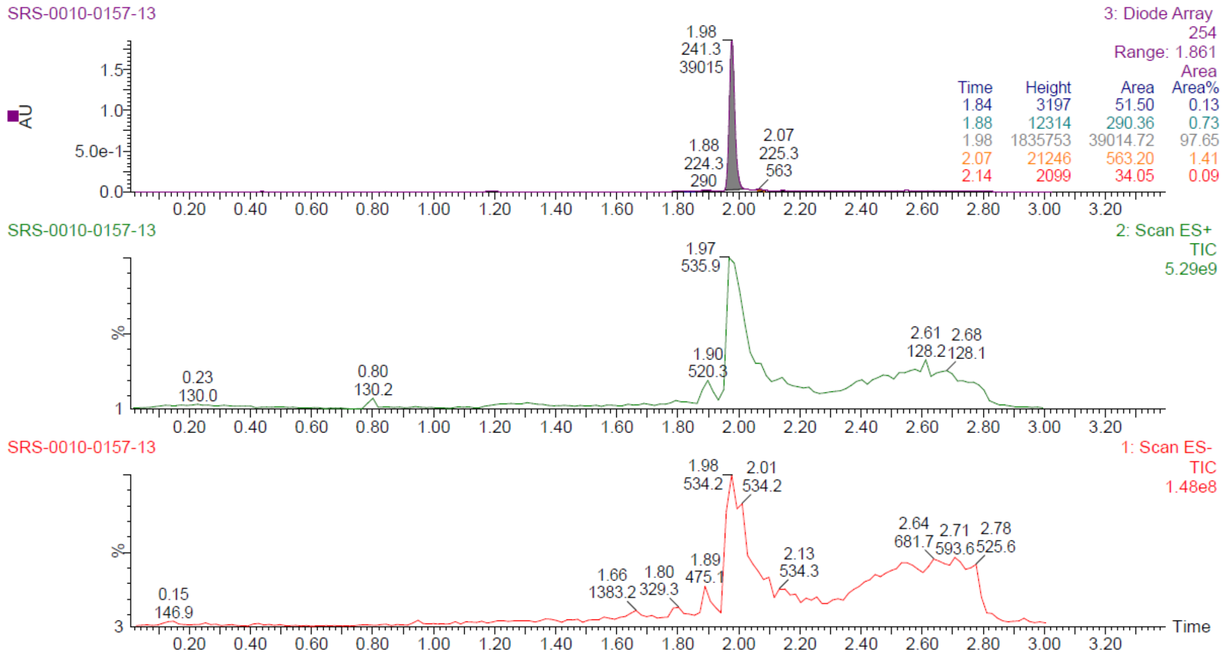

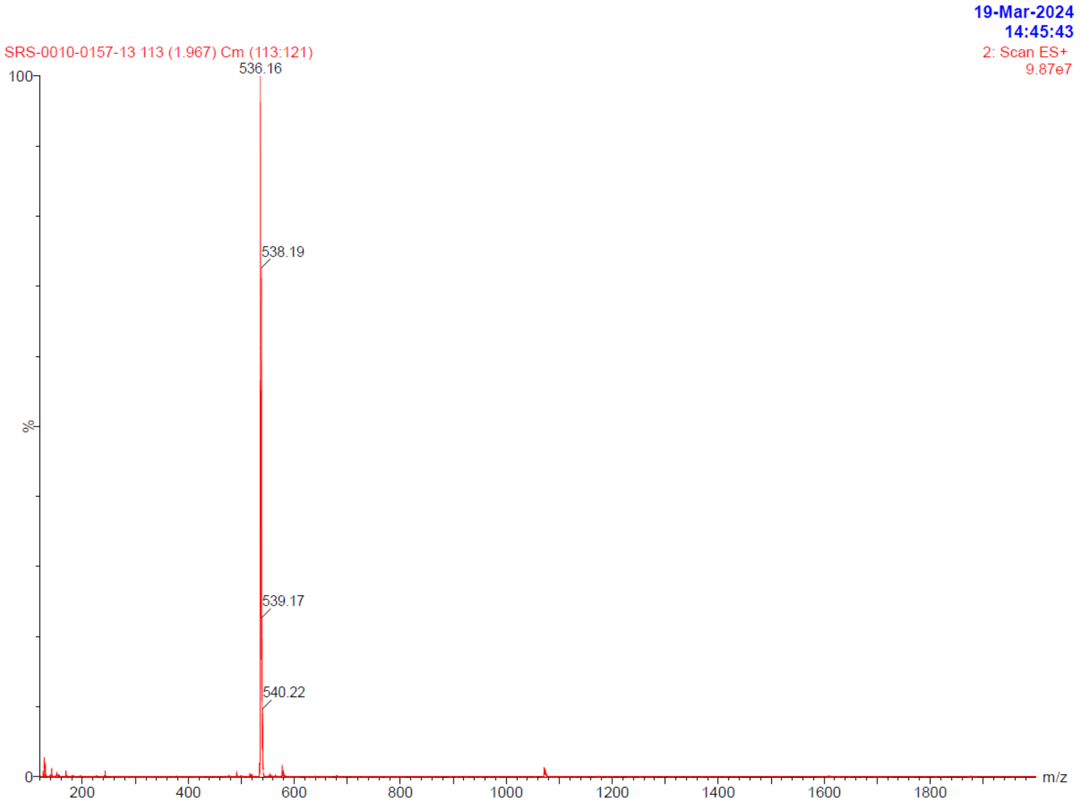

LCMS spectra for compound **9b**

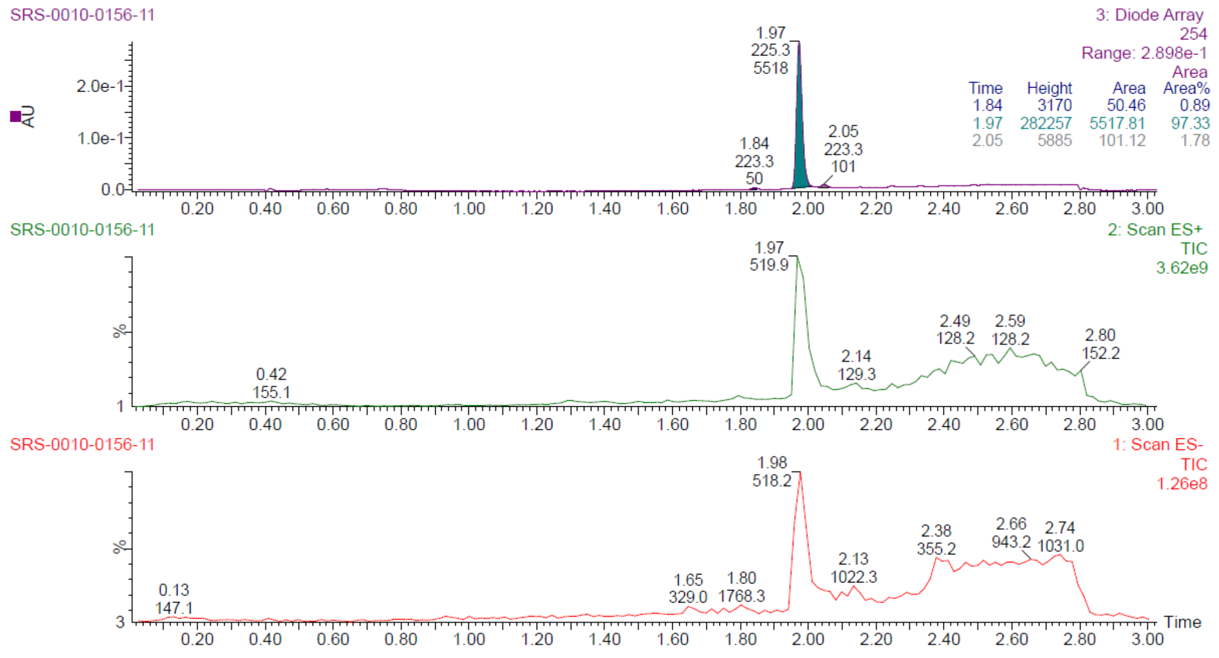

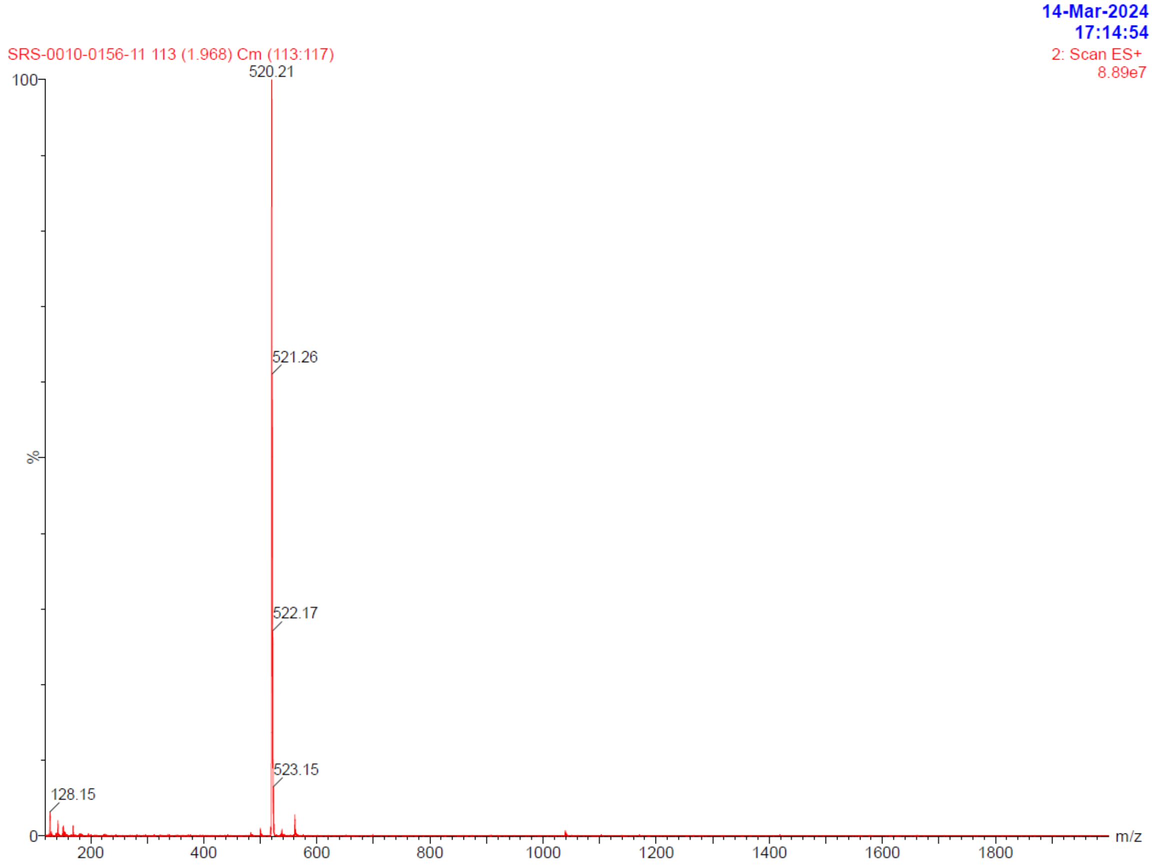

LCMS spectra for compound **9c**

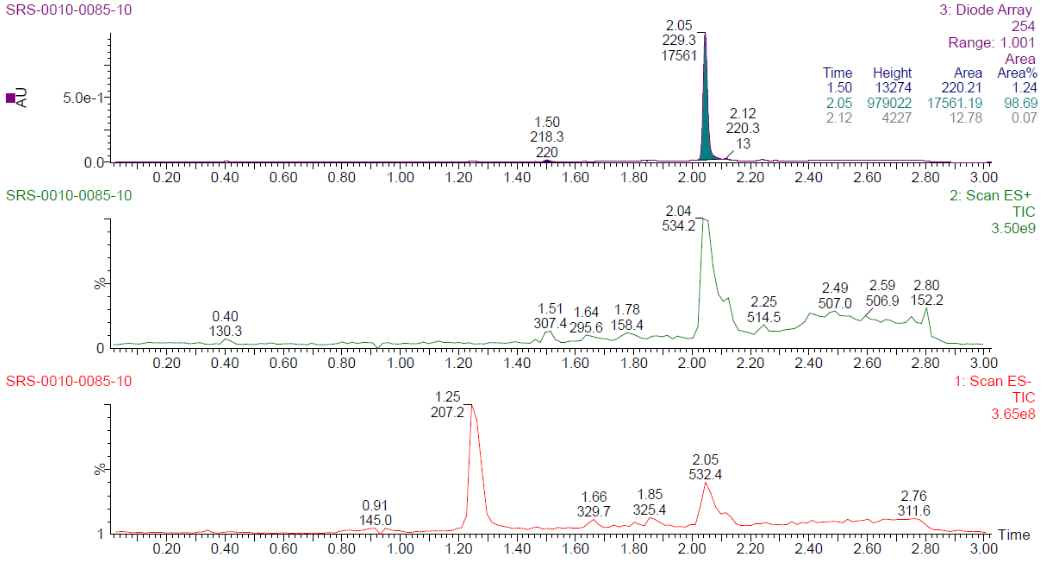

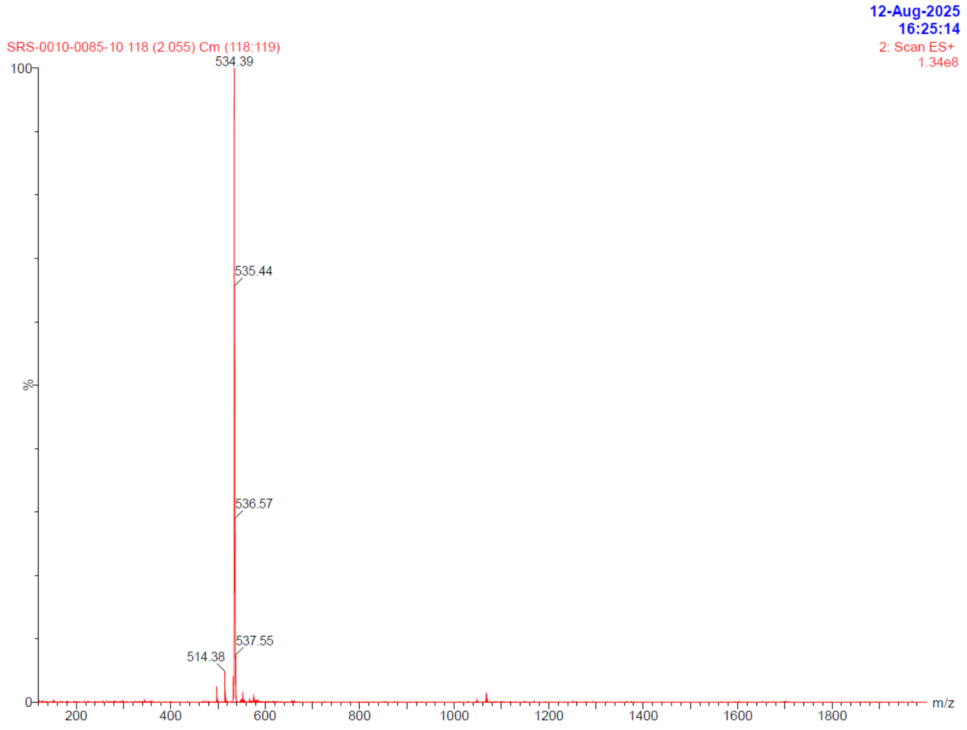

LCMS spectra for compound **9d**

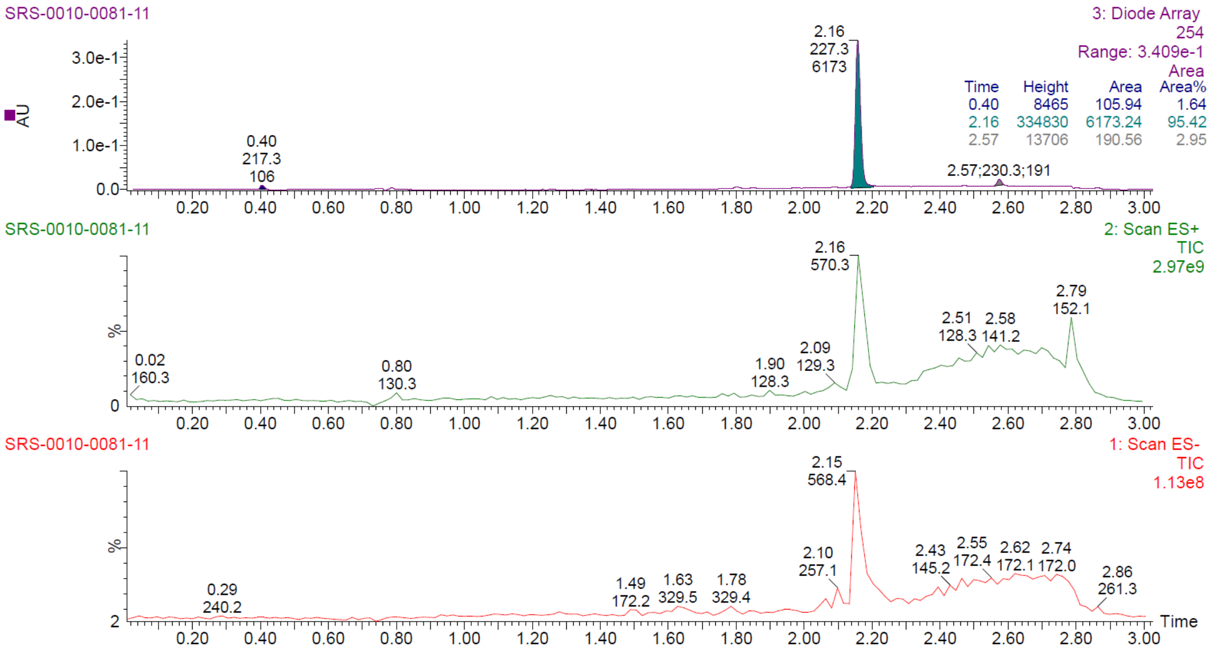

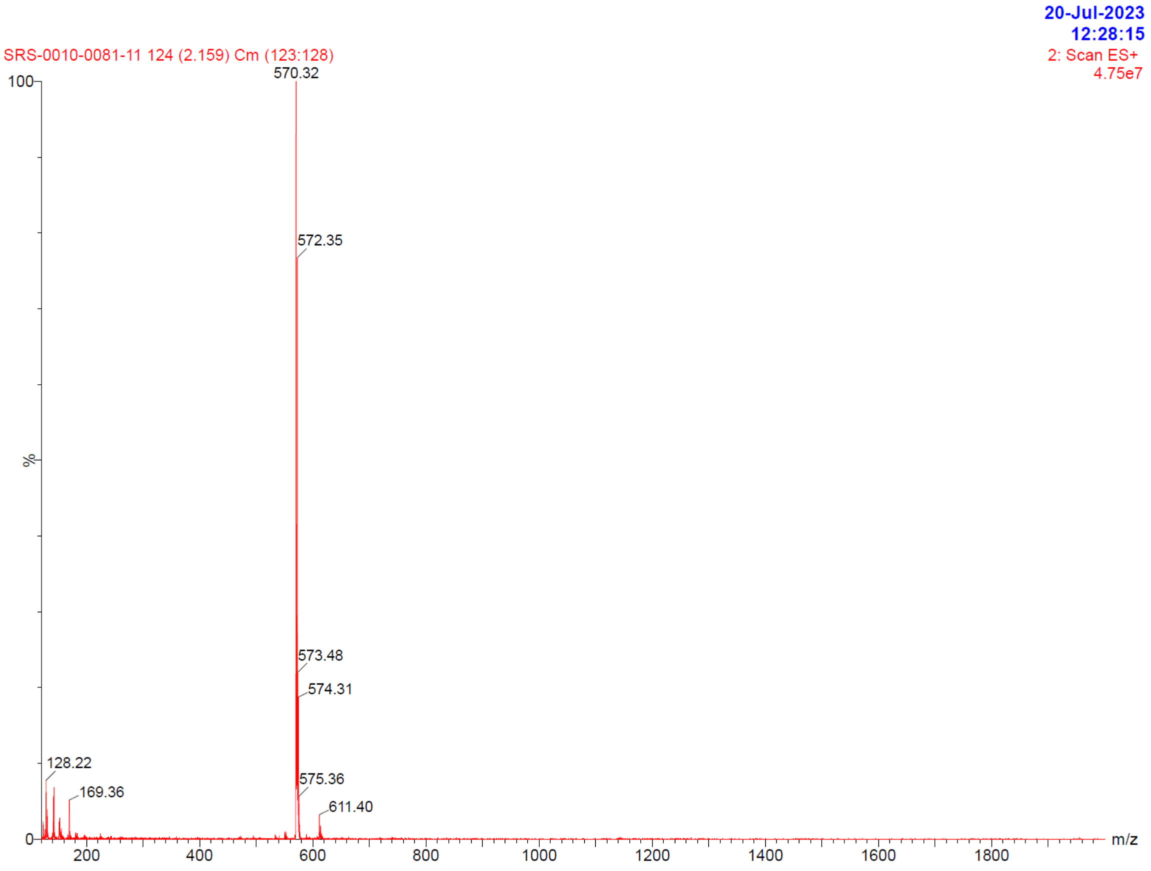

LCMS spectra for compound **9e**

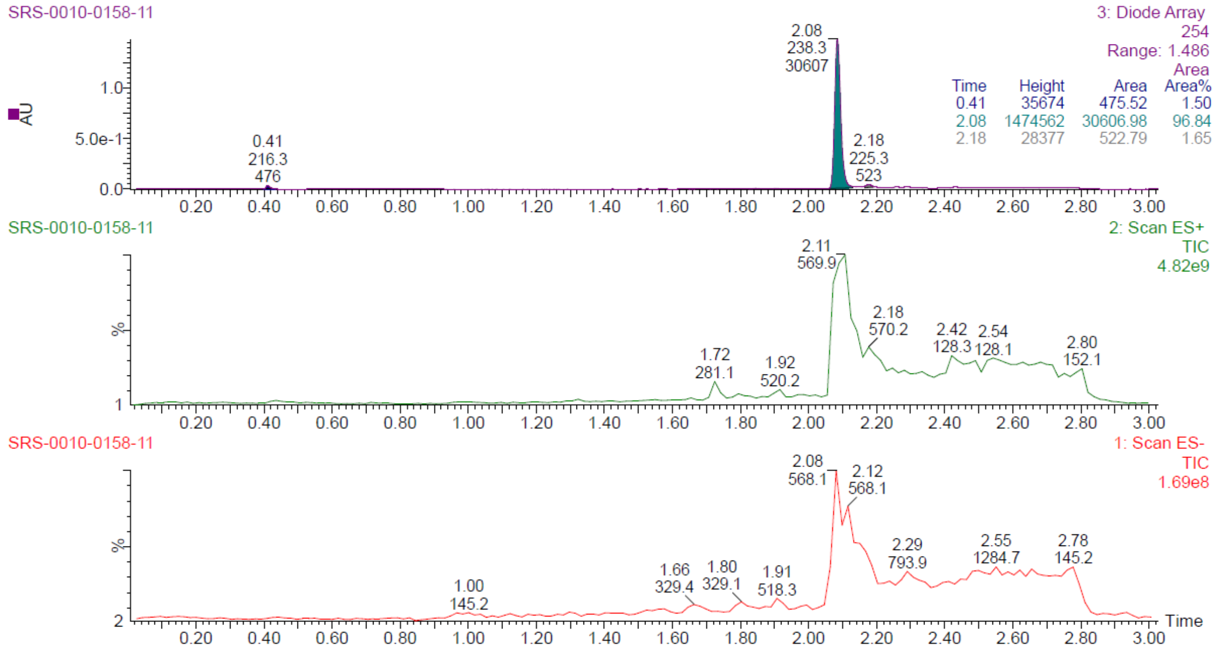

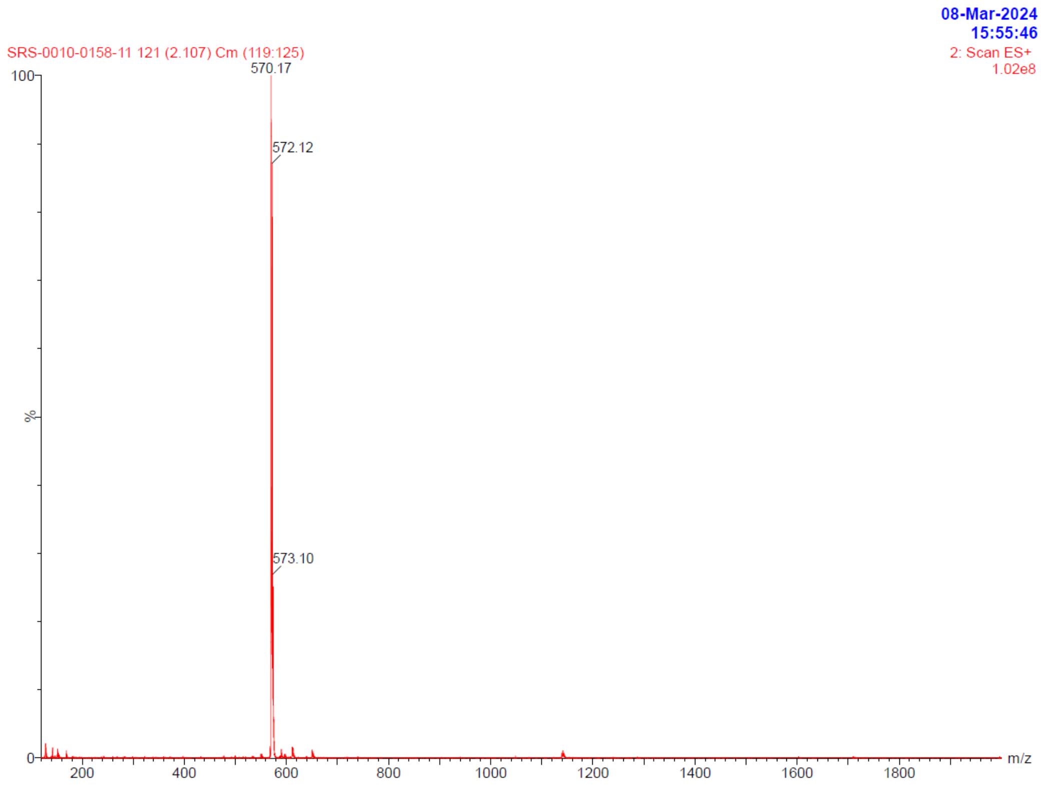

LCMS spectra for compound **9f**

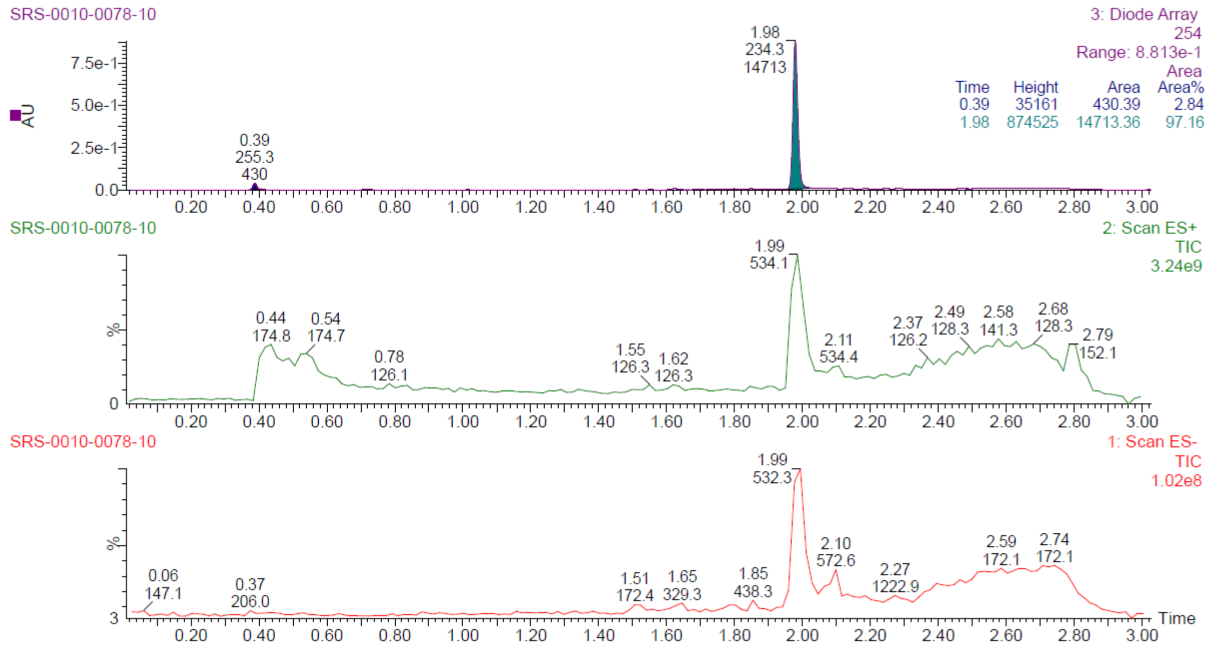

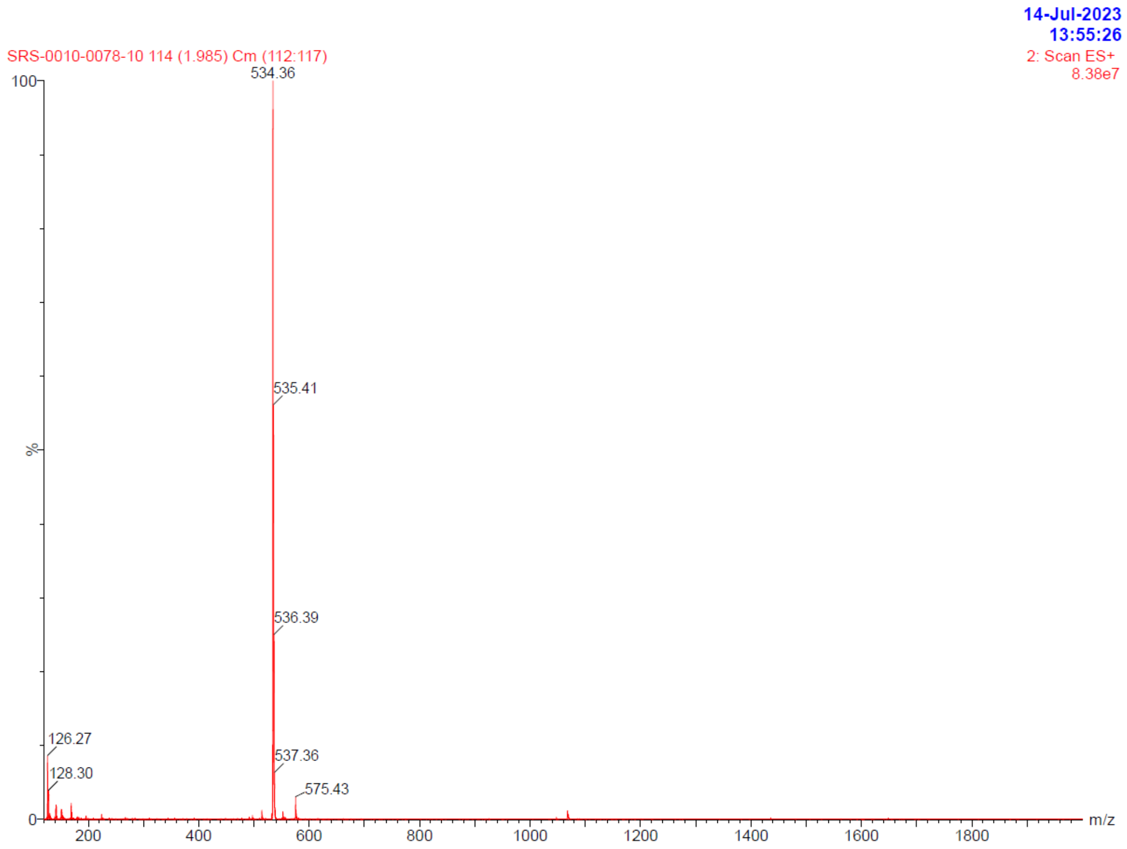

LCMS spectra for compound **9g**

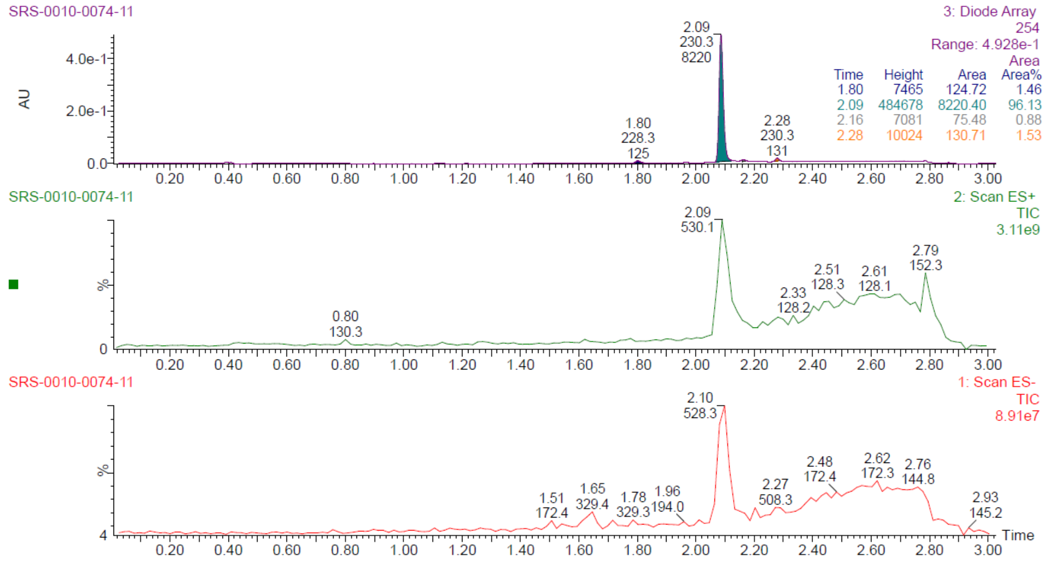

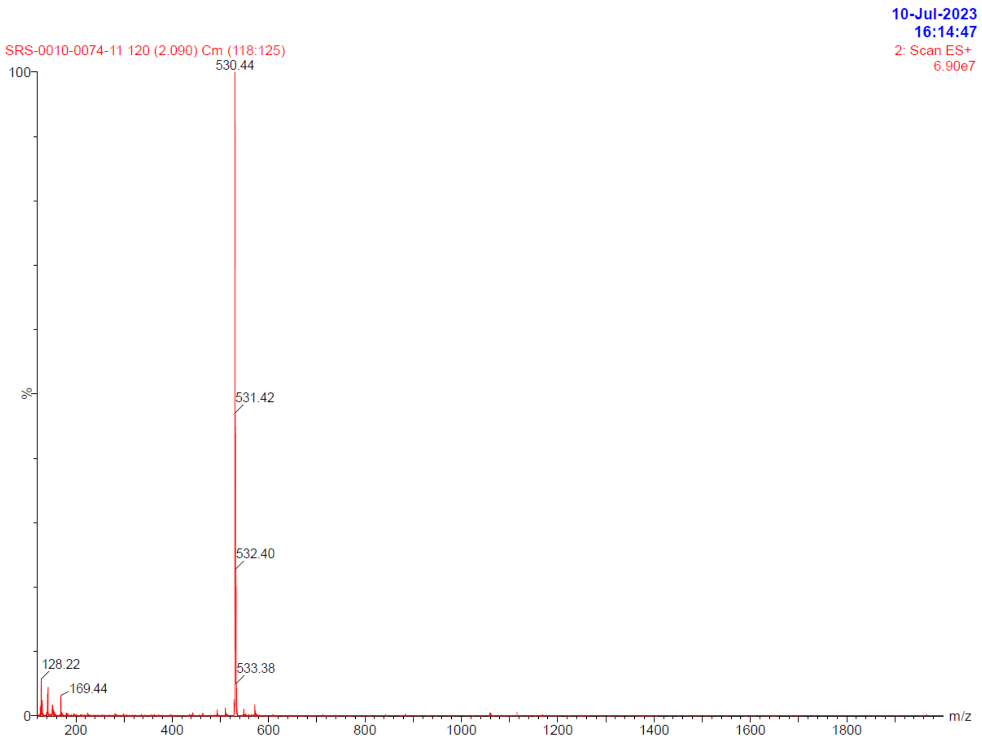

LCMS spectra for compound **11**

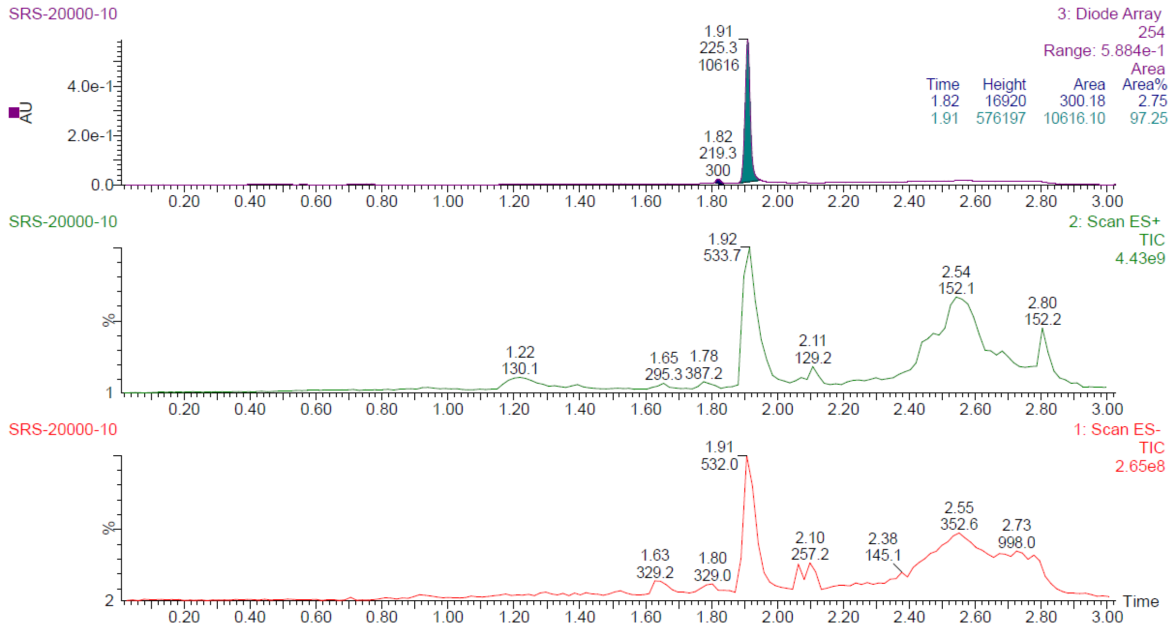

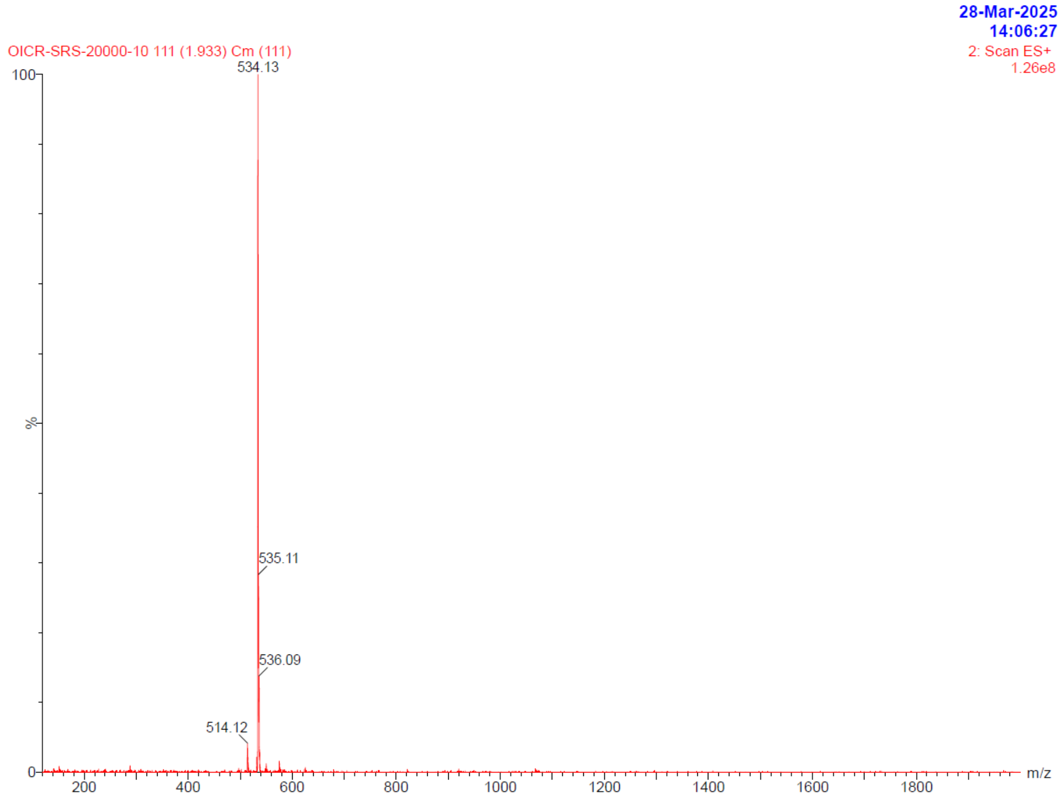

Table S1. Bolz2 analysis output for OICR kinase library screen (associated with **Figure 1**).

affinity_probability_binary	affinity_pred_value	inhibitor	PDB	drug
0.949086845	-2.381996155	PIK-75_hydrochloride	6gn1	
0.870629907	-2.15611124	TAK1_inhibitor	4L53	
0.629960299	-1.944641352	_5Z_-7-Oxozeaenol		
0.803962767	-1.899152756	Gilteritinib	6jqr	gilteritinib
0.913902044	-1.859213471	A-443654	2jdr	
0.403842866	-1.656147242	RAF709		
0.087087944	-1.651736259	FK-506		
0.525977075	-1.633382797	PHA-665752		
0.269260645	-1.60321784	GDC-0032		
0.068187907	-1.595876217	FGF401		
0.526108384	-1.540652275	Temsirolimus		
0.806342006	-1.533747911	SGI-7079		
0.602460265	-1.498177528	HDS_029		
0.787214875	-1.487784386	OTSSP167	4CQG	
0.644244432	-1.455658436	JNJ-28312141		
0.055364519	-1.450004339	Pimecrolimus		
0.770232797	-1.446525097	GSK-2118436B	4xv2	dabrafenib
0.751610875	-1.439136505	LGH447	5DWR	
0.179348052	-1.411057234	GSK-1120212		
0.718443334	-1.398660898	CNX-774		
0.829029083	-1.395325184	MK-1775	5v5y	
0.598249435	-1.392098904	PF431396		
0.770168066	-1.377500057	PD_166285_dihydrochloride	5VC5	
0.036286559	-1.365311384	Torin_1		
0.287933052	-1.36265111	Erdafitinib		
0.762977958	-1.347178221	LY2835219	7O7K	abemaciclib
0.531396687	-1.346767902	Everolimus		
0.885061324	-1.343996167	PHA-848125	2WIH	
0.479890466	-1.323966026	Cerdulatinib		
0.849392653	-1.29577899	URMC-099		
0.819179595	-1.279085517	SB-218078	1NVS	
0.847833276	-1.274111748	Dianilinopyrimidine_01		
0.609496474	-1.271860719	ON-123300		
0.715397477	-1.268510103	PRT-2607		
0.590200782	-1.240021229	BI-D1870		
0.711713314	-1.23410964	AVL-292		
0.315034628	-1.230598211	Ridaforolimus		
0.470080137	-1.201246738	CP-22		
0.755575299	-1.182056069	GSK-1059615		
0.64694953	-1.181971073	GW-843682X		
0.829102278	-1.171656132	R-112		
0.815811872	-1.168987751	Staurosporine		
0.218892336	-1.166946888	BLU-285		
0.648837864	-1.135908127	CT-98014		
0.460235119	-1.134488344	BMS-536924		
0.646282792	-1.122392893	BAY_61-3606_hydrochloride		
0.825944364	-1.102496147	CYT11387		
0.282271147	-1.098431706	LY2784544		
0.695964217	-1.097787142	WZ8040		
0.104653127	-1.096867204	PX-866		
0.117987767	-1.096479654	Rapamycin		
0.691703737	-1.079999685	GS-9973		
0.75800705	-1.070796251	Defactinib		
0.823292136	-1.068731785	Lck_inhibitor		
0.779525399	-1.055498838	TG101209		
0.619217694	-1.054127574	SB-415286		
0.548489392	-1.047529697	Rottlerin		
0.590602279	-1.045229077	TCS_2312_dihydrochloride		
0.544049621	-1.041330814	XL-880		
0.213665023	-1.04085815	AZ-20		
0.641690612	-1.036461353	R406		
0.327563524	-1.035158396	BIX-RSK2_Inhibitor		
0.802023888	-1.023254633	IRAK_inhibitor_6		
0.702972531	-1.012196898	PIK-93		
0.685527325	-1.007790089	AMG-47a		

0.643598557	-1.001286983	R-333		
0.107398376	-0.997417212	GSK-1904529A		
0.050819993	-0.997070849	RO-5126766		
0.154927716	-0.992961586	NSC_109555_ditosylate		
0.567698717	-0.983910203	Encorafenib		
0.048589915	-0.974845171	BMS-599626		
0.284217238	-0.951869726	BMS-5		
0.627746165	-0.94756031	CP-673451		
0.616004467	-0.946068168	WZ3146		
0.332839072	-0.94155705	BAY_1000394		
0.671935678	-0.92583853	Olmutinib		
0.621949792	-0.916999578	SU-11274		
0.326028556	-0.914487243	R428		
0.522154212	-0.908411026	FRAX-486		
0.568856955	-0.907092929	Purvalanol_B		
0.149752498	-0.89357996	Ki-20227		
0.721299171	-0.886765718	GLPG-0259		
0.500436068	-0.875095248	CCT137690		
0.198753029	-0.870810866	IPI-549		
0.78761065	-0.868488193	K-252a		
0.609827459	-0.865614355	Brigatinib		
0.730073452	-0.8517977	AT9283		
0.380009592	-0.845573425	CHIR-124		
0.623280942	-0.836667776	PD-158780		
0.164832115	-0.833444595	Gedatolisib		
0.466037989	-0.832915068	CO-1686		
0.663165033	-0.824577689	MPI-0479605		
0.602841258	-0.813361049	G-749		
0.362369657	-0.808713436	PLX7904		
0.208204716	-0.804469287	Wortmannin		
0.252213806	-0.799989283	VX-702		
0.093657583	-0.798881173	PD318088		
0.771595597	-0.794976115	Lestaurtinib		
0.650643229	-0.794716835	AS-252424		
0.663096607	-0.794329345	Pacritinib		
0.044816054	-0.786934614	AS-703026		
0.377823919	-0.786770046	VPS34-IN1		
0.654246509	-0.774244785	AMG458		
0.236969054	-0.771695971	MLN8054		
0.45011121	-0.765809953	Ensartinib		
0.128987134	-0.75214982	JNJ-38877605		
0.310841799	-0.75171864	AG-183		
0.353961647	-0.750696778	ZSTK474		
0.223551631	-0.750589132	GSK_2334470		
0.08406391	-0.746097744	EMD-1214063		
0.399280131	-0.735782743	BMS-754807		
0.528055549	-0.71147424	GW-5074		
0.595960617	-0.710303187	NMS-1286937		
0.512961924	-0.709741771	LDN-212854		
0.058873691	-0.704691768	AMG-511		
0.461891174	-0.700867891	R788		
0.152529061	-0.697416008	MLN-8237		
0.5792799	-0.696473956	WH-4-023		
0.129990518	-0.69512248	XL-147		
0.507734358	-0.691078067	TCS_2312		
0.808137596	-0.689730108	PD173955		
0.206535041	-0.68764478	BMS-3		
0.247157156	-0.687325954	Ro-31-8220_mesylate		
0.075937718	-0.685317755	BMS-582664		
0.733182251	-0.681566358	SGI-1776		
0.095092267	-0.67907244	PF_3644022		
0.174374878	-0.67026329	AZD3264		
0.583786547	-0.66669625	CEP-37440		
0.50204128	-0.663024366	LY-2606368		
0.55817008	-0.656590223	LJH685		
0.603690147	-0.656369448	Dinaciclib		
0.196652979	-0.643578887	MK-2206		
0.508272588	-0.64026165	PF562271		

0.091823913	-0.640011251	AZD1390		
0.768629909	-0.637166083	Hesperadin		
0.507155299	-0.63119638	THZ1		
0.750474274	-0.629326284	TAE-684		
0.077791467	-0.625817657	RHO-15		
0.198428899	-0.605088592	Alvocidib		
0.65568471	-0.603222311	CGP-74514A_hydrochloride		
0.491635203	-0.599131465	6-bromoindirubin-3_-oxime		
0.404558748	-0.594873428	Bosutinib		
0.20020169	-0.593100309	AC220		
0.572460473	-0.589810133	AG_213		
0.098360993	-0.588380337	RDEA-119		
0.153194696	-0.587735772	AZD-0156		
0.341549635	-0.587074935	Bafetinib		
0.798176169	-0.586801767	LY3009120		
0.613910496	-0.585753441	GSK-461364		
0.573454916	-0.581754684	BX912		
0.42377609	-0.580202103	BX-795		
0.571468294	-0.57904613	PHA-739358		
0.276618361	-0.569388986	BGT-226		
0.483153909	-0.568365276	SU-5402		
0.25598672	-0.567579865	Radotinib		
0.632467687	-0.566067994	Dorsomorphin_dihydrochloride		
0.089748561	-0.547086596	BIBW-2992		
0.559196949	-0.542598963	AP-24534		
0.388794959	-0.541803718	TG100-115		
0.169959977	-0.538542509	Bisindoylmaleimide_X		
0.649077177	-0.538178682	TG-101348		
0.190125495	-0.537172556	MK-8033		
0.30522579	-0.536741734	ALW-II-41-27		
0.760807872	-0.535585523	CX-4945		
0.666426599	-0.533771873	GSK-650394		
0.397316635	-0.529302835	PF-02341066		
0.105426289	-0.527734578	BAY_80-6946		
0.582560897	-0.526307106	AMG-Tie2-1		
0.208569407	-0.524529755	RN486		
0.461367399	-0.523639381	PIM_1_Inhibitor_2		
0.308185846	-0.520483315	Ro3280		
0.267089128	-0.514092445	AZD-1152-HQPA		
0.521050572	-0.513089895	NG_25		
0.189331025	-0.502478778	XL-388		
0.339768767	-0.500369608	UNC-2250		
0.013425615	-0.493020445	Merck-22-6		
0.663102627	-0.492182106	NVP-BSK805		
0.589176714	-0.490796387	Dorsomorphin		
0.402699649	-0.488515586	UNC-569		
0.748552203	-0.48772189	Arcyriaflavin_A		
0.420818061	-0.487590998	TC-A_2317_hydrochloride		
0.565446675	-0.486480504	L-779450		
0.757494509	-0.48408103	BIRB_796		
0.176297069	-0.481959462	Selonsertib		
0.547225237	-0.479071349	CP466722		
0.216475874	-0.476878554	PF-04691502		
0.710740566	-0.47609067	AZD_7762		
0.087881401	-0.474478632	Asciminib		
0.104457594	-0.469146371	PD-184352		
0.540493131	-0.463967532	IPA_3		
0.737178802	-0.458309233	PD-407824		
0.735081792	-0.456053317	K-252c		
0.179677904	-0.45427689	YM201636		
0.629884124	-0.453740001	IKK_16		
0.120410919	-0.433893085	Tenalisib		
0.055319812	-0.427374005	SAR405		
0.450201422	-0.419907659	NVP-BHG712		
0.225260586	-0.416570038	SR3677		
0.735874176	-0.415816665	CYC-116		
0.378567636	-0.415549725	PF-573228		
0.150393248	-0.411923349	GDC-0980		

0.34837687	-0.408788353	SB590885		
0.758157134	-0.40163973	Crenolanib		
0.121209145	-0.39347297	Brivanib		
0.442445636	-0.39157179	R-348		
0.197943687	-0.387204617	AZ-960		
0.442260057	-0.382821411	EKI-785		
0.126753867	-0.381359041	MK-5108		
0.287189484	-0.379938364	GW8510		
0.27740109	-0.377416223	Gleevec		
0.427473277	-0.37624073	Pazopanib_hydrochloride		
0.418382883	-0.375530958	CCT129202		
0.593154788	-0.374963105	PD-0332991		
0.626730084	-0.370598972	Palbociclib_HCl		
0.03819593	-0.368337303	PD-0325901		
0.237721696	-0.365225255	Nilotinib		
0.658511639	-0.361059785	Ellagic_acid		
0.203639895	-0.356600612	eFT508		
0.041184202	-0.348238856	KN-62		
0.579755008	-0.347622693	Cyclapolin_9		
0.073619843	-0.345283061	MGCD-265		
0.708146334	-0.33971253	AS604850		
0.328928083	-0.339581847	PLX-4032		
0.269138575	-0.338039219	FM-381		
0.606453776	-0.337924123	NU-6102		
0.257661581	-0.336806297	TAK-632		
0.395534039	-0.330161154	AZD-1152		
0.114573024	-0.328830361	BMS-863233		
0.526263177	-0.325093865	VE-822		
0.311832905	-0.324826866	OSI-906		
0.19695738	-0.323429525	Entrectinib		
0.100812733	-0.316125393	AZD-1208		
0.106994241	-0.306080729	GW-2580		
0.251004845	-0.303973496	AG13958		
0.224860102	-0.301303953	Lifirafenib		
0.123147547	-0.297104061	CC-930		
0.055565044	-0.295654058	PI-3065		
0.57738167	-0.291817605	CRT-0066101		
0.188419968	-0.288393021	CC-223		
0.350897431	-0.288342476	LY2090314		
0.047614541	-0.270567834	KU-60019		
0.613501787	-0.265460849	AZ191		
0.209071934	-0.265397608	LY2603618		
0.317254424	-0.258486241	BMS_777607		
0.451074183	-0.258263052	PF-06463922		
0.309047163	-0.257674515	ABT-869		
0.613963246	-0.256898284	ML167		
0.295267403	-0.255167842	SGX-523		
0.525651276	-0.251977086	Compound_401		
0.014576028	-0.250641316	Belizatinib		
0.130693734	-0.247563899	Savolitinib		
0.218151301	-0.246537834	GSK_PERK_Inhibitor		
0.376795322	-0.23613216	SCH-772984		
0.266994059	-0.235353708	Sal003		
0.172405124	-0.233795941	RWJ-67657		
0.330971569	-0.2259707	GDC-0879		
0.322907805	-0.218881965	IRAK_inhibitor_4		
0.095309958	-0.20633091	P276-00		
0.542715073	-0.203979939	Imidazolo-oxindole_PKR_inhibitor_C16		
0.324624419	-0.202615142	AG_825		
0.259264201	-0.196271285	GDC-0834		
0.434129	-0.192992643	AZD5438		
0.089770049	-0.190293163	HTH-01-015		
0.4742167	-0.188118637	WZ4002		
0.505364716	-0.186932877	LDN193189		
0.443659365	-0.186730847	INCB018424		
0.672098517	-0.186254367	AS605240		
0.069933899	-0.185175166	PF-04217903		

0.692058802	-0.171476677	GW441756_hydrochloride		
0.452963471	-0.167862028	TWS-119		
0.11699333	-0.167340606	ZM_336372		
0.620554984	-0.166018143	Dovitinib		
0.398772657	-0.161881283	IRAK_inhibitor_3		
0.190396518	-0.161380202	SAR-131675		
0.589995503	-0.157399148	TCS_359		
0.04495221	-0.155052796	AZD-2014		
0.334403813	-0.151960179	Peficitinib		
0.374144763	-0.150500074	GNE-0877		
0.300253689	-0.145430192	JNK-IN-8		
0.625071526	-0.1423181	CC-401		
0.618033767	-0.141647115	SU-9516		
0.47775507	-0.138893604	JNJ-7706621		
0.73419112	-0.136473581	VE-821		
0.397045791	-0.135287166	PI-103		
0.509357333	-0.13267538	PI-828		
0.391266197	-0.131802708	SNS-314		
0.495366603	-0.127906635	ENMD-2076		
0.688968182	-0.121521458	Compound_52		
0.352621913	-0.121124357	Neratinib		
0.160507157	-0.106665015	BI-6727		
0.396954298	-0.105850659	TCS_PIM-1_1		
0.362342656	-0.098335415	AG_494		
0.204864085	-0.096581444	TAK-715		
0.449822187	-0.092429921	Amcasertib		
0.10749808	-0.090183675	BRD-8899		
0.654050231	-0.089480616	MRX-6313		
0.474459171	-0.089077778	PD-173074		
0.639306664	-0.08725667	NVP-TAE_226		
0.194628328	-0.086286679	LOXO-101		
0.530799568	-0.08173582	PF-3758309		
0.140431955	-0.079854175	AZD6482		
0.472991973	-0.076954246	WZ-4003		
0.356788576	-0.074248299	A-770041		
0.206373557	-0.068152428	LY_333531_mesylate		
0.248656765	-0.064650282	AS-601245		
0.402360767	-0.064518705	Y-39983		
0.605494499	-0.059783585	Masitinib		
0.172926486	-0.051932044	GDC-0068		
0.543729067	-0.048170961	BGJ-398		
0.212907553	-0.047544573	MLN2480		
0.066865578	-0.047298998	Compound_71		
0.3373577	-0.042573642	DMAT		
0.027302492	-0.041771516	PH-797804		
0.488420129	-0.037026912	AZD-4547		
0.105162561	-0.032601744	CP-724714		
0.309735298	-0.02921623	GSK-269962A		
0.701206386	-0.02841191	Go_6976		
0.214747161	-0.025896877	MP-470		
0.412698269	-0.023068383	GSK690693		
0.427055031	-0.02261129	XL-184		
0.62171793	-0.022107348	GNF-5837		
0.154454619	-0.021507546	Dacomitinib_hydrate		
0.455234349	-0.018283203	KW_2449		
0.299452186	-0.01730036	Bisindolylmaleimide_I_hydrochloride		
0.346567929	-0.015655898	PLX-4720		
0.668157458	-0.009010796	XL-228		
0.094809949	-0.005957276	Chk2_Inhibitor_II		
0.098753825	-0.004349798	Binimetinib		
0.312063545	-0.001692466	BIBX_1382_dihydrochloride		
0.228271142	0.006657749	Ruboxistaurin_HCl		
0.208992481	0.007086635	EAI045		
0.407106519	0.009379625	Sorafenib		
0.06336771	0.009676054	AZD3759		
0.60943079	0.012072742	A_484954		
0.304374814	0.014755279	AZ_3146		
0.59651643	0.014847502	LY3023414		

0.34742853	0.016014934	AMG-900		
0.102783069	0.025647871	MLN-518		
0.495169938	0.027942508	A_1070722		
0.301840425	0.029120401	CP-690550		
0.226033539	0.032724202	PS1145_dihydrochloride		
0.559960485	0.036293484	PKC-412		
0.065762714	0.037146769	E-3810		
0.116518028	0.038622409	AR-12		
0.123755217	0.039080627	SB_203580		
0.526233852	0.040604256	TPCA-1		
0.176155224	0.041248851	Lavendustin_A		
0.446803361	0.047972754	GSK1070916		
0.608966112	0.050013676	Quinalizarin		
0.276858211	0.056661874	KU-55933		
0.287082225	0.058518998	ML347		
0.093808115	0.059808686	BMS_794833		
0.283685654	0.069224939	SPHINX31		
0.332614779	0.070337817	Itacitinib		
0.272003472	0.077548906	BIBF-1120		
0.531513155	0.077671722	LDN-214117		
0.155446291	0.07899709	CI-1033		
0.613916755	0.08069244	FR-180204		
0.066859752	0.08433789	CT-99021		
0.065603159	0.084622592	PD-184161		
0.150154322	0.086609125	NVP-BEZ235		
0.116163626	0.08681114	ARRY334543		
0.651416004	0.087722972	Masitinib_mesylate		
0.124263652	0.088665992	LY-2157299		
0.057258725	0.089862585	AZD6244		
0.253931105	0.094752334	Pelitinib		
0.294935793	0.096291006	KD025		
0.108398333	0.098654747	Enzastaurin		
0.557418048	0.09923251	SU_6656		
0.540439725	0.112305395	INK_128		
0.264255106	0.114270538	Toceranib		
0.165240958	0.1153078	Tyrphostin_B44		
0.479809731	0.115855753	NPS-1034		
0.428050309	0.120741993	PHA-680632		
0.196426243	0.125160336	Imatinib_Mesylate		
0.229505837	0.1266056	Aurora_A_Inhibitor_I		
0.744137168	0.12721777	PD-180970		
0.318860859	0.132587701	BAY_73-4506		
0.249563694	0.134454235	ONO-WG-307		
0.285872728	0.136290014	ST638		
0.199552834	0.141130641	GSK583		
0.048438605	0.143947005	Tucatinib		
0.420393467	0.145704225	Filgotinib		
0.488318443	0.148933753	Sunitinib_Malate		
0.56346041	0.153025642	CAN-508		
0.547358274	0.153263062	Purvalanol_A		
0.173612744	0.156676278	HMN-214		
0.499461502	0.157288745	compound_26		
0.273133218	0.157336518	WHI-P_154		
0.173949242	0.164114207	Vandetanib		
0.11171522	0.164822295	A_769662		
0.282206416	0.171726003	BYL-719		
0.617322087	0.172471717	GSK-3_inhibitor_II		
0.331935346	0.173392415	ACHP		
0.133936629	0.175823867	GDC-0941_bismesylate		
0.62668699	0.181693897	Pp242		
0.114494547	0.183774814	CHZ868		
0.057468209	0.185279101	ON-01910Na		
0.622688055	0.186379492	SJN_2511		
0.125026882	0.186663836	CAL-101		
0.18196404	0.190948099	Akt-I-1		
0.244041413	0.192613363	FPA_124		
0.195800647	0.19342722	H3B-6527		
0.21077843	0.199366748	Tirabrutinib		

0.163010463	0.202105463	ZM_323881_hydrochloride		
0.185915649	0.205434456	AMG319		
0.215774745	0.212462544	CH5424802		
0.320871651	0.213267326	BI_2536		
0.532220483	0.213539094	TBB		
0.461694479	0.214405954	TTP_22		
0.015820485	0.216189861	ABC294640		
0.140481278	0.21985504	PF_4708671		
0.414207339	0.22070612	Vactosertib		
0.075488031	0.222393408	PD-198306		
0.116307914	0.223484531	BKM120		
0.37375015	0.227561831	VX-680		
0.114513695	0.228117496	SRPIN340		
0.124469347	0.230877593	GDC-0941		
0.105820768	0.235956341	LEE011		
0.428197861	0.241238341	EW-7197		
0.069245264	0.242792591	AZD-8055		
0.092956118	0.244986206	AZD-8186		
0.068227202	0.247806191	IC87114		
0.036976807	0.253121495	KU0063794		
0.558674753	0.259863049	NSC_625987		
0.377718508	0.263611436	NMS-P715		
0.059375532	0.263944387	AZD8330		
0.118007861	0.264417678	D4476		
0.05178646	0.265201956	WAY-600		
0.436160028	0.267175317	Quercetin		
0.26737079	0.267374933	DMH1		
0.054991528	0.27880764	LY2584702_Tosylate		
0.273190588	0.282778323	GW_788388		
0.257561594	0.286314338	CGI-1746		
0.099543944	0.287276149	AMG_PERK_44		
0.469750404	0.291202724	AZ628		
0.270789057	0.291221201	AEW-541		
0.233320042	0.293198347	ML-281		
0.365032852	0.294483572	TBCA		
0.176461503	0.30290997	MPS1-IN-2		
0.085802883	0.304891229	AL_8697		
0.266096592	0.312670946	SAR407899_HCl		
0.139305338	0.318956673	CGK_733		
0.465994895	0.321100473	BAY_1895344		
0.177013338	0.3273772	GNE-7915		
0.063261405	0.330223769	SIS3		
0.419985175	0.330730736	PND-1186		
0.20511049	0.332601815	S6K-18		
0.387973249	0.342801869	BMS-2		
0.211070105	0.348846555	HG-10-102-01		
0.085698985	0.358334333	WYE-125132		
0.228410885	0.361978769	SR-3576		
0.576685667	0.366796941	U0126		
0.27330178	0.371089816	AZD-9291		
0.175563172	0.373254955	AG_490		
0.121836275	0.375606567	AV-412		
0.065899044	0.375890702	W-7_hydrochloride		
0.282536	0.377157629	SB-202190		
0.450111926	0.380552411	AR-A014418		
0.139611781	0.382077545	Sapitinib		
0.227736577	0.403020144	Sphingosine_kinase_Inhibitor_2		
0.167196557	0.403716564	ONO-4059		
0.169717953	0.41664046	RG-7842		
0.376934618	0.423790485	AZD-1480		
0.150989756	0.425760269	XMD8-92		
0.193088084	0.426702499	ABT-702_dihydrochloride		
0.257097721	0.427693248	AT7867		
0.161428109	0.428225219	AZD-5363		
0.089550003	0.428454399	Pexidartinib		
0.120720506	0.429818869	H_89_dihydrochloride		
0.384023666	0.43356663	BS-181_hydrochloride		
0.36116302	0.437634856	SD_208		

0.316375375	0.438535094	XL765		
0.345196754	0.44389528	Dasatinib		
0.529004574	0.448394835	LY-294002		
0.494786263	0.453227818	SU-6668		
0.288622499	0.462398618	AT-7519_hydrochloride		
0.22274062	0.462907344	TGX-221		
0.166497409	0.464986175	CRT0066854		
0.187037557	0.465697289	E7080		
0.361824036	0.470315278	SU_16f		
0.65496552	0.472182274	SP-600125		
0.504276335	0.473846972	ASP-3026		
0.410963476	0.476831496	5-Iodotubercidin		
0.077078938	0.479730397	Tipifarnib		
0.111912459	0.483715892	BI605906		
0.307388604	0.489358366	BI_78D3		
0.111785531	0.490151048	PV-1019		
0.119397305	0.491815418	SD-06		
0.376145244	0.494871914	AV951		
0.177473277	0.496699423	SKI-II		
0.109371185	0.497244537	Arctigenin		
0.402512223	0.501204729	Ceritinib		
0.102080286	0.503084779	Cediranib		
0.202367976	0.511460304	SMI-4a		
0.501510024	0.514423788	Alsterpaullone		
0.151988298	0.515146911	S-99		
0.169053778	0.517305851	CCT245737		
0.439065218	0.522452772	AMG-706		
0.228076816	0.52441144	BIBU_1361_dihydrochloride		
0.800270796	0.524879217	Olomoucine		
0.375475109	0.540121436	ER_27319_maleate		
0.334196419	0.545393229	Vatalanib_dihydrochloride		
0.491539568	0.552705705	PKC_theta_inhibitor		
0.400433451	0.55787015	AMG_548		
0.415852338	0.563065231	SF2523		
0.341912061	0.567144394	PP-2		
0.08962512	0.567861438	10-DEBC_hydrochloride		
0.383517087	0.573905528	NQDI-1		
0.161984682	0.573911905	Axitinib		
0.482109427	0.575099111	JNJ-10198409		
0.337519884	0.575323701	OSI-930		
0.148661554	0.580292702	AEG_3482		
0.429972589	0.58286798	MLN-1117		
0.34285906	0.584256411	PHA-793887		
0.562901855	0.586284518	TX-1918		
0.15756318	0.58829093	ML_9_hydrochloride		
0.166999072	0.592558026	Losmapimod		
0.535858393	0.600316048	SU_5416		
0.102046818	0.602498114	SB_239063		
0.130883247	0.603148937	H_1152		
0.175400212	0.606099725	AZD0530		
0.134333149	0.608477592	Rho_Kinase_Inhibitor_V		
0.249816298	0.609081507	SNS-032		
0.249799952	0.611542642	SCH900776		
0.277822822	0.611913085	Decernotinib		
0.23954469	0.612946451	AZ5104		
0.368116289	0.616081357	PF_06447475		
0.343562543	0.618441999	PCI-32765		
0.461679459	0.624188781	SU_4312		
0.195648938	0.624377847	GSK-2636771		
0.275108993	0.628425598	Kenpaullone		
0.194809347	0.632366002	Uprosertib		
0.68800211	0.639381289	TAK-901		
0.28656131	0.640514851	Apatinib_mesylate		
0.237384051	0.643301725	GSK2269557		
0.164822131	0.644528568	CX-6258_HCl		
0.283998579	0.646683753	PF-03394197		
0.237869442	0.649304032	ZM_306416_hydrochloride		
0.195623085	0.66007036	HCl		

0.42324537	0.665467739	LDN-209929_dihydrochloride		
0.110053062	0.667321563	GW583340_dihydrochloride		
0.188187927	0.668757558	Erlotinib_HCl		
0.178549647	0.676159382	PIK-294		
0.338217616	0.685017765	MAZ51		
0.152363718	0.688797712	Src_I1		
0.259257764	0.689778805	IMD_0354		
0.096523732	0.699080944	JNJ-38158471		
0.293946534	0.702103198	Ki-8751		
0.315952778	0.711367965	CGP_57380		
0.172636241	0.713748455	PIK-90		
0.528256178	0.721071243	BMS-345541		
0.299006432	0.72111398	A_83-01		
0.377557665	0.728179276	TG003		
0.222085938	0.729439616	SB-525334		
0.295106858	0.745342016	CZC_24832		
0.118293598	0.745768845	SKF-86002		
0.456475765	0.74650681	SC-514		
0.123045109	0.746790171	SKF_86002_dihydrochloride		
0.122077897	0.752494693	Afuresertib		
0.390177906	0.752591968	Genistein		
0.085060529	0.755722642	WYE-354		
0.358245611	0.757154167	BAY_57-9352		
0.290645301	0.771386743	AG-1478		
0.222045213	0.771679401	SB_431542		
0.129406154	0.779097974	Gefitinib		
0.438711226	0.782506585	CVT-313		
0.043037929	0.787427425	Mubritinib		
0.291437328	0.790884972	NVP-ADW742		
0.286638677	0.79723525	GW2974		
0.114508823	0.812269211	KRN_633		
0.653710067	0.829100609	NSC_693868		
0.094412223	0.829985619	ARQ-197		
0.223594725	0.830120027	Ryuvidine		
0.098013595	0.830661356	R_59-022		
0.49110347	0.832501173	PHA-767491_hydrochloride		
0.190171808	0.836012006	SB-505124_hydrochloride_hydrate		
0.571611345	0.843744278	GTP_14564		
0.266354769	0.847131312	LY-364947		
0.372803509	0.848678112	CID_755673		
0.27766636	0.85133791	PD-153035		
0.264237046	0.871265352	ACP-196		
0.387281686	0.876672387	LY2228820		
0.303875864	0.87919414	CGP-57380		
0.115410402	0.890674531	ZM_39923_hydrochloride		
0.38372907	0.891167939	Janex-1		
0.565858245	0.898423135	NU-2058		
0.098239124	0.902058959	W-13_hydrochloride		
0.098346397	0.920282483	MSC2530818		
0.173572645	0.929717183	BMS-582949		
0.139018357	0.932780266	BIX_02189		
0.269664705	0.934394777	RAF265		
0.167410612	0.944963813	ZM-447439		
0.094234981	0.94570756	EO-1428		
0.409214973	0.965741277	NU-7026		
0.4591645	0.994400859	AEE_788		
0.379130244	0.996837378	IC-261		
0.15923363	0.999109209	LFM-A13		
0.324687719	1.001937509	SB_216763		
0.070624843	1.002651334	XRP44X		
0.213848948	1.018298864	PHA690509		
0.053168669	1.019093156	Mk-5		
0.133461028	1.040836334	Y-27632		
0.31553334	1.049549937	PD-98059		
0.452988476	1.068839192	TC-DAPK_6		
0.209906727	1.077801228	PD-169316		
0.442750096	1.081946135	Tyrphostin_AG-1296		
0.232083976	1.09171629	Dioctanoylglycol		

0.402130902	1.103013635	BLU-554		
0.072396711	1.110839367	JX-401		
0.279158503	1.114215255	Bikinin		
0.415923834	1.122614622	DMPQ_dihydrochloride		
0.198448449	1.124701023	JNJ_28871063_hydrochloride		
0.252362102	1.127402067	NSC-663284		
0.195527181	1.1275419	SB-505124		
0.362258762	1.158845544	Chelerythrine_chloride		
0.528048158	1.159965158	Indirubin		
0.044964079	1.164615273	SB242235		
0.130884781	1.175948381	PQ401		
0.118642934	1.188962817	Lapatinib_ditosylate		
0.528862715	1.190080047	Bohemine		
0.289768338	1.191050172	Triciribine		
0.455514759	1.198472619	AG_18		
0.391508549	1.19859457	PP-1		
0.053184561	1.221295834	VX-745		
0.281337917	1.229375124	BLU9931		
0.158469513	1.231286526	PF-670462		
0.095539041	1.263664246	SL327		
0.422001123	1.264507055	Roscovitine		
0.422557145	1.300775051	1-Naphthyl_PP1		
0.338208646	1.304750443	Compound_10		
0.029368676	1.378956556	TIC10		
0.143175915	1.399492979	NH125		
0.162890166	1.416130781	Tyrphostin_SU_1498		
0.234935835	1.417016387	R1487		
0.178483397	1.469416142	HA_1100_hydrochloride		
0.196403593	1.476625443	TCS_JNK_5a		
0.180960506	1.48987627	Rho_Kinase_Inhibitor_II		
0.238532633	1.491795063	Fasudil_HCl		
0.495842516	1.542924881	PF-06651600		
0.274467915	1.546026707	IC86621		
0.227276027	1.644542098	Necrostatin-1		
0.200753883	1.746892691	C-1		
0.548345387	2.166034222	FAK_Inhibitor_14		
0.250114769	2.712764263	SD_169		
0.203555807	3.361917496	DCA		

Table S2. Bolz2 analysis output for pyrimidine analogs (associated with **Table 2)**

compound	affinity_pred_value	affinity_probability_binary	SMILES	pIC50 boltz (kcal/mol)	IC50 eurofins (nM)
5a	-0.93	0.22	CC(C)(C1=NC(C2=C(F)C([N-]S(=O)(C3=C(F)C=CC=C3F)=O)=CC=C2)=C(C4=NC(N)=NC(C)=C4)S1)C	9.457939029	263
5b	-0.82	0.26	CC(C)(C1=NC(C2=C(F)C([N-]S(=O)(C3=C(F)C=CC=C3F)=O)=CC=C2)=C(C4=NC(N)=NC(N)=C4)S1)C	9.304901289	213
5c	-0.97	0.13	CC(C)(C1=NC(C2=C(F)C([N-]S(=O)(C3=C(F)C=CC=C3F)=O)=CC=C2)=C(C4=NC(N)=NC(CC)=C4)S1)C	9.506295812	2104
5d	-0.32	0.07	CC(C)(C1=NC(C2=C(F)C([N-]S(=O)(C3=C(F)C=CC=C3F)=O)=CC=C2)=C(C4=NC(N)=NC(OC)=C4)S1)C	8.620079503	1964
5e	-1.04	0.62	CC(C)(C1=NC(C2=C(F)C([N-]S(=O)(C3=C(F)C=CC=C3F)=O)=CC=C2)=C(C4=NC(N)=NC=C4F)S1)C	9.6038407 6	69
5f	-0.85	0.23	CC(C)(C1=NC(C2=C(F)C([N-]S(=O)(C3=C(F)C=CC=C3F)=O)=CC=C2)=C(C4=NC(NC)=NC(C)=C4)S1)C	9.338525484	299

Table S3. Bolz2 analysis output for sulfonamide analog screen (associated with **Figure 3**).

ID	affinity_pred_value (lo	affinity_probability_binary	SMILES	Descriptor	pIC50 boltz (kcal/mol)	compound	IC50 eurofins (nM)
A001	-1.495148182	0.735411763	CC(C)(C)c1nc(-c2cccc([N-]S(=O)(=O)c3cccc(F)c3F)c2F)c(-c2ccnc(N)n2)s1	2F-3F	10.22		
A002	-1.076559186	0.582701921	CC(C)(C)c1nc(-c2cccc([N-]S(=O)(=O)c3cccc(Cl)c3F)c2F)c(-c2ccnc(N)n2)s1	2F-3Cl	9.65		
A003	-1.149311662	0.552250803	CC(C)(C)c1nc(-c2cccc([N-]S(=O)(=O)c3cccc(Br)c3F)c2F)c(-c2ccnc(N)n2)s1	2F-3Br	9.75		
A004	-0.537397027	0.354960561	CC(C)(C)c1nc(-c2cccc([N-]S(=O)(=O)c3cccc(C(F)(F)F)c3F)c2F)c(-c2ccnc(N)n2)s1	2F-3CF3	8.92		
A005	-0.742129683	0.532051444	Cc1cccc(S(=O)(=O)[N-]c2cccc(-c3nc(C(C)(C)C)sc3-c3ccnc(N)n3)c2F)c1F	2F-3Me	9.2		
A006	-0.992483616	0.609434485	CCc1cccc(S(=O)(=O)[N-]c2cccc(-c3nc(C(C)(C)C)sc3-c3ccnc(N)n3)c2F)c1F	2F-3Et	9.54		
A007	-0.774189353	0.483126909	COc1cccc(S(=O)(=O)[N-]c2cccc(-c3nc(C(C)(C)C)sc3-c3ccnc(N)n3)c2F)c1F	2F-3OMe	9.24		
A008	-1.21592164	0.60774231	CC(C)(C)c1nc(-c2cccc([N-]S(=O)(=O)c3ccc(F)cc3F)c2F)c(-c2ccnc(N)n2)s1	2F-4F	9.84		
A009	-1.090446591	0.547375917	CC(C)(C)c1nc(-c2cccc([N-]S(=O)(=O)c3ccc(Cl)cc3F)c2F)c(-c2ccnc(N)n2)s1	2F-4Cl	9.67		
A010	-1.133425951	0.5457322	CC(C)(C)c1nc(-c2cccc([N-]S(=O)(=O)c3ccc(Br)cc3F)c2F)c(-c2ccnc(N)n2)s1	2F-4Br	9.73		
A011	0.114281736	0.212409526	CC(C)(C)c1nc(-c2cccc([N-]S(=O)(=O)c3ccc(C(F)(F)F)cc3F)c2F)c(-c2ccnc(N)n2)s1	2F-4CF3	8.03		
A012	-0.081893861	0.401737213	Cc1ccc(S(=O)(=O)[N-]c2cccc(-c3nc(C(C)(C)C)sc3-c3ccnc(N)n3)c2F)c(F)c1	2F-4Me	8.3		
A013	-0.784397006	0.416773677	CCc1ccc(S(=O)(=O)[N-]c2cccc(-c3nc(C(C)(C)C)sc3-c3ccnc(N)n3)c2F)c(F)c1	2F-4Et	9.25		
A014	-0.497461975	0.442068189	COc1ccc(S(=O)(=O)[N-]c2cccc(-c3nc(C(C)(C)C)sc3-c3ccnc(N)n3)c2F)c(F)c1	2F-4OMe	8.86		
A015	-1.503505111	0.733935475	CC(C)(C)c1nc(-c2cccc([N-]S(=O)(=O)c3cc(F)ccc3F)c2F)c(-c2ccnc(N)n2)s1	2F-5F	10.23	9b	35
A016	-1.00896728	0.545364559	CC(C)(C)c1nc(-c2cccc([N-]S(=O)(=O)c3cc(Cl)ccc3F)c2F)c(-c2ccnc(N)n2)s1	2F-5Cl	9.56		
A017	-1.577356339	0.746534169	CC(C)(C)c1nc(-c2cccc([N-]S(=O)(=O)c3cc(Br)ccc3F)c2F)c(-c2ccnc(N)n2)s1	2F-5Br	10.34		
A018	-0.319038808	0.337919742	CC(C)(C)c1nc(-c2cccc([N-]S(=O)(=O)c3cc(C(F)(F)F)ccc3F)c2F)c(-c2ccnc(N)n2)s1	2F-5CF3	8.62		
A019	-1.150054455	0.693563461	Cc1ccc(F)c(S(=O)(=O)[N-]c2cccc(-c3nc(C(C)(C)C)sc3-c3ccnc(N)n3)c2F)c1	2F-5Me	9.75		
A020	-1.076941252	0.587635875	CCc1ccc(F)c(S(=O)(=O)[N-]c2cccc(-c3nc(C(C)(C)C)sc3-c3ccnc(N)n3)c2F)c1	2F-5Et	9.65		
A021	-1.327483416	0.577862263	COc1ccc(F)c(S(=O)(=O)[N-]c2cccc(-c3nc(C(C)(C)C)sc3-c3ccnc(N)n3)c2F)c1	2F-5OMe	9.99		
A022	-1.401934743	0.800259829	CC(C)(C)c1nc(-c2cccc([N-]S(=O)(=O)c3c(F)cccc3F)c2F)c(-c2ccnc(N)n2)s1	2F-6F	10.1	dabrafenib	69
A023	-1.291791677	0.777049661	CC(C)(C)c1nc(-c2cccc([N-]S(=O)(=O)c3c(F)cccc3Cl)c2F)c(-c2ccnc(N)n2)s1	2F-6Cl	9.95	9a	185
A024	-1.370838881	0.776632309	CC(C)(C)c1nc(-c2cccc([N-]S(=O)(=O)c3c(F)cccc3Br)c2F)c(-c2ccnc(N)n2)s1	2F-6Br	10.05		
A025	-0.488405943	0.563205719	CC(C)(C)c1nc(-c2cccc([N-]S(=O)(=O)c3c(F)cccc3C(F)(F)F)c2F)c(-c2ccnc(N)n2)s1	2F-6CF3	8.85		
A026	-0.461772263	0.484978676	Cc1cccc(F)c1S(=O)(=O)[N-]c1cccc(-c2nc(C(C)(C)C)sc2-c2ccnc(N)n2)c1F	2F-6Me	8.81		
A027	-0.785022676	0.599951625	CCc1cccc(F)c1S(=O)(=O)[N-]c1cccc(-c2nc(C(C)(C)C)sc2-c2ccnc(N)n2)c1F	2F-6Et	9.25		
A028	-1.239548802	0.654884577	COc1cccc(F)c1S(=O)(=O)[N-]c1cccc(-c2nc(C(C)(C)C)sc2-c2ccnc(N)n2)c1F	2F-6OMe	9.87		
A029	-1.06352222	0.581643045	CC(C)(C)c1nc(-c2cccc([N-]S(=O)(=O)c3ccc(F)c(F)c3F)c2F)c(-c2ccnc(N)n2)s1	2F-3F-4F	9.63		
A030	-0.927357197	0.479336143	CC(C)(C)c1nc(-c2cccc([N-]S(=O)(=O)c3ccc(Cl)c(F)c3F)c2F)c(-c2ccnc(N)n2)s1	2F-3F-4Cl	9.45		
A031	-0.961951494	0.491663724	CC(C)(C)c1nc(-c2cccc([N-]S(=O)(=O)c3ccc(Br)c(F)c3F)c2F)c(-c2ccnc(N)n2)s1	2F-3F-4Br	9.5		
A032	-0.157058701	0.215427324	CC(C)(C)c1nc(-c2cccc([N-]S(=O)(=O)c3ccc(C(F)(F)F)c(F)c3F)c2F)c(-c2ccnc(N)n2)s1	2F-3F-4CF3	8.4		
A033	-0.873646617	0.450606674	Cc1ccc(S(=O)(=O)[N-]c2cccc(-c3nc(C(C)(C)C)sc3-c3ccnc(N)n3)c2F)c(F)c1F	2F-3F-4Me	9.38		
A034	-0.794319034	0.318903059	CCc1ccc(S(=O)(=O)[N-]c2cccc(-c3nc(C(C)(C)C)sc3-c3ccnc(N)n3)c2F)c(F)c1F	2F-3F-4Et	9.27		
A035	-1.099293232	0.45273596	COc1ccc(S(=O)(=O)[N-]c2cccc(-c3nc(C(C)(C)C)sc3-c3ccnc(N)n3)c2F)c(F)c1F	2F-3F-4OMe	9.68		
A036	-0.706719279	0.467824578	CC(C)(C)c1nc(-c2cccc([N-]S(=O)(=O)c3ccc(F)c(Cl)c3F)c2F)c(-c2ccnc(N)n2)s1	2F-3Cl-4F	9.15		
A037	-0.870410085	0.550555468	CC(C)(C)c1nc(-c2cccc([N-]S(=O)(=O)c3ccc(Cl)c(Cl)c3F)c2F)c(-c2ccnc(N)n2)s1	2F-3Cl-4Cl	9.37		
A038	-0.885684609	0.395464897	CC(C)(C)c1nc(-c2cccc([N-]S(=O)(=O)c3ccc(Br)c(Cl)c3F)c2F)c(-c2ccnc(N)n2)s1	2F-3Cl-4Br	9.39		
A039	-0.556372702	0.118499771	CC(C)(C)c1nc(-c2cccc([N-]S(=O)(=O)c3ccc(C(F)(F)F)c(Cl)c3F)c2F)c(-c2ccnc(N)n2)s1	2F-3Cl-4CF3	8.94		
A040	-0.614824653	0.297089219	Cc1ccc(S(=O)(=O)[N-]c2cccc(-c3nc(C(C)(C)C)sc3-c3ccnc(N)n3)c2F)c(F)c1Cl	2F-3Cl-4Me	9.02		
A041	-0.652378321	0.31707108	CCc1ccc(S(=O)(=O)[N-]c2cccc(-c3nc(C(C)(C)C)sc3-c3ccnc(N)n3)c2F)c(F)c1Cl	2F-3Cl-4Et	9.07		
A042	-1.049124956	0.385416389	COc1ccc(S(=O)(=O)[N-]c2cccc(-c3nc(C(C)(C)C)sc3-c3ccnc(N)n3)c2F)c(F)c1Cl	2F-3Cl-4OMe	9.62		
A043	-0.656137705	0.406322002	CC(C)(C)c1nc(-c2cccc([N-]S(=O)(=O)c3ccc(F)c(Br)c3F)c2F)c(-c2ccnc(N)n2)s1	2F-3Br-4F	9.08		
A044	-1.004381299	0.445243359	CC(C)(C)c1nc(-c2cccc([N-]S(=O)(=O)c3ccc(Cl)c(Br)c3F)c2F)c(-c2ccnc(N)n2)s1	2F-3Br-4Cl	9.55		
A045	-0.972573757	0.419706464	CC(C)(C)c1nc(-c2cccc([N-]S(=O)(=O)c3ccc(Br)c(Br)c3F)c2F)c(-c2ccnc(N)n2)s1	2F-3Br-4Br	9.51		
A046	-0.338314235	0.135568172	CC(C)(C)c1nc(-c2cccc([N-]S(=O)(=O)c3ccc(C(F)(F)F)c(Br)c3F)c2F)c(-c2ccnc(N)n2)s1	2F-3Br-4CF3	8.65		
A047	-1.138514757	0.361331642	Cc1ccc(S(=O)(=O)[N-]c2cccc(-c3nc(C(C)(C)C)sc3-c3ccnc(N)n3)c2F)c(F)c1Br	2F-3Br-4Me	9.74		
A048	-0.489100575	0.275544912	CCc1ccc(S(=O)(=O)[N-]c2cccc(-c3nc(C(C)(C)C)sc3-c3ccnc(N)n3)c2F)c(F)c1Br	2F-3Br-4Et	8.85		
A049	-0.864487052	0.286143064	COc1ccc(S(=O)(=O)[N-]c2cccc(-c3nc(C(C)(C)C)sc3-c3ccnc(N)n3)c2F)c(F)c1Br	2F-3Br-4OMe	9.36		
A050	-0.825785637	0.409435362	CC(C)(C)c1nc(-c2cccc([N-]S(=O)(=O)c3ccc(F)c(C(F)(F)F)c3F)c2F)c(-c2ccnc(N)n2)s1	2F-3CF3-4F	9.31		
A051	-0.804602981	0.281129956	CC(C)(C)c1nc(-c2cccc([N-]S(=O)(=O)c3ccc(Cl)c(C(F)(F)F)c3F)c2F)c(-c2ccnc(N)n2)s1	2F-3CF3-4Cl	9.28		
A052	-0.760140896	0.363025606	CC(C)(C)c1nc(-c2cccc([N-]S(=O)(=O)c3ccc(Br)c(C(F)(F)F)c3F)c2F)c(-c2ccnc(N)n2)s1	2F-3CF3-4Br	9.22		
A053	-0.706435204	0.269232124	CC(C)(C)c1nc(-c2cccc([N-]S(=O)(=O)c3ccc(C(F)(F)F)c(C(F)(F)F)c3F)c2F)c(-c2ccnc(N)n2)s1	2F-3CF3-4CF3	9.15		
A054	-0.809100986	0.334760845	Cc1ccc(S(=O)(=O)[N-]c2cccc(-c3nc(C(C)(C)C)sc3-c3ccnc(N)n3)c2F)c(F)c1C(F)(F)F	2F-3CF3-4Me	9.29		
A055	-1.103084087	0.224183381	CCc1ccc(S(=O)(=O)[N-]c2cccc(-c3nc(C(C)(C)C)sc3-c3ccnc(N)n3)c2F)c(F)c1C(F)(F)F	2F-3CF3-4Et	9.69		
A056	-0.701580465	0.27367112	COc1ccc(S(=O)(=O)[N-]c2cccc(-c3nc(C(C)(C)C)sc3-c3ccnc(N)n3)c2F)c(F)c1C(F)(F)F	2F-3CF3-4OMe	9.14		
A057	-0.904479444	0.552214324	Cc1c(F)ccc(S(=O)(=O)[N-]c2cccc(-c3nc(C(C)(C)C)sc3-c3ccnc(N)n3)c2F)c1F	2F-3Me-4F	9.42		
A058	-1.224074364	0.567011118	Cc1c(Cl)ccc(S(=O)(=O)[N-]c2cccc(-c3nc(C(C)(C)C)sc3-c3ccnc(N)n3)c2F)c1F	2F-3Me-4Cl	9.85		
A059	-1.182054996	0.530608952	Cc1c(Br)ccc(S(=O)(=O)[N-]c2cccc(-c3nc(C(C)(C)C)sc3-c3ccnc(N)n3)c2F)c1F	2F-3Me-4Br	9.8		
A060	-0.453763425	0.286634982	Cc1c(C(F)(F)F)ccc(S(=O)(=O)[N-]c2cccc(-c3nc(C(C)(C)C)sc3-c3ccnc(N)n3)c2F)c1F	2F-3Me-4CF3	8.8		
A061	-0.763198018	0.576549411	Cc1ccc(S(=O)(=O)[N-]c2cccc(-c3nc(C(C)(C)C)sc3-c3ccnc(N)n3)c2F)c(F)c1C	2F-3Me-4Me	9.23		
A062	-0.861488879	0.290092528	CCc1ccc(S(=O)(=O)[N-]c2cccc(-c3nc(C(C)(C)C)sc3-c3ccnc(N)n3)c2F)c(F)c1C	2F-3Me-4Et	9.36		
A063	-0.797693014	0.338488758	COc1ccc(S(=O)(=O)[N-]c2cccc(-c3nc(C(C)(C)C)sc3-c3ccnc(N)n3)c2F)c(F)c1C	2F-3Me-4OMe	9.27		
A064	-1.029104829	0.524954498	CCc1c(F)ccc(S(=O)(=O)[N-]c2cccc(-c3nc(C(C)(C)C)sc3-c3ccnc(N)n3)c2F)c1F	2F-3Et-4F	9.59		
A065	-0.825870156	0.440554798	CCc1c(Cl)ccc(S(=O)(=O)[N-]c2cccc(-c3nc(C(C)(C)C)sc3-c3ccnc(N)n3)c2F)c1F	2F-3Et-4Cl	9.31		
A066	-0.694270909	0.361435473	CCc1c(Br)ccc(S(=O)(=O)[N-]c2cccc(-c3nc(C(C)(C)C)sc3-c3ccnc(N)n3)c2F)c1F	2F-3Et-4Br	9.13		
A067	-0.330830991	0.141627789	CCc1c(C(F)(F)F)ccc(S(=O)(=O)[N-]c2cccc(-c3nc(C(C)(C)C)sc3-c3ccnc(N)n3)c2F)c1F	2F-3Et-4CF3	8.64		
A068	-0.622758925	0.346809447	CCc1c(C)ccc(S(=O)(=O)[N-]c2cccc(-c3nc(C(C)(C)C)sc3-c3ccnc(N)n3)c2F)c1F	2F-3Et-4Me	9.03		
A069	-0.638378382	0.250673294	CCc1ccc(S(=O)(=O)[N-]c2cccc(-c3nc(C(C)(C)C)sc3-c3ccnc(N)n3)c2F)c(F)c1CC	2F-3Et-4Et	9.05		
A070	-1.005127192	0.272807002	CCc1c(OC)ccc(S(=O)(=O)[N-]c2cccc(-c3nc(C(C)(C)C)sc3-c3ccnc(N)n3)c2F)c1F	2F-3Et-4OMe	9.55		
A071	-1.407886028	0.525620341	COc1c(F)ccc(S(=O)(=O)[N-]c2cccc(-c3nc(C(C)(C)C)sc3-c3ccnc(N)n3)c2F)c1F	2F-3OMe-4F	10.1		
A072	-1.100120544	0.452628613	COc1c(Cl)ccc(S(=O)(=O)[N-]c2cccc(-c3nc(C(C)(C)C)sc3-c3ccnc(N)n3)c2F)c1F	2F-3OMe-4Cl	9.68		
A073	-1.042179823	0.382939816	COc1c(Br)ccc(S(=O)(=O)[N-]c2cccc(-c3nc(C(C)(C)C)sc3-c3ccnc(N)n3)c2F)c1F	2F-3OMe-4Br	9.61		
A074	-0.50889194	0.144965231	COc1c(C(F)(F)F)ccc(S(=O)(=O)[N-]c2cccc(-c3nc(C(C)(C)C)sc3-c3ccnc(N)n3)c2F)c1F	2F-3OMe-4CF3	8.88		
A075	-0.789404035	0.407298833	COc1c(C)ccc(S(=O)(=O)[N-]c2cccc(-c3nc(C(C)(C)C)sc3-c3ccnc(N)n3)c2F)c1F	2F-3OMe-4Me	9.26		
A076	-1.110441923	0.346925855	CCc1ccc(S(=O)(=O)[N-]c2cccc(-c3nc(C(C)(C)C)sc3-c3ccnc(N)n3)c2F)c(F)c1OC	2F-3OMe-4Et	9.7		
A077	-0.799808621	0.314991206	COc1ccc(S(=O)(=O)[N-]c2cccc(-c3nc(C(C)(C)C)sc3-c3ccnc(N)n3)c2F)c(F)c1OC	2F-3OMe-4OMe	9.27		
A078	-1.403156281	0.68313539	CC(C)(C)c1nc(-c2cccc([N-]S(=O)(=O)c3cc(F)cc(F)c3F)c2F)c(-c2ccnc(N)n2)s1	2F-3F-5F	10.1		
A079	-1.779778361	0.759401083	CC(C)(C)c1nc(-c2cccc([N-]S(=O)(=O)c3cc(Cl)cc(F)c3F)c2F)c(-c2ccnc(N)n2)s1	2F-3F-5Cl	10.61		
A080	-1.754333019	0.799429417	CC(C)(C)c1nc(-c2cccc([N-]S(=O)(=O)c3cc(Br)cc(F)c3F)c2F)c(-c2ccnc(N)n2)s1	2F-3F-5Br	10.58		
A081	-0.662728131	0.447480142	CC(C)(C)c1nc(-c2cccc([N-]S(=O)(=O)c3cc(C(F)(F)F)cc(F)c3F)c2F)c(-c2ccnc(N)n2)s1	2F-3F-5CF3	9.09		
A082	-1.338192701	0.6658355	Cc1cc(F)c(F)c(S(=O)(=O)[N-]c2cccc(-c3nc(C(C)(C)C)sc3-c3ccnc(N)n3)c2F)c1	2F-3F-5Me	10.01	9c	58
A083	-1.103663683	0.611890197	CCc1cc(F)c(F)c(S(=O)(=O)[N-]c2cccc(-c3nc(C(C)(C)C)sc3-c3ccnc(N)n3)c2F)c1	2F-3F-5Et	9.69		

A084	-1.579684496	0.732805967	COc1cc(F)c(F)c(S(=O)(=O)[N-]c2cccc(-c3nc(C(C)(C)C)sc3-c3ccnc(N)n3)c2F)c1	2F-3F-5OMe	10.34		
A085	-1.198202133	0.61806941	CC(C)(C)c1nc(-c2cccc([N-]S(=O)(=O)c3cc(F)cc(Cl)c3F)c2F)c(-c2ccnc(N)n2)s1	2F-3Cl-5F	9.82		
A086	-1.451578856	0.587033391	CC(C)(C)c1nc(-c2cccc([N-]S(=O)(=O)c3cc(Cl)cc(Cl)c3F)c2F)c(-c2ccnc(N)n2)s1	2F-3Cl-5Cl	10.16	9d	898
A087	-1.462189674	0.605333924	CC(C)(C)c1nc(-c2cccc([N-]S(=O)(=O)c3cc(Br)cc(Cl)c3F)c2F)c(-c2ccnc(N)n2)s1	2F-3Cl-5Br	10.18		
A088	-0.695687294	0.426521391	CC(C)(C)c1nc(-c2cccc([N-]S(=O)(=O)c3cc(C(F)(F)F)cc(Cl)c3F)c2F)c(-c2ccnc(N)n2)s1	2F-3Cl-5CF3	9.13		
A089	-0.784050465	0.395473242	Cc1cc(Cl)c(F)c(S(=O)(=O)[N-]c2cccc(-c3nc(C(C)(C)C)sc3-c3ccnc(N)n3)c2F)c1	2F-3Cl-5Me	9.25		
A090	-0.776134491	0.501787543	CCc1cc(Cl)c(F)c(S(=O)(=O)[N-]c2cccc(-c3nc(C(C)(C)C)sc3-c3ccnc(N)n3)c2F)c1	2F-3Cl-5Et	9.24		
A091	-1.001859188	0.402052522	COc1cc(Cl)c(F)c(S(=O)(=O)[N-]c2cccc(-c3nc(C(C)(C)C)sc3-c3ccnc(N)n3)c2F)c1	2F-3Cl-5OMe	9.55		
A092	-1.141290188	0.588999867	CC(C)(C)c1nc(-c2cccc([N-]S(=O)(=O)c3cc(F)cc(Br)c3F)c2F)c(-c2ccnc(N)n2)s1	2F-3Br-5F	9.74		
A093	-1.352759838	0.568653047	CC(C)(C)c1nc(-c2cccc([N-]S(=O)(=O)c3cc(Cl)cc(Br)c3F)c2F)c(-c2ccnc(N)n2)s1	2F-3Br-5Cl	10.03		
A094	-1.464152813	0.575739443	CC(C)(C)c1nc(-c2cccc([N-]S(=O)(=O)c3cc(Br)cc(Br)c3F)c2F)c(-c2ccnc(N)n2)s1	2F-3Br-5Br	10.18		
A095	-0.793917894	0.487460852	CC(C)(C)c1nc(-c2cccc([N-]S(=O)(=O)c3cc(C(F)(F)F)cc(Br)c3F)c2F)c(-c2ccnc(N)n2)s1	2F-3Br-5CF3	9.27		
A096	-0.613860667	0.308021128	Cc1cc(Br)c(F)c(S(=O)(=O)[N-]c2cccc(-c3nc(C(C)(C)C)sc3-c3ccnc(N)n3)c2F)c1	2F-3Br-5Me	9.02		
A097	-0.882211685	0.42010355	CCc1cc(Br)c(F)c(S(=O)(=O)[N-]c2cccc(-c3nc(C(C)(C)C)sc3-c3ccnc(N)n3)c2F)c1	2F-3Br-5Et	9.39		
A098	-1.306416512	0.410043597	COc1cc(Br)c(F)c(S(=O)(=O)[N-]c2cccc(-c3nc(C(C)(C)C)sc3-c3ccnc(N)n3)c2F)c1	2F-3Br-5OMe	9.97		
A099	-1.152503014	0.559488475	CC(C)(C)c1nc(-c2cccc([N-]S(=O)(=O)c3cc(F)cc(C(F)(F)F)c3F)c2F)c(-c2ccnc(N)n2)s1	2F-3CF3-5F	9.76		
A100	-1.121334553	0.604299664	CC(C)(C)c1nc(-c2cccc([N-]S(=O)(=O)c3cc(Cl)cc(C(F)(F)F)c3F)c2F)c(-c2ccnc(N)n2)s1	2F-3CF3-5Cl	9.71		
A101	-0.832872033	0.320093602	CC(C)(C)c1nc(-c2cccc([N-]S(=O)(=O)c3cc(Br)cc(C(F)(F)F)c3F)c2F)c(-c2ccnc(N)n2)s1	2F-3CF3-5Br	9.32		
A102	-0.838267803	0.413778394	CC(C)(C)c1nc(-c2cccc([N-]S(=O)(=O)c3cc(C(F)(F)F)cc(C(F)(F)F)c3F)c2F)c(-c2ccnc(N)n2)s1	2F-3CF3-5CF3	9.33		
A103	-0.636785388	0.239911497	Cc1cc(C(F)(F)F)c(F)c(S(=O)(=O)[N-]c2cccc(-c3nc(C(C)(C)C)sc3-c3ccnc(N)n3)c2F)c1	2F-3CF3-5Me	9.05		
A104	-0.696093976	0.212091893	CCc1cc(C(F)(F)F)c(F)c(S(=O)(=O)[N-]c2cccc(-c3nc(C(C)(C)C)sc3-c3ccnc(N)n3)c2F)c1	2F-3CF3-5Et	9.13		
A105	-1.047357202	0.297579348	COc1cc(C(F)(F)F)c(F)c(S(=O)(=O)[N-]c2cccc(-c3nc(C(C)(C)C)sc3-c3ccnc(N)n3)c2F)c1	2F-3CF3-5OMe	9.61		
A106	-1.153235674	0.673700809	Cc1cc(F)cc(S(=O)(=O)[N-]c2cccc(-c3nc(C(C)(C)C)sc3-c3ccnc(N)n3)c2F)c1F	2F-3Me-5F	9.76		
A107	-1.235835552	0.679353237	Cc1cc(Cl)cc(S(=O)(=O)[N-]c2cccc(-c3nc(C(C)(C)C)sc3-c3ccnc(N)n3)c2F)c1F	2F-3Me-5Cl	9.87		
A108	-1.136385798	0.55562067	Cc1cc(Br)cc(S(=O)(=O)[N-]c2cccc(-c3nc(C(C)(C)C)sc3-c3ccnc(N)n3)c2F)c1F	2F-3Me-5Br	9.73		
A109	-0.640014708	0.28649646	Cc1cc(C(F)(F)F)cc(S(=O)(=O)[N-]c2cccc(-c3nc(C(C)(C)C)sc3-c3ccnc(N)n3)c2F)c1F	2F-3Me-5CF3	9.06		
A110	-1.206164002	0.660642982	Cc1cc(C)c(F)c(S(=O)(=O)[N-]c2cccc(-c3nc(C(C)(C)C)sc3-c3ccnc(N)n3)c2F)c1	2F-3Me-5Me	9.83		
A111	-0.929507852	0.411137462	CCc1cc(C)c(F)c(S(=O)(=O)[N-]c2cccc(-c3nc(C(C)(C)C)sc3-c3ccnc(N)n3)c2F)c1	2F-3Me-5Et	9.45		
A112	-1.002806783	0.52810061	COc1cc(C)c(F)c(S(=O)(=O)[N-]c2cccc(-c3nc(C(C)(C)C)sc3-c3ccnc(N)n3)c2F)c1	2F-3Me-5OMe	9.55		
A113	-1.284322739	0.616715848	CCc1cc(F)cc(S(=O)(=O)[N-]c2cccc(-c3nc(C(C)(C)C)sc3-c3ccnc(N)n3)c2F)c1F	2F-3Et-5F	9.94		
A114	-1.167068601	0.591659904	CCc1cc(Cl)cc(S(=O)(=O)[N-]c2cccc(-c3nc(C(C)(C)C)sc3-c3ccnc(N)n3)c2F)c1F	2F-3Et-5Cl	9.78		
A115	-1.122275591	0.53807956	CCc1cc(Br)cc(S(=O)(=O)[N-]c2cccc(-c3nc(C(C)(C)C)sc3-c3ccnc(N)n3)c2F)c1F	2F-3Et-5Br	9.71		
A116	-1.418847084	0.280379117	CCc1cc(C(F)(F)F)cc(S(=O)(=O)[N-]c2cccc(-c3nc(C(C)(C)C)sc3-c3ccnc(N)n3)c2F)c1F	2F-3Et-5CF3	10.12		
A117	-1.204654932	0.434143811	CCc1cc(C)cc(S(=O)(=O)[N-]c2cccc(-c3nc(C(C)(C)C)sc3-c3ccnc(N)n3)c2F)c1F	2F-3Et-5Me	9.83		
A118	-1.027246833	0.2917597	CCc1cc(CC)c(F)c(S(=O)(=O)[N-]c2cccc(-c3nc(C(C)(C)C)sc3-c3ccnc(N)n3)c2F)c1	2F-3Et-5Et	9.59		
A119	-0.946818709	0.436551869	CCc1cc(OC)cc(S(=O)(=O)[N-]c2cccc(-c3nc(C(C)(C)C)sc3-c3ccnc(N)n3)c2F)c1F	2F-3Et-5OMe	9.48		
A120	-1.074715614	0.490465194	COc1cc(F)cc(S(=O)(=O)[N-]c2cccc(-c3nc(C(C)(C)C)sc3-c3ccnc(N)n3)c2F)c1F	2F-3OMe-5F	9.65		
A121	-1.265473366	0.547715247	COc1cc(Cl)cc(S(=O)(=O)[N-]c2cccc(-c3nc(C(C)(C)C)sc3-c3ccnc(N)n3)c2F)c1F	2F-3OMe-5Cl	9.91		
A122	-0.965656996	0.511561096	COc1cc(Br)cc(S(=O)(=O)[N-]c2cccc(-c3nc(C(C)(C)C)sc3-c3ccnc(N)n3)c2F)c1F	2F-3OMe-5Br	9.5		
A123	-0.987179637	0.241388068	COc1cc(C(F)(F)F)cc(S(=O)(=O)[N-]c2cccc(-c3nc(C(C)(C)C)sc3-c3ccnc(N)n3)c2F)c1F	2F-3OMe-5CF3	9.53		
A124	-1.209252954	0.43851772	COc1cc(C)cc(S(=O)(=O)[N-]c2cccc(-c3nc(C(C)(C)C)sc3-c3ccnc(N)n3)c2F)c1F	2F-3OMe-5Me	9.83		
A125	-1.263002634	0.376150072	CCc1cc(OC)c(F)c(S(=O)(=O)[N-]c2cccc(-c3nc(C(C)(C)C)sc3-c3ccnc(N)n3)c2F)c1	2F-3OMe-5Et	9.91		
A126	-1.020012975	0.421153873	COc1cc(OC)c(F)c(S(=O)(=O)[N-]c2cccc(-c3nc(C(C)(C)C)sc3-c3ccnc(N)n3)c2F)c1	2F-3OMe-5OMe	9.58		
A127	-1.706237912	0.809937358	CC(C)(C)c1nc(-c2cccc([N-]S(=O)(=O)c3c(F)ccc(F)c3F)c2F)c(-c2ccnc(N)n2)s1	2F-3F-6F	10.51		
A128	-1.776303411	0.850259423	CC(C)(C)c1nc(-c2cccc([N-]S(=O)(=O)c3c(Cl)ccc(F)c3F)c2F)c(-c2ccnc(N)n2)s1	2F-3F-6Cl	10.61		
A129	-1.729747295	0.802071214	CC(C)(C)c1nc(-c2cccc([N-]S(=O)(=O)c3c(Br)ccc(F)c3F)c2F)c(-c2ccnc(N)n2)s1	2F-3F-6Br	10.54		
A130	-0.996005893	0.662358284	CC(C)(C)c1nc(-c2cccc([N-]S(=O)(=O)c3c(C(F)(F)F)ccc(F)c3F)c2F)c(-c2ccnc(N)n2)s1	2F-3F-6CF3	9.54		
A131	-1.039642572	0.575599909	Cc1ccc(F)c(F)c1S(=O)(=O)[N-]c1cccc(-c2nc(C(C)(C)C)sc2-c2ccnc(N)n2)c1F	2F-3F-6Me	9.6		
A132	-1.557974339	0.812298417	CCc1ccc(F)c(F)c1S(=O)(=O)[N-]c1cccc(-c2nc(C(C)(C)C)sc2-c2ccnc(N)n2)c1F	2F-3F-6Et	10.31		
A133	-1.639392138	0.713801384	COc1ccc(F)c(F)c1S(=O)(=O)[N-]c1cccc(-c2nc(C(C)(C)C)sc2-c2ccnc(N)n2)c1F	2F-3F-6OMe	10.42		
A134	-1.576071739	0.759091496	CC(C)(C)c1nc(-c2cccc([N-]S(=O)(=O)c3c(F)ccc(Cl)c3F)c2F)c(-c2ccnc(N)n2)s1	2F-3Cl-6F	10.33		
A135	-1.453864932	0.706815004	CC(C)(C)c1nc(-c2cccc([N-]S(=O)(=O)c3c(Cl)ccc(Cl)c3F)c2F)c(-c2ccnc(N)n2)s1	2F-3Cl-6Cl	10.17		
A136	-1.362474918	0.699335873	CC(C)(C)c1nc(-c2cccc([N-]S(=O)(=O)c3c(Br)ccc(Cl)c3F)c2F)c(-c2ccnc(N)n2)s1	2F-3Cl-6Br	10.04		
A137	-0.612535775	0.52138114	CC(C)(C)c1nc(-c2cccc([N-]S(=O)(=O)c3c(C(F)(F)F)ccc(Cl)c3F)c2F)c(-c2ccnc(N)n2)s1	2F-3Cl-6CF3	9.02		
A138	-0.652359962	0.426391095	Cc1ccc(Cl)c(F)c1S(=O)(=O)[N-]c1cccc(-c2nc(C(C)(C)C)sc2-c2ccnc(N)n2)c1F	2F-3Cl-6Me	9.07		
A139	-0.650312662	0.443112195	CCc1ccc(Cl)c(F)c1S(=O)(=O)[N-]c1cccc(-c2nc(C(C)(C)C)sc2-c2ccnc(N)n2)c1F	2F-3Cl-6Et	9.07		
A140	-0.843695641	0.278479576	COc1ccc(Cl)c(F)c1S(=O)(=O)[N-]c1cccc(-c2nc(C(C)(C)C)sc2-c2ccnc(N)n2)c1F	2F-3Cl-6OMe	9.33		
A141	-1.836404324	0.815844417	CC(C)(C)c1nc(-c2cccc([N-]S(=O)(=O)c3c(F)ccc(Br)c3F)c2F)c(-c2ccnc(N)n2)s1	2F-3Br-6F	10.69		
A142	-1.626142502	0.741901517	CC(C)(C)c1nc(-c2cccc([N-]S(=O)(=O)c3c(Cl)ccc(Br)c3F)c2F)c(-c2ccnc(N)n2)s1	2F-3Br-6Cl	10.4		
A143	-1.530934095	0.643092036	CC(C)(C)c1nc(-c2cccc([N-]S(=O)(=O)c3c(Br)ccc(Br)c3F)c2F)c(-c2ccnc(N)n2)s1	2F-3Br-6Br	10.27		
A144	-0.948744655	0.540049136	CC(C)(C)c1nc(-c2cccc([N-]S(=O)(=O)c3c(C(F)(F)F)ccc(Br)c3F)c2F)c(-c2ccnc(N)n2)s1	2F-3Br-6CF3	9.48		
A145	-0.830974758	0.426220477	Cc1ccc(Br)c(F)c1S(=O)(=O)[N-]c1cccc(-c2nc(C(C)(C)C)sc2-c2ccnc(N)n2)c1F	2F-3Br-6Me	9.32		
A146	-0.738414764	0.382146776	CCc1ccc(Br)c(F)c1S(=O)(=O)[N-]c1cccc(-c2nc(C(C)(C)C)sc2-c2ccnc(N)n2)c1F	2F-3Br-6Et	9.19		
A147	-1.025146723	0.386171758	COc1ccc(Br)c(F)c1S(=O)(=O)[N-]c1cccc(-c2nc(C(C)(C)C)sc2-c2ccnc(N)n2)c1F	2F-3Br-6OMe	9.58		
A148	-1.177380323	0.644302368	CC(C)(C)c1nc(-c2cccc([N-]S(=O)(=O)c3c(F)ccc(C(F)(F)F)c3F)c2F)c(-c2ccnc(N)n2)s1	2F-3CF3-6F	9.79		
A149	-1.078109264	0.497546613	CC(C)(C)c1nc(-c2cccc([N-]S(=O)(=O)c3c(Cl)ccc(C(F)(F)F)c3F)c2F)c(-c2ccnc(N)n2)s1	2F-3CF3-6Cl	9.65		
A150	-1.06284225	0.505614996	CC(C)(C)c1nc(-c2cccc([N-]S(=O)(=O)c3c(Br)ccc(C(F)(F)F)c3F)c2F)c(-c2ccnc(N)n2)s1	2F-3CF3-6Br	9.63		
A151	-0.876776576	0.418423146	CC(C)(C)c1nc(-c2cccc([N-]S(=O)(=O)c3c(C(F)(F)F)ccc(C(F)(F)F)c3F)c2F)c(-c2ccnc(N)n2)s1	2F-3CF3-6CF3	9.38		
A152	-1.250125051	0.269781768	Cc1ccc(C(F)(F)F)c(F)c1S(=O)(=O)[N-]c1cccc(-c2nc(C(C)(C)C)sc2-c2ccnc(N)n2)c1F	2F-3CF3-6Me	9.89		
A153	-1.24256587	0.399050444	CCc1ccc(C(F)(F)F)c(F)c1S(=O)(=O)[N-]c1cccc(-c2nc(C(C)(C)C)sc2-c2ccnc(N)n2)c1F	2F-3CF3-6Et	9.88		
A154	-1.040903687	0.295377821	COc1ccc(C(F)(F)F)c(F)c1S(=O)(=O)[N-]c1cccc(-c2nc(C(C)(C)C)sc2-c2ccnc(N)n2)c1F	2F-3CF3-6OMe	9.6		
A155	-1.142104268	0.653118014	Cc1ccc(F)c(S(=O)(=O)[N-]c2cccc(-c3nc(C(C)(C)C)sc3-c3ccnc(N)n3)c2F)c1F	2F-3Me-6F	9.74		
A156	-1.024440765	0.559632182	Cc1ccc(Cl)c(S(=O)(=O)[N-]c2cccc(-c3nc(C(C)(C)C)sc3-c3ccnc(N)n3)c2F)c1F	2F-3Me-6Cl	9.58		
A157	-1.106588244	0.587591648	Cc1ccc(Br)c(S(=O)(=O)[N-]c2cccc(-c3nc(C(C)(C)C)sc3-c3ccnc(N)n3)c2F)c1F	2F-3Me-6Br	9.69		
A158	-0.863725305	0.476647735	Cc1ccc(C(F)(F)F)c(S(=O)(=O)[N-]c2cccc(-c3nc(C(C)(C)C)sc3-c3ccnc(N)n3)c2F)c1F	2F-3Me-6CF3	9.36		
A159	-0.915180802	0.455559134	Cc1ccc(C)c(S(=O)(=O)[N-]c2cccc(-c3nc(C(C)(C)C)sc3-c3ccnc(N)n3)c2F)c1F	2F-3Me-6Me	9.43		
A160	-0.883918762	0.35997963	CCc1ccc(C)c(F)c1S(=O)(=O)[N-]c1cccc(-c2nc(C(C)(C)C)sc2-c2ccnc(N)n2)c1F	2F-3Me-6Et	9.39		
A161	-1.472389698	0.508925676	COc1ccc(C)c(F)c1S(=O)(=O)[N-]c1cccc(-c2nc(C(C)(C)C)sc2-c2ccnc(N)n2)c1F	2F-3Me-6OMe	10.19		
A162	-0.869431853	0.423865974	CCc1ccc(F)c(S(=O)(=O)[N-]c2cccc(-c3nc(C(C)(C)C)sc3-c3ccnc(N)n3)c2F)c1F	2F-3Et-6F	9.37		
A163	-0.736890078	0.480246603	CCc1ccc(Cl)c(S(=O)(=O)[N-]c2cccc(-c3nc(C(C)(C)C)sc3-c3ccnc(N)n3)c2F)c1F	2F-3Et-6Cl	9.19		
A164	-1.282014966	0.557943821	CCc1ccc(Br)c(S(=O)(=O)[N-]c2cccc(-c3nc(C(C)(C)C)sc3-c3ccnc(N)n3)c2F)c1F	2F-3Et-6Br	9.93		
A165	-1.615328074	0.622284055	CCc1ccc(C(F)(F)F)c(S(=O)(=O)[N-]c2cccc(-c3nc(C(C)(C)C)sc3-c3ccnc(N)n3)c2F)c1F	2F-3Et-6CF3	10.39		
A166	-1.028370857	0.396273315	CCc1ccc(C)c(S(=O)(=O)[N-]c2cccc(-c3nc(C(C)(C)C)sc3-c3ccnc(N)n3)c2F)c1F	2F-3Et-6Me	9.59		
A167	-1.123053312	0.436712116	CCc1ccc(CC)c(S(=O)(=O)[N-]c2cccc(-c3nc(C(C)(C)C)sc3-c3ccnc(N)n3)c2F)c1F	2F-3Et-6Et	9.72		
A168	-1.115739107	0.36019963	CCc1ccc(OC)c(S(=O)(=O)[N-]c2cccc(-c3nc(C(C)(C)C)sc3-c3ccnc(N)n3)c2F)c1F	2F-3Et-6OMe	9.71		
A169	-0.875660241	0.407480359	COc1ccc(F)c(S(=O)(=O)[N-]c2cccc(-c3nc(C(C)(C)C)sc3-c3ccnc(N)n3)c2F)c1F	2F-3OMe-6F	9.38		
A170	-1.156520128	0.353554904	COc1ccc(Cl)c(S(=O)(=O)[N-]c2cccc(-c3nc(C(C)(C)C)sc3-c3ccnc(N)n3)c2F)c1F	2F-3OMe-6Cl	9.76		
A171	-1.224252224	0.352455288	COc1ccc(Br)c(S(=O)(=O)[N-]c2cccc(-c3nc(C(C)(C)C)sc3-c3ccnc(N)n3)c2F)c1F	2F-3OMe-6Br	9.85		

A172	-1.203558922	0.432491124	COc1ccc(C(F)(F)F)c(S(=O)(=O)[N-]c2cccc(-c3nc(C(C)(C)C)sc3-c3ccnc(N)n3)c2F)c1F	2F-3OMe-6CF3	9.83		
A173	-1.297985792	0.45364514	COc1ccc(C)c(S(=O)(=O)[N-]c2cccc(-c3nc(C(C)(C)C)sc3-c3ccnc(N)n3)c2F)c1F	2F-3OMe-6Me	9.95		
A174	-0.936043143	0.257084101	CCc1ccc(OC)c(F)c1S(=O)(=O)[N-]c1cccc(-c2nc(C(C)(C)C)sc2-c2ccnc(N)n2)c1F	2F-3OMe-6Et	9.46		
A175	-0.718752623	0.253324926	COc1ccc(OC)c(S(=O)(=O)[N-]c2cccc(-c3nc(C(C)(C)C)sc3-c3ccnc(N)n3)c2F)c1F	2F-3OMe-6OMe	9.16		
A176	-1.127674818	0.56404829	CC(C)(C)c1nc(-c2cccc([N-]S(=O)(=O)c3cc(F)c(F)cc3F)c2F)c(-c2ccnc(N)n2)s1	2F-4F-5F	9.72		
A177	-1.297930241	0.701456904	CC(C)(C)c1nc(-c2cccc([N-]S(=O)(=O)c3cc(Cl)c(F)cc3F)c2F)c(-c2ccnc(N)n2)s1	2F-4F-5Cl	9.95		
A178	-1.553930044	0.758191705	CC(C)(C)c1nc(-c2cccc([N-]S(=O)(=O)c3cc(Br)c(F)cc3F)c2F)c(-c2ccnc(N)n2)s1	2F-4F-5Br	10.3		
A179	-0.699467361	0.493954986	CC(C)(C)c1nc(-c2cccc([N-]S(=O)(=O)c3cc(C(F)(F)F)c(F)cc3F)c2F)c(-c2ccnc(N)n2)s1	2F-4F-5CF3	9.14		
A180	-0.881094337	0.554909587	Cc1cc(S(=O)(=O)[N-]c2cccc(-c3nc(C(C)(C)C)sc3-c3ccnc(N)n3)c2F)c(F)cc1F	2F-4F-5Me	9.39		
A181	-0.87466085	0.478032351	CCc1cc(S(=O)(=O)[N-]c2cccc(-c3nc(C(C)(C)C)sc3-c3ccnc(N)n3)c2F)c(F)cc1F	2F-4F-5Et	9.38		
A182	-0.923005342	0.325794369	COc1cc(S(=O)(=O)[N-]c2cccc(-c3nc(C(C)(C)C)sc3-c3ccnc(N)n3)c2F)c(F)cc1F	2F-4F-5OMe	9.44		
A183	-1.080058336	0.474066943	CC(C)(C)c1nc(-c2cccc([N-]S(=O)(=O)c3cc(F)c(Cl)cc3F)c2F)c(-c2ccnc(N)n2)s1	2F-4Cl-5F	9.66		
A184	-1.298413515	0.566116214	CC(C)(C)c1nc(-c2cccc([N-]S(=O)(=O)c3cc(Cl)c(Cl)cc3F)c2F)c(-c2ccnc(N)n2)s1	2F-4Cl-5Cl	9.96		
A185	-1.474365711	0.646900952	CC(C)(C)c1nc(-c2cccc([N-]S(=O)(=O)c3cc(Br)c(Cl)cc3F)c2F)c(-c2ccnc(N)n2)s1	2F-4Cl-5Br	10.2		
A186	-0.897383571	0.475738525	CC(C)(C)c1nc(-c2cccc([N-]S(=O)(=O)c3cc(C(F)(F)F)c(Cl)cc3F)c2F)c(-c2ccnc(N)n2)s1	2F-4Cl-5CF3	9.41		
A187	-0.480814457	0.402831495	Cc1cc(S(=O)(=O)[N-]c2cccc(-c3nc(C(C)(C)C)sc3-c3ccnc(N)n3)c2F)c(F)cc1Cl	2F-4Cl-5Me	8.84		
A188	-0.769307733	0.463678896	CCc1cc(S(=O)(=O)[N-]c2cccc(-c3nc(C(C)(C)C)sc3-c3ccnc(N)n3)c2F)c(F)cc1Cl	2F-4Cl-5Et	9.23		
A189	-0.812216818	0.294572294	COc1cc(S(=O)(=O)[N-]c2cccc(-c3nc(C(C)(C)C)sc3-c3ccnc(N)n3)c2F)c(F)cc1Cl	2F-4Cl-5OMe	9.29		
A190	-1.267333746	0.578232467	CC(C)(C)c1nc(-c2cccc([N-]S(=O)(=O)c3cc(F)c(Br)cc3F)c2F)c(-c2ccnc(N)n2)s1	2F-4Br-5F	9.91		
A191	-1.430116653	0.596810758	CC(C)(C)c1nc(-c2cccc([N-]S(=O)(=O)c3cc(Cl)c(Br)cc3F)c2F)c(-c2ccnc(N)n2)s1	2F-4Br-5Cl	10.13		
A192	-1.314824104	0.605920136	CC(C)(C)c1nc(-c2cccc([N-]S(=O)(=O)c3cc(Br)c(Br)cc3F)c2F)c(-c2ccnc(N)n2)s1	2F-4Br-5Br	9.98		
A193	-0.819738984	0.481770933	CC(C)(C)c1nc(-c2cccc([N-]S(=O)(=O)c3cc(C(F)(F)F)c(Br)cc3F)c2F)c(-c2ccnc(N)n2)s1	2F-4Br-5CF3	9.3		
A194	-0.464285523	0.377472162	Cc1cc(S(=O)(=O)[N-]c2cccc(-c3nc(C(C)(C)C)sc3-c3ccnc(N)n3)c2F)c(F)cc1Br	2F-4Br-5Me	8.82		
A195	-0.774686635	0.430325747	CCc1cc(S(=O)(=O)[N-]c2cccc(-c3nc(C(C)(C)C)sc3-c3ccnc(N)n3)c2F)c(F)cc1Br	2F-4Br-5Et	9.24		
A196	-1.019637823	0.340033114	COc1cc(S(=O)(=O)[N-]c2cccc(-c3nc(C(C)(C)C)sc3-c3ccnc(N)n3)c2F)c(F)cc1Br	2F-4Br-5OMe	9.57		
A197	-0.65088582	0.356855154	CC(C)(C)c1nc(-c2cccc([N-]S(=O)(=O)c3cc(F)c(C(F)(F)F)cc3F)c2F)c(-c2ccnc(N)n2)s1	2F-4CF3-5F	9.07		
A198	-0.971806049	0.448755145	CC(C)(C)c1nc(-c2cccc([N-]S(=O)(=O)c3cc(Cl)c(C(F)(F)F)cc3F)c2F)c(-c2ccnc(N)n2)s1	2F-4CF3-5Cl	9.51		
A199	-0.293112516	0.189165667	CC(C)(C)c1nc(-c2cccc([N-]S(=O)(=O)c3cc(Br)c(C(F)(F)F)cc3F)c2F)c(-c2ccnc(N)n2)s1	2F-4CF3-5Br	8.58		
A200	-0.547694743	0.233922422	CC(C)(C)c1nc(-c2cccc([N-]S(=O)(=O)c3cc(C(F)(F)F)c(C(F)(F)F)cc3F)c2F)c(-c2ccnc(N)n2)s1	2F-4CF3-5CF3	8.93		
A201	-0.457287341	0.49552384	Cc1cc(S(=O)(=O)[N-]c2cccc(-c3nc(C(C)(C)C)sc3-c3ccnc(N)n3)c2F)c(F)cc1C(F)(F)F	2F-4CF3-5Me	8.81		
A202	-0.637019992	0.190827489	CCc1cc(S(=O)(=O)[N-]c2cccc(-c3nc(C(C)(C)C)sc3-c3ccnc(N)n3)c2F)c(F)cc1C(F)(F)F	2F-4CF3-5Et	9.05		
A203	-0.652140141	0.224532738	COc1cc(S(=O)(=O)[N-]c2cccc(-c3nc(C(C)(C)C)sc3-c3ccnc(N)n3)c2F)c(F)cc1C(F)(F)F	2F-4CF3-5OMe	9.07		
A204	-0.757257104	0.475289702	Cc1cc(F)c(S(=O)(=O)[N-]c2cccc(-c3nc(C(C)(C)C)sc3-c3ccnc(N)n3)c2F)cc1F	2F-4Me-5F	9.22		
A205	-0.912099361	0.538755178	Cc1cc(F)c(S(=O)(=O)[N-]c2cccc(-c3nc(C(C)(C)C)sc3-c3ccnc(N)n3)c2F)cc1Cl	2F-4Me-5Cl	9.43		
A206	-1.063581705	0.494508028	Cc1cc(F)c(S(=O)(=O)[N-]c2cccc(-c3nc(C(C)(C)C)sc3-c3ccnc(N)n3)c2F)cc1Br	2F-4Me-5Br	9.63		
A207	-0.020874143	0.264411151	Cc1cc(F)c(S(=O)(=O)[N-]c2cccc(-c3nc(C(C)(C)C)sc3-c3ccnc(N)n3)c2F)cc1C(F)(F)F	2F-4Me-5CF3	8.21		
A208	-0.723651171	0.452386916	Cc1cc(F)c(S(=O)(=O)[N-]c2cccc(-c3nc(C(C)(C)C)sc3-c3ccnc(N)n3)c2F)cc1C	2F-4Me-5Me	9.17		
A209	-0.967206836	0.40574488	CCc1cc(S(=O)(=O)[N-]c2cccc(-c3nc(C(C)(C)C)sc3-c3ccnc(N)n3)c2F)c(F)cc1C	2F-4Me-5Et	9.5		
A210	-0.980037451	0.432043672	COc1cc(S(=O)(=O)[N-]c2cccc(-c3nc(C(C)(C)C)sc3-c3ccnc(N)n3)c2F)c(F)cc1C	2F-4Me-5OMe	9.52		
A211	-0.692110479	0.400332958	CCc1cc(F)c(S(=O)(=O)[N-]c2cccc(-c3nc(C(C)(C)C)sc3-c3ccnc(N)n3)c2F)cc1F	2F-4Et-5F	9.13		
A212	-0.981428862	0.433038771	CCc1cc(F)c(S(=O)(=O)[N-]c2cccc(-c3nc(C(C)(C)C)sc3-c3ccnc(N)n3)c2F)cc1Cl	2F-4Et-5Cl	9.52		
A213	-0.910120845	0.394945204	CCc1cc(F)c(S(=O)(=O)[N-]c2cccc(-c3nc(C(C)(C)C)sc3-c3ccnc(N)n3)c2F)cc1Br	2F-4Et-5Br	9.43		
A214	-0.856072903	0.326295435	CCc1cc(F)c(S(=O)(=O)[N-]c2cccc(-c3nc(C(C)(C)C)sc3-c3ccnc(N)n3)c2F)cc1C(F)(F)F	2F-4Et-5CF3	9.35		
A215	-1.13750422	0.325127065	CCc1cc(F)c(S(=O)(=O)[N-]c2cccc(-c3nc(C(C)(C)C)sc3-c3ccnc(N)n3)c2F)cc1C	2F-4Et-5Me	9.74		
A216	-0.919015527	0.266438276	CCc1cc(F)c(S(=O)(=O)[N-]c2cccc(-c3nc(C(C)(C)C)sc3-c3ccnc(N)n3)c2F)cc1CC	2F-4Et-5Et	9.44		
A217	-1.055764079	0.356309891	CCc1cc(F)c(S(=O)(=O)[N-]c2cccc(-c3nc(C(C)(C)C)sc3-c3ccnc(N)n3)c2F)cc1OC	2F-4Et-5OMe	9.62		
A218	-0.91484499	0.525895715	COc1cc(F)c(S(=O)(=O)[N-]c2cccc(-c3nc(C(C)(C)C)sc3-c3ccnc(N)n3)c2F)cc1F	2F-4OMe-5F	9.43		
A219	-1.113333106	0.570529044	COc1cc(F)c(S(=O)(=O)[N-]c2cccc(-c3nc(C(C)(C)C)sc3-c3ccnc(N)n3)c2F)cc1Cl	2F-4OMe-5Cl	9.7		
A220	-1.046345711	0.501813948	COc1cc(F)c(S(=O)(=O)[N-]c2cccc(-c3nc(C(C)(C)C)sc3-c3ccnc(N)n3)c2F)cc1Br	2F-4OMe-5Br	9.61		
A221	-0.933402419	0.382757008	COc1cc(F)c(S(=O)(=O)[N-]c2cccc(-c3nc(C(C)(C)C)sc3-c3ccnc(N)n3)c2F)cc1C(F)(F)F	2F-4OMe-5CF3	9.46		
A222	-0.774953485	0.463386357	COc1cc(F)c(S(=O)(=O)[N-]c2cccc(-c3nc(C(C)(C)C)sc3-c3ccnc(N)n3)c2F)cc1C	2F-4OMe-5Me	9.24		
A223	-1.104726791	0.367402732	CCc1cc(S(=O)(=O)[N-]c2cccc(-c3nc(C(C)(C)C)sc3-c3ccnc(N)n3)c2F)c(F)cc1OC	2F-4OMe-5Et	9.69		
A224	-0.673784375	0.467992246	COc1cc(F)c(S(=O)(=O)[N-]c2cccc(-c3nc(C(C)(C)C)sc3-c3ccnc(N)n3)c2F)cc1OC	2F-4OMe-5OMe	9.1		
A225	-1.252012014	0.718527675	CC(C)(C)c1nc(-c2cccc([N-]S(=O)(=O)c3c(F)cc(F)cc3F)c2F)c(-c2ccnc(N)n2)s1	2F-4F-6F	9.89		
A226	-1.234731674	0.677884221	CC(C)(C)c1nc(-c2cccc([N-]S(=O)(=O)c3c(F)cc(F)cc3Cl)c2F)c(-c2ccnc(N)n2)s1	2F-4F-6Cl	9.87		
A227	-1.380754948	0.62329644	CC(C)(C)c1nc(-c2cccc([N-]S(=O)(=O)c3c(F)cc(F)cc3Br)c2F)c(-c2ccnc(N)n2)s1	2F-4F-6Br	10.07		
A228	-0.976951003	0.610364795	CC(C)(C)c1nc(-c2cccc([N-]S(=O)(=O)c3c(F)cc(F)cc3C(F)(F)F)c2F)c(-c2ccnc(N)n2)s1	2F-4F-6CF3	9.52		
A229	-0.795662224	0.49549067	Cc1cc(F)cc(F)c1S(=O)(=O)[N-]c1cccc(-c2nc(C(C)(C)C)sc2-c2ccnc(N)n2)c1F	2F-4F-6Me	9.27		
A230	-0.97042805	0.666332841	CCc1cc(F)cc(F)c1S(=O)(=O)[N-]c1cccc(-c2nc(C(C)(C)C)sc2-c2ccnc(N)n2)c1F	2F-4F-6Et	9.51		
A231	-0.964242101	0.440476149	COc1cc(F)cc(F)c1S(=O)(=O)[N-]c1cccc(-c2nc(C(C)(C)C)sc2-c2ccnc(N)n2)c1F	2F-4F-6OMe	9.5		
A232	-1.337233305	0.58884418	CC(C)(C)c1nc(-c2cccc([N-]S(=O)(=O)c3c(F)cc(Cl)cc3F)c2F)c(-c2ccnc(N)n2)s1	2F-4Cl-6F	10.01		
A233	-1.491687655	0.623687625	CC(C)(C)c1nc(-c2cccc([N-]S(=O)(=O)c3c(F)cc(Cl)cc3Cl)c2F)c(-c2ccnc(N)n2)s1	2F-4Cl-6Cl	10.22		
A234	-1.365446806	0.541373312	CC(C)(C)c1nc(-c2cccc([N-]S(=O)(=O)c3c(F)cc(Cl)cc3Br)c2F)c(-c2ccnc(N)n2)s1	2F-4Cl-6Br	10.05		
A235	-0.790368199	0.496496499	CC(C)(C)c1nc(-c2cccc([N-]S(=O)(=O)c3c(F)cc(Cl)cc3C(F)(F)F)c2F)c(-c2ccnc(N)n2)s1	2F-4Cl-6CF3	9.26		
A236	-0.257505536	0.386384189	Cc1cc(Cl)cc(F)c1S(=O)(=O)[N-]c1cccc(-c2nc(C(C)(C)C)sc2-c2ccnc(N)n2)c1F	2F-4Cl-6Me	8.54		
A237	-0.577074766	0.425217748	CCc1cc(Cl)cc(F)c1S(=O)(=O)[N-]c1cccc(-c2nc(C(C)(C)C)sc2-c2ccnc(N)n2)c1F	2F-4Cl-6Et	8.97		
A238	-0.94159013	0.434823632	COc1cc(Cl)cc(F)c1S(=O)(=O)[N-]c1cccc(-c2nc(C(C)(C)C)sc2-c2ccnc(N)n2)c1F	2F-4Cl-6OMe	9.47		
A239	-1.403231621	0.548121572	CC(C)(C)c1nc(-c2cccc([N-]S(=O)(=O)c3c(F)cc(Br)cc3F)c2F)c(-c2ccnc(N)n2)s1	2F-4Br-6F	10.1		
A240	-1.244431496	0.545879841	CC(C)(C)c1nc(-c2cccc([N-]S(=O)(=O)c3c(F)cc(Br)cc3Cl)c2F)c(-c2ccnc(N)n2)s1	2F-4Br-6Cl	9.88		
A241	-1.305140972	0.540738583	CC(C)(C)c1nc(-c2cccc([N-]S(=O)(=O)c3c(F)cc(Br)cc3Br)c2F)c(-c2ccnc(N)n2)s1	2F-4Br-6Br	9.96		
A242	-0.740675509	0.496362597	CC(C)(C)c1nc(-c2cccc([N-]S(=O)(=O)c3c(F)cc(Br)cc3C(F)(F)F)c2F)c(-c2ccnc(N)n2)s1	2F-4Br-6CF3	9.19		
A243	-0.544566214	0.326422095	Cc1cc(Br)cc(F)c1S(=O)(=O)[N-]c1cccc(-c2nc(C(C)(C)C)sc2-c2ccnc(N)n2)c1F	2F-4Br-6Me	8.93		
A244	-0.764481544	0.324718475	CCc1cc(Br)cc(F)c1S(=O)(=O)[N-]c1cccc(-c2nc(C(C)(C)C)sc2-c2ccnc(N)n2)c1F	2F-4Br-6Et	9.23		
A245	-0.918046355	0.376384616	COc1cc(Br)cc(F)c1S(=O)(=O)[N-]c1cccc(-c2nc(C(C)(C)C)sc2-c2ccnc(N)n2)c1F	2F-4Br-6OMe	9.44		
A246	-0.435979456	0.300144702	CC(C)(C)c1nc(-c2cccc([N-]S(=O)(=O)c3c(F)cc(C(F)(F)F)cc3F)c2F)c(-c2ccnc(N)n2)s1	2F-4CF3-6F	8.78		
A247	-0.336098462	0.26362282	CC(C)(C)c1nc(-c2cccc([N-]S(=O)(=O)c3c(F)cc(C(F)(F)F)cc3Cl)c2F)c(-c2ccnc(N)n2)s1	2F-4CF3-6Cl	8.64		
A248	-0.335378647	0.279892892	CC(C)(C)c1nc(-c2cccc([N-]S(=O)(=O)c3c(F)cc(C(F)(F)F)cc3Br)c2F)c(-c2ccnc(N)n2)s1	2F-4CF3-6Br	8.64		
A249	-0.304702282	0.276110768	CC(C)(C)c1nc(-c2cccc([N-]S(=O)(=O)c3c(F)cc(C(F)(F)F)cc3C(F)(F)F)c2F)c(-c2ccnc(N)n2)s1	2F-4CF3-6CF3	8.6		
A250	-0.409229249	0.292440236	Cc1cc(C(F)(F)F)cc(F)c1S(=O)(=O)[N-]c1cccc(-c2nc(C(C)(C)C)sc2-c2ccnc(N)n2)c1F	2F-4CF3-6Me	8.74		
A251	-0.642159343	0.190728635	CCc1cc(C(F)(F)F)cc(F)c1S(=O)(=O)[N-]c1cccc(-c2nc(C(C)(C)C)sc2-c2ccnc(N)n2)c1F	2F-4CF3-6Et	9.06		
A252	-0.807316184	0.193543047	COc1cc(C(F)(F)F)cc(F)c1S(=O)(=O)[N-]c1cccc(-c2nc(C(C)(C)C)sc2-c2ccnc(N)n2)c1F	2F-4CF3-6OMe	9.29		
A253	-0.216545835	0.41875273	Cc1cc(F)c(S(=O)(=O)[N-]c2cccc(-c3nc(C(C)(C)C)sc3-c3ccnc(N)n3)c2F)c(F)c1	2F-4Me-6F	8.48	9f	150
A254	-0.671637118	0.410981089	Cc1cc(F)c(S(=O)(=O)[N-]c2cccc(-c3nc(C(C)(C)C)sc3-c3ccnc(N)n3)c2F)c(Cl)c1	2F-4Me-6Cl	9.1		
A255	-0.454225928	0.376316756	Cc1cc(F)c(S(=O)(=O)[N-]c2cccc(-c3nc(C(C)(C)C)sc3-c3ccnc(N)n3)c2F)c(Br)c1	2F-4Me-6Br	8.8		
A256	-0.804224789	0.509474158	Cc1cc(F)c(S(=O)(=O)[N-]c2cccc(-c3nc(C(C)(C)C)sc3-c3ccnc(N)n3)c2F)c(C(F)(F)F)c1	2F-4Me-6CF3	9.28		
A257	-0.716011643	0.372999489	Cc1cc(C)c(S(=O)(=O)[N-]c2cccc(-c3nc(C(C)(C)C)sc3-c3ccnc(N)n3)c2F)c(F)c1	2F-4Me-6Me	9.16	9g	212
A258	-1.020361662	0.396427721	CCc1cc(C)cc(F)c1S(=O)(=O)[N-]c1cccc(-c2nc(C(C)(C)C)sc2-c2ccnc(N)n2)c1F	2F-4Me-6Et	9.58		
A259	-1.290961742	0.441809714	COc1cc(C)cc(F)c1S(=O)(=O)[N-]c1cccc(-c2nc(C(C)(C)C)sc2-c2ccnc(N)n2)c1F	2F-4Me-6OMe	9.94		

A260	-0.882297218	0.394409418	CCc1cc(F)c(S(=O)(=O)[N-]c2cccc(-c3nc(C(C)(C)C)sc3-c3ccnc(N)n3)c2F)c(F)c1	2F-4Et-6F	9.39		
A261	-0.600244462	0.311167479	CCc1cc(F)c(S(=O)(=O)[N-]c2cccc(-c3nc(C(C)(C)C)sc3-c3ccnc(N)n3)c2F)c(Cl)c1	2F-4Et-6Cl	9		
A262	-0.678903401	0.337021351	CCc1cc(F)c(S(=O)(=O)[N-]c2cccc(-c3nc(C(C)(C)C)sc3-c3ccnc(N)n3)c2F)c(Br)c1	2F-4Et-6Br	9.11		
A263	-0.675358951	0.314962983	CCc1cc(F)c(S(=O)(=O)[N-]c2cccc(-c3nc(C(C)(C)C)sc3-c3ccnc(N)n3)c2F)c(C(F)(F)F)c1	2F-4Et-6CF3	9.11		
A264	-0.86889106	0.260954499	CCc1cc(C)c(S(=O)(=O)[N-]c2cccc(-c3nc(C(C)(C)C)sc3-c3ccnc(N)n3)c2F)c(F)c1	2F-4Et-6Me	9.37		
A265	-0.912302315	0.229449183	CCc1cc(F)c(S(=O)(=O)[N-]c2cccc(-c3nc(C(C)(C)C)sc3-c3ccnc(N)n3)c2F)c(CC)c1	2F-4Et-6Et	9.43		
A266	-1.001969099	0.311701119	CCc1cc(F)c(S(=O)(=O)[N-]c2cccc(-c3nc(C(C)(C)C)sc3-c3ccnc(N)n3)c2F)c(OC)c1	2F-4Et-6OMe	9.55		
A267	-0.805224419	0.431245267	COc1cc(F)c(S(=O)(=O)[N-]c2cccc(-c3nc(C(C)(C)C)sc3-c3ccnc(N)n3)c2F)c(F)c1	2F-4OMe-6F	9.28		
A268	-0.95575726	0.415213227	COc1cc(F)c(S(=O)(=O)[N-]c2cccc(-c3nc(C(C)(C)C)sc3-c3ccnc(N)n3)c2F)c(Cl)c1	2F-4OMe-6Cl	9.49		
A269	-0.982569396	0.417631894	COc1cc(F)c(S(=O)(=O)[N-]c2cccc(-c3nc(C(C)(C)C)sc3-c3ccnc(N)n3)c2F)c(Br)c1	2F-4OMe-6Br	9.52		
A270	-0.890664518	0.473253131	COc1cc(F)c(S(=O)(=O)[N-]c2cccc(-c3nc(C(C)(C)C)sc3-c3ccnc(N)n3)c2F)c(C(F)(F)F)c1	2F-4OMe-6CF3	9.4		
A271	-0.940862656	0.450022429	COc1cc(C)c(S(=O)(=O)[N-]c2cccc(-c3nc(C(C)(C)C)sc3-c3ccnc(N)n3)c2F)c(F)c1	2F-4OMe-6Me	9.47		
A272	-0.934981227	0.40480122	CCc1cc(OC)cc(F)c1S(=O)(=O)[N-]c1cccc(-c2nc(C(C)(C)C)sc2-c2ccnc(N)n2)c1F	2F-4OMe-6Et	9.46		
A273	-0.654910207	0.364172876	COc1cc(F)c(S(=O)(=O)[N-]c2cccc(-c3nc(C(C)(C)C)sc3-c3ccnc(N)n3)c2F)c(OC)c1	2F-4OMe-6OMe	9.08		
A274	-1.558275342	0.806669712	CC(C)(C)c1nc(-c2cccc([N-]S(=O)(=O)c3c(F)ccc(F)c3F)c2F)c(-c2ccnc(N)n2)s1	2F-5F-6F	10.31		
A275	-1.409639597	0.746519327	CC(C)(C)c1nc(-c2cccc([N-]S(=O)(=O)c3c(F)ccc(F)c3Cl)c2F)c(-c2ccnc(N)n2)s1	2F-5F-6Cl	10.11		
A276	-1.456443191	0.713342905	CC(C)(C)c1nc(-c2cccc([N-]S(=O)(=O)c3c(F)ccc(F)c3Br)c2F)c(-c2ccnc(N)n2)s1	2F-5F-6Br	10.17		
A277	-1.138404608	0.691867828	CC(C)(C)c1nc(-c2cccc([N-]S(=O)(=O)c3c(F)ccc(F)c3C(F)(F)F)c2F)c(-c2ccnc(N)n2)s1	2F-5F-6CF3	9.74		
A278	-0.569439769	0.566086352	Cc1c(F)ccc(F)c1S(=O)(=O)[N-]c1cccc(-c2nc(C(C)(C)C)sc2-c2ccnc(N)n2)c1F	2F-5F-6Me	8.96		
A279	-0.963592172	0.465873897	CCc1c(F)ccc(F)c1S(=O)(=O)[N-]c1cccc(-c2nc(C(C)(C)C)sc2-c2ccnc(N)n2)c1F	2F-5F-6Et	9.5		
A280	-1.214039922	0.547357976	COc1c(F)ccc(F)c1S(=O)(=O)[N-]c1cccc(-c2nc(C(C)(C)C)sc2-c2ccnc(N)n2)c1F	2F-5F-6OMe	9.84		
A281	-1.547826171	0.750472486	CC(C)(C)c1nc(-c2cccc([N-]S(=O)(=O)c3c(F)ccc(Cl)c3F)c2F)c(-c2ccnc(N)n2)s1	2F-5Cl-6F	10.3		
A282	-1.528552771	0.705239773	CC(C)(C)c1nc(-c2cccc([N-]S(=O)(=O)c3c(F)ccc(Cl)c3Cl)c2F)c(-c2ccnc(N)n2)s1	2F-5Cl-6Cl	10.27	9e	68
A283	-1.568716288	0.700372696	CC(C)(C)c1nc(-c2cccc([N-]S(=O)(=O)c3c(F)ccc(Cl)c3Br)c2F)c(-c2ccnc(N)n2)s1	2F-5Cl-6Br	10.32		
A284	-1.347280025	0.74148047	CC(C)(C)c1nc(-c2cccc([N-]S(=O)(=O)c3c(F)ccc(Cl)c3C(F)(F)F)c2F)c(-c2ccnc(N)n2)s1	2F-5Cl-6CF3	10.02		
A285	-0.932169318	0.544311404	Cc1c(Cl)ccc(F)c1S(=O)(=O)[N-]c1cccc(-c2nc(C(C)(C)C)sc2-c2ccnc(N)n2)c1F	2F-5Cl-6Me	9.46		
A286	-1.020665526	0.382683218	CCc1c(Cl)ccc(F)c1S(=O)(=O)[N-]c1cccc(-c2nc(C(C)(C)C)sc2-c2ccnc(N)n2)c1F	2F-5Cl-6Et	9.58		
A287	-1.066021323	0.50240159	COc1c(Cl)ccc(F)c1S(=O)(=O)[N-]c1cccc(-c2nc(C(C)(C)C)sc2-c2ccnc(N)n2)c1F	2F-5Cl-6OMe	9.64		
A288	-1.896467447	0.81963408	CC(C)(C)c1nc(-c2cccc([N-]S(=O)(=O)c3c(F)ccc(Br)c3F)c2F)c(-c2ccnc(N)n2)s1	2F-5Br-6F	10.77		
A289	-1.703855038	0.751480579	CC(C)(C)c1nc(-c2cccc([N-]S(=O)(=O)c3c(F)ccc(Br)c3Cl)c2F)c(-c2ccnc(N)n2)s1	2F-5Br-6Cl	10.51		
A290	-1.773207664	0.734500647	CC(C)(C)c1nc(-c2cccc([N-]S(=O)(=O)c3c(F)ccc(Br)c3Br)c2F)c(-c2ccnc(N)n2)s1	2F-5Br-6Br	10.6		
A291	-0.701935291	0.570534229	CC(C)(C)c1nc(-c2cccc([N-]S(=O)(=O)c3c(F)ccc(Br)c3C(F)(F)F)c2F)c(-c2ccnc(N)n2)s1	2F-5Br-6CF3	9.14		
A292	-0.42784369	0.302307546	Cc1c(Br)ccc(F)c1S(=O)(=O)[N-]c1cccc(-c2nc(C(C)(C)C)sc2-c2ccnc(N)n2)c1F	2F-5Br-6Me	8.77		
A293	-1.209584355	0.564284563	CCc1c(Br)ccc(F)c1S(=O)(=O)[N-]c1cccc(-c2nc(C(C)(C)C)sc2-c2ccnc(N)n2)c1F	2F-5Br-6Et	9.83		
A294	-0.923364997	0.293529451	COc1c(Br)ccc(F)c1S(=O)(=O)[N-]c1cccc(-c2nc(C(C)(C)C)sc2-c2ccnc(N)n2)c1F	2F-5Br-6OMe	9.44		
A295	-1.196701288	0.676028788	CC(C)(C)c1nc(-c2cccc([N-]S(=O)(=O)c3c(F)ccc(C(F)(F)F)c3F)c2F)c(-c2ccnc(N)n2)s1	2F-5CF3-6F	9.82		
A296	-1.281574368	0.701301932	CC(C)(C)c1nc(-c2cccc([N-]S(=O)(=O)c3c(F)ccc(C(F)(F)F)c3Cl)c2F)c(-c2ccnc(N)n2)s1	2F-5CF3-6Cl	9.93		
A297	-1.15859282	0.683473349	CC(C)(C)c1nc(-c2cccc([N-]S(=O)(=O)c3c(F)ccc(C(F)(F)F)c3Br)c2F)c(-c2ccnc(N)n2)s1	2F-5CF3-6Br	9.76		
A298	-0.922318578	0.588079929	CC(C)(C)c1nc(-c2cccc([N-]S(=O)(=O)c3c(F)ccc(C(F)(F)F)c3C(F)(F)F)c2F)c(-c2ccnc(N)n2)s1	2F-5CF3-6CF3	9.44		
A299	-0.46194303	0.458093762	Cc1c(C(F)(F)F)ccc(F)c1S(=O)(=O)[N-]c1cccc(-c2nc(C(C)(C)C)sc2-c2ccnc(N)n2)c1F	2F-5CF3-6Me	8.81		
A300	-0.878885329	0.489208192	CCc1c(C(F)(F)F)ccc(F)c1S(=O)(=O)[N-]c1cccc(-c2nc(C(C)(C)C)sc2-c2ccnc(N)n2)c1F	2F-5CF3-6Et	9.38		
A301	-1.055866599	0.421622992	COc1c(C(F)(F)F)ccc(F)c1S(=O)(=O)[N-]c1cccc(-c2nc(C(C)(C)C)sc2-c2ccnc(N)n2)c1F	2F-5CF3-6OMe	9.62		
A302	-1.10909164	0.637207508	Cc1ccc(F)c(S(=O)(=O)[N-]c2cccc(-c3nc(C(C)(C)C)sc3-c3ccnc(N)n3)c2F)c1F	2F-5Me-6F	9.7		
A303	-0.885946035	0.575161994	Cc1ccc(F)c(S(=O)(=O)[N-]c2cccc(-c3nc(C(C)(C)C)sc3-c3ccnc(N)n3)c2F)c1Cl	2F-5Me-6Cl	9.39		
A304	-1.075514555	0.557391047	Cc1ccc(F)c(S(=O)(=O)[N-]c2cccc(-c3nc(C(C)(C)C)sc3-c3ccnc(N)n3)c2F)c1Br	2F-5Me-6Br	9.65		
A305	-0.979624093	0.605468452	Cc1ccc(F)c(S(=O)(=O)[N-]c2cccc(-c3nc(C(C)(C)C)sc3-c3ccnc(N)n3)c2F)c1C(F)(F)F	2F-5Me-6CF3	9.52		
A306	-0.990301669	0.640447259	Cc1ccc(F)c(S(=O)(=O)[N-]c2cccc(-c3nc(C(C)(C)C)sc3-c3ccnc(N)n3)c2F)c1C	2F-5Me-6Me	9.53		
A307	-1.2230165	0.459498286	CCc1c(C)ccc(F)c1S(=O)(=O)[N-]c1cccc(-c2nc(C(C)(C)C)sc2-c2ccnc(N)n2)c1F	2F-5Me-6Et	9.85		
A308	-1.184168696	0.481244922	COc1c(C)ccc(F)c1S(=O)(=O)[N-]c1cccc(-c2nc(C(C)(C)C)sc2-c2ccnc(N)n2)c1F	2F-5Me-6OMe	9.8		
A309	-0.787284553	0.450495094	CCc1ccc(F)c(S(=O)(=O)[N-]c2cccc(-c3nc(C(C)(C)C)sc3-c3ccnc(N)n3)c2F)c1F	2F-5Et-6F	9.26		
A310	-0.766325474	0.427820325	CCc1ccc(F)c(S(=O)(=O)[N-]c2cccc(-c3nc(C(C)(C)C)sc3-c3ccnc(N)n3)c2F)c1Cl	2F-5Et-6Cl	9.23		
A311	-0.747244954	0.403087765	CCc1ccc(F)c(S(=O)(=O)[N-]c2cccc(-c3nc(C(C)(C)C)sc3-c3ccnc(N)n3)c2F)c1Br	2F-5Et-6Br	9.2		
A312	-0.819203258	0.374753743	CCc1ccc(F)c(S(=O)(=O)[N-]c2cccc(-c3nc(C(C)(C)C)sc3-c3ccnc(N)n3)c2F)c1C(F)(F)F	2F-5Et-6CF3	9.3		
A313	-1.186016321	0.477286041	CCc1ccc(F)c(S(=O)(=O)[N-]c2cccc(-c3nc(C(C)(C)C)sc3-c3ccnc(N)n3)c2F)c1C	2F-5Et-6Me	9.8		
A314	-1.161473155	0.358897746	CCc1ccc(F)c(S(=O)(=O)[N-]c2cccc(-c3nc(C(C)(C)C)sc3-c3ccnc(N)n3)c2F)c1CC	2F-5Et-6Et	9.77		
A315	-1.103169322	0.297013879	CCc1ccc(F)c(S(=O)(=O)[N-]c2cccc(-c3nc(C(C)(C)C)sc3-c3ccnc(N)n3)c2F)c1OC	2F-5Et-6OMe	9.69		
A316	-0.812433958	0.408203244	COc1ccc(F)c(S(=O)(=O)[N-]c2cccc(-c3nc(C(C)(C)C)sc3-c3ccnc(N)n3)c2F)c1F	2F-5OMe-6F	9.29		
A317	-0.951696038	0.426761925	COc1ccc(F)c(S(=O)(=O)[N-]c2cccc(-c3nc(C(C)(C)C)sc3-c3ccnc(N)n3)c2F)c1Cl	2F-5OMe-6Cl	9.48		
A318	-1.191705227	0.444043875	COc1ccc(F)c(S(=O)(=O)[N-]c2cccc(-c3nc(C(C)(C)C)sc3-c3ccnc(N)n3)c2F)c1Br	2F-5OMe-6Br	9.81		
A319	-0.89574337	0.416701883	COc1ccc(F)c(S(=O)(=O)[N-]c2cccc(-c3nc(C(C)(C)C)sc3-c3ccnc(N)n3)c2F)c1C(F)(F)F	2F-5OMe-6CF3	9.41		
A320	-1.086098671	0.362473845	COc1ccc(F)c(S(=O)(=O)[N-]c2cccc(-c3nc(C(C)(C)C)sc3-c3ccnc(N)n3)c2F)c1C	2F-5OMe-6Me	9.67		
A321	-1.100142002	0.316157728	CCc1c(OC)ccc(F)c1S(=O)(=O)[N-]c1cccc(-c2nc(C(C)(C)C)sc2-c2ccnc(N)n2)c1F	2F-5OMe-6Et	9.68		
A322	-0.493885279	0.323934436	COc1ccc(F)c(S(=O)(=O)[N-]c2cccc(-c3nc(C(C)(C)C)sc3-c3ccnc(N)n3)c2F)c1OC	2F-5OMe-6OMe	8.86		

Table S4. profiling of dabrafenib and compound **5a** against 58 diverse kinases at Eurofins. Kinases that were inhibited by greater than 50% are highlighted in red.

Kinase	% kinase activity remaining with 1 μM dabrafenib	% kinase activity remaining with 1 μM compound 5a
Abl(h)	64	102
ALK(h)	100	92
AMPKα1(h)	71	99
ASK1(h)	104	101
Aurora-A(h)	7	99
CaMKI(h)	20	48
CDK1/cyclinB(h)	84	95
CDK2/cyclinA(h)	90	88
CDK6/cyclinD3(h)	93	102
CDK7/cyclinH/MAT1(h)	96	101
CDK9/cyclin T1(h)	88	91
CHK1(h)	109	108
CK1γ1(h)	86	88
CK2α2(h)	97	77
c-RAF(h)	32	75
DRAK1(h)	110	66
eEF-2K(h)	79	87
EGFR(h)	87	97
EphA5(h)	92	96
EphB4(h)	111	102
Fyn(h)	20	115
GSK3β(h)	98	90
IGF-1R(h)	105	116
IKKα(h)	111	110
IRAK4(h)	89	84
JAK2(h)	32	107
KDR(h)	75	107
LOK(h)	33	101
Lyn(h)	62	86
MAPKAP-K2(h)	106	102
MEK1(h)	81	98
MLK1(h)	19	80
Mnk2(h)	84	94
MSK2(h)	99	108
MST1(h)	74	90
mTOR(h)	115	77
NEK2(h)	70	76
p70S6K(h)	94	82
PAK2(h)	83	86
PDGFRβ(h)	71	94
Pim-1(h)	83	100
PKA(h)	88	91
PKBα(h)	99	97
PKCα(h)	89	110
PKCθ(h)	95	97
PKG1α(h)	101	117
Plk3(h)	97	91
PRAK(h)	101	105
ROCK-I(h)	89	94
Rse(h)	108	103
Rsk1(h)	70	108
SAPK2a(h)	92	97
SRPK1(h)	98	92
TAK1(h)	88	92
PI3 Kinase (p110β/p85α)(h)	102	101
PI3 Kinase (p120γ)(h)	107	103
PI3 Kinase (p110δ/p85α)(h)	99	99
PI3 Kinase (p110α/p85α)(h)	96	102

Additional Boltz2 information

Boltz2 predictions were carried out following published prediction instructions (https://github.com/jwohlwend/boltz/blob/main/docs/prediction.md). A representative input file in .yaml format is included in the supporting information file. Specifically, the protein component of the complex is PKR residues 250 – 543, the ligand component is the kinase inhibitor in SMILES format, and the property calculated is affinity. No additional constraints or templates were included. Flags requested for prediction were "—use_potentials", and "recycling_steps 10". All other settings were set as default. A single run was carried out for each compound. Known non-binding compounds encorafenib, plx7904, xp-102, and lxh254 served as negative control references. Inhibitors included in screens are listed in table S1 – S3.

In screening a kinase inhibitor library against PKR using Boltz2, we identified a series of hits from which four of four selected for validation displayed nanomolar potency against PKR *in vitro*. To assess how well Boltz2 discriminated between potential binders and non-binders in our data set, we carried out a receiver operating characteristic curve (ROC) analysis (as in PMID 41592323) of the four validated hits (PIK-75, gilteritinib, A-443654, and dabrafenib) and four non-binder controls (encorafenib, PLX7904, XP-102, and LXH254) using their predicted probability binary scores, which Boltz2 uses to distinguishing binders from decoys in screens (https://github.com/jwohlwend/boltz/blob/main/docs/prediction.md).

The validated binders displayed predicted probability binary scores of 0.95, 0.8, 0.91, and 0.77 for PIK75, gilteritinib, A-443654, and dabrafenib respectively, while the non-binder controls displayed scores of 0.54, 0.40, 0.28, and 0.77 for encorafenib, plx7904, xp-102, and lxh254, respectively. Across all 16 pairs between positive and negative binders, the positive binders scored higher than the non-binder controls in 15 pairs and tied in 1 pair, giving an area under the curve (AUC) value of 0.97. This analysis provides support that Boltz2 scoring can successfully discriminate between binders and non-binders in our screening experiment (Plot below).

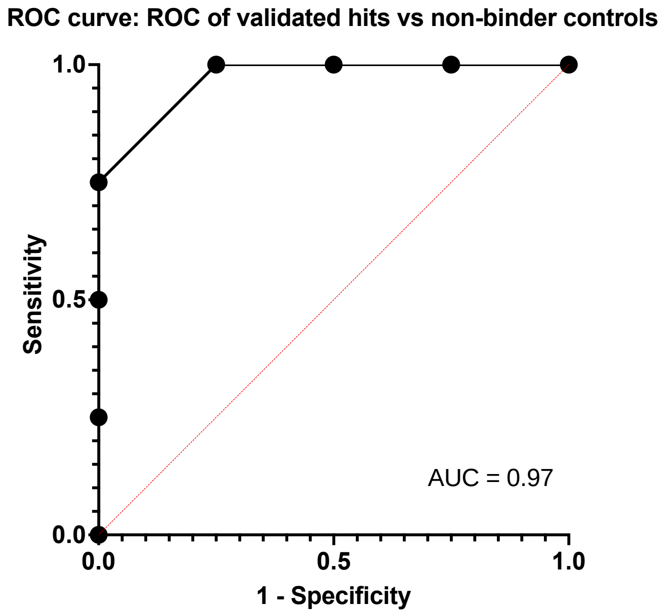

In our inhibitor optimization studies, we calculated Boltz2 scores for 14 molecules that we prioritized for synthesis based on visual inspection of docked poses and available starting materials (sulfonamide analogs 9a – 9g and pyrimidine analogs 5a to 5f, 14 molecules in total). The measured IC_{50} values determined *in vitro* and the Boltz2 predicted binding affinity showed weak correlation with an R^2 value of 0.3 (Plot below). Hence, the utility of Boltz2 as a prospective design tool for SAR proved limited in our application.

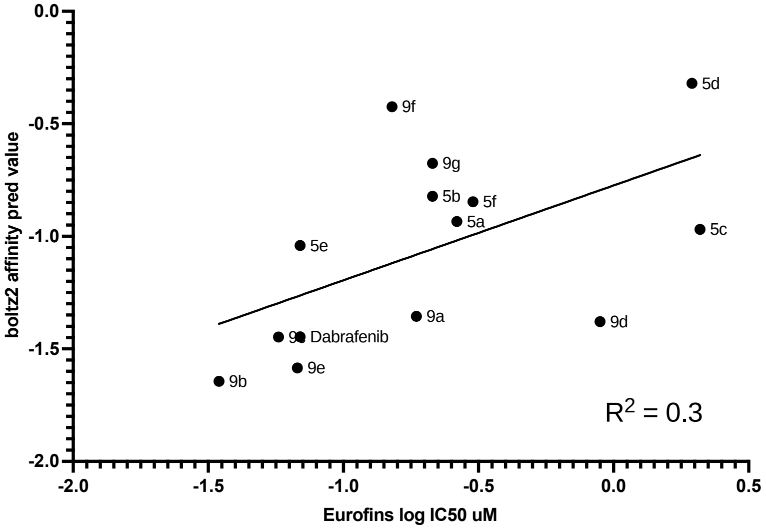

Representative input file format for Boltz2 predicting binding of PKR kinase domain to PIK75

```
version: 1

sequences:

  - protein:

     id: A

     sequence:
PDMKETKYTVDKRFGMDFKEIELIGSGGFGQVFKAKHRIDGKTYVIKRVKYNNEKAEREVKALAKLDHVNIVHYNGCWDGFDYDPE
TSDDSLESSDYDPENSKNSSRSKTKCLFIQMEFCDKGTLEQWIEKRRGEKLDKVLALELFEQITKGVDYIHSKKLIHRDLKPSNIFLVDT
KQVKIGDFGLVTSLKNDGKRTRSKGTLRYMSPEQISSQDYGKEVDLYALGLILAELLHVCDTAFETSKFFTDLRDGIISDIFDKKEKTLL
QKLLSKKPEDRPNTSEILRTLTVWKKSP

     msa: path_to_msa

  - ligand:

     id: B

     smiles: 'CC1=C(S(=O)(=O)N(C)N=CC2=CN=C3C=CC(Br)=CN23)C=C([N+](=O)[O-])C=C1'


properties:

  - affinity:

     binder: B
```
